# Sustained effectiveness and cost-effectiveness of Counselling for Alcohol Problems, a brief psychological treatment for harmful drinking in men, delivered by lay counsellors in primary care: 12-month follow-up of a randomised controlled trial

**DOI:** 10.1371/journal.pmed.1002386

**Published:** 2017-09-12

**Authors:** Abhijit Nadkarni, Helen A. Weiss, Benedict Weobong, David McDaid, Daisy R. Singla, A-La Park, Bhargav Bhat, Basavaraj Katti, Jim McCambridge, Pratima Murthy, Michael King, G. Terence Wilson, Betty Kirkwood, Christopher G. Fairburn, Richard Velleman, Vikram Patel

**Affiliations:** 1 Sangath, Socorro, Goa, India; 2 London School of Hygiene & Tropical Medicine, London, United Kingdom; 3 Personal Social Services Research Unit, London School of Economics and Political Science, London, United Kingdom; 4 Department of Psychiatry, Sinai Health Network, University of Toronto, Toronto, Ontario, Canada; 5 Department of Health Sciences, University of York, York, United Kingdom; 6 National Institute of Mental Health and Neurosciences, Bengaluru, India; 7 Division of Psychiatry, University College London, London, United Kingdom; 8 Department of Psychology, School of Arts and Sciences, Rutgers University, New Brunswick, New Jersey, United States of America; 9 Department of Psychiatry, University of Oxford, Oxford, United Kingdom; 10 Department of Psychology, University of Bath, Bath, United Kingdom; 11 Department of Global Health and Social Medicine, Harvard Medical School, Boston, Massachusetts, United States of America; Massachusetts General Hospital, UNITED STATES

## Abstract

**Background:**

Counselling for Alcohol Problems (CAP), a brief intervention delivered by lay counsellors, enhanced remission and abstinence over 3 months among male primary care attendees with harmful drinking in a setting in India. We evaluated the sustainability of the effects after treatment termination, the cost-effectiveness of CAP over 12 months, and the effects of the hypothesized mediator ‘readiness to change’ on clinical outcomes.

**Methods and findings:**

Male primary care attendees aged 18–65 years screening with harmful drinking on the Alcohol Use Disorders Identification Test (AUDIT) were randomised to either CAP plus enhanced usual care (EUC) (*n =* 188) or EUC alone (*n =* 189), of whom 89% completed assessments at 3 months, and 84% at 12 months. Primary outcomes were remission and mean standard ethanol consumed in the past 14 days, and the proposed mediating variable was readiness to change at 3 months. CAP participants maintained the gains they showed at the end of treatment through the 12-month follow-up, with the proportion with remission (AUDIT score < 8: 54.3% versus 31.9%; adjusted prevalence ratio [aPR] 1.71 [95% CI 1.32, 2.22]; *p <* 0.001) and abstinence in the past 14 days (45.1% versus 26.4%; adjusted odds ratio 1.92 [95% CI 1.19, 3.10]; *p =* 0.008) being significantly higher in the CAP plus EUC arm than in the EUC alone arm. CAP participants also fared better on secondary outcomes including recovery (AUDIT score < 8 at 3 and 12 months: 27.4% versus 15.1%; aPR 1.90 [95% CI 1.21, 3.00]; *p =* 0.006) and percent of days abstinent (mean percent [SD] 71.0% [38.2] versus 55.0% [39.8]; adjusted mean difference 16.1 [95% CI 7.1, 25.0]; *p =* 0.001). The intervention effect for remission was higher at 12 months than at 3 months (aPR 1.50 [95% CI 1.09, 2.07]). There was no evidence of an intervention effect on Patient Health Questionnaire 9 score, suicidal behaviour, percentage of days of heavy drinking, Short Inventory of Problems score, WHO Disability Assessment Schedule 2.0 score, days unable to work, or perpetration of intimate partner violence. Economic analyses indicated that CAP plus EUC was dominant over EUC alone, with lower costs and better outcomes; uncertainty analysis showed a 99% chance of CAP being cost-effective per remission achieved from a health system perspective, using a willingness to pay threshold equivalent to 1 month’s wages for an unskilled manual worker in Goa. Readiness to change level at 3 months mediated the effect of CAP on mean standard ethanol consumption at 12 months (indirect effect −6.014 [95% CI −13.99, −0.046]). Serious adverse events were infrequent, and prevalence was similar by arm. The methodological limitations of this trial are the susceptibility of self-reported drinking to social desirability bias, the modest participation rates of eligible patients, and the examination of mediation effects of only 1 mediator and in only half of our sample.

**Conclusions:**

CAP’s superiority over EUC at the end of treatment was largely stable over time and was mediated by readiness to change. CAP provides better outcomes at lower costs from a societal perspective.

**Trial registration:**

ISRCTN registry ISRCTN76465238

## Abstract 2

In a randomized controlled trial, Vikram Patel and colleagues examine the 12 month effectiveness and cost-effectiveness of a brief psychological treatment for harmful drinking in men, delivered by lay counsellors in primary care.

## Author summary

**Why was this study done?:**

- Alcohol use disorders (AUDs), including harmful drinking, are one of the leading mental health contributors to the global burden of disease.
- Access to effective treatments is low globally, but especially so in low- and middle-income countries (LMICs) like India, where a recent national survey reported a treatment gap of 86% for AUDs.
- Counselling for Alcohol Problems (CAP) is a brief psychological treatment based on the principles of motivational interviewing and delivered by non-specialist providers; we have earlier reported the effectiveness of this intervention in increasing abstinence and promoting remission at the end of treatment.
- The goal of the present study was to evaluate the sustained effectiveness and the cost-effectiveness of CAP over 12 months.

**What did the researchers do and find?:**

- We implemented a randomised controlled trial in which 377 adult male primary healthcare attendees with harmful drinking were assigned to either the CAP treatment plus enhanced usual care (EUC) (*n =* 188) or EUC alone (*n =* 189); those assigned to CAP received treatment over 2 months.
- CAP participants maintained the gains they showed at the end of treatment through the 12-month period, with higher remission and abstinence rates than among individuals who received EUC alone.
- Cost analyses indicated that CAP was likely to be cost-effective, and could even save money if productivity costs were taken into account.

**What do these findings mean?:**

- CAP is associated with sustained effects on drinking outcomes over a 12-month period and represents good value for money.
- CAP is ideally suited for scaling up to reduce the treatment gap for harmful drinking.

## Introduction

Alcohol use disorders (AUDs) [1] contribute substantially to the disability and premature mortality attributable to mental and substance use disorders [2]. In low- and middle-income countries (LMICs), alcohol use is a leading risk factor for disease and injuries [3]. Harmful drinking is also associated with socioeconomic consequences for the drinker (e.g., loss of earnings), harm to others (e.g., domestic violence), and harm to society at large (e.g., loss of productive years of life to death and disability) [1]. Economic growth in India has made it a key target for trans-national producers of alcoholic beverages, resulting in increased alcohol availability, alcohol consumption, and alcohol-related problems [4,5]. Although the less severe forms of AUDs (hazardous or harmful drinking) affect a larger proportion of the population than the more severe AUD (dependent drinking), the policy response in India remains focused predominantly on the latter [4]. There is substantial evidence for the effectiveness of brief psychological treatments for AUDs [6], and, with larger effect sizes in studies that have excluded dependent drinkers [7], such interventions are recommended for scaling up in primary care [8]. However, the vast majority of people in LMICs, including India, lack access to such interventions; for example, the recent National Mental Health Survey of India reported that 86% of persons with AUDs had not received any treatment in the previous 12 months [9].

The PRogram for Effective Mental health Interventions in Under-resourced health systeMs (PREMIUM) used a systematic framework to develop and evaluate the Healthy Activity Programme (HAP) for depression and Counselling for Alcohol Problems (CAP) for harmful drinking, both potentially scalable psychological treatments that are culturally appropriate, affordable, and feasible for delivery by the same pool of non-specialist health workers (whom we refer to as lay counsellors) [10–13], as they would be delivered in actual clinical practice. We have previously reported the findings of the impact of the CAP treatment on the primary (drinking) and secondary (consequences of alcohol use and costs of illness) outcomes at the primary endpoint of 3 months [14]. At 3 months, there was an intervention effect on remission on the Alcohol Use Disorders Identification Test (AUDIT) (36.0% in the CAP plus enhanced usual care [EUC] arm versus 25.6% in the EUC alone arm; aPR 1.50 [95% CI 1.09, 2.07]), the proportion abstinent in the past 14 days (41.5% versus 18.0%; adjusted odds ratio [aOR] 3.00 [95% CI 1.76, 5.13]), and percent of days abstinent in past 14 days (mean 69.4% versus 54.4%; adjusted mean difference [AMD] 16.0%; *p <* 0.001), but no effect on other drinking and related outcomes. Having reported the favourable results of the effectiveness of CAP in reducing harmful drinking and increasing abstinence at the primary end-of-treatment endpoint of 3 months post-enrolment, the question now becomes whether these effects were sustained following the end of treatment in a disorder that is highly prone to relapse, especially given the delivery of CAP by non-specialised workers (brief treatments for AUDs in high-income countries are typically delivered by highly trained professionals). In addition, a meaningful sustained effect should be accompanied by evidence of the mediating factor targeted by the intervention accounting for its effects. In this paper we address 4 new questions: the effects of the intervention on drinking and other outcomes 12 months post-enrolment, the cost-effectiveness of the intervention over this period, for whom and under what circumstances (moderators) the intervention works, and the mediation of these outcomes by patient ‘readiness to change’ assessed at 3 months.

## Methods

The methods are described in detail in the protocol (S1 Text) [15] and the 3-month outcome paper [14], and a summary is presented below. The trial was conducted in alignment with the protocol (ISRCTN76465238) (S1 Text) [15], which was approved by the trial steering committee (TSC). Approval for the conduct of the trial was obtained from the institutional review boards of the London School of Hygiene & Tropical Medicine, Sangath (the implementing institution in India), and the Indian Council of Medical Research. Written (or witnessed, if the participant was illiterate) informed consent was mandatory for enrolment. This study is reported as per CONSORT guidelines (S1 CONSORT Checklist).

### Study design and participants

This was a parallel-arm, single-blind, individually randomised controlled trial conducted in 10 primary health centres (PHCs) in Goa, a state on the west coast of India. The Directorate of Health Services in Goa gave permission for PREMIUM to operate in 10 of the 14 PHCs in the north district of Goa. We started screening in 8 PHCs, but during the trial, 2 of these PHCs were replaced: 1 had low attendance and 1 had a large proportion of migrant labourers. So, while the trial was conducted in a total of 10 PHCs, at any given time point in the trial, screening was happening in only 8 of these facilities. Participants were consenting males aged 18–65 years who met the a priori eligibility criteria (residing within the PHC catchment area, planning to stay at the same address for at least 12 months, able to speak English or the local vernacular, and not having been screened for harmful drinking in the past 3 months) [14,15] and were harmful drinkers, defined as scoring 12–19 on AUDIT [16], a 10-item screening questionnaire developed by the World Health Organization for the detection of AUDs and validated in India [17]. Consenting participants were randomised in a 1:1 allocation scheme to either of 2 intervention arms (EUC or CAP plus EUC) after completion of the baseline assessments, using sequentially numbered opaque sealed envelopes [18]. Baseline assessments included data on socio-demographic factors and potential moderators of treatment outcome: illness severity (baseline AUDIT score), readiness to change, and expectations from treatment. Enrolment was conducted between 28 October 2013 and 29 July 2015, 3-month assessment was completed on 30 November 2015, and the final 12-month assessment was completed on 30 August 2016. Physicians providing EUC were masked to allocation status, as were the independent assessors who did the outcome assessments, and these people had no contact with the PHCs or other team members. All authors, apart from the data manager (BB), were masked until the trial results were unmasked.

### Outcomes

The following outcomes were examined at 12 months post-enrolment. The 2 primary outcomes were remission, defined as an AUDIT score < 8, and mean standard ethanol (in grams) consumed in the past 14 days immediately preceding the 12-month outcome evaluation. A range of secondary outcomes (S1 Table) included recovery (AUDIT score < 8 at both 3 and 12 months), percent of days abstinent in past 14 days, percent of days of heavy drinking in past 14 days, the Short Inventory of Problems (SIP) mean score, Patient Health Questionnaire 9 (PHQ-9) mean score, disability (WHO Disability Assessment Schedule 2.0 [WHODAS 2.0] score), total days unable to work in past 30 days, suicidal behaviour (suicidal thoughts in past 14 days and/or suicidal attempts in past 3 months), perpetration of intimate partner violence in past 3 months, and resource use and costs of illness estimated from the Client Service Receipt Inventory (CSRI) [19]. Percentage of days abstinent and percentage of days of heavy drinking generated from the Alcohol Timeline Followback were not pre-specified but were added prior to commencing analysis to bring the trial in line with recommendations of the US National Institute on Alcohol Abuse and Alcoholism. Similarly, our proposed mediator of patient-reported readiness to change at 3 months was added to the trial protocol midway through the trial, and thus data were available for only a subset of participants. Patient-reported readiness to change at 3 months was pre-selected as a potential mediator of the intervention for the mean standard ethanol consumption outcome, rather than the remission outcome as measured by the AUDIT score, for 2 reasons: the former is the most widely used outcome in alcohol trials, and it represents a continuous score, which is recommended over binary variables in mediation analyses to capture adequate variance [20]. Description of all the outcome tools and their contextual validity is provided in the published trial protocol (S1 Text) [15].

### Sample size estimations

Based on the assumptions that participants would be randomised within each of the clinics, with 1 counsellor per PHC at any one time, an intra-cluster correlation of 0.04, a loss to follow-up of 15% over 3 months, and a 1:1 allocation ratio, a trial size of 400 enrolled participants with harmful drinking had 90% power to detect the hypothesized effects (effect size of 0.45 for mean standard ethanol consumed; remission rate of 68% for CAP plus EUC versus 40% for EUC alone) for the primary outcomes, with a 5% type I error. No multiple testing adjustment was made for multiple primary outcomes.

### Interventions

EUC comprised consultation with the PHC physician enhanced by providing the AUDIT screening results to the patient and physician, and a contextualised version of the WHO Mental Health Gap Action Programme (mhGAP) guidelines [21] for harmful drinking to the physician, which included information on when and where to refer for psychiatric care.

CAP is a manualised psychological treatment (S2 Text) delivered in 3 phases over a maximum of 4 sessions (each lasting approximately 30–45 minutes) at weekly to fortnightly intervals. The psychosocial strategies used include detailed assessment followed by personalised feedback, cognitive and behavioural skills, and relapse prevention. The stance adopted by the counsellor is that of motivational interviewing [22]. A participant was classified as a ‘planned discharge’ if at least 1 of the following criteria were met: participant’s exit from treatment was decided in collaboration with the counsellor, treatment goals were achieved, or the maximum of 4 sessions were completed. The 11 counsellors were adults who had no prior professional training and/or qualification in the field of mental health, had completed at least high school education, were fluent in the vernacular languages used in the study settings, and were trained and supervised in delivering CAP through a rigorous process. Further details of the intervention [12] and of the selection, training, and supervision of the counsellors are provided elsewhere [23]. The full intervention can be accessed online (http://cap.nextgenu.org).

Process and fidelity assessments were based on treatment completion rates from the counsellors’ clinical records, CAP therapy quality scores from peer and expert supervisor ratings of audio-recordings of sessions during weekly group supervision, and therapy quality ratings of a random selection of 10% of all sessions by an expert involved in the development of CAP. The same counsellors also delivered HAP to adults who met criteria for moderate to severe depression. Counsellors maintained separate clinical registers for the 2 groups of patients and reviewed individual patient records before each session. In order to ensure that their treatment-specific counselling skills were maintained throughout the trial, weekly peer-led group supervision sessions were structured in ways that involved holding separate sessions for each of the 2 treatments. This arrangement allowed the expert supervisors for each of the 2 treatments to provide more focused feedback to the counsellors.

### Statistical analyses

Analyses were intention-to-treat, with multiple imputation (20 iterations) for missing outcome data, assuming data were missing at random, and assuming predictive mean matching for positively skewed outcomes. The following variables were used in the imputation model: age, marital status, and baseline AUDIT score. Zero-inflated negative binomial (ZINB) regression [24] was used to estimate the intervention effect for positively skewed over-dispersed outcomes with an excess of zeros. Continuous outcomes with normally distributed residuals were analysed using linear regression, and binary outcomes were analysed using binary logistic regression. All models were adjusted for baseline AUDIT score and for PHC as a fixed effect to allow for within-PHC clustering. For ZINB regression, the intervention effect was estimated for all participants in 1 model as an adjusted prevalence ratio (aPR) with a 95% CI for proportion with zero (i.e., no reported drinking), and with an adjusted count ratio among those with non-zero responses. For other continuous outcomes, the intervention effect was reported as the AMD and 95% CI; for binary outcomes, the intervention effect was reported as aPR estimated using the marginal standardisation technique, with 95% CIs for the prevalence ratios estimated using the delta method [25] following logistic regression. Moderation of treatment effect was assessed for a priori defined moderators. Sensitivity analyses for linear and logistic regression models included adjustment for counsellor as a random effect, and complete case analysis. In addition, repeated measures analysis was conducted, including analysis of change over time between the 3- and 12-month endpoints. The repeated measures analysis included a treatment-by-time interaction term to allow for a different intervention effect at 3 versus 12 months. The Monte Carlo method for assessing mediation (MCMAM) [26] was used for assessing the mediating effects of readiness to change assessed at 3 months on the 12-month primary outcome of mean standard ethanol consumption over the previous 14 days for the sub-sample of participants for whom the mediating variable data had been collected. In the current study, 95% CI was computed with 20,000 repetitions.

Economic evaluation was performed from the healthcare system perspective and from a broader societal perspective, which also took account of productivity impacts on patients and families. Costs, including the intervention costs for CAP, per additional remission, additional individual in recovery and quality-adjusted life year (QALY) gained were calculated. Information on the use of health services, including contacts with PHCs, hospital doctor contacts and inpatient stays (including detoxification), medication use, and diagnostic tests, was collected from service users using a tailored version of the CSRI at 3 and 12 months. Unit costs for doctor contacts and inpatient stays were inflated to 2015 prices using published unit costs previously used in an economic evaluation of a brief psychological intervention in Goa [27]. Detailed information on medications and lab tests used were extracted from medical records, including costs to the public purse. Mean health system costs were then extrapolated to cover the full 12 months. Detailed information was also recorded on the time taken to deliver each CAP session and whether it was delivered at a PHC, over the telephone, or at a patient’s home. Travel time and transportation costs (mainly petrol costs) were also recorded for home visits, including ‘no show’ home visits. Per minute unit costs for counsellors, taking account of their training, supervision, costs related to home delivery, and other overheads, were then attached to time to estimate the total costs of intervention delivery. The number of days completely out of normal role (i.e., days unable to work) over the previous 30 days was based on responses to the WHODAS 2.0. WHODAS 2.0 data on days of activity cutback over this period were also included, with the assumption that each day of cutback would have half the value of a complete day out of role. The value of time that patients reported attending health services was estimated; when patients reported being accompanied by someone, it was assumed that 1 family member also incurred the same level of productivity loss. We assumed that the mean of patient and family time costs at 3 months and 12 months would also apply to the rest of the year. Costs due to cutback and complete days out of role were adjusted to avoid double counting time that patients spent attending health services. All patient and family time was valued using different daily wage rates recommended in 2015 by the Indian Office of the Labour Commissioner. The rate used was dependent on whether the patient/family member was classified as an unskilled, skilled, or clerical/professional worker. We assumed the value of days out of role for those classified as unemployed was the same as that for unskilled workers. Further information on data collection methods is provided elsewhere [14,15].

Differences in mean costs were compared using standard parametric tests. QALYs were derived through transformation of WHODAS 2.0 12-item scores [27]. Five imputations were run to deal with missing values for QALYs and cost data. Statistical uncertainty was explored through bootstrapping and the generation of cost-effectiveness acceptability curves showing the likelihood that CAP would be cost-effective at different willingness to pay thresholds. All costs are presented in 2015 international dollars. Statistical analyses were conducted using Excel 2016 and SPSS 21 for the cost-effectiveness analyses, SAS and R-Studio for the mediation analyses, and STATA 13/14 for all other analyses.

The PREMIUM statistical analysis plan (version 2, 17 December 2015) was originally drafted to address both 3- and 12-month outcomes. However, following the analyses of the 3-month outcomes, modifications to the plan for the 12-month outcome analyses were proposed, principally by making it a stand-alone plan specifically for the 12-month outcomes. The key differences from the registered protocol included changing SIP to a secondary outcome to reduce multiplicity of the primary outcomes and adding 2 secondary outcomes (percentage of days abstinent and percentage of days of heavy drinking generated from the Alcohol Timeline Followback) to bring the trial in line with recommendations of the US National Institute on Alcohol Abuse and Alcoholism. The draft revised analysis plan was then circulated to the TSC (independent chairperson) and data safety monitoring committee (DSMC) (independent members) for review and discussion through teleconference. The final analysis was started (1 October 2016) only after the analysis plan was approved and locked by the TSC/DSMC members (4 September 2016) (S3 Text).

## Results

### Trial conduct

**Fig 1:**
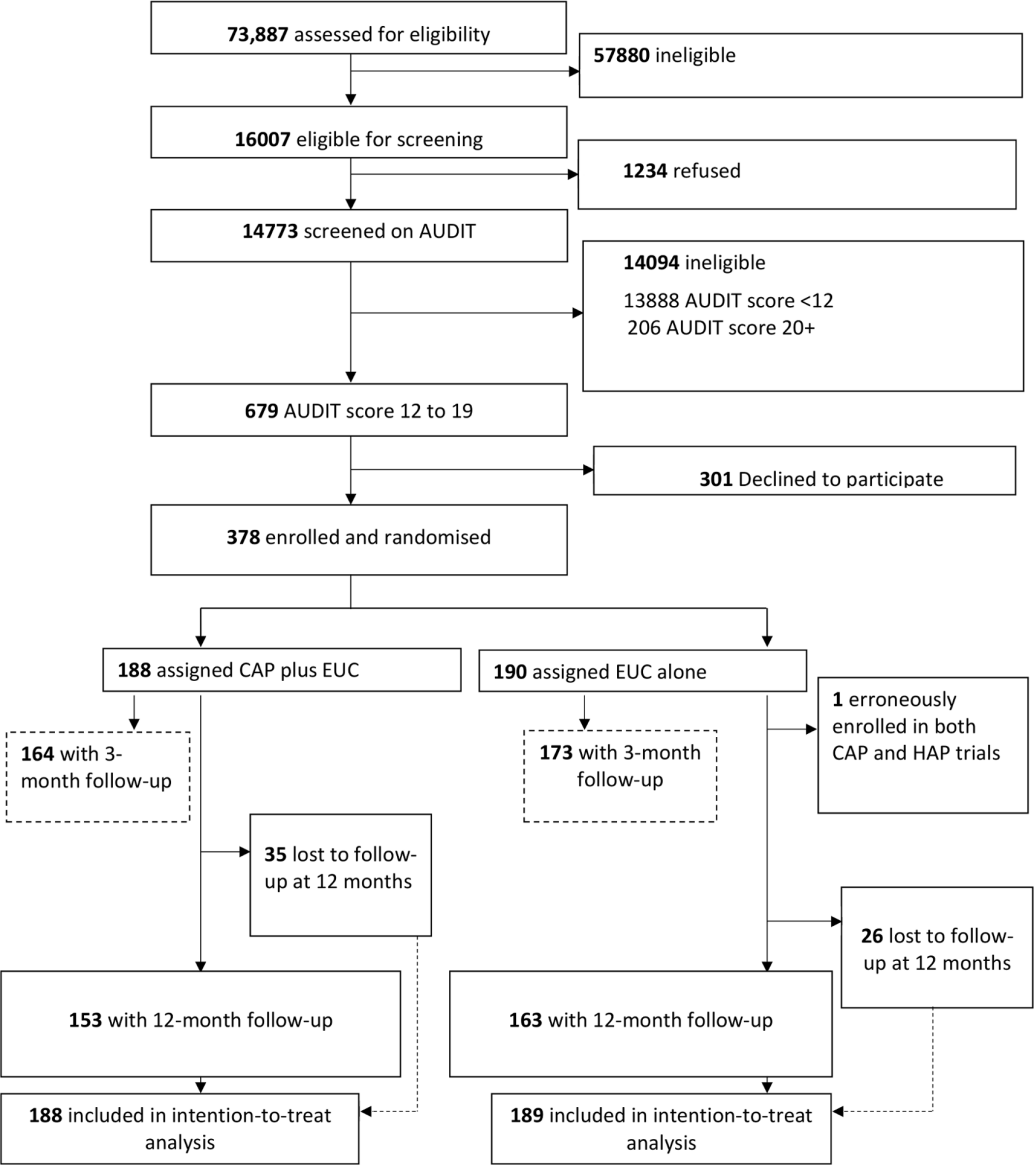
Counselling for alcohol problems trial flow chart. AUDIT, Alcohol Use Disorders Identification Test; CAP, Counselling for Alcohol Problems; EUC, enhanced usual care; HAP, Healthy Activity Programme.

A detailed description of the conduct of the trial is provided in the primary trial paper [14]. Between 28 October 2013 and 29 July 2015, 16,007 (21.7%) of the 73,887 adult male PHC attendees assessed met inclusion/exclusion criteria. Of these, 14,773 were screened for harmful drinking using AUDIT, of whom 679 (4.6%) were eligible (AUDIT score 12–19) for inclusion in the trial, and 378 (55.7%) consented to participate and were enrolled. A total of 190 participants were randomised to EUC alone, and 188 to CAP plus EUC. Of the former, 1 was subsequently excluded (erroneously enrolled in this trial as well as the one for depression), leaving a total of 189 participants in the EUC arm (Fig 1). The leading reasons for ineligibility for screening included age younger than 18 years or older than 65 years, already having been screened within the last 3 months, not planning to be resident in the study area for the duration of the study, and being resident outside of the study catchment areas. The common reasons for refusal to participate included ‘no interest’ in the trial (45.2%), ‘no time’ to participate (30.5%), and the patient’s belief that he did not have a problem (17.6%). There was no statistically significant difference in socio-demographic profile and baseline mean AUDIT score between those who consented and those who refused participation. Baseline characteristics were similar by arm. In all, 337 participants (89.4%) completed the primary outcomes at the 3-month post-enrolment endpoint, and 316 participants (83.8%) at the 12-month follow-up; rates were similar between arms. At the 12-month outcome evaluation, 315 participants completed PHQ-9, 312 completed the WHODAS 2.0, and 311 completed the CSRI. A total of 305 (81%) participants had primary outcome data for both follow-up time points. In all, only 30 (8%) participants did not have any follow-up data. Those lost to follow-up at 12 months were significantly younger (mean age [SD] 38.8 [11.8] versus 42.6 [11.2]; *p =* 0.02) (S2 Table), and this was consistent with the 3-month post-enrolment endpoint. Reasons for loss to follow-up were inability to track down the participant (29 [47.5%] of 61), refusal (20 [32.8%]), and death (12 [19.7%]). In all, 258 (81.1%) participants were seen within the 4-week window period for the 12-month assessment, and there were no statistically significant differences on baseline predictors of delayed 12-month outcome evaluation, i.e., outcome data collected outside the 4-week window period. In the CAP plus EUC arm, 131 (70%) of the participants had a planned discharge. The mean number of sessions for those who had a planned discharge was 2.8 (95% CI 2.7, 3.0), and for those who had an unplanned discharge it was 1.1 (95% CI 1.0, 1.3). Mean therapy quality scores on the basis of peer ratings (*n =* 183; mean 2.35 [95% CI 2.29, 2.41]), expert supervisor ratings (*n =* 183; mean 2.44 [95% CI 2.36, 2.51]), and independent ratings for a randomly selected 10% of sessions (*n =* 40; mean 2.64 [95% CI 2.42, 2.87]) were similar.

### Impact on clinical outcomes

Table 1 describes the intervention effect on all primary and secondary clinical outcomes at 12 months. The proportion with remission (AUDIT < 8) (54.3% versus 31.9%; aPR 1.71 [95% CI 1.32, 2.22]; *p <* 0.001) was significantly higher in the CAP plus EUC arm than in the EUC alone arm. Analysis of mean standard ethanol consumption showed a significantly higher proportion of participants reporting no alcohol consumption in the past 14 days in the CAP plus EUC arm than in the EUC alone arm (45.1% versus 26.4%; aOR 1.92 [95% CI 1.19, 3.10]; *p =* 0.008), but no difference in consumption among those who reported any drinking in this period. The proportion of participants in recovery (AUDIT < 8 at both 3 and 12 months) (27.4% versus 15.1%; aPR 1.90 [95% CI 1.21, 3.00]; *p =* 0.006) and mean percent of days abstinent (71.0% [SD 38.2] versus 55.0% [SD 39.8]; AMD 16.1 [95% CI 7.1, 25.0]; *p =* 0.001) were significantly higher in the CAP plus EUC arm than in the EUC alone arm. There was no evidence of an intervention effect on PHQ-9 scores, suicidal behaviour, and percentage of days of heavy drinking. The results were similar when adjusted for counsellor as a random effect, and when using complete case analyses (S3 Table).

**Table 1:** Effects of CAP plus EUC compared with EUC alone on primary and secondary clinical outcomes at 12 months.

Outcome	CAP + EUC^1^ (*n =* 153)^2^	EUC^1^ (*n =* 163)^2^	Intervention effect (95% CI)^3^	*p-*Value
**Primary outcomes**				
Remission (AUDIT < 8)*	83 (54.3%)	52 (31.9%)	aPR: 1.71 (1.32, 2.22)	<0.001
Mean standard ethanol consumed in the past 14 days^4^				
Non-drinkers**	69 (45.1%)	43 (26.4%)	aOR: 1.92 (1.19, 3.10)	0.008
Ethanol consumption (grams) among drinkers, mean (SD)	38.0 (40.0)	38.2 (34.8)	Count ratio: 0.99 (0.74, 1.33)	0.95
**Secondary outcomes**				
Recovery (AUDIT < 8 at 3 and 12 months)***	40 (27.4%)	24 (15.1%)	aPR: 1.90 (1.21, 3.0)	0.006
Percent of days abstinent, mean percent (SD)***	71.0 (38.2)	55.0 (39.8)	AMD: 16.1 (7.1, 25.0)	0.001
Percent of days of heavy drinking, mean percent (SD)***	12.5 (30.7)	11.0 (27.3)	AMD: 1.5 (−4.9, 7.9)	0.65
PHQ-9, mean (SD)	3.8 (5.0)	3.7 (5.1)	AMD: 0.2 (−1.0, 1.3)	0.77
Suicidal behaviour^#^	14 (8.6%)	17 (11.2%)	aPR: 1.31 (0.66, 2.61)	0.44

Data given as number (percent) unless otherwise indicated.

^1^Among those with observed data at 12 months.

^2^Number of participants for whom AUDIT and Alcohol Timeline Followback were available.

^3^Including imputed outcome data for those with missing data; adjusted for primary health centre as a fixed effect and baseline AUDIT score.

^4^Analysed with a zero-inflated negative binomial model that fits 2 parameters in 1 model, i.e., the proportion with response of zero (e.g., no drinking in 14 days or no days unable to work) and the mean count (e.g., ethanol consumption or days unable to work) among people with a non-zero (positive) response.

*Sensitivity analysis (complete case): 1.74 (95% CI 1.35–2.24).

**Sensitivity analysis (complete case): 2.03 (95% CI 1.25–3.32).

***Not previously specified in trial protocol but specified in published analysis plan.

^#^Suicidal thoughts over the past 2 weeks were assessed through the relevant PHQ-9 item while suicide attempts were assessed over the 3-month period leading up to the 12-month outcome follow-up assessment.

AMD, adjusted mean difference; aOR, adjusted odds ratio; aPR, adjusted prevalence ratio; AUDIT, Alcohol Use Disorders Identification Test; CAP, Counselling for Alcohol Problems; EUC, enhanced usual care; PHQ-9, Patient Health Questionnaire 9.

In repeated measures analyses for the primary outcomes, there was no significant interaction with time for mean standard ethanol consumption in the past 14 days (*p =* 0.09), amount of drinking among drinkers (*p =* 0.54), or remission (*p =* 0.17). We observed no evidence of significant effect modification by baseline AUDIT score, expectations of the usefulness of counselling, or readiness to change on the 2 primary outcomes (S4 Table).

**Fig 2:**
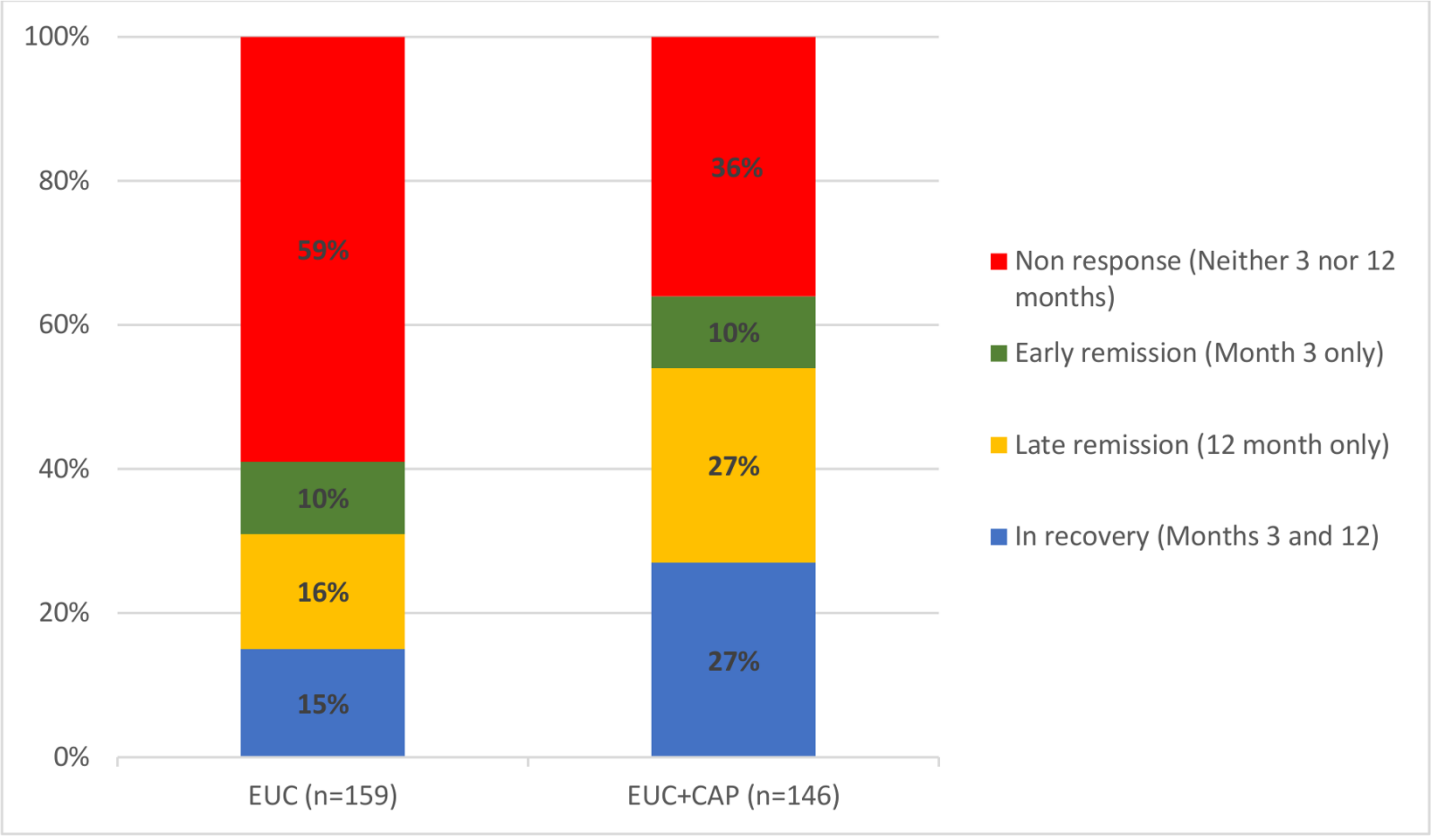
Clinical trajectories in participants with 3- and 12-month AUDIT data (n = 305) (complete case). Remission defined as AUDIT score < 8. AUDIT, Alcohol Use Disorders Identification Test; CAP, Counselling for Alcohol Problems; EUC, enhanced usual care.

**Fig 3:**
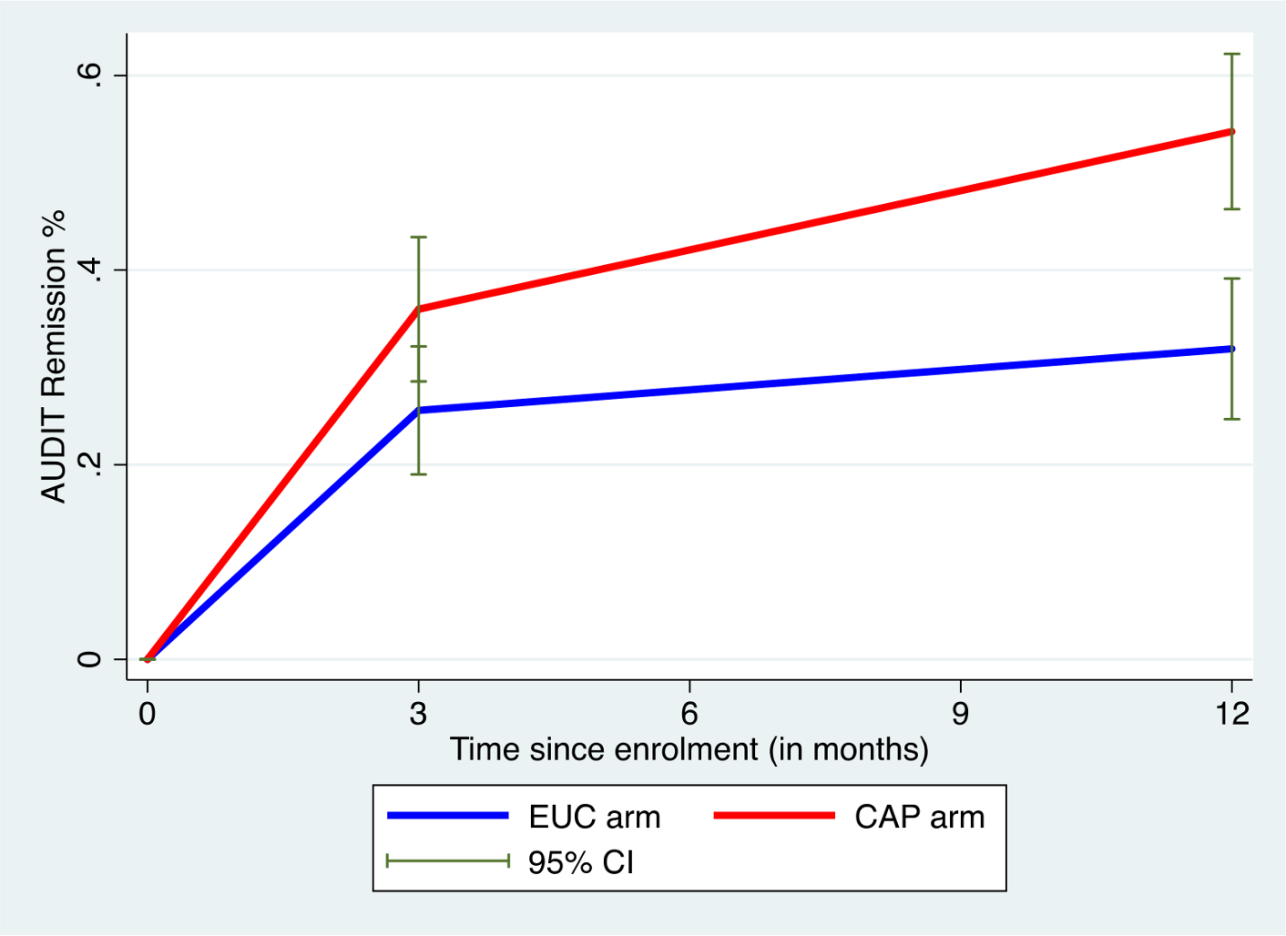
Remission in CAP plus EUC and EUC alone arms at 3 and 12 months. Remission defined as AUDIT score < 8. AUDIT, Alcohol Use Disorders Identification Test; CAP, Counselling for Alcohol Problems; EUC, enhanced usual care.

AUDIT scores at 3 and 12 months were available in 305 participants (80.9%). Compared to the EUC arm, a greater proportion in the intervention arm experienced a late remission (27.4% versus 15.7%) or were in recovery (27.4% versus 15.1%); in contrast, a greater proportion in the EUC arm remained harmful drinkers at both endpoints (59.1% versus 35.6%) (Fig 2). The remission rate showed an increase at 12 months (aPR 1.71 [95% CI 1.32, 2.22]), compared with 3 months (aPR 1.50 [95% CI 1.09, 2.07]) (Fig 3).

### Other outcomes and mediation analyses

There was no evidence of an intervention effect on SIP score, WHODAS 2.0 score, days unable to work, and perpetration of intimate partner violence. We observed no significant differences in the number of serious adverse events between the 2 arms (23 in CAP plus EUC versus 33 in EUC; *p =* 0.37) (S5 Table).

**Fig 4:**
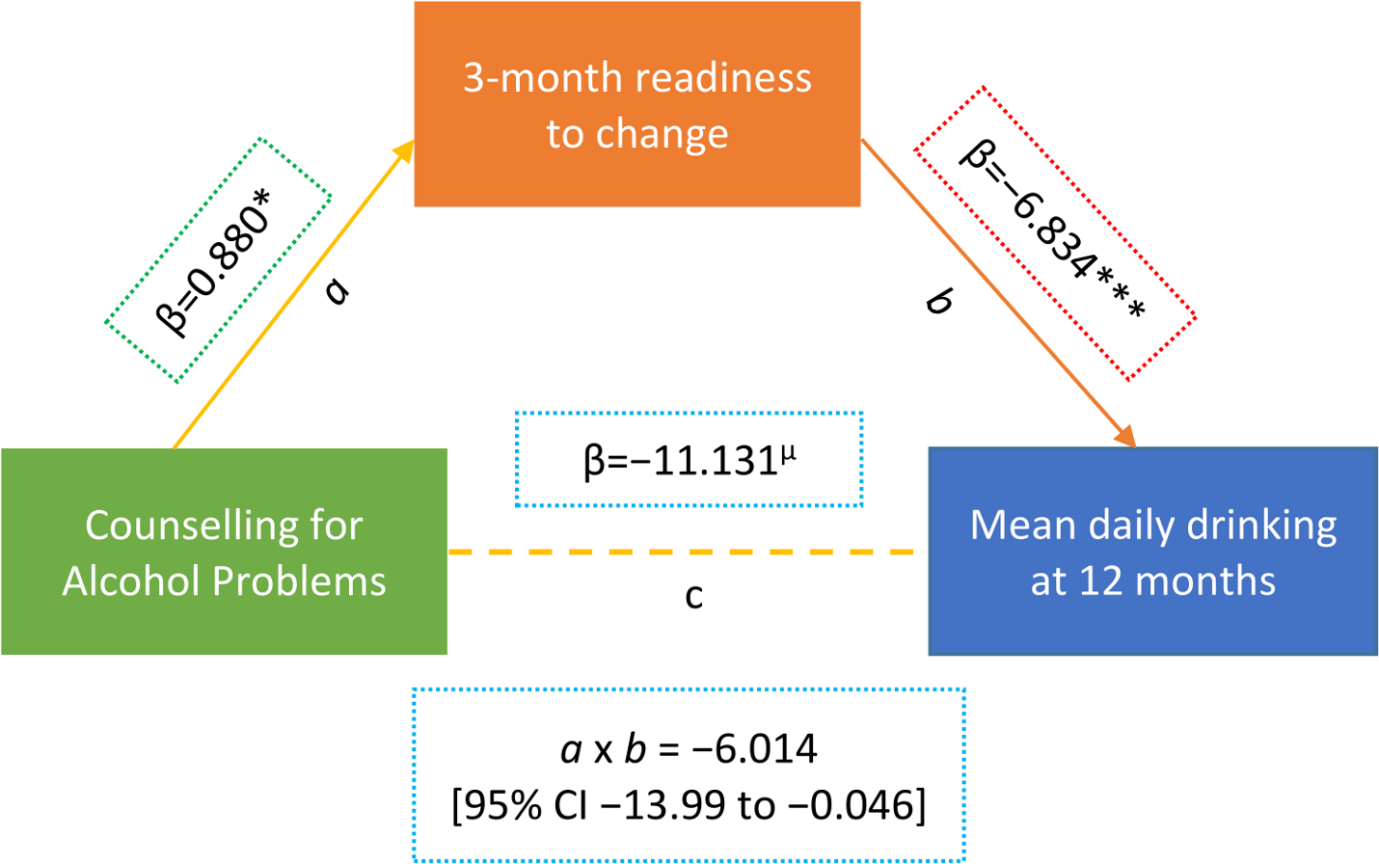
Mediating effect of readiness to change at 3 months on mean standard ethanol consumption at 12 months (n = 151). Beta estimates (β) are unstandardised. Multiple linear regression models controlled for baseline readiness to change, AUDIT scores, and PHQ-9 scores, where the intervention was delivered (primary health centre) and who delivered it (health counsellor), and patient education. µp ≤ 0.10. *p < 0.05. ***p < 0.001. Variables as follows: β, Beta coefficient; a, a-path (CAP→mediator); b, b-path (mediator→outcome); c, direct effect (CAP→outcome); a × b, indirect effect. AUDIT, Alcohol Use Disorders Identification Test; Counselling for Alcohol Problems; PHQ-9, Patient Health Questionnaire 9.

Data on readiness to change at 3 months was available for 151 participants (38.8% in CAP plus EUC arm versus 41.3% in EUC arm; *p =* 0.62). There was no significant difference in age (mean [SD] 42.8 [10.7] versus 41.4 [11.8] years; *p =* 0.24) or baseline AUDIT score (mean [SD] 15.0 [2.2] versus 14.8 [2.1]; *p =* 0.38) between those for whom the data were available and those for whom they were not available. Our mediation results found evidence of the CAP intervention having a predictive role in increased readiness to change at 3 months, and of increased readiness to change having a predictive role in reduced drinking outcomes at 12 months; thus, patient-reported readiness to change at 3 months mediated the effects of the CAP intervention on drinking outcomes at 12 months, whereby the indirect effect (*a* × *b*) was −6.014 (95% CI −13.99, −0.046) (Fig 4; S6 and S7 Tables). These relations remained even after controlling for variables that were related to participants’ readiness to change scores or drinking outcomes, including baseline readiness to change, AUDIT and depression scores, which PHC the intervention was delivered in or by which counsellor, and patient education level. Patient-reported readiness to change could account for 54% of the total effect of CAP plus EUC. None of the models demonstrated evidence of multicollinearity between independent variables (variance inflation factor < 4).

**Table 2:** Effect of CAP plus EUC compared with EUC alone on impact of harmful drinking, disability and intimate partner violence at 12 months.

Outcome	CAP + EUC^1^ (*n =* 153)^2^	EUC^1^ (*n =* 163)^2^	Intervention effect (95% CI)^3^	*p-*Value
**SIP, mean (SD)**	6.0 (8.9)	6.6 (8.7)	AMD: −0.4 (−2.4, 1.6)	0.70
**WHODAS 2.0 score, mean (SD)**	3.4 (5.7)	3.6 (6.1)	AMD: −0.3 (−1.7, 1.1)	0.65
**Days unable to work**^**4**^				
No days unable to work, *n* (%)	119 (78.3%)	116 (72.5%)	aOR: 1.27 (0.74, 2.18)	0.38
Days unable to work when ≤1 day reported, mean (SD)	12.1 (10.8)	11.3 (10.5)	Count ratio: 0.80 (0.47, 1.38)	0.43
**Perpetration of intimate partner violence**^**5**^**, *n* (%)**	7 (5.7%)	13 (9.7%)	aPR: 0.63 (0.27, 1.46)	0.28

^1^Among those with observed data at 12 months.

^2^Number of participants for whom AUDIT and Alcohol Timeline Followback were available.

^3^Including imputed outcome data for those with missing data.

^4^Analysed with a zero-inflated negative binomial model that fits 2 parameters in 1 model, i.e., the proportion with response of zero (e.g., no drinking in 14 days or no days unable to work) and the mean count (e.g., ethanol consumption or days unable to work) among people with a non-zero (positive) response.

^5^Among married participants only.

AMD, adjusted mean difference; aOR, adjusted odds ratio; aPR, adjusted prevalence ratio; AUDIT, Alcohol Use Disorders Identification Test; CAP, Counselling for Alcohol Problems; EUC, enhanced usual care; SIP, Short Inventory of Problems; WHODAS 2.0, WHO Disability Assessment Schedule 2.0.

### Costs and cost-effectiveness

**Fig 5:**
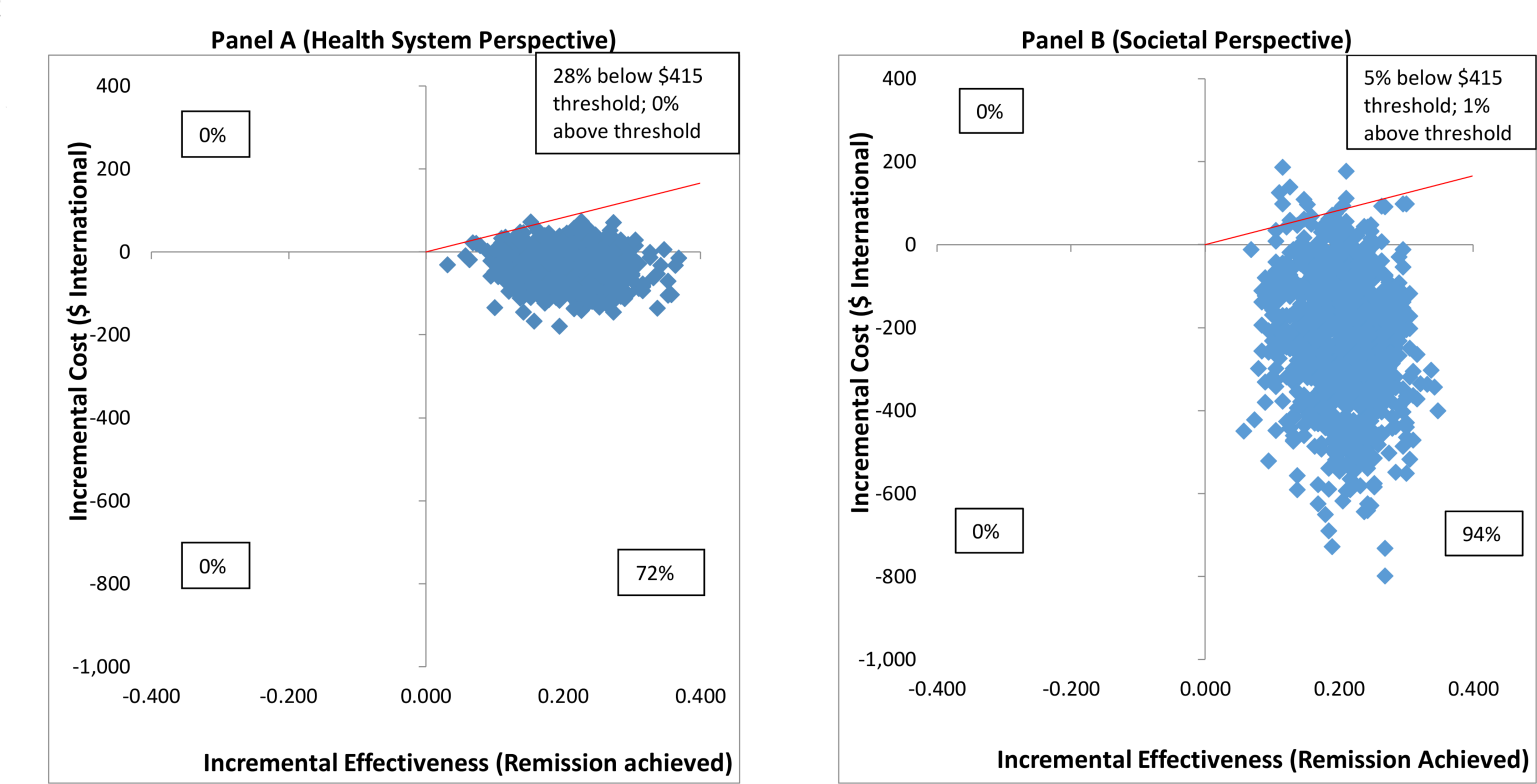
Cost effectiveness planes: CAP plus EUC compared to EUC per remission achieved. (A) Health system perspective; (B) societal perspective. CAP, Counselling for Alcohol Problems; EUC, enhanced usual care.

From the health system perspective, by 12 months, the mean estimated costs to the health system of providing the intervention were no longer significantly different from the costs of EUC, being slightly lower (though not statistically significantly so) than those for EUC, at $179.59 compared to $206.98 (mean difference −$27.40 [95% CI −$105.90, $51.10]; *p =* 0.49) (S8 Table). These costs had been significantly higher at 3 months due to the cost of providing CAP; by 12 months these costs were offset by reductions in the use of health services. From a wider societal perspective, which combines impacts on the health system with impacts on productivity costs, CAP plus EUC had a lower overall mean cost than EUC at 12 months of $484.31 per participant, but the difference between arms was not significant (mean difference −$223.12 [95% CI −$524.05, $77.82]; *p =* 0.15). There was no difference in mean QALYs gained per person at 12 months (mean difference 0.0006 [95% CI −0.091, 0.0102]). Table 3 provides an assessment of incremental cost-effectiveness. From a health system perspective, the CAP plus EUC arm dominates the EUC alone arm, with lower costs per additional remission (−$134 [95% CI −$598, $200]) or additional participant in recovery (−$269 [95% CI −$2,017, $608]) achieved at 12 months. It is difficult to draw conclusions on cost per QALY gained given the negligible difference in QALY outcomes between the 2 arms and thus the wide confidence intervals around incremental costs per QALY gained. To test the robustness of incremental cost-effectiveness ratio (ICER) results, cost-effectiveness analysis planes using 1,000 randomly resampled pairs of costs and remission outcomes or pairs of costs and recovery outcomes from both the health system and societal perspectives were used to generate further estimates of incremental cost per remission (Fig 5) or recovery gained (S1 Fig). Any observations in the southeast quadrant of these planes indicate that CAP plus EUC is cost-saving, having both lower costs and better outcomes than EUC, while observations in the northeast quadrant indicate that CAP plus EUC has increased costs and better outcomes than EUC. A threshold for what society is willing to pay for better outcomes must be determined. In this case we have assumed a very low threshold per additional remission achieved or additional participant in recovery of no more than the monthly wage for an unskilled manual worker in Goa ($415) [28]. This threshold is represented by a solid red line shown in the northeast quadrant. Fig 5A indicates that the ICER for CAP plus EUC compared to EUC has a 72% chance of being in the southeast quadrant—cost-saving per remission at 12 months from a health system perspective, i.e., having both lower costs and better remission outcomes than EUC—while there is a 28% chance of the ICER being in the northeast quadrant, where the intervention is still cost-effective even if costs are higher than for EUC, if an incremental cost per remission achieved threshold of $415 is applied. Overall, therefore, the case for investment is very strong, with a more than 99% likelihood that the intervention represents good value for money. In Fig 5B, when a societal perspective is used, where impacts on productivity losses are also considered, the likelihood of CAP plus EUC being cost-saving increases to 94% and the overall chances of its being cost-effective are over 99%.

**Table 3:** Cost-effectiveness analyses from health system and societal perspectives (costs in 2015 international dollars).

Category	Health system perspective		Societal perspective	
	Cost (95% CI)	Likelihood ICER is CS and CE	Cost (95% CI)	Likelihood ICER is CS and CE
Per remission at 12 months*	−$134 (−$598, $200)	CS: 72%; CE: 99%	−$1,094 (−$3,139, $268)	CS: 94%; CE: 99%
Per recovery at 12 months*	−$269 (−$2,017, $608)	CS: 72%; CE: 96%	−$2,192 (−$10,709, $796)	CS: 93%; CE: 96%
Per QALY gained at 12 months**	−$49,566 (−$104,045, $105,625)	CS: 74%; CE: 65%	−$403,622 (−$738,059, $945,923)	CS: 93%; CE: 87%

*Assumes willingness to pay threshold equivalent to 1 month’s wages for unskilled manual worker in Goa ($415).

**Assumes willingness to pay threshold equivalent to GDP per capita in Goa ($16,060).

CE, cost-effective; CS, cost-saving; ICER, incremental cost-effectiveness ratio; QALY, quality-adjusted life year.

## Discussion

We report on the sustained effects, cost-effectiveness, and role of readiness to change in mediating the effectiveness of CAP, a brief psychological treatment for harmful drinking delivered by lay counsellors in routine primary care settings, in a randomised controlled trial in India. Our findings demonstrate (1) that the effects of CAP on remission and abstinence outcomes were not just maintained at 12 months but enhanced in comparison to those observed at 3 months post-enrolment [14], indicating evidence of sustained recovery among harmful drinkers, (2) that the healthcare costs of provision of CAP are offset over 12 months, (3) that CAP produces gains in terms of productivity that have real implications for the individuals involved and for the larger society in which they are embedded, and (4) that patient-reported readiness to change at 3 months mediated the effect of CAP on mean standard ethanol consumption at 12 months.

To our knowledge, this is the first randomised controlled trial to evaluate the sustained effectiveness and cost-effectiveness of a brief treatment for harmful drinking delivered by lay counsellors in a low- or middle-income country. The existing evidence base on brief interventions for hazardous or harmful drinking is for delivery by health professionals (e.g., general practitioners, practice nurses) and relates to briefer forms of advice and counselling [29]. Thus, CAP adds to the existing evidence base on 3 main parameters: being targeted specifically at harmful drinkers, being delivered by lay counsellors, and involving a specific psychological treatment intervention. In addition, very few studies in the global literature have assessed what accounts for trial results; our mediation results highlight that patients who were already engaged in changing their behaviours at 3 months were more likely to have reduced drinking alcohol at 12 months. Importantly, this finding suggests a confirmation of the theoretical basis of the motivational interviewing stance of CAP. In addition to the sustained clinical effects, an important economic consideration in favour of CAP is that, over time, the additional costs of providing CAP were offset by reductions in subsequent utilisation of health services; thus, we also observed a high probability of the intervention being cost-saving from a health system perspective, indicating it represented good value for money for policymakers.

Although brief interventions have been shown to be effective in the short term, there is a decay in impact over time [7]. Our findings indicate that the effect of CAP on the outcome of remission was sustained between 3 and 12 months, potentially because of the effect of the treatment on readiness to change at 3 months. In addition to motivating the drinker to make a change in thought and action, CAP also provides the harmful drinker with tools to change behaviour and handle a variety of underlying problems. This skill transfer from the counsellor to the harmful drinker potentially empowers the latter to autonomously make changes without the need for continued reinforcement by the counsellor. This would be consistent with findings about cognitive behavioural interventions that demonstrate sustained impact beyond the intervention delivery period due to transfer of skills and people’s empowerment to use them [30,31]. Finally, the mediating role of ‘readiness to change’ on 12-month outcomes parallels previous brief intervention findings [32] and a recent treatment study that identified a large effect of 3-month stage of change on 12-month outcomes [33]. These results underscore the importance of making gains during treatment, particularly securing abstinence after 3 months, in relation to 12-month drinking outcomes.

Our findings (a significant effect on remission and abstinence status, yet not on reduction of the amount of alcohol consumed amongst those who did consume alcohol at 12 months) are consistent with the findings at the 3-month outcome evaluation and possibly reflect the prevailing cultural norms that stress the importance of abstinence in India [34]. At 3 months, we also found a greater effect of CAP on those who were not already trying to make a change in their drinking behaviour compared with those who had already started to make a change, which indicates that the treatment enhanced motivation to change. In this paper we now demonstrate that readiness to change at 3 months does mediate the effect of CAP on the amount of alcohol consumed at 12 months, bearing in mind the key role of abstinence in the findings of this study. Notwithstanding the notable benefits of CAP in terms of drinking outcomes, it is clear that CAP did not have an effect on harmful drinking per se (mean standard ethanol consumption) or on the impact of harmful drinking on other domains such as disability and perpetration of domestic violence. There could be several reasons for the absence of these findings. It is possible that it takes longer than 12 months for change in heavy drinking patterns to translate into reduced levels of alcohol problems and into benefits in other related spheres of life, and hence we were not able to detect any differences between the 2 arms. Finally, due to the fluctuating clinical course of AUDs, the symptoms of harmful drinking vary significantly depending on the functional state of the individual, e.g., a harmful drinker with intermittent drinking bouts may show little impact of drinking on other domains of life between bouts, which makes it difficult to consistently measure these outcomes.

A key methodological limitation of this trial is reliance on self-reported drinking as the primary outcome. If drinking is under-reported in both randomised arms, this biases effect estimates towards the null. If social desirability bias disproportionately affects the intervention arm, this could lead to exaggeration of treatment effectiveness [35,36]. However, neither biological indicators nor collateral reports are regarded as sufficiently accurate for use in alcohol treatment trials [37]. Another limitation is the relatively high rate of refusal to participate in the trial, but this is very similar to other primary care trials that rely on opportunistic screening to identify participants with AUDs [38]. Finally, we did not adjust for multiple testing although we conducted analyses for 2 primary outcomes. There are also methodological strengths, such as minimal assessments at baseline to avoid assessment reactivity [39], as discussed in the 3-month outcome paper [14]. While the inclusion here of a mediation analysis is a key strength in a pragmatic trial for a psychological treatment for an AUD, we were able to examine mediation in only half of our sample because of the post hoc decision to measure this particular variable in the trial. However, this sub-sample was comparable to the whole sample enrolled in the trial. In addition, we did not measure other potential variables such as self-efficacy or behavioural skills that may mediate the effectiveness of psychological treatments. Nevertheless, the identification of readiness to change as a mediator suggests the importance of assessing this variable as a treatment outcome in interim analyses of longer term outcomes. A key strength of this trial was the use of minimal exclusion criteria to determine eligibility for participation.

Our findings have important clinical and policy implications. Despite evidence supporting the effectiveness of brief psychological interventions for AUDs and their selection as a best practice intervention for inclusion in a universal package of interventions for mental and substance use disorders, primary care in the global context has been slow to address the needs of problem drinkers [7,40]. This lack of implementation is due to multiple knowledge, attitudinal, and logistical barriers to implementation [41], including the fact that the majority of research has been conducted in high-income countries, in specialist alcohol treatment settings, and with treatment provided by specialised healthcare providers, all of which greatly limit generalisability to primary care and to LMICs because of varying drinking patterns, acceptability of psychological treatments or acknowledgment of a drinking problem, and lack of specialist health resources. Thus, CAP with its contextual sensitivity to primary care, treatment-naïve populations and suitability for delivery by lay counsellors in primary care, can potentially help to overcome these barriers globally. Furthermore, the importance of our findings cannot be overemphasised for a disorder with a relapsing and remitting course, in which sustained clinical effects are good value for the money. Finally, the scalability of CAP is enhanced by the fact that the lay counsellors had no prior professional mental health training (as would be the case in most real-world settings) and that they were concurrently delivering a different psychological treatment for depression (as would be the case in the real world) [10]. Future research on CAP may include the assessment of potential treatment-, therapist-, and patient-relevant variables on clinical outcomes within the CAP intervention arm, follow-ups of the trial cohorts to assess longer term enduring effects, and the evaluation of methods for scaling up the intervention when delivered by routine healthcare personnel.

## Supporting information

S1 CONSORT Checklist (DOC)

S1 Fig Cost-effectiveness planes: CAP plus EUC compared to EUC alone per recovery achieved. (DOCX)

S2 Fig Cost-effectiveness acceptability curve: Willingness to pay per remission achieved via counselling for alcohol problems from a health system perspective. (DOCX)

S1 Table Secondary outcomes at 12 months. (DOCX)

S2 Table Baseline characteristics of completers of outcome evaluation and those lost to follow-up. ^1^Includes those who completed the 3- and 12-month evaluations (*n =* 305) and those who completed only the 12-month evaluation (*n =* 11). ^2^Includes those who completed only the 3-month evaluation (*n =* 31) and those who dropped out before the 3-month evaluation (*n =* 30).
(DOCX)

S3 Table Intervention effect on outcomes at 12 months (complete case analysis and random effects). ^1^Among those with observed data at 12 months. ^2^Number of participants for whom AUDIT and Alcohol Timeline Followback were available. ^3^Including imputed outcome data for those with missing data. ^4^Analysed with a zero-inflated negative binomial model that fits 2 parameters in 1 model, i.e., the proportion with response of zero (e.g., no drinking in 14 days or no days unable to work) and the mean count (e.g., ethanol consumption or days unable to work) among people with a non-zero (positive) response.
(DOCX)

S4 Table Interaction effect of readiness to change, expectations of treatment, and drinking severity (AUDIT score) at baseline on the effect of CAP on primary outcomes. (DOCX)

S5 Table Description of serious adverse events over 12 months by arm. (DOCX)

S6 Table Means and 95% CIs for key variables used in mediation analyses. (DOCX)

S7 Table Mediation results examining patient-reported readiness to change at 3 months on mean standard ethanol consumption at 12 months (*n =* 151). Beta estimates (β) are unstandardised. Multiple linear regression models controlled for baseline AUDIT score, baseline PHQ-9 score, where the intervention was delivered (primary health centre) and who delivered it (health counsellor), and patient education. ^µ^*p* ≤ 0.10. *p < 0.05. ***p < 0.001.
(DOCX)

S8 Table Mean costs (2015 international dollars) and QALYs gained per person over 12 months. (DOCX)

S1 Text Study protocol. The effectiveness and cost-effectiveness of lay-counsellor-delivered psychological treatments for harmful and dependent drinking and moderate to severe depression in primary care in India: PREMIUM study protocol for randomised controlled trials.
(PDF)

S2 Text Counselling for Alcohol Problems (CAP) manual. (PDF)

S3 Text Statistical analysis plan for the PREMIUM randomised controlled trials of the effectiveness and cost-effectiveness of lay-counsellor-delivered psychological treatments for harmful and dependent drinking and moderate to severe depression in primary care in India. (DOC)

## Abbreviations

AMD: adjusted mean difference
aOR: adjusted odds ratio
aPR: adjusted prevalence ratio
AUD: alcohol use disorder
AUDIT: Alcohol Use Disorders Identification Test
CAP: Counselling for Alcohol Problems
CSRI: Client Service Receipt Inventory
EUC: enhanced usual care
HAP: Healthy Activity Programme
ICER: incremental cost-effectiveness ratio
LMICs: low- and middle-income countries
PHC: primary health centre
PHQ-9: Patient Health Questionnaire 9
PREMIUM: PRogram for Effective Mental health Interventions in Under-resourced health systeMs
QALY: quality-adjusted life year
SIP: Short Inventory of Problems
TSC: trial steering committee
WHODAS 2.0: WHO Disability Assessment Schedule 2.0
