# Supplementary material for: Sustained effectiveness and cost-effectiveness of Counselling for Alcohol Problems, a brief psychological treatment for harmful drinking in men, delivered by lay counsellors in primary care: 12-month follow-up of a randomised controlled trial
Source: PLoS Med. 2017 Sep 12;14(9):e1002386. doi: 10.1371/journal.pmed.1002386 (PMC5595289; doi:10.1371/journal.pmed.1002386)
Supplement: S2 Fig — (DOCX) [file pmed.1002386.s003.docx]

**S2 Figure: Cost-effectiveness acceptability curve: willingness to pay per remission achieved from Counselling for Alcohol Problems from a health system perspective**
