# Supplementary material for: Sustained effectiveness and cost-effectiveness of Counselling for Alcohol Problems, a brief psychological treatment for harmful drinking in men, delivered by lay counsellors in primary care: 12-month follow-up of a randomised controlled trial
Source: PLoS Med. 2017 Sep 12;14(9):e1002386. doi: 10.1371/journal.pmed.1002386 (PMC5595289; doi:10.1371/journal.pmed.1002386)
Supplement: S1 Table — (DOCX) [file pmed.1002386.s004.docx]

**S1 Table: Secondary outcomes at 12 months**

| **Secondary outcome** | **Measure of outcome** |
| --- | --- |
| Recovery from harmful drinking | AUDIT score <8 at both 3 and 12 months |
| Percent of days abstinent | Percentage of days patients remained abstinent from alcohol during the 14 days preceding the outcome evaluation. |
| Percent days of heavy drinking | Percentage of days patients consumed >70g alcohol during the 14 days preceding the outcome evaluation. |
| Impact of harmful drinking | Mean score on the Short Inventory of Problems (SIP) |
| Disability | Mean disability score on the WHO Disability Assessment Schedule version 2 (WHO-DAS II) |
| Depression | Mean PHQ-9 score |
| Total days unable to work | Mean total days unable to work in the previous month on the WHO-DAS II. |
| Suicidal behaviour | Proportion reporting suicide thoughts in the last two weeks on the PHQ-9; proportion reporting any suicide attempts in the last 3 months |
| Intimate partner violence | Proportion reporting perpetration of intimate partner violence over the past 3 months. |
| Resource impacts for the health system | Estimates of cost-effectiveness/cost-saving using detailed electronic records on HAP delivery, as well as other use of primary and secondary care services collected from patients using the Client Service Receipt Inventory. |
