# Supplementary material for: Sustained effectiveness and cost-effectiveness of Counselling for Alcohol Problems, a brief psychological treatment for harmful drinking in men, delivered by lay counsellors in primary care: 12-month follow-up of a randomised controlled trial
Source: PLoS Med. 2017 Sep 12;14(9):e1002386. doi: 10.1371/journal.pmed.1002386 (PMC5595289; doi:10.1371/journal.pmed.1002386)
Supplement: S2 Table — Baseline characteristics of completers of outcome evaluation and those lost to follow-up. 1Includes those who completed the 3- and 12-month evaluations (n = 305) and those who completed only the 12-month evaluation (n = 11). 2Includes those who completed only the 3-month evaluation (n = 31) and those who dropped out before the 3-month evaluation (n = 30). (DOCX) [file pmed.1002386.s005.docx]

**S2 Table: Baseline characteristics of completers of outcome evaluation and those lost to follow-up (LTFU)**

|  | **Completed 12 month outcome evaluation^1^**  **n=316 (83.8%)** | **LTFU^2^**  **n=61 (16.2%)** | **p** |
| --- | --- | --- | --- |
| Age (years) (mean [SD]) | 42.6 (11.2) | 38.8 (11.8) | 0.02 |
| Marital status (n [%]) |  |  | 0.08 |
| Married | 258 (81.7) | 43 (70.5) |  |
| Never married | 54 (17.1) | 16 (26.2) |  |
| Widowed, Divorced or separated | 4 (1.3) | 2 (3.3) |  |
| Education status (n [%]) |  |  | 0.32 |
| None | 59 (18.7) | 11 (18.0) |  |
| Primary | 170 (53.8) | 27 (44.3) |  |
| Secondary | 70 (22.2) | 17 (27.9) |  |
| Higher Secondary and above | 17 (5.4) | 6 (9.8) |  |
| Occupation (n [%]) |  |  | 0.14 |
| Unemployed | 49 (15.5) | 4 (6.6) |  |
| Unskilled manual | 220 (69.6) | 46 (75.4) |  |
| Skilled manual | 20 (6.3) | 5 (8.2) |  |
| Clerical or professional | 27 (8.5) | 6 (9.8) |  |
| AUDIT score |  |  |  |
| Median [IQR] | 15 (13-17) | 14 (13-16) | 0.07 |
| Mean [SD] | 14.96 (2.14) | 14.41 (1.94) | 0.06 |
| AUDIT categories (n[%]) |  |  | 0.06 |
| 12-15 | 192 (60.8) | 45 (73.8) |  |
| 16-19 | 124 (39.2) | 16 (26.2) |  |
| PHQ score |  |  |  |
| Median [IQR] | 4 (1-9) | 4 (1.5-7.5) | 0.96 |
| Mean [SD] | 5.31 (4.93) | 5.0 (4.13) | 0.65 |
| Readiness to make changes in drinking (n [%]) |  |  | 0.71 |
| No | 52 (16.5) | 11 (18.0) |  |
| Yes | 264 (83.5) | 50 (82.0) |  |
| Patient’s expectation of usefulness of counselling (n [%]) |  |  | 1.0 |
| Not useful or somewhat useful | 66 (20.9) | 12 (19.7) |  |
| Moderately or very useful | 250 (79.1) | 49 (80.3) |  |

^1^Includes those who completed 3 & 12 month evaluations (n=305), and those who completed only 12 month evaluation (n=11) ^2^Includes those who completed only 3 month evaluation (n=31) and those who dropped out before 3 month evaluation (n=30)
