# Supplementary material for: Sustained effectiveness and cost-effectiveness of Counselling for Alcohol Problems, a brief psychological treatment for harmful drinking in men, delivered by lay counsellors in primary care: 12-month follow-up of a randomised controlled trial
Source: PLoS Med. 2017 Sep 12;14(9):e1002386. doi: 10.1371/journal.pmed.1002386 (PMC5595289; doi:10.1371/journal.pmed.1002386)
Supplement: S3 Table — 1Among those with observed data at 12 months. 2Number of participants for whom AUDIT and Alcohol Timeline Followback were available. 3Including imputed outcome data for those with missing data. 4Analysed with a zero-inflated negative binomial model that fits 2 parameters in 1 model, i.e., the proportion with response of zero (e.g., no drinking in 14 days or no days unable to work) and the mean count (e.g., ethanol consumption or days unable to work) among people with a non-zero (positive) response. (DOCX) [file pmed.1002386.s006.docx]

**S3 Table: Intervention effect on outcomes at 12 months (complete case analysis and random effects)**

| **Outcome** | **EUC+CAP^1^**  **(n=153)^2^** | **EUC^1^**  **(n=163)^2^** | **Intervention effect (95% CI)^3^** | **p** |
| --- | --- | --- | --- | --- |
| **PRIMARY OUTCOMES** |  |  |  |  |
| **Complete case** |  |  |  |  |
| Remission (AUDIT<8) (n [%]) | 83 (54.3) | 52 (31.9) | aPR 1.74 (1.35-2.24)  aOR 2.99 (1.82-4.92) | <0.001  <0.001 |
| Daily standard ethanol consumed in the past 14 days^4^ |  |  |  |  |
| - Non-drinkers (n [%]) | 69 (45.1) | 43 (26.4) | aOR 2.03 (1.25-3.32) | 0.004 |
| - Ethanol consumption among drinkers (g) (mean (SD)) | 38.0 (40.0) | 38.2 (34.8) | Count ratio 0.98 (0.74-1.31) | 0.91 |
| **Random effects** |  |  |  |  |
| Remission (AUDIT<8) (n [%]) | 83 (54.3) | 52 (31.9) | aOR 2.99 (1.82-4.92) | <0.001 |
| **SECONDARY OUTCOMES** |  |  |  |  |
| **Complete case** |  |  |  |  |
| Recovery (AUDIT<8 at 3 and 12 months (n [%]) | 40 (27.4) | 24 (15.1) | aPR 1.91 (1.23-3.0)  aOR 2.39 (1.32-4.32) | 0.003  0.004 |
| Percent of days abstinent (mean% [SD]) | 71.0 (38.2) | 55. 0 (39.8) | AMD 16.3 (7.8-24.9) | <0.001 |
| Percent days of heavy drinking (mean% [SD]) | 12.5 (30.7) | 11.0 (27.3) | AMD 1.5 (-4.7-7.8) | 0.63 |
| Short inventory of problems (SIP) (mean [SD]) | 6.0 (8.9) | 6.6 (8.7) | AMD -0.5 (-2.4-1.4) | 0.61 |
| Patient Health Questionnaire-9 (PHQ-9) (mean [SD]) | 3.8 (5.0) | 3.7 (5.1) | AMD 0.2 (-0.9-1.2) | 0.78 |
| WHO-DAS score (mean (SD)) | 3.4 (5.7) | 3.6 (6.1) | AMD -0.4 (-1.7-0.9) | 0.58 |
| Days unable to work^4^ |  |  |  |  |
| - None (n [%]) | 119 (78.3) | 116 (72.5) | aOR 1.34 (0.77-2.32) | 0.30 |
| - Days unable to work when >1 day reported (mean (SE)) | 12.1 (10.8) | 11.3 (10.5) | Count Ratio 0.72 (0.43-1.20) | 0.20 |
| Suicidal behaviour (n [%]) | 14 (8.6) | 17 (11.2) | aPR 1.23 (0.06-11.20)  aOR 1.30 (0.59-2.88) | 0.86  0.52 |
| **Random effects** |  |  |  |  |
| Recovery (AUDIT<8 at 3 and 12 months (n [%]) | 40 (27.4) | 24 (15.1) | aOR 2.39 (1.32-4.32) | 0.004 |
| Percent of days abstinent (mean% [SD]) | 71.0 (38.2) | 55. 0 (39.8) | AMD 16.3 (8.0-24.7) | <0.001 |
| Percent days of heavy drinking (mean% [SD]) | 12.5 (30.7) | 11.0 (27.3) | AMD 1.5 (-4.6-7.7) | 0.62 |
| Short inventory of problems (SIP) (mean (SD)) | 6.0 (8.9) | 6.6 (8.7) | AMD -0.5 (-2.4-1.4) | 0.60 |
| Patient Health Questionnaire-9 (PHQ-9) (mean [SD]) | 3.8 (5.0) | 3.7 (5.1) | AMD 0.2 (-0.9-1.2) | 0.77 |
| WHO-DAS score (mean (SD)) | 3.4 (5.7) | 3.6 (6.1) | AMD -0.4 (-1.6-0.9) | 0.57 |
| Suicidal behaviour (n [%]) | 14 (8.6) | 17 (11.2) | aOR 1.30 (0.59-2.88) | 0.52 |

^1^Among those with observed data at 12 months ^2^Number of participants for whom AUDIT and TLFB is available ^3^ Including imputed outcome data for those with missing data ^4^Analysed with a zero-inflated negative binomial model which fits two parameters in one model i.e. the proportion with response of zero (e.g. no drinking in 14 days; or no days unable to work), and the mean count (e.g. ethanol consumption or days unable to work) among people with a non-zero (positive) response
