# Supplementary material for: Sustained effectiveness and cost-effectiveness of Counselling for Alcohol Problems, a brief psychological treatment for harmful drinking in men, delivered by lay counsellors in primary care: 12-month follow-up of a randomised controlled trial
Source: PLoS Med. 2017 Sep 12;14(9):e1002386. doi: 10.1371/journal.pmed.1002386 (PMC5595289; doi:10.1371/journal.pmed.1002386)
Supplement: S4 Table — (DOCX) [file pmed.1002386.s007.docx]

**S4 Table:** **Interaction effect of readiness to change, expectations of treatment, and drinking severity at baseline on the effect of CAP on primary outcomes**

| **Effect Modifier** | **EUC**  **N=163** | **EUC+CAP**  **N=153** | **Intervention effect (95% CI), p** | **p** |
| --- | --- | --- | --- | --- |
| **DAILY STANDARD ETHANOL CONSUMED IN PAST 14 DAYS** | | | | |
| **Readiness to change at baseline** |  |  | p for effect modification: Non-drinkers 0.72; Ethanol consumption among drinkers 0.63 | |
| No |  |  |  |  |
| - Non-drinkers (n [%]) | 6 (22.2) | 8 (32.0) | aPR 1.55 (0.48-5.00) | 0.46 |
| - Ethanol consumption among drinkers (g) (mean (SD)) | 34.4 (27.2) | 27.4 (33.4) | Count ratio 1.00 (0.53-1.87) | 0.99 |
| Yes |  |  |  |  |
| - Non-drinkers (n [%]) | 37 (27.2) | 61 (47.7) | aPR 1.95 (1.19-3.22) | 0.008 |
| - Ethanol consumption among drinkers (g) (mean (SD)) | 39.0 (36.2) | 40.7 (41.3) | Count ratio 1.03 (0.76-1.41) | 0.84 |
| **Patient expectations** |  |  | p-effect modification: Non-drinkers 0.33; Ethanol consumption among drinkers 0.86 | |
| No |  |  |  |  |
| - Non-drinkers (n [%]) | 9 (25.7) | 10 (32.3) | aPR 1.06 (0.36-1.73) | 0.92 |
| - Ethanol consumption among drinkers (g) (mean (SD)) | 47.0 (35.6) | 41.0 (43.4) | Count ratio 1.05 (0.62-1.77) | 0.85 |
| Yes |  |  |  |  |
| - Non-drinkers (n [%]) | 34 (26.6) | 59 (48.7) | aPR 2.10 (1.26-3.50) | 0.004 |
| - Ethanol consumption among drinkers (g) (mean (SD)) | 35.8 (34.3) | 37.0 (39.1) | Count ratio 0.99 (0.72-1.36) | 0.94 |
| **Baseline AUDIT score** |  |  | p-effect modification: Non-drinkers 0.42; Ethanol consumption among drinkers 0.47 | |
| 12-15 |  |  |  |  |
| - Non-drinkers (n [%]) | 26 (27.1) | 47 (49.0) | aPR 2.14 (1.21-3.78) | 0.009 |
| - Ethanol consumption among drinkers (g) (mean (SD)) | 31.9 (29.7) | 28.4 (32.3) | Count ratio 0.94 (0.67-1.33) | 0.74 |
| 16-19 |  |  |  |  |
| - Non-drinkers (n [%]) | 17 (25.4) | 22 (38.6) | aPR 1.46 (0.70-3.03) | 0.31 |
| - Ethanol consumption among drinkers (g) (mean (SD)) | 47.1 (39.4) | 51.5 (45.9) | Count ratio 1.11 (0.72-1.70) | 0.64 |
| **REMISSION (AUDIT<8)** | | | | |
| **Readiness to change at baseline** |  |  | p-effect modification=0.55 | |
| No | 10 (37.0) | 17 (68.0) | aOR 4.04 (1.23-13.26) | 0.02 |
| Yes | 42 (30.9) | 66 (51.6) | aOR 2.75 (1.62-4.66) | <0.001 |
| **Patient expectations** |  |  | p-effect modification=0.26 | |
| No | 10 (28.6) | 12 (38.7) | aOR 1.61 (0.53-4.87) | 0.40 |
| Yes | 42 (32.8) | 71 (58.2) | aOR 3.32 (1.92-5.70) | <0.001 |
| **Baseline AUDIT score** |  |  | p-effect modification=0.08 | |
| 12-15 | 34 (35.4) | 63 (65.6) | aOR 3.95 (2.06-7.59) | <0.001 |
| 16-19 | 18 (26.9) | 20 (35.1) | aOR 1.55 (0.69-3.49) | 0.29 |
