# Supplementary material for: Sustained effectiveness and cost-effectiveness of Counselling for Alcohol Problems, a brief psychological treatment for harmful drinking in men, delivered by lay counsellors in primary care: 12-month follow-up of a randomised controlled trial
Source: PLoS Med. 2017 Sep 12;14(9):e1002386. doi: 10.1371/journal.pmed.1002386 (PMC5595289; doi:10.1371/journal.pmed.1002386)
Supplement: S5 Table — (DOCX) [file pmed.1002386.s008.docx]

**S5 Table: Description of serious adverse events over 12 months by arm**

| **Type of SAE** | **EUC+CAP**  **Number of SAEs (No. of participants)** | **EUC**  **Number of SAEs (No. of participants)** | **p-value** |
| --- | --- | --- | --- |
| Total SAEs | 23 (22) | 33 (32) | 0.37 |
| Death | 3 (3) | 9 (9) | 0.14 |
| Suicide attempt | 1 (1) | 4 (4) | 0.37 |
| Unplanned hospitalisation | 19 (18) | 20 (19) | 1.0 |
