# Supplementary material for: Sustained effectiveness and cost-effectiveness of Counselling for Alcohol Problems, a brief psychological treatment for harmful drinking in men, delivered by lay counsellors in primary care: 12-month follow-up of a randomised controlled trial
Source: PLoS Med. 2017 Sep 12;14(9):e1002386. doi: 10.1371/journal.pmed.1002386 (PMC5595289; doi:10.1371/journal.pmed.1002386)
Supplement: S6 Table — (DOCX) [file pmed.1002386.s009.docx]

**S6 Table: Means and 95% Cis for key variables used in mediation analyses.**

| **Variable** | **CAP + EUC (N=188)*** | | **EUC (N=189)*** | |
| --- | --- | --- | --- | --- |
|  | Mean | 95% CI | Mean | 95% CI |
| AUDIT Scores at Baseline | 14.75 | 14.44 to 15.05 | 14.99 | 14.69 to 15.30 |
| Readiness to Change at Baseline | 4.25 | 4.11 to 4.39 | 4.21 | 4.07 to 4.36 |
| Depression Scores at Baseline | 5.03 | 4.38 to 5.68 | 5.49 | 4.76 to 6.21 |
| Readiness to Change at 3-months (N=151) | 4.81 | 4.47 to 5.14 | 4.37 | 4.02 to 4.72 |
| Total Daily Ethanol Consumption over past 14 days | 314.97 | 238.43 to 391.52 | 381.06 | 311.37 to 450.75 |
| Mean Daily Ethanol Consumption over past 14 days | 22.50 | 17.03 to 27.97 | 27.22 | 22.24 to 32.20 |
