# Supplementary material for: Sustained effectiveness and cost-effectiveness of Counselling for Alcohol Problems, a brief psychological treatment for harmful drinking in men, delivered by lay counsellors in primary care: 12-month follow-up of a randomised controlled trial
Source: PLoS Med. 2017 Sep 12;14(9):e1002386. doi: 10.1371/journal.pmed.1002386 (PMC5595289; doi:10.1371/journal.pmed.1002386)
Supplement: S7 Table — Beta estimates (β) are unstandardised. Multiple linear regression models controlled for baseline AUDIT score, baseline PHQ-9 score, where the intervention was delivered (primary health centre) and who delivered it (health counsellor), and patient education. µp ≤ 0.10. *p < 0.05. ***p < 0.001. (DOCX) [file pmed.1002386.s010.docx]

**S7 Table: Mediation results examining patient-reported readiness to change at 3-months on mean daily drinking outcomes at 12-months (N=151).**

| **Model** | **Regression Result** | | **Bootstrap 95% CI** |
| --- | --- | --- | --- |
|  | β* | SE | -13.99 to -0.046 |
| *c*’ (CAP 🡪 daily drinking at 12-months) | -11.131^µ^ | 6.663 |  |
| *a*’ (CAP 🡪 readiness to change at 3-months) | 0.880* | 0.451 |  |
| *b*’ (readiness to change at 3-months 🡪 daily drinking at 12 months) | -6.834*** | 1.879 |  |
| a x b | -6.041 | |  |

Note. Beta estimates (β) are unstandardized. Multiple linear regression models controlled for baseline AUDIT scores, baseline PHQ-9 scores, where the intervention was delivered (PHC) and who delivered it (Health Counsellor), and patient education.
^µ^*p*≤.10. **p*<0.05. ***p*<0.01. ****p*<0.001
