# Supplementary material for: Sustained effectiveness and cost-effectiveness of Counselling for Alcohol Problems, a brief psychological treatment for harmful drinking in men, delivered by lay counsellors in primary care: 12-month follow-up of a randomised controlled trial
Source: PLoS Med. 2017 Sep 12;14(9):e1002386. doi: 10.1371/journal.pmed.1002386 (PMC5595289; doi:10.1371/journal.pmed.1002386)
Supplement: S8 Table — (DOCX) [file pmed.1002386.s011.docx]

**S8 Table: Mean costs (2015 International Dollars) and QALYs gained per person over 12 months**

| **Type of Cost** | **EUC+CAP (n=188)** | **EUC (n=189)** | **Mean Difference (95% CI)** | **p** |
| --- | --- | --- | --- | --- |
| **CAP Intervention Costs** | | | | |
| CAP Intervention (SE) | 33.50 (2.19) | 0 (0) | 33.50 (29.19, 37.81) | <0.001 |
| **Health Service Utilisation** | | | | |
| PHC Doctor Consultations (SE) | 35.82 (3.55) | 61.67 (31.99) | -25.84 (-89.33, 37.64) | 0.42 |
| Hospital Doctor Consultations (SE) | 25.78 (6.05) | 21.65 (3.34) | 4.13 (-9.46, 17.74) | 0.55 |
| Hospital Admissions (SE) | 49.28 (9.96) | 73.64 (14.30) | -24.36 (-58.64, 9.91) | 0.16 |
| Laboratory Tests (SE) | 18.85 (2.66) | 25.50 (3.88) | -6.65 (-15.90, 2.61) | 0.16 |
| Medicines (SE) | 16.35 (2.28) | 24.54 (3.38) | -8.18 (-16.20, -0.15) | 0.05 |
| Total Health Service Utilisation Costs (SE) | 146.08 (14.79) | 206.98 (36.92) | -60.90 (-139.24, 17.43) | 0.13 |
| **Total Health System Costs** | | | | |
| Total Health System Costs (SE) | 179.59 (15.01) | 206.98 (36.92) | -27.40 (-105.90, 51.10) | 0.49 |
| **Productivity Costs** | | | | |
| Time costs to service users and families (SE) | 60.41 (7.62) | 66.79 (8.36) | -6.37 (-28.62, 15.88) | 0.57 |
| Productivity losses (SE) | 244.31 (73.21) | 433.65 (126.66) | -189.34 (-477.25, 98.56) | 0.20 |
| **Total Societal Costs** |  |  |  |  |
| Societal perspective (SE) | 484.31 (76.92) | 707.43 (132.17) | -223.12 (-524.05, 77.82) | 0.15 |
| **QALYs** | | | | |
| QALYs gained (SE) | 0.9013 (0.0035) | 0.9007 (0.0034) | 0.0006 (-0.0091, 0.0102) | 0.91 |
