# Supplementary material for: Sustained effectiveness and cost-effectiveness of Counselling for Alcohol Problems, a brief psychological treatment for harmful drinking in men, delivered by lay counsellors in primary care: 12-month follow-up of a randomised controlled trial
Source: PLoS Med. 2017 Sep 12;14(9):e1002386. doi: 10.1371/journal.pmed.1002386 (PMC5595289; doi:10.1371/journal.pmed.1002386)
Supplement: S2 Text — (PDF) [file pmed.1002386.s013.pdf]

Counselling For Alcohol Problems (CAP)

2013

Authors

Hamid Dabholkar

Abhijit Nadkarni

Richard Velleman

Vikram Patel

Acknowledgements

The authors would like to acknowledge the following:

- Dr Shanti Ranganathan, Dr Pratima Murthy and Dr Jim Mc Cambridge for their valuable inputs which helped to enhance the content of this manual.
- The investigators group, including Betty Kirkwood, Chris Fairburn, Michael King, Atif Rahman, Ricardo Araya, Pratima Murthy, Helena Verdeli, Terry Wilson, Mark Jordans, Richard Velleman and Vivek Benegal for their involvement throughout the treatment development of the Counselling for Alcohol Problems (CAP).
- Bindiya Chodankar and Kishore Kalokhe for their tireless efforts which (alongside the authors) provided the clinical data to inform this manual.
- Sachin Shinde, Madhumita Balaji and Benedict Weobong for coordinating the research work which helped us to contextualise this manual to the cultural setting.
- The health counsellors at the frontline of implementing the manual and in doing so helping us to fine-tuning its contents.
- The General Practitioners, Parivartan (Satara) and Directorate of Health Services, Goa for allowing us access to their facilities to conduct the clinical and research activities which informed this manual.
- The doctors, nurses and other staff of the PHCs who welcomed us into their clinics and supported us in our clinical and research activities.
- The participants in the treatment development workshops, case series and pilot studies without whom this manual would not have come to being.
- Dielle D’Souza for help in formatting and technical assistance.
- The PREMIUM administrative team who worked silently in the background supporting and encouraging us in developing this manual.

The work that has led to this manual has been entirely funded by the Wellcome Trust through a Senior Research Fellowship grant to Vikram Patel.

TABLE OF CONTENTS		
**Sr No**	**Chapter**	**Page No**
1	Understanding Harmful Drinking	5
2	An Introduction to the Counselling for Alcohol Problems (CAP)	12
3	The Style of a CAP Counsellor	19
	CAP SESSION BY SESSION GUIDE	31
4	A: The General Format of All Sessions<br>• Four Tasks: Developing an engaged relationship, Working together, Helping to set the agenda, and Building the patient's motivation to change<br>• Structure of each session	32
5	B: Initial Phase (Session One – Getting Started)<br>• Introduce CAP to our patient<br>• Set the agenda<br>• Help our patient to better understand his drinking, based on initial AUDIT assessment<br>• Give personalised feedback to our patient about his drinking<br>• Help our patient to decide on his drinking and other goals<br>• Working with our patient on developing a change and action plan<br>• Special situation: Providing a very short version of Session One	37
6	C: Middle Phase (Session Two – Helping the Patient to Change)<br>• Reviewing progress<br>• Problem solving<br>• Drink refusal skills<br>• Handling urge to drink<br>• Handling your difficult emotions	65
7	D: Ending Phase (Session Three and Four – Dealing with Relapses and Ending Well)<br>• Preventing and dealing with relapse<br>• Ending well	89
8	Dealing With Challenges	103
9	Dealing With Patients Having Depression As Well As Drinking Problems	112
10	Dealing With Patients Having Tobacco Problem As Well As Drinking Problems	115
	Appendices	
	(i) Appendix 1: Glossary	120
	(ii) Appendix 2: Referral Pathways	122
	(iii) Appendix 3: Role of Medication	123
	(iv) Appendix 4: AUDIT	124
	(v) Appendix 5: Quality of CAP (Q-CAP)	

Introduction

The Counselling for Alcohol Problems (CAP) manual aims at providing counsellors with information about counselling patients with Harmful or Dependent Drinking, in primary care settings. It is designed to accompany the Counselling Relationship manual.

This manual has been developed by the PREMIUM team as part of a five-year project that seeks to develop and evaluate culturally appropriate psychological treatments for two priority mental health conditions – depression and harmful/dependent drinking - that can be delivered by counsellors in primary health clinics. While there are different ways to help people overcome harmful/dependent drinking problems, our preparatory research has demonstrated that the type of counselling treatment which is most likely to be effective in our setting is one that uses a mixture of motivational approaches, and approaches that help people to change their thinking about, and behaviour towards, drinking alcohol. In addition to working with people on improving their motivation to address drinking problems, CAP aims to help people change the way that they behave – what they do – so that they stop having problems related to their drinking. We help people plan to change what they do, and we then get them to practice these different ways, both in our sessions with them, and outside the sessions by getting them to do homework. Getting people to think and talk about their drinking and how to change it is important – but the most important thing is that they actually do change, not just talk about changing.

The manual has 10 separate chapters. We have emphasised a number of salient points within the text and key concepts are illustrated with the use of case examples and scripts. We provide a step-by-step guide for each phase of the treatment and include exercise charts and hand-outs that the counsellor can use with the patients. Counsellors may face significant challenges during the course of their work and we have recommended solutions that can be implemented to overcome them.

This manual is meant to act as a practical guide that counsellors can refer to on an on-going basis to develop and strengthen their skills in helping patients with Harmful or Dependent drinking.

Chapter 1

Understanding Harmful Drinking & Alcohol Dependence

Learning Objectives

In this chapter, we will learn:

- What are some warning signs of drinking problems?
- When does drinking become ‘problem drinking’ and what are the types of problem drinking?
- Why does some people’s drinking become Harmful or Dependent?
- What is the impact of Harmful or Dependent Drinking?
- What are the treatments for Harmful and Dependent Drinking?

CONTENT

INTRODUCTION

Raja is a 32-year-old married man who used to work as a driver until he lost his job recently. Initially he used to drink beer with friends at the local bar. This used to happen once every two or three months, but after losing his job his drinking has increased to two or three times a week, and when he drinks, he does so for longer – sometimes drinking all day. Drinking alcohol has now started affecting his health too. After drinking for two days at a stretch he developed stomach pain and lost his appetite. He has come to the PHC with his wife to get treatment for his stomach problem and decreased appetite.

Amit is a young man of 20 years who goes to the local college. He is very popular among his friends and likes to party every weekend. On Saturday and Sunday evenings he goes out with friends to clubs and parties through the night. He drinks up to a bottle of vodka on each of these nights. Sometimes he also drinks 1-2 cans of beer on weekdays too. Very recently his professors have commented that his work at college has not been as good as it used to be and he has been missing college frequently. Last night, on his way back from a party, he had an accident and injured his hand. He has come to the PHC with his brother for dressing of his injuries.

Joseph is a 65-year-old male who lives by himself. He is a retired policeman. His wife divorced him 10 years ago as they used to have a lot of arguments and sometimes he used to beat her. He has been drinking 1-2 pegs of feni (a type of country liquor available in Goa) 3-4 times a week for the past 40 years. Since his retirement five years ago he has been drinking a quarter of feni every evening before going to bed. He was diagnosed with diabetes two years ago and has been on treatment for the same. Despite being on continuous treatment, his blood sugar has been poorly controlled. Recently he developed an ulcer of the foot as a complication of his diabetes and it has not been healing despite treatment.

Rashid is a 45-year-old man who lives with his wife. He started drinking alcohol when he was 18 years old and was initially drinking a couple of bottles of beer on weekends but this increased to drinking every day, which he has been doing for the past 15 years. His drink of choice has also changed over this time, from beer to feni, and he now drinks a bottle of feni every day. He has his first drink after he wakes in the morning and then continues to drink through the day. If he does not drink in the morning his hands start shaking and he is unable to do any work. He lost his job a few years ago because of his drinking. He gets into arguments with his wife and many times he ends up beating her. A few months ago he got admitted to hospital after he started vomiting blood. Last week he did not drink any alcohol for two days because of a religious festival and developed fits for which he was brought to the PHC.

We may encounter patients like Raja**,** Amit**,** Joseph and Rashid during our work as a counsellor in the PHC. This section of the manual is designed to introduce us to their problems.

Though the immediate problem that each of these patients is coming to the PHC for is physical (stomach problem and decreased appetite, hand injury, foot ulcer, diabetes and fits), as you must have guessed these problems are caused by their excessive consumption of alcohol. Excessive drinking is causing them physical and social harm**.**

WHATARE SOME WARNING SIGNS OF DRINKING PROBLEMS?

Some signs which should warn us of drinking problems are when one(‘s):

- Regularly uses alcohol to feel confident
- Drinks to get drunk
- Drinks alcohol to cope with anger, frustration, anxiety or depression
- Is not able to socialise without a drink

- Can drink a lot without becoming drunk
- Needs to drink more and more to feel good
- Drinking affects one's relationships with other people
- Carries on drinking even though it is interfering with work, family and relationships
- Misses days at work due to hangover / takes a lot of sick leave to recover from the effects of alcohol
- Drives occasionally under the influence of alcohol
- Drinking makes one feel disgusted, angry, or suicidal
- Struggles at work because of hangovers
- Hides the amount one drinks from friends and family
- Gets angry if challenged about one's drinking
- Favoured drink changes from drinks with lower alcohol content to those with higher alcohol content e.g. beer to brandy
- Still drinks despite having been advised by a doctor / counsellor to stop or reduce drinking as it is harming health or social life
- Is unable to say 'no' when offered a drink
- Vomits or gets pain in the stomach after an episode of drinking
- Gets "memory blanks" where one can't remember what happened for a period of hours or days after drinking
- Tries to stop, but can't
- Gets very shaky, sweaty, and tense a few hours after the last drink
- Stops drinking for a short period but gets back to excessive drinking again very speedily
- Needs a drink to start the day

The more of these that someone has, the more likely it is that he has a drinking problem; and the ones further down the list are more indicative of greater problems.

What are the reasons for drinking alcohol?

We frequently encounter people who drink alcohol, and there are lots of reasons why people drink. Some common reasons are:

- To relax
- To join in with others in social occasions
- Enjoyment of taste
- Enjoyment of how it makes a person feel
- Pressure from friends
- Makes socialising easier
- To cope with stress
- To reduce physical pain
- To help with sleep

WHEN DOES DRINKING BECOME PROBLEM DRINKING AND WHAT ARE THE TYPES OF PROBLEM DRINKING?

The fact that people drink alcohol for any of these reasons does not mean that they will develop problems. But some people do, and it is these people that we need to help with our counselling. When a person starts drinking in a way which negatively affects him or his life (such as affecting his health, or his job, or his relationships) then this is considered to be problem drinking.

Depending on severity, there are at least three different types of drinking which might cause us concern, showing increasing severity. It is important for us to understand these types clearly because there are different approaches to helping each of these types.

- When a person drinks in a way which increases their risk of them developing some physical, mental or social harm in the future, it is called **Hazardous Drinking**.
- When a person drinks in a way which has already started causing some physical (e.g. liver damage), mental (e.g. depression) or social harm (e.g. arguments with family or friends), it is called **Harmful Drinking**.

- **Dependent Drinking** is the most severe drinking problem and usually involves daily drinking. It includes a combination of harmful behaviours (e.g. continued heavy drinking despite clear evidence of harmful consequences), harmful thoughts (e.g. strong desire to drink alcohol) and physical signs (e.g. *withdrawal symptoms*' [See *Appendix 1*] such as sweating, nausea, and in serious cases uncontrollable shaking, vomiting, or fits, if they do not drink).

In PREMIUM, we will be focusing on patients whose drinking is Harmful or Dependent, for three important reasons:

- There are a lot of men in Goa (and in the rest of India) with such drinking problems
- There are effective brief psychological treatments for these drinking problems, which can be delivered by counsellors like you, who have appropriate training and supervision
- Although these effective treatments for patients with Harmful or Dependent drinking exist, there is hardly any provision of them in developing countries like India

WHY DOES SOME PEOPLE'S DRINKING BECOME HARMFUL OR DEPENDENT?

Lots of people drink alcohol and never develop any problem. They may drink alcohol at some social occasions, or they may drink at home, but their drinking does not cause problems, either for themselves or for other people.

But for some people, their drinking does become problematic. There is no single reason for this which applies to everybody.

- For some people, drinking relieves stress or worry that they may have, and they start to rely on the drinking instead of finding other ways to deal with the anxiety
- For other people, their drinking gradually increases and they do not realise that they are developing a habit of drinking that is difficult to cut down or stop
- For yet others, they become so used to drinking heavily at social occasions such as family parties that they cannot face a party without drinking
- For yet others, they suffer some event in their lives (such as a bereavement or losing their job) which triggers them to start drinking more problematically

However, although there is no one reason which applies to everybody, there is always a reason (or a set of reasons) which applies to any individual patient – so when we work with a Harmful or Dependent Drinker, one of our tasks is to help the patient understand why he is drinking so heavily, and therefore what he needs to change so that he can cut down or stop his drinking.

WHAT IS THE IMPACT OF HARMFUL OR DEPENDENT DRINKING?

Alcohol affects different spheres of life. As we observed in Raja's, Amit's, Rashid's and Joseph's cases, alcohol affected their physical health. But the first signs can occur in any of a range of different spheres: mental health, family life, physical health, work or social functioning. Importantly though, as the severity of someone's drinking problem increases, it starts affecting more and more of these spheres, so gradually people start to show problems in all of them.

Harmful or Dependent Drinking can affect a person's life in many ways. It can affect

- Physical Health: As we can see from the picture below, alcohol can negatively affect almost every part of the body.
- Mental Health: Harmful or Dependent Drinking can cause *depression* [See *Appendix 1*] (Persistent sadness and loss of interest with reduced sleep/appetite, guilt feelings, feelings of hopelessness, etc.), anxiety (fear or worry without any apparent reason), suicidality, loss of memory and *hallucinations* [See *Appendix 1*] (hearing voices or seeing visions even when one is alone).
- Social and Family Life: Harmful and Dependent drinking can cause arguments and fights, debts and other financial problems, neglect of parental responsibilities, missing of family functions and legal problems.
- Functioning at work: Harmful and Dependent Drinking can cause absenteeism, frequent job changes, lack of efficiency, fights and arguments, and accidents and injuries.

- Intoxication: Many people get slightly intoxicated when they drink alcohol – a slight feeling of light-headedness, and feeling less inhibited. But many Harmful and Dependent Drinkers will become much more intoxicated. They will show much larger signs of intoxication - mood changes, a loss of social inhibitions, slurred speech, and loss of balance are all important signs of intoxication due to alcohol. Many of the problems associated with Harmful or Dependent Drinking are caused by drinking alcohol to a high level of intoxication – problems such as aggression, vandalism, fights, domestic violence, drunken driving, and alcohol-related accidents.
- Withdrawal symptoms: Only people with Dependent Drinking get these – they are one key sign that someone's drinking is dependent. When such people suddenly reduce or stop their drinking they often experience some or all of the following symptoms - disturbed sleep, excessive sweating, heartbeat speeding up, raised blood pressure, restlessness, headache, weakness, shakiness of the hands, vomiting, seeing / hearing imaginary things, and fits. Experiencing more of these symptoms, and to a greater severity, is a sign of a higher level of dependence.

HOW DO WE KNOW THAT SOMEONE HAS HARMFUL OR DEPENDENT DRINKING?
In PREMIUM, we are using the Alcohol Use Disorders Identification Test (AUDIT) [See *Appendix 4*] as the tool to assess a person's drinking behaviour.

- The AUDIT is a set of 10 simple questions. It gives scores which range from 0 to 40.
- If a person scores 12 to 19, it suggests that they are likely to be in the Harmful Drinking category, and if they score 20 or more, it suggests that they are likely to be Dependent Drinkers.
- The AUDIT will be completed by a Health Assistant before the patient comes to see us and only those patients who score 12 and above (Harmful and Dependent Drinkers) will be referred on to us.

Figure 1: Long-term health effects of alcohol

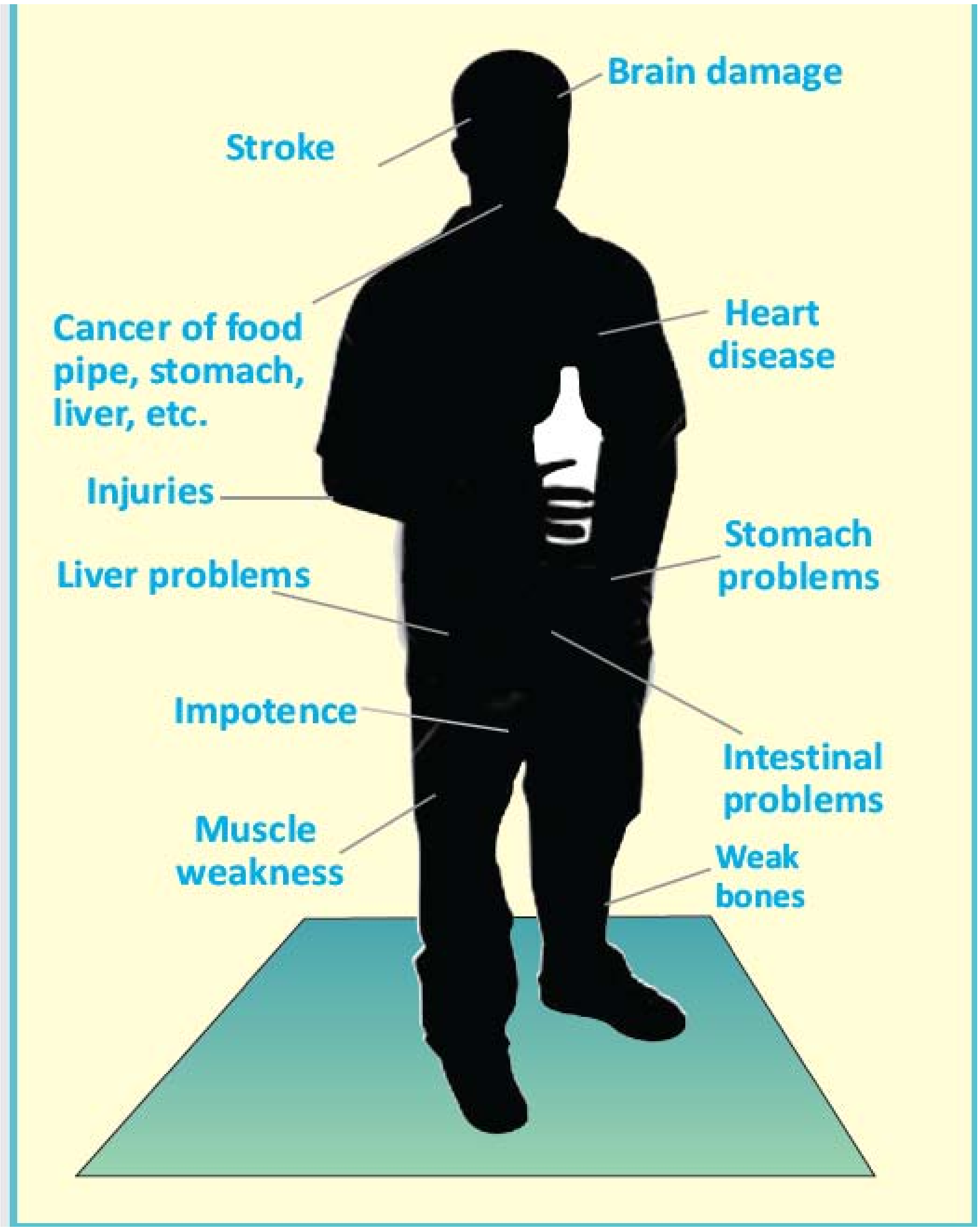

WHAT ARE THE TREATMENTS FOR HARMFUL AND DEPENDENT DRINKING?

The most common and effective way to treat harmful and dependent drinking is by counselling, where help is offered by talking to patients about their drinking problem.

In this manual, we will learn how to deliver CAP. This form of counselling seeks to change harmful and dependent drinking by helping people to evaluate their drinking habit, develop motivation to address the drinking problem, and make changes in both their drinking and the reasons that they are drinking. It helps them plan what they want to change, and then helps them to make and maintain those changes.

CAP helps both Harmful and Dependent drinkers. However, because Dependent drinkers may need other help (such as medicines to help their withdrawal symptom) we will also refer Dependent drinkers to a specialist Consultant Psychiatrist, who will work with the CAP counsellor to provide whatever additional help is needed for those Dependent drinkers. This might involve either some specific medicines, or even an in-patient stay. We won't do this with the Harmful drinkers because

they do not require such kind of treatments. However, harmful drinkers might have certain alcohol-related physical health problems like disturbed sleep or reduced appetite which might benefit from medicines. The doctor might also do some blood tests in Harmful as well as Dependent Drinkers to find out the extent of damage caused to the patient's body by alcohol. With patients who are Dependent drinkers, even where medicines are used, outcomes are much better if the patient is also engaged in counselling. [See *Appendix 3*]

SUMMARY

- People drink alcohol for various reasons but everyone who drinks does not develop alcohol problems.
- Problems due to drinking alcohol can be categorised into hazardous, harmful and dependent drinking.
- Harmful and Dependent drinking can affect various areas of a person's life including physical health, mental health, family life, social functioning, and work.
- Harmful and Dependent drinking are the target groups for PREMIUM
- The most common and effective way to treat both Harmful and Dependent drinking is by counselling. Patients who have Dependent drinking will also be referred to a Consultant Psychiatrist who will assess whether that patient might also require medicines to be prescribed, or even need an in-patient stay.

Chapter 2

An Introduction To Counselling For Alcohol Problems (CAP)

Learning Objectives

In this chapter, we will learn:

- How do people stop or reduce their drinking?
- What is CAP?
- What happens in sessions 1-4 of CAP

CONTENT

HOW DO PEOPLE STOP OR REDUCE THEIR DRINKING?

There are different ways:

- Most people stop or reduce their drinking without any help. Others need help to do so and some who can change on their own can also benefit from help.
- For those that need help, with some the only help they need is from a supportive family member or friend, whereas others need professional help. These are the patients that we will see in PREMIUM.
- Some people can stop or reduce their drinking suddenly without any problems. Others cannot, as they develop ‘withdrawal symptoms’ – craving (intense desire for alcohol), shakiness, restlessness, fits etc. If this happens, a doctor’s help should be sought. The doctor can often help with some medication for a short time to prevent these problems. This can be done at home or in hospital.
- As well as medications to help withdrawal symptoms, there are also a few medications which can help people to cut down or stop their drinking. They are Disulfiram, Acamprosate, Baclofen and Naltrexone (See *Appendix 3* for details). These should be taken only on the advice and under the supervision of a doctor. Even when one is taking one of these medications, counselling plays an important role in the treatment.
- In the longer term, working with trained counsellors can help to tackle the problem. Working with the counsellor helps to develop and maintain motivation to change drinking behaviour as well as to develop skills to counter situations which lead to drinking.
- There are many useful things that patients can do to help themselves. In CAP we will work with the patient to help them try some of these listed below.

If a patient is trying to cut his drinking down, there are useful things he can do, including:

<u>Drink on fewer occasions</u>

- Work out when and why he drinks and plan to do something else instead
- Plan ahead each week which days he will avoid alcohol. He should have at least two alcohol-free days a week
- Not attend social events where alcohol is served

<u>Drink fewer alcoholic drinks</u>

- The patient needs to pace himself – he can plan how long he will be out and how many drinks he will have, and then he needs to stick to his plan
- Introduce some drinking rules, e.g. don’t drink before 8pm
- Get him to pace his drinking. “Sip, don’t gulp”
- When drinking, take smaller sips
- Put the glass down between sips
- The patient should occupy himself – don’t just drink but participate in other activities, e.g. playing a game, reading, talking or eating
- Avoid joining in buying rounds of drinks - or when it is the patient’s round, have a non-alcoholic drink (e.g. soft drink, fruit juice)
- Try to drink at the same pace as a slower-drinking friend
- At home, don’t finish the bottle – keep some for another day
- Learn and practice how to refuse a drink. The patient needs to decide for himself when and how much he wants to drink

<u>Reduce the amount of alcohol in each drink</u>

- Not all brands of alcohol are the same strength. Switch from a higher alcohol content to a lower one, e.g. from feni to beer, or from a strong beer to a lower strength one
- Switch to smaller measures:
  - from a large peg (60 ml) to a small peg (30 ml)
  - from large bottles of beer (650 ml) to small ones (330 ml)
  - use a smaller glass at home

- Eat before and while drinking. Food makes the body absorb alcohol more slowly and so limits how quickly it gets into the bloodstream. It also reduces the chances of developing acidity caused by alcohol

Overall

- Get the patient to involve a family member or a friend. They can help to support the patient to keep to drinking less and controlling his drinking, and keep track of his progress
- Get the patient to talk through problems and worries. Stress and worries can influence how much one drinks. Talking about these stresses and worries can be a good first step to resolving problems with alcohol

If a patient is trying to stop drinking, there are useful things he can do, including:

- Work out other enjoyable or interesting things to do, instead of drinking
- Avoid high-risk situations for drinking, like going to the bar or wine shop or socialising with others who drink. Don't go out with friends when you get paid for the week, or at other times when you have a lot of money with you
- Learn and practice how to refuse a drink
- Involve a family member or a friend. They can help to support the patient to keep to not drinking, and keep track of progress
- Talk through problems and worries. Stress and worries can influence how much one drinks. Talking about these stresses and worries can be a good first step to resolving problems with alcohol

(If you are explaining these strategies to the patient, then you can use the hand out **Some Strategies To Reduce Or Stop Drinking** on pg 10 of the patient booklet)

WHAT IS CAP?

Counselling for Alcohol Problems (CAP) was developed in the PREMIUM programme. It is a treatment that we have developed for use with Harmful and Dependent Drinkers like Raja**,** Amit, Rashid and Joseph. It is based both on successful methods used in the west as well as the experiences of working with people with harmful and dependent drinking in India. The treatment is brief: most of the times it will be limited to just two sessions, and the maximum number of sessions will be four. It uses many of the tips outlined above, to help patients with Harmful or Dependent drinking to change. It is important to remember that, although this treatment has some written material and written homework, it is equally helpful even for patients who are illiterate.

WHAT HAPPENS IN THE PHASES AND SESSIONS OF CAP?

CAP is a treatment which has a number of phases, and when we counsel someone, we go through these phases in order. There is

- An ***Initial Phase***, where we help the patient understand whether his drinking may be causing problems and may need to change
- A ***Middle Phase*** where we help the person to make those changes
- And an ***Ending Phase*** where we help the patient plan to deal with any potential or actual *lapses or relapses* [See *Appendix 1*], and we end our treatment.

In many cases, we will cover all of the *Initial Phase* in the first session, and all of *Middle Phase* (and often the *Ending Phase*) in the second session.

- But with some patients our work may move faster, and we may cover all of phases 1 and 2, and sometimes phase 3, in one session (although this will be very rare);
- And with other patients our work may move more slowly, and it may take 2 sessions to work through all of phase 1, and we may need to use up to two extra sessions to work through phases 2 and 3.

So, the key to our approach is flexibility, tailoring our counselling to the specific needs of the particular patient we are working with (i.e. what we might call a personalised approach).

Things that we do throughout all the phases of CAP

People change their drinking because

- They are *motivated to change*
- They have the *confidence* to change
- They have *decided* to change, and
- They have *the skills* to behave and to think differently

When we examine the different phases in detail, we look at skills to help people think and behave differently. But because change is difficult for many people, throughout each of our sessions we have to help our patients to develop and maintain their motivation to change, their confidence that they can do so, and to reinforce their decision to make changes. Helping our patients to do these things (be motivated, feel confident, and be decided) are the most important tasks that we have, which we undertake at all times, throughout all of the phases.

We help our patients to develop and maintain their motivation to change, and their confidence that they can do so, by

- Developing engagement
- Working together,
- Developing and encouraging *change talk*, and
- Identifying and discouraging *sustained talk*

All of these terms will be explained later in the manual, when we look in detail at each phase.

Another element that we try and develop throughout is to involve a 'Significant Other' (SO) (usually a family member or a close friend) in the treatment (as long as the patient agrees that they want this 'significant other' to be involved). We involve an SO in order to help the patient achieve whatever goals we agree on: as outlined in the Counselling Relationship manual.

Things that we do in individual phases of CAP

Besides these elements that we undertake throughout all the phases, we will also do the following specific activities in individual phases:

Initial Phase (usually session one)

In this initial phase we help the patient understand that his drinking may be causing problems and may need to change. We do this by:

- Helping the patient (and his SO, if the patient wants an SO to be present and if this SO has come to this Initial Phase) to get a better understanding of his drinking. We do this based on the initial AUDIT (*Appendix 4*) screening, and we ask other assessment questions if we need to so that both the counsellor and the patient can understand how his drinking is affecting him and his life
- As a result of this better understanding of his drinking, we give the patient *personalised feedback* [See *Appendix 1*] about his drinking (that is, we let him know how and why his drinking may be harmful, and how it may be related to any problems or issues that he may have told us about, during the assessment)
- We then help the patient (and his SO) to decide on what he wants to do about his drinking: whether he wants to change and if so what his drinking goal is (that is, does he want to stop drinking entirely, or to reduce it in some way, or to exercise more control over his drinking?)
- Finally, we help the patient (and his SO**)** to formulate both a *Change Plan* [See *Appendix 1*] - a set of decisions which summarises what he wants to do to change his drinking and its related problems, and an *Action Plan* [See *Appendix 1*] – the actual actions that the patient and the counsellor have agreed will be done in between one session and another

Middle Phase (usually session two)

In this middle phase we help the person to make those changes. So, in this session we:

- Discuss with the patient (and his SO) what has happened with the patient's drinking in between the first session and this present one
- Look again at the change plan and clarify whether this is still what the patient and his SO want to do
- Help the patient to implement the change plan – we work out with the patient (and the SO) how he is going to implement the change plan. This will also help us get clear what new skills

and techniques the patient needs in order to make the changes that the change plan calls for (that is, we work with the patient on *thinking and behavioural skills*, explained later)

Ending Phase (often session two, sometimes session three, less often running into session four)

In this ending phase we will

- Work with the patient (and his SO) on relapse prevention and management (we help the patient plan how he will deal with any potential or actual relapses)
- End our treatment (by reviewing the skills and strategies that the patient has learnt, clarifying any skills or information that are not clear, and reinforcing the patient's motivation to use these skills and strategies across other areas in his life)

Ensuring treatment quality

A rating scale, Quality of CAP (Q-CAP) will be used to assess treatment quality during group supervision sessions. In each supervision session, the scale will be rated by an expert supervisor, a peer supervisor and peers.

Figure 2: Phases of CAP

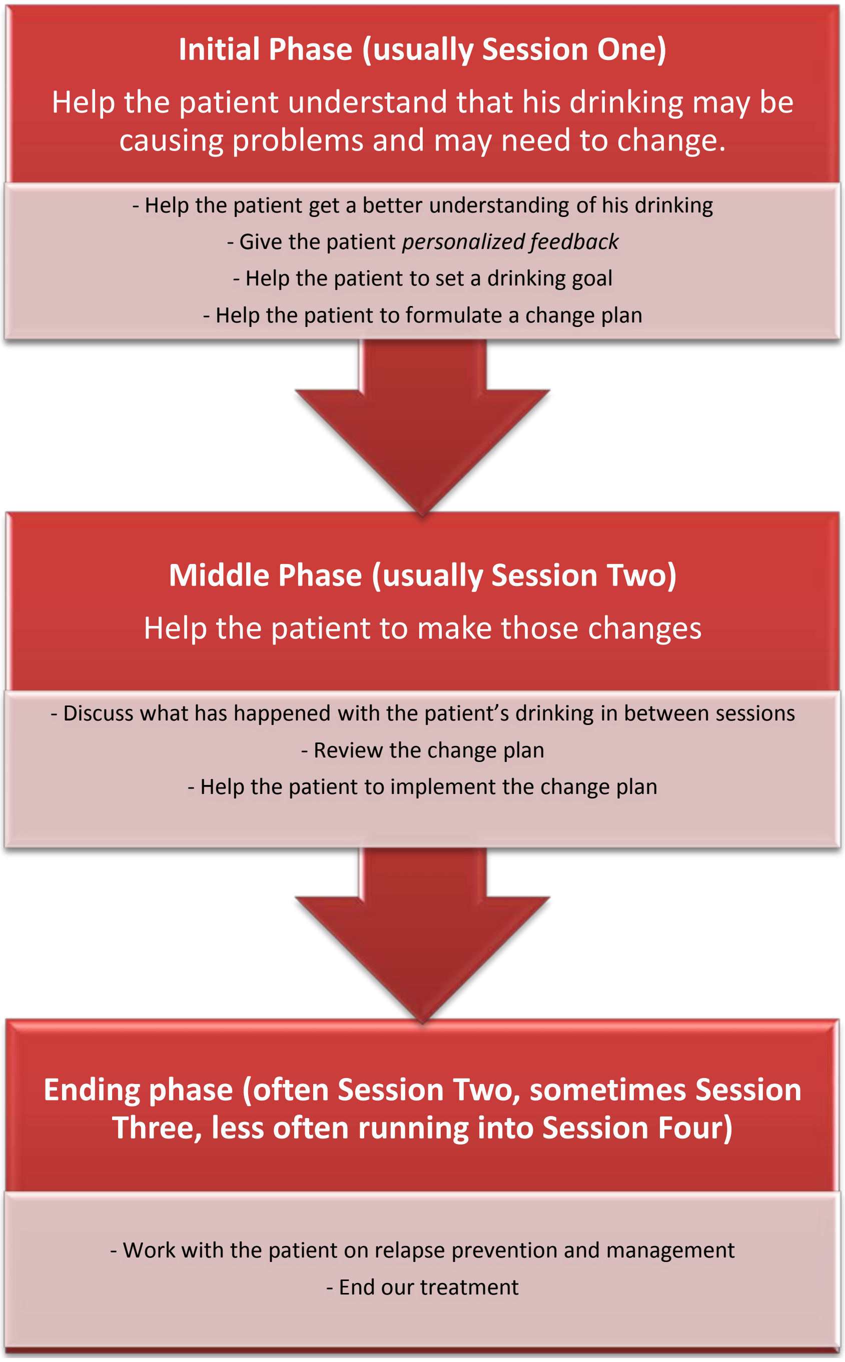

We will often explain this structure to our patients early on in our first session:

'There are three parts to the counselling that we do here:

- *In the first part, we get to know each other, discuss your drinking and whether it has given rise to any problems, and we decide together what you want to do about it*
- *In the second part, we plan out together how you are going to make any changes that you want to*
- *In the third part, we work out how to make those changes long-lasting*
- *We will meet a maximum number of four times, although we may get these three parts competed faster – with some people it only takes one or two meetings, with others, three, or all four that we have available.'*

SUMMARY

- There are many useful things that people can do to stop or reduce their drinking
- CAP is a psychological treatment for Harmful and Dependent Drinking that includes many of these techniques
- CAP is based on successful methods used in the West and the experiences of working with patients in India
- CAP has three phases, which could all be undertaken in one session, more usually will take two sessions, and in some cases may take up to a maximum of four sessions
- The key overarching task undertaken throughout CAP is developing the patient's motivation to change his drinking behaviour and his confidence that he can
- It is important to involve a significant other (SO) in the treatment both as a strategy to develop the patient's motivation and as a support for the changes that the patient wants to make
- Other key tasks in the *Initial Phase* are assessment, personalised feedback, deciding on the drinking goal and deciding on the change plan
- The key task in the *Middle Phase* is to help the patient implement his change plan, by clarifying what skills are needed, helping the patient to develop the skills that he does not have, and helping him to use his skills to make changes
- The key tasks in the *Ending Phase* are to help the patient to develop relapse prevention and management skills, and then to end our counselling positively

Chapter 3

The Style Of A CAP Counsellor

Learning Objectives

In this chapter, we will learn:

- What style of counselling we should adopt in CAP
- That we need to ensure that we:

- Develop an engaged relationship with the patient (and the SO if they are present)
- Work together (collaborate) with the patient (and the SO if they are present)
- Promote independence by allowing the patient to make decisions rather than making them ourselves, while still guiding the patient and the session (navigating)
- Build and develop the patient’s motivation to change

CONTENT

All the skills we learnt in the earlier module on general counselling skills [see the Counselling Relationship Manual] are applicable to counselling patients with harmful drinking using CAP**.** But there are some key aspects of the counselling style that are particularly important in CAP and these are highlighted below.

- Throughout, we work to develop an *engaged* relationship with the patient (and the SO if they are present)
- We work together (or *collaboratively)* with the patient (and their SO as well)
- We allow the patient to make decisions rather than making them ourselves – we promote independence
- But also, we as counsellors must guide the process – *navigation.* We need to ensure that the key tasks are undertaken and the structure of the session is followed
- As counsellors, we must not be afraid to have our own views about what might help a patient – but we must express them collaboratively and respectfully, and never impose our views over the patient's view. Giving advice (in a collaborative way) is part of CAP; but forcing the patient to accept our advice or telling patients what they should do is not part of CAP

These key aspects are shown by our use of many of the skills which we described in the Counselling Relationship manual. In order to effectively engage, collaborate, promote independence, navigate and guide the sessions, and express our views collaboratively and respectfully, we must always:

- Be non-judgmental
- Be respectful of the patient and of their SO
- Give the patient our full attention, and try hard to pick up on what he is saying and meaning (accurate empathy)
- Validate or confirm the patient's experience
- Express warmth and be genuine
- Encourage the patient to make positive (although realistic) statements about his progress or his hopes for change – evocation
- Be caring and friendly (although we are not being 'a friend')

For a more detailed description of these skills, go back and read the relevant chapter in the Counselling Relationship Manual.

The way that some of these skills fit together are so key that we will explain them further here (although bear in mind that this a repetition and summary of what we have said in the Counselling Relationship manual – CAP is using the same approach and the same skills as we have described in the Counselling Relationship manual**).**

The four key things that we need to do to form the basis of our style are described below:

1) Developing an engaged relationship with the patient (and the SO if they are present)

This is one of the most important elements in counselling people for a wide range of problems (e.g. depression, anxiety, excessive worrying etc, and of course drinking problems). We have already described in section two of the Counselling Relationship manual how we develop an engaged relationship with a patient (and you should go back to look at that manual to refresh your memory of the details), but the key elements are that the counsellor comes across to the patient as warm,

empathic and genuine. As the Counselling Relationship manual describes, there are many smaller skills that we use to show to our patients that we are warm, empathic and genuine.

Although we start to develop this relationship as soon as we have any contact with a patient, we never stop developing it. We always have to work at developing our engagement with our patients. *No matter what else we do, if a patient does not engage and does not come back to see us, we will not be able to continue to help him.*

Engaging a patient's SO

If the patient has an SO with him, then we must work equally as hard to engage her or him as well. The SO has a very important part to play in both

- Developing the patient's motivation to change, and
- In helping the patient to achieve his goals.

Also, if we do not engage the SO, she or he will not understand what we are doing with the patient, and so could work in an opposite direction and undermine the patient's motivation and his efforts to achieve his goals. So we need to ensure that the SO feels an equal part (although not a dominant one) in the counselling, and that the SO understands what the patient's problems are, and agrees with the goals that we go on to set.

We need to ask the patient:

- If they have an SO with them today, and if so, we need to encourage them to bring the SO into the session with them.
- If not, we need to encourage the patient to bring an SO with them when they come for their next session.

Counsellor:

'*Did you come to the health centre today with someone – your wife, or a parent, or a friend?*

If so,

'*Can I suggest that it would be useful for her/him to join with us? We find that it is very helpful to have people who are close to the patient involved in what we do, as it helps make sure that people at home do not say things which are different to what we agree to here in the clinic'.*

If not,

'*Can I suggest that you ask someone to come with you when you come back to see me in a week or so? We find that it is very helpful to have people who are close to the patient involved in what we do, as it helps make sure that people at home do not say things which are different to what we work out here in the clinic. We can talk at the end about who would be a good person for you to bring with you'.* (It will usually be a wife if they have one, unless their relationship is very bad; it could also be a parent, or an adult child, or a good friend).

2) Working together with the patient (and the SO if they are present)

As well as engaging with the patient, we need to work together with the patient (and his SO if she /he is attending), and to respect our patient's independence right from the beginning of the treatment, and throughout the treatment. This style of working together with the patient is also known as '*collaboration*'.

There are certain key ways which demonstrate how we work together with each patient:

- We are *respectful* of them and their views (and of their SO and their views as well)
- We give them our *full attention*, and try hard to pick up on what they are saying and meaning (*accurate empathy*)
- We allow the patient to make decisions rather than making them ourselves – we nurture *independence*
- But we also guide the process – *navigation*
- We work in *partnership* with our patient and his SO – we don't take over and we are not bossy
- We are *non-judgmental*
- We *validate* or *confirm* the patient's experience
- We *encourage* the patient's progress, and we also *encourage the patient* to make positive (although realistic) statements about their progress or their hopes for change – *evocation*
- We are *caring* and *friendly* (although we are not being 'a friend')

Using this style, the counsellor encourages the patient to take the lead role in the different aspects of treatment as shown in the example below:

Counsellor	*Mr A, as you know, you were asked some questions about your drinking alcohol. We use those questions to help us see if maybe someone is drinking too much or starting to get problems. Will it be OK if we spend some time discussing these results?*
Patient	*Yes sure. I am eager to know the results.*

In this example, the counsellor is asking the patient's permission to discuss the results rather directly telling him the results of screening. The counsellor uses the word 'we' ('*Will it be OK if we spend some time*') which indicates the spirit of working together rather than using the word 'you'.

Counsellor:

'*It's really good Mr A that you have decided to control your drinking. Have you given any thought to how we can go about this?'*

In this example, the counsellor is *encouraging* of the patient over the decision he has made (in this case, to control his drinking) and has *asked the patient* about his plans for achieving this, rather than *telling* the patient what he should do in order to achieve this goal.

The stance we want to avoid at all costs is when the counsellor plays the role of the 'boss' or the 'person in charge' of the counselling and simply tells the patient how he should change (or even orders him to change). Consider this example:

Counsellor:

'*Mr Khan, I have results of your screening for drinking problems. It is obvious to me from these results that you have to stop drinking immediately. If you continue to drink in the same way then it is very likely that you will develop serious alcohol-related problems. In order to stop drinking you really must avoid friends who drink and also avoid going to places which remind you of drinking.'*

In this third example above, the counsellor does not even ask the patient whether he wants advice or not, and his style of giving advice is to tell the patient what to do.

<u>Note</u> – we are not saying that the advice that this counsellor is offering is wrong! What is wrong is the way in which he is giving the advice.

- Research has shown that if we simply tell a patient what they should do, they often ignore it or argue with it. This style of giving advice can generate a lot of negative feelings in the patient's mind and it reduces the chances of gaining a patient's cooperation in achieving the desired change. In CAP, it is our aim to avoid using such a bossy stance.
- Our job as a counsellor is to *help* and *encourage* the patient to reach a conclusion which is going to help the patient to make his life better. Our job is to *empower* the patient so that these conclusions come from him and not directly from us. If he realises that he needs to change, he is much more likely to make those changes than if we simply tell him to make the same changes.

<u>Note</u> also that the problem in the example above is not that the counsellor offered advice. There are many times when we do need to do this - for example when we discuss with patients the results of the screening tests. But we can offer advice using a collaborative stance, and not a bossy one. Consider the earlier example of the bossy counsellor (discussing the result of the screening) and how the same information could have been given in a collaborative manner:

Counsellor	*Mr Khan, as you know, you were asked some questions about your drinking alcohol. We use those questions to help us see if maybe someone is drinking too much or starting to get problems. We've been discussing those questions and I've asked you some more questions as well - so will it be OK if we spend some time discussing what you've told me?*
Khan	*Sure. What do the results show?*
Counsellor	*These results show that the amount that you are drinking presently might be affecting your health. What do you think?*
Khan	*Hmmm. It might well be the case. When I met the doctor today he said that my liver has enlarged.*
Counsellor	*Did he say how that might have happened?*
Khan	*He said that it is probably because of the alcohol.*

Counsellor	*And do you agree with his assessment?*
Khan	*I think so. I have been drinking quite heavily for the past few months and now I find it difficult to stop.*
Counsellor	*Hmmm. Are you trying to say that you have tried to stop but have not been able to do so?*
Khan	*Exactly. It is especially difficult when my friends come over and insist that I should have a drink with them at the bar. I find it difficult to say no.*

Using the same example (Mr Khan and the results of his assessment), another way the conversation could go might be:

Counsellor	*Mr Khan, as you know, you were asked some questions about your drinking alcohol. We use those questions to help us see if maybe someone is drinking too much or starting to get problems. We've been discussing those questions and I've asked you some more questions as well - so will it be OK if we spend some time discussing what you've told me?*
Khan	*Sure. What do the results show?*
Counsellor	*First, what do you think about what you have told me? Does it make you think anything about your drinking and the effects it is having on you and your family (or on your work, etc, depending on what the results show).*
Khan	*Hmm. I had not really realised that I was drinking so much, for sure. Nor that it was affecting my health. I am not happy about that.*
Counsellor	*OK – that is helpful. Would it be useful to you if I also said what these answers suggest to me?*
Khan	*Yes, of course – that is why I am here!*
Counsellor	*I agree with a lot of what you have said. You are right that the effects on your health are worrying. And you also told me earlier that your drinking does seem to be related to your money worries and your arguments at home.*<br>• *So I think that we need to work together to decide what you want to do. Most people who are getting these effects on their physical health decide to stop drinking. Some people decide to stop for good. Other people decide to stop for a while (so that they bodies can start to recover) and decide to re-start drinking when they feel better. Obviously what you do is very much up to you to decide, but it is important that you realise that if you continue to drink in the same way that you have been, then it is very likely that these alcohol-related problems will get worse and become even more serious*<br>• *Whether you decide to stop for good, or to stop for a while and then re-start, we may also need to think together about what you will need to do about your friends who drink – will you continue to see them, and how will you avoid getting caught up in drinking? And about what you will need to do about going to places which remind you of drinking – will you try to avoid them, or are there other things that you can do, so that they do not make you want to drink heavily?*

Similarly, sometimes a patient may ask the counsellor to provide direction. The following example can help us to understand how to respond to such a situation using a collaborative stance.

Patient	*I am ready to do whatever you tell me to do but I want to get my drinking under control.*
Counsellor	*It's really good to know that you are putting so much trust in me and we will definitely work on the things which are known to be effective for drinking problems. However, rather than me simply telling you what to do, it will be much more beneficial for you if both of us work together on this. Is that ok?*

In this way, rather than simply giving directive advice we can help the patient to foster independence by working with him in a collaborative way.

3) Promoting independence by allowing the patient to make decisions rather than making them ourselves, while still guiding the patient and the session (navigating)

We promote independence in many ways – for example, we get the patient (and their SO if they are present) to

- Help develop the agenda for the session
- Decide on what things they want to work on in the sessions
- Decide on the overall drinking goal (stopping completely versus controlling the drinking)

There could be many other examples like this.

In each of these examples, and in other examples, we are not saying that as a counsellor, we have to simply follow whatever it is that the patient (or his SO) wants to talk about, or have to simply agree with his goals. As trained counsellors, we also have our own ideas over what will be helpful for this patient to work on. But we work in partnership, we collaborate, with the patient (and his SO if present) to ensure that both his views and our views are brought together. And, in almost all cases, if there is a disagreement over what is to be discussed or worked on, and if having given our view the patient still wishes to follow his own view, then we agree with the patient rather than insisting on our view.

An example – Setting an agenda for each session

To make effective use of the time available for each session, it is important that we all (the counsellor and the patient, and the SO if he or she is there) agree at the start of each session what we are going to discuss. We need to work together with our patient (and the SO) to set this agenda, rather than simply providing an agenda for the session our self.

On the other hand, we don't simply follow whatever it is that they say that they want to discuss. There is an agenda which we need to cover as part of the treatment. So the key idea here is that we (the counsellor, the patient and the SO) collaborate to decide on what needs to be discussed and, where needed, the counsellor *navigates* the agenda to ensure that key issues are covered.

Here is a list of questions which can help you to set an agenda together with the patient

<u>It is always useful to ask near the start of every session:</u>

- *What would you like to discuss today?*

<u>If the patient raises a number of possible areas, we can say:</u>

- *Which of these concerns would you like to discuss first?*

<u>But we can also add in our own thoughts:</u>

- In the first counselling session, we might say:
  'I wondered if it would be helpful for us to discuss the results of the screening test you took, which is why it was suggested that we should meet?'
- In the second counselling session, we might say:
  'Last week you told me that you did have some concerns about your drinking – you said that you were worried about your health, and about the arguments you had at home about it. Which of these concerns would you like to discuss first? (Or, *of all the reasons that you mentioned about you're drinking, which one do you want to discuss first?)'*
- Or we might say:
  'We finished our session last week by discussing a 'change plan' – can we put on the agenda a discussion about how that went?'

4) <u>Building and developing the patient's motivation for change</u>

Developing a patient's motivation for change is central to the treatment that we provide in CAP. Motivation is built using some of the skills discussed already - developing an engaged relationship with the patient, and working collaboratively with the patient. But, there are also some specific ideas and techniques, outlined below:

Most of the patients coming for treatment have '*ambivalent*' feeling about change [see *Appendix 1*]. By ambivalence we mean 'having strong feelings in opposing directions'. For example, a patient with harmful drinking may realise that his drinking has started creating problems in his life and he should change; and at the same time he may also have a strong desire to continue drinking. Helping a patient

to resolve such opposing feelings is a very important step in developing his '*motivation to change*' [see *Appendix 1*].
Patients who come to us for treatment will be at different stages of motivation to change.
Based on the level of motivation we can have three groups of patients and we should try and match our treatment to the level of motivation that the patient has.

Table 1: Different levels of motivation and area of focus for counselling at each level

**Level of motivation**	**Focus of counselling process**
Patient has never seriously thought about changing his drinking behaviour	We should focus more on helping the patient to understand the advantages and disadvantages of drinking, and see if we can help him make a decision on whether to change things
Patient has been thinking about making a positive change to his drinking behaviour, but at the same time has a strong feeling for continuing drinking	We should focus more on helping the patient to resolve the dilemma and to make up his mind to change in a positive direction
Patient has already decided to either control or completely stop his drinking	We should focus more on helping the patient to develop the change plan and work with him on implementation of the change plan, based on a sound understanding of why he has decided to change

However, whatever stage of motivation the patient is in, our key task is to:

- Help the patient to develop motivation for change
- Work with them to bring that change in reality and maintain that change

Two important ways that we can help build patients motivation to change are

- To encourage them to speak positively about the changes that they are or will be making (this is called '*change talk*' [see *Appendix 1*])
- To discourage them from speaking positively about staying as they are (when a patient talks against change, or speaks positively about their current harmful drinking, this talk is called '*sustain talk*' [see *Appendix 1*])

Important skills for a counsellor are to be able to:

- Identify 'change talk' within what a patient says, and to encourage it
- Elicit 'change talk' if the patient is not coming up with it spontaneously
- Identify 'sustain talk' within what a patient says, and work at discouraging it (or ignoring it)

Identifying and encouraging patients to speak positively about the changes that they are or will be making (changing talk)
It helps patients to change their behaviour if they hear themselves stating that they want to do this. If we can encourage the patient to make 'change statements' or to generate 'change talk', it is more likely that they will go on to change.
<u>How to identify and encourage change talk:</u>
Often a patient will spontaneously come up with change talk early in the course of our first session. Here is a list of ways in which change talk is often expressed by a patient during our counselling session:

- *I want to stop/control my drinking*, - this shows a desire to change (want, like, wish ….)
- *I am sure I can stop/control my drinking*, - this shows a belief in the ability to change (can, could ….)
- *I want to stop/control my drinking for sake of my children*, - this shows reasons to change (if I stop/change, then …..)
- *I need to change my drinking habit if I want to continue playing cricket*, - this shows a felt need to change (need, have to, got to ….)

It is an important skill for a counsellor to be able to identify such change talk and encourage it. When we encourage someone's '*change talk*' we are '*affirming*' them [see *Appendix 1*]. Some affirmations which we can use (based on the context) are:

- *Very good!*
- *Excellent!*
- *It's really nice to know that you want to stop / change your drinking!*

As we will see later in *Chapter 5*, *Chapter 6* and *Chapter 7,* there are things that we need to do in each phase of CAP. So, even though strong '*change talk*' statements can come from the patient in the early part of our counselling, it is not useful to jump directly to the *change plan* [see *Appendix 1*, and *Chapter 5* where the change plan is discussed] before performing a detailed assessment of the drinking problem and giving *personalised feedback* [see *Appendix 1* and *Chapter 5*]**.** However, we should note down all such '*change talk statements*' and continue with the process of assessment and giving personalised feedback. Then, once we have completed the assessment and have given personalised feedback, we need to come back to these '*change talk statements*' when we start working with the patient on deciding the goals. The example below shows how we might do this:

Patient	I have now decided in my mind to stop drinking completely [strong change talk from the patient].
Counsellor	It takes a lot of courage to stop your drinking and I am here to help you to achieve that goal [*strong affirmation from the counsellor*]. But do you mind if we talk a bit more about your drinking first, so that I can understand it a little better, as then I will be much more able to help you achieve the goal that you want to achieve

How to elicit change talk from the patient:

There are times when patients do not come up with '*change talk*' on their own. In such situations we need to try to elicit '*change talk*' from patient. There are several ways to do this. One is to ask them directly, via open-ended questions. For example:

'We've been discussing your drinking, but can you tell me, what do you like about drinking? What's positive about drinking for you? [And then, after the patient has told you a few things]: *And what's the other side? Are there any things about drinking that you don't like, or that seem to be negative?'*

This is an important technique, where we:

- First ask the patient to describe the good things about their drinking
- and then ask him to tell us about the less good things about continuing to drink in the way that he currently is doing
- Then getting the patient to compare these

This often helps the patient to clarify whether the advantages of changing outweigh the advantages of continuing to drink as he is currently doing. Other ways of phrasing these questions include:

- *Can you think of any disadvantages of changing your drinking?*
- *Can you think of any advantages of changing your drinking?*
- *What might be the negative consequences for you of changing or stopping drinking?*
- *What might be the negative consequences for you of continuing to drink as you do now?*

We might also draw their attention to the following hand-outs in the patient booklet we have given them: **Impact On Social And Family Life** (pg 4), **Health Effects** (pg 5) and **Why It Is Important That You Take Charge Of Your Drinking** (pg 7).

Other ways of eliciting '*change talk*' include:

- *You have told me while we have been discussing your drinking that your liver is being affected, and that your drinking is causing you some problems both at home and at work. Has that made you think about changing your drinking at all?*
- *Do you have any worries about drinking? What are they?*
- *Tell me what you've noticed about your drinking. How has it changed over time? What things have you noticed that concern you, which you think could be problems, or might become problems?*

- *What have other people told you about your drinking? What are other people worried about?* [If an SO is present, this can also be asked directly to her/him, after first asking the patient]
- *What makes you think that you need to make a change in your drinking?*
- *Is there anything about your drinking that you want to change?*
- *Is there anything that you have tried in the past, on your own, to change you're drinking?*

Of course, it is also useful to bring information from the screening and assessment into the conversation in this situation:

‘*One of the questions you answered for my colleague was that you sometimes needed an alcoholic drink in the mornings to get started. Thinking about that, does this make you feel that you want to change your drinking at all?’*

Some more techniques that can also be used for eliciting ‘change talk’ are:

- Looking forward (to a time in the future): how does the patient see the future, what would the patient like the future to look like?

  ‘*Suppose I ask you to imagine your future after one year, what you want your life to be like then?’*
- Looking back (to a time in the past when things were better):

  ‘*If we had the ability to go back in time and change certain things about your drinking, what are those things that you would like to change?’*
- You could ask the patient *"On a scale of 0 to 10, how ready are you to make a change right now in your drinking?"* with 10 indicating ready to change right now, and 0 not ready to consider a change. If the patient rates his readiness as 3, you can inquire, "*Why did you not say 0?*" which will prompt the patient to voice reasons for considering a change.

  Sometimes the patient might not understand what you mean by ‘a scale of 0 to 10’. An alternate way of putting it to the patient could be ‘*Out of 100 rupees, how many rupees worth are you ready to make a change right now in your drinking*?’ Indicate that 0 rupees is not ready at all and 100 rupees is fully ready.

Once this process is underway we can simply keep it going by using a few other skills:

- Elaborating - Asking for more detail
- Affirming – Commenting positively on the person’s statement
- Reflecting - Feeding back both the content and the feelings in what a patient has told us
- Summarising – Repeating the important points in the discussion

If we get bogged down and are not making much progress in eliciting ‘change talk’, we can review general areas, such as:

- Health – ‘*Are you aware of any health problems related to your drinking which we have not yet discussed?*’
- Financial – ‘*Has drinking contributed to any money problems?*’
- Relationships –‘*Has your drinking affected your relationships with anyone – your wife, your family, your friends, or your work relationships?*’
- Memory – ‘*Have you even not remembered the next day what happened while you were drinking? Or have you ever had any other memory problems related to you drinking?*’
- Legal – ‘*Have you ever been arrested, or had any other contacts with the police or the law because of your behaviour while drunk?*’

The nature of ‘*change talk*’ can include a variety of elements, from identifying concerns to expressing a desire to change. It includes

- The patient thinking that he has the ability to change
- A belief that life (or health, or social functioning or any other area affected by the drinking) will be better once change has taken place.

Eliciting ‘change talk’ forms the basis for building a commitment to change and making change plans – and it is the counsellor’s job to follow each occasion that ‘change talk’ occurs with reflection and reinforcement. This focuses the conversation in a constructive way.

All the ‘change talk’ statements made by a patient form the raw material for the discussion we will have with the patient where we will decide on the goals of the treatment and also for generating the ‘*change plan*’ [see *Appendix 1*] and then the specific ‘*action plan*’ [see *Appendix 1*]. We must therefore make a habit of writing down each ‘change talk’ statement. The process of collecting all

'change talk statements' is like making a bouquet of flowers by combining individual flowers. This collection of all these 'change talk statements' needs to be given back to the patient as a summary statement, while we work with him on deciding drinking goals or the change plan.

Identifying and discouraging patients from speaking positively about not changing (*sustain talk*)
A patient's statements which argue against any change in drinking behaviour are called '*sustain talk*'. The more those patients hear themselves say that they don't want to change, the less likely it is that they will change. So we need to discourage our patients from speaking positively about staying as they are, or from showing an interest in continuing to drink harmfully or problematically by carefully moving the discussion on.
Here are some common examples of 'sustain talk', showing the same ideas that lay behind the 'change talk' examples shown above: Desire, (In)Ability, Reasons, and Need

- *I don't want to change my drinking*, showing a desire not to change
- *I enjoy drinking so much that I just cannot give it up*, showing a belief in the inability to change
- *I have so many tensions at home/work. Unless all these tensions at home/work are taken care of, I cannot stop drinking*, showing felt reasons not to change
- *I need to drink at parties to keep my friends happy*, showing a felt need not to change.

'sustain talk' can also be expressed in different ways, such as interrupting, arguing, and denying a problem. Here are some examples:

Figure 3: Expressing 'Sustain Talk'

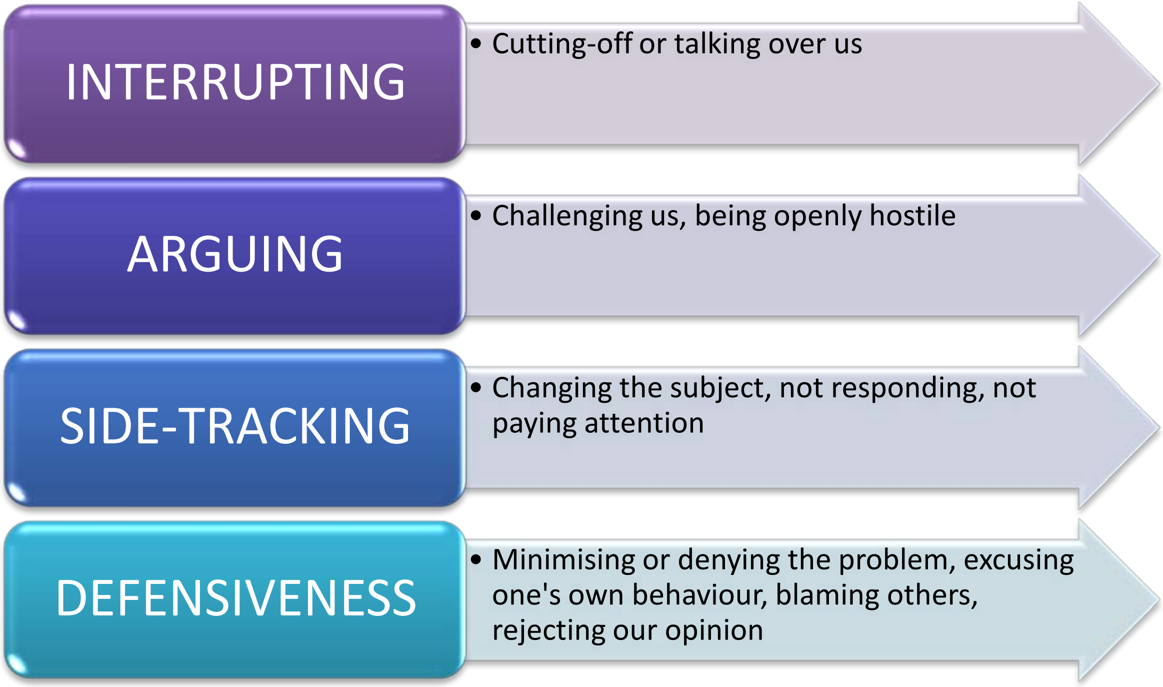

Key points

- The more often they hear themselves talk positively about changing [change talk], the more likely it is that they will change
- The more often patients hear themselves talk against change [sustain talk]**,** the more likely it is that they will not change
- It is our task in counselling to try to encourage 'change talk' and to discourage 'sustain talk'.

And there is a clear relationship between our style as a counsellor, and what the patient expresses.

- The more we work collaboratively with the patient, the less likely it is that patients will respond with 'sustain talk' and the more likely it is that they will use 'change talk'
- The bossier we are, the more likely it is that patients will respond with 'sustain talk'

There are certain things that we might do in counselling which seem to push patients into responding with more ‘sustain talk’ (called ‘*resistance*’) [see *Appendix 1*], and therefore we must avoid doing these things. These include:

- Assuming the role of expert or being bossy
  ‘As your counsellor I am telling you that you should stop drinking.’
- Criticising and blaming
  ‘Your drinking is a very irresponsible behaviour.’
- Provoking guilt and anxiety
  ‘You have a wife and two children to look after. Don't you feel your drinking is affecting your ability to look after them?’
- Telling the patient not to talk, just to listen to us as the counsellor
  ‘Don’t give me excuses. How many times do I need to tell you to stop drinking?’

So, an important goal in the treatment of drinking problems is to avoid evoking ‘*resistance*’. Remember that we want the patient to engage in ‘change talk’ (for example, "*I have a problem*" and "*I need to do something about it*").

However, when a patient uses ‘sustain talk’, it is important not to get into an argument with him, to convince him that he is wrong. Arguing with someone often makes them stick to their point of view and hold to what they are saying even more firmly. Instead, we try to deflect his ‘sustain talk’. Here are some strategies for deflecting ‘sustain talk’, all of which involve a recognition that something needs to change in the discussion.

- One strategy is simply to reflect what the patient is saying. This sometimes has the effect of eliciting the opposite and balancing the picture.

Patient	*I'm fed up with talking about my drinking!*
Counsellor	*Hmm... So you're fed up with talking about your drinking.*
Patient	*Yes that’s true … but you know, recently I have been thinking that I should give myself one last chance of sorting out this problem.*

- Another way is to exaggerate what the patient is saying to the point where the patient is likely to reject this view. (There is a subtle balance here, because overdoing an exaggeration can make the patient defensive or hostile.) An example is:

Patient	*But I'm not an alcoholic or anything like that.*
Counsellor	*You don't want to be labelled.*
Patient	*No. I don't think I have a drinking problem.*
Counsellor	*So as far as you're concerned then, there haven't really been any problems or harm caused by your drinking.*
Patient	*Well ….. I wouldn't say that.*
Counsellor	*I see. You don't like the idea of being called an alcoholic – or even saying that you have ‘a drinking problem’, yet you think your drinking maybe has caused – or at least contributed – to some problems?*

The counsellor statement in the example above shows how both sides of the patient's views are put together by the counsellor, which is another way to deal with sustain talk. If a patient offers sustain talk, reflect it back and add content from earlier in the session). Here is another example:

Patient	*But I can't stop drinking. All my friends do it!*
Counsellor	*It's difficult for you to imagine not drinking with your friends, and at the same time you're worried about how it's affecting you.*

- Another strategy is to defuse sustain talk by shifting attention away from the problematic issue, by changing the subject. (People sometimes call this 'rolling with' the sustained talk or 'rolling with the resistance'). This strategy can be particularly useful with patients who seem to reject every idea or suggestion.

Patient	*But I can't stop drinking. All my friends do it!*
Counsellor	*It sounds like this is not the right time to try to make a decision like this. Let's just carry on with what we're doing here - going through my feedback to you - and later on we can talk about what, if anything, you want to do about it.*

SUMMARY

- All the skills we learnt in the earlier module on general counselling skills are applicable to counselling patients with harmful or dependent drinking using CAP
- We always need to develop and maintain an engaged relationship with each patient
- Some key aspects of the counselling style that are particularly important in CAP are
  - ➢ Working collaboratively and respectfully with our patient
  - ➢ Nurturing independence while also guiding the process of our counselling
  - ➢ Not being afraid to have our own views about what might help a patient – but always expressing them collaboratively and respectfully and never imposing our views over the patient's view
  - ➢ Being non-judgemental
  - ➢ Expressing warmth and genuineness
  - ➢ Giving full attention and accurate empathy
  - ➢ Validating the patient's experience and encouraging his progress
  - ➢ Encouraging the patient to make positive (although realistic) statements about his progress or his hopes for change
- Particular emphasis is needed to ensure that we
  - ➢ Develop an *engaged* relationship with the patient (and the SO if they are present)
  - ➢ Work together (*collaborate*) with the patient (and the SO if they are present)
  - ➢ Promote *independence* by allowing the patient to make decisions rather than making them ourselves, while still guiding the patient and the session (*navigating*)
  - ➢ Build and develop the patient's Motivation to Change

Chapter 4

CAP Session-By-Session Guide

A. General Format of All the Sessions

Learning Objectives

In this chapter, we will learn:

- What the structure is of each of our sessions

CONTENTS

The key points discussed in the previous chapter about our ‘style’ of counselling need to underpin the structure of every session we have with our patients (and their SOs if they are present).
We always have to:

- Develop an engaged relationship with the patient (and the SO if they are present)
- Work together (collaborate) with the patient (and the SO if they are present)
- Promote independence by allowing the patient to make decisions rather than making them ourselves, whilst still guiding the patient and the session (navigating), and
- Build and develop the patient’s Motivation to Change

Plans
One of the ways that we work with our patients on CAP is to help them develop plans. This is so that both, our patient and us as the counsellor, know what our patient is aiming to achieve overall (what his main goals are – this is the *Change Plan*), and also what specific things he is aiming to achieve in the week or two between one session and another (this is the *Action Plan*).

- The Change Plan is the overall plan, outlining what the changes are that the patient wants to achieve in counselling (e.g. to completely stop drinking, or to learn how to control his drinking). Although we can revise and change this overall plan (and we check out in each session that the patient is still happy with the overall plan and the overall aims of our treatment), generally the plan does not change
- The Action Plan is the set of actions that the patient and the counsellor have agreed will be done in between one session and another. So the Action Plan often changes – one action plan might involve the patient practicing ‘drink refusal skills’ in between one session and the next, and another one might involve the patient practicing, dealing differently with tensions at home. Within each Action Plan there will be specific Homework Tasks, such as ‘fill out the drinking diary’, or ‘go to the college and get information about the course that we have discussed’, etc

We also mention later in this chapter something called the Target Behaviour.

- Target Behaviour is the thing (or set of things) that we are going to work on in the current counselling session. Typically, these include targets
  - related to drinking (e.g. not drinking at all over the past week, or drinking on only some days and to a pre-agreed level)
  - And those related to other things (e.g. not seeing friends who put pressure on the patient to drink; or dealing with conflict at work or at home in a different way, which does not involve drinking)

Of course, all three of these – overall Change Plan, Target Behaviours we work on together in the session, and the Action Plan describing what the patient has agreed to do differently in between the current session and the next one - relate to each other. But one is an overall plan (Change Plan), the second is some specific ways of behaving that we have agreed with the patient to work on in the present session (Target Behaviours), and third specifies some more detailed steps that the patient will be taking between one session and another (Action Plans).

THE STRUCTURE OF EACH SESSION
There are some things that we do in every session with a patient, and other things that we only do in phase 1 or phase 2 or phase 3.
This section below covers what we do in every session.

Each session may last from 30 minutes to an hour; and in each session, we aim to complete the five tasks below:

- Set an agenda
- (In each session after the first one) Review progress on the overall Change Plan [see *Appendix 1*] and on the specific Action Plan and any linked homework [see *Appendix 1*] that we set in each session. This will always include checking progress on our patient’s drinking

- Work on target behaviour [see *Appendix 1*]
- Plan Action Plan and specific homework to be done in between this session and the next one
- Summarise

We also always plan to tape record our counselling sessions. How we introduce this to our patients is described in the Counselling Relationship manual, in the section on 'Creating the Right Conditions for Getting Started'.

1. Set an agenda

What does it mean?

At the beginning of every session, we work together with the patient (and his SO, if present) to create a plan for the session. This plan or agenda includes a list of topics to discuss or tasks to complete in the session. It is valuable to take time to do this at the start of each session because it helps to ensure that the session focuses on what is most important for the patient and helps to provide a guide for us to use during the session to make sure that we are staying on track.

How is it done?

At the beginning of treatment, it may be helpful to orient the patient (and the SO if present) to the process of agenda-setting as the starting point for each session. Many patients will not be familiar with a structure that invites them to be active participants with their healthcare providers. It is often helpful to explain to patients (and their SOs) the reason described above for using agendas to guide sessions. It may be necessary to explain a few times the value of agenda setting and to reinforce for patients that we really want them to be active collaborators with us.

Because patients will be unfamiliar with this process of agenda setting, in the first session we may need to take the lead in setting the agenda (we describe how to do this in the chapter on the *Initial Phase*). In subsequent sessions, however, we first invite the patient to list agenda items, then we ask the SO (if present) to do so, to which we then add ours. Hence at the beginning of the second or other sessions, we can welcome the patient and ask simply and directly what he would like to focus on during that session. For example, the counsellor might say

'It is great to see you today, Paresh. Can we start by working out what we should discuss in our session together today? Are there certain things you want us to discuss or focus on?'

Once he has done so, if the SO is present we will ask the SO if they have any other things they would like to add.

As well as asking the patient and his SO, there will also be things that we want to put on the agenda - in most sessions, the counsellor also will have specific items to add to the agenda. Typical agenda items that the counsellor will contribute include:

- Checking how this week has gone
- Checking in about how the patient has been feeling
- Asking about drinking – whether or not they have drank anything, and if so, the details about this - how much, when, where, with whom, etc
- Asking about progress on the Action Plan and the homework e.g. was he able to fill in the drinking diary? Or in the past two weeks was he able to not drink when he was with friends, as agreed in the last session? Or was he able deal with any conflict between his wife and his mother without feeling that he needed to go out and drink?

In general, it is important to remember that agenda setting is:

- *Collaborative*, such that the session focuses on what matters most to the patient(and the SO)
- *Useful*, such that patients and counsellors have a guide for conducting the session and ensuring that they stay on track with the goals for the session
- *Flexible*, such that new topics or tasks can be added, but are best if done so in a clear and collaborative manner by directly discussing and deciding whether to modify the original agenda

2. Review progress on the overall change plan and on the specific action plan and linked homework that had been set in the previous session

What does it mean?

Checking progress on the Overall Change Plan and on the specific Action Plan and homework means

- Asking how much the patient's drinking and any problems associated with that drinking has changed since the last session
- Checking whether the patient has been able to follow through on the 'action plan' that we agree at the end of each session, which lays out what the patient is going to try to do differently prior to the next session, and specifies any specific homework tasks
- Checking that the overall Change Plan is still what the patient wants to do (for example, does he still wish to stop drinking completely, or does he now want to try to control his drinking)

Reviewing progress and the results of the action plan

- Gives us a report of the patient's current state
- Is useful to allow us to provide feedback to the patient (and his SO, if present) of how his condition is progressing
- And helps both us and the patient to identify the next steps for the patient to take

In addition to improving the patient's motivation to address his alcohol problem, CAP also aims to help people change the way they behave – what they do – so that they stop having problems related to their drinking. To stop having problems, they need to change their drinking – they need either to stop drinking, or to change the way that they drink. We help people plan to change what they do, and we then get them to practice doing things differently. We practice both in our sessions with them, and we get our patients to practice outside of the sessions by getting them to do homework.

So reviewing progress over what our patient has actually done since we saw him last is extremely important. Getting people to talk about their drinking and how to change it is important – but the most important thing is that they actually do change, not just talk about changing, so we need to check out with our patient in each session whether or not his plans for changing his drinking are working.

How is it done?

After having set the agenda, our first task in each session (other than the first session) is to clarify how things have been since our last meeting. So, after agreeing the agenda, our first question should be:

'*OK Mr Fernandes, we agreed that our first task in this session is to look at how things have been for you since we last met. So please, tell me, how has it been? …..*'

(If he gives us general feedback about his week but does not tell us about his drinking, we can (respectfully) stop him and say:

'*I'm sorry to interrupt, Mr Fernandes, but we talked last time about how you wanted to cut down on your drinking. Will you tell me – how has that gone?'*

[Note: we need detail here – we need to know how much he has drank each day or each week since we last saw him, and the circumstances of each occasion (or of some of the main ones if there are many) - when, where, with whom. If he simply tells us that "it has been fine" or "it has been better this week", we need to ask further questions to get a much clearer picture of what that means (e.g., "*Can you tell me a little more here – it has been* [for example] *14 days since we last met – on how many days did you drink any alcohol at all?*" and then for each of those days "*so on that day, how much did you drink – can you talk me through what happened (where you were, who you were with) and how did it come about that you drank when you had planned not to"* (or "*drank more then you had planned to*", if he is trying to control his drinking).

If it turns out that he has reduced his drinking, then we must praise him, and also ask further questions**:**

'*That is really great that you did manage to cut down / stop since we last met – well done! So tell me, how did you manage to do that? Did anyone help you to cut down / stop since we last met?*'

Also, we may have asked him to complete a 'Drinking Diary' for each day since we last saw him – and if we did, we can look at it together with our patient, to see what he has reported about his drinking each day/week.

We will also need to ask simply and directly about the patient's experience with the Action Plan agreed in the last session. For example, we might say,

'*We made a plan last time about what you were going to do differently - How did that go this week?*"

or

"*What was your experience with trying not to drink at the family party you were planning to go to last week*?'

The action plan might have contained specific homework (such as completing a drinking diary, or going somewhere to get some specific information to bring to the session). We need to also ask about the homework as well**:**

‘*We agreed last time that you would be completing a drinking diary – how did that go? Have you brought it here for us to look at together?*’

It is important to encourage any progress that the patient has made on the action plan or the homework, and to review what was done (or not done) in detail.

If the patient has not completed the action or the homework, it is important to ask directly and simply**,** “*what happened?*” or “*what got in the way?*”

We will first ask the patient, and we may then also ask the SO, if present (who may have a different perspective on why action was not taken or homework was not done). It is important to address this topic in a matter of fact and non-judgmental manner. Learning from what got in the way of completing the agreed action plan is critical in knowing how to help the patient take the next steps. Thus, although we as counsellors may feel awkward asking about things that patients didn’t complete, such discussions are essential. It is not possible to help the patient if we do not know what barriers he is facing. Remember that it is very common for patients to not complete actions and homework, and typical barriers will be discussed in more detail in subsequent sections.

3. Work on target behaviour

What does it mean?

Every session has at least one main target that is decided, on the basis of the patient’s treatment goals and the current status. Typically, these include targets related to drinking (e.g. not drinking at all over the past week, or drinking on only some days and to a pre-agreed level) and those related to other things (e.g. not seeing friends who put pressure on the patient to drink; or dealing with conflict at work or at home in a different way, which does not involve drinking).

How is it done?

The target behaviour is identified through a collaborative process of assessment that begins with the AUDIT score and the patient’s reaction to this. From this foundation, the counsellor and patient (and their SO) together decide on the priorities for a given session or week when setting the agenda.

4. Plan action and specific homework

What does it mean?

The main focus of CAP is to help the patient change his drinking behaviour (either to stop drinking altogether or to change the way that he drinks so that it is no longer harmful). The way that we know whether or not the patient is being successful depends on what he does outside of our sessions with him – so developing and planning an ‘action plan’ (which specifies what he is going to do in between our sessions) is a crucial part of each session.

So in each session, we and the patient (and their SO if present) need to work together to develop an action plan which will describe what the patient will try to do differently prior to the next session. It may also involve agreeing on specific homework tasks such as completing a form or obtaining specific information. The idea is that the patient follows the plan and does the homework, and then we review the patient’s experiences with using the action plan and doing the homework in each following session.

How is it done?

The counsellor and the patient (and the SO, if present) develop the action plan collaboratively. It is based on an assessment of the links between the patient’s drinking and the activities (such as attending family parties, or having a brother-in-law/friend actively encouraging the patient to drink) and problems the patient is facing currently (such as having a family argument when he returns home having drunk alcohol, or being warned at work about failing performance due to his drinking). The most effective action plans are specific, clear and concrete. In addition, it includes some planning for problems or barriers that may get in the way of the patient both following the action plan, and doing any specific homework tasks.

5. Summarise

What does it mean?

At the end of each session, it is always helpful to summarise a brief description of the session. It is always a good idea to first ask the patient to summarise what he has learned during the session and what actions he is committing to take prior to the next session. It is then good to ask the SO (if one is present) whether s/he has anything to add or change. We would then add in anything important that we feel has been left out, either from the summary of the session, or from the action plan. Sometimes, if the patient and the SO prefers, we can provide the summary without hearing from them, although this is not as useful.

However it is generated, the summary needs to include the key points that were discussed and the action plan and any specific homework for the coming week. A good summary is short but comprehensive so that the patient remembers what happened in a session. If the patient is literate, it is often a good idea to get the patient to write down some key parts, and to write down their action plan and any specific homework tasks. If they are literate enough to read but cannot write well, we should write down these key points for them.

How is it done?

The summary is provided verbally either by the patient or counsellor, and then often reinforced by getting the patient to write it down.

SUMMARY

Each session has the same basic structure:

- Set an agenda
- Review progress on the Action Plan and specific homework (in each session after the first one)
- Work on target behaviour
- Plan Action Plan and specific homework
- Summarise

Chapter 5

CAP Session-By-Session Guide

B. Initial Phase (Session One)

Learning Objectives

In this chapter, we will learn:

- How to introduce CAP to our patient
- Set the agenda
- Help our patient better understand his drinking based on initial AUDIT assessment
- Give personalised feedback to our patient about his drinking
- Help our patient to decide on his drinking and other goals
- Working with our patient on developing a change and action plan

CONTENT

INTRODUCE CAP TO OUR PATIENT

The first thing that we do after we have introduced you to the patient and his SO if they are there is to introduce CAP to the patient.

Counsellor	*Hello Mr Naik – it is very good to meet you today, and thank you for coming. My name is XXXX. As I think you know, your doctor has suggested we meet to discuss your answers to the questions you were asked about your drinking earlier today.*<br><br>*Can I try and explain you how we are going to work together on this?*
Patient	*Ok*
Counsellor	*Thanks. What I'd like to do is to work together with you (and with your wife/brother/sister/mother/friend etc, if you are happy for us to include her/him in this discussion) to clarify what the issues are around your drinking, then we can all work together to decide what you want to do about these issues, and then I help you to make any changes that you want to make.*<br>*There are three parts to the work that we do here.*<br>• *In the first part, we discuss your drinking and whether it has given rise to any problems, and we decide together on what you want to do about it. We will certainly do this in time that we spend together today – about 40-60 minutes.*<br>• *In the second part, we plan out together how you are going to make any changes that you want to. We'll probably get into this today, but, if you want, we will probably meet up again for a second session, after a gap of about seven to 15 days. That will give you time to try out any change that we discuss today. Then in that second session we'll see how things have gone and work more on helping you to achieve whatever goals it is that you may set today*<br>• *In the third part, we work out how to make those changes long-lasting. We may also do some of that in our second session. We will probably stop our meetings at that point, but if you want, you can contact me within the following two-and-a-half months to ask for one or two more sessions, which again would be focused on helping you to achieve and maintain the treatment goals which you and I will set together today*<br>*So, just to summarise, we will meet a maximum number of four times, although we may get these three parts competed faster – with some people it only takes one or two meetings, with others, three, or all four that we have available.*

(At this point, you can open the patient booklet to the hand out '**Treatment For Drinking Problems**' on pg 3 and explain to the patient that brief details about the treatment are provided in it.)

As you will have seen from the discussions in the previous chapters, at this stage we try to involve the patient's Significant Other, in the way that we outlined earlier (see the section on 'Engaging a patient's SO' and the example on it in *Chapter 3*). If he has come to the PHC clinic with an SO, and if he is OK with that person being included, we will invite the SO to join us at this point for the rest of this (and subsequent) session(s). If there is no SO there, we raise the idea of the patient bringing one with him to the next session.

(You can now open the patient booklet to the hand out '**Getting Help From Someone Close To You**' on pg 3 and explain to the patient how an SO can help in the treatment.)

As discussed in the Counselling Relationship manual, it is also important to discuss with the patient about the confidentiality of the information that he shares with us and the ways by which he can contact us outside the counselling sessions for any emergency.

SETTING THE AGENDA FOR THE SESSION
Once we have introduced CAP to the patient (and his SO if present) the next step is to set an agenda for the session, as discussed in *Chapter 4*. We need to explain to the patient and his SO that we set an agenda (have a list of what it is that we want to cover in the session) as the starting point for each session. We explain that in future sessions we will expect that the patient and his SO will tell us first what they want to work on; but that as this is the first session, we will provide a list to start with. We might say:

Counsellor	*To make most out of the 40-50 minutes we have today, it will be very helpful if we plan how we are going to spend that time. This will ensure that we focus on what is most important for you and will provide a guide for us to use during the session, to make sure that we are staying on track. Normally I'll expect you to come to the session telling me what you want us to talk about, but as this is the first session, can I start? You can then tell me your thoughts about this outline. Then we can start the session based on the things that we have agreement on. Is that fine with you?*
Patient	*Ok*
Counsellor	*Thanks. As you know, my colleague asked you some questions about you're drinking of alcohol when you came to the PHC today The answers you gave made the doctor who was seeing you think that it might be helpful for you to talk to someone about your drinking So, if you are happy with this, what we are going to do today is look at the answers you gave to those questions, and then I may ask some more questions, (and ask some questions as well to your wife/brother/sister/mother/friend etc., if you are happy for us to include her/him in this discussion?), so that I can get a better understanding about your drinking.*<br><br>*At the end of that, I'll share with you what I have understood about your drinking. Then, if you want, we can decide together whether you want to work with me to decide if you want to change anything about your drinking.*<br><br>*If you decide that you do want to work with me on this, then I'll tell you more at that time about what that would involve. Is that OK?*<br><br>*How does this list sound to (both of) you? Is there anything else (either of) you would like to discuss?*

Based on the consensus we develop with the patient we can start the session with the process of helping the patient in better understanding his drinking based on his AUDIT scores.

HELPING OUR PATIENT TO BETTER UNDERSTAND HIS DRINKING (based on the initial AUDIT assessment)
Assuming that the patient has agreed with our agenda, we then go through the AUDIT results, and for each question answered showing a potential problem (e.g. anything scoring 2 or more), we check out that the patient is still happy with the answers that he gave, and then ask further assessment questions so that we can feel surer that we understand the patient's drinking.
This is a very important part of counselling. Going through the AUDIT and assessing someone's drinking is not something that is isolated from the counselling process: we want this part of the process to be a motivating experience for the patient. Also, information gathered during this process, when related back to the patient ('Personalised Feedback') forms an important part of the motivational process.
The AUDIT will be completed by a Health Assistant before the patient comes to see us. So, when we see the patient, we will already have information about the following:

1. How frequently the patient drinks alcohol
2. The typical quantity consumed by the patient
3. How frequently the patient drinks heavily
4. Whether the patient is able to control his drinking

5. Whether alcohol plays an increasingly central role in the life of the patient relative to other responsibilities
6. Whether the patient needs to take a drink first thing in the morning to get going
7. Whether the patient feels guilty after drinking
8. Whether he has no recollection of events after a session of heavy drinking
9. Whether the patient or anyone else has suffered injuries as a consequence of the patient's drinking
10. Whether other people have expressed concern about his drinking

When we check with the patient the answers suggesting harmful drinking (e.g. anything scoring 2 or more), we will often need to ask more detailed follow-up questions. For example, question 10 asks:

'*Has a relative or friend or a doctor or another health worker been concerned about your drinking or suggested you cut down?*'

Let's assume that the patient told the screener that the last time he had come to the PHC, his doctor had suggested this. We might want to feed this back and ask more about this:

Counsellor	*From what I can see, when you answered those questions put by my colleague earlier today, you mentioned that your doctor has been concerned about your drinking. Can you tell me a little more about that?*
Patient	*I was not feeling very well last week and so I had come to see the doctor.*
Counsellor	*What happened?*
Patient	*I vomited a few times in the night and I was feeling very weak in the morning.*
Counsellor	*Hmm. I can imagine you must have been worried.*
Patient	*Yes. This was the second time this had happened to me in the past month.*
Counsellor	*Did he say how that might have happened?*
Patient	*He told me that it is because of my drinking and that my liver is damaged.*
Counsellor	*What do you think?*
Patient	*I am not sure. But I have been drinking quite a lot in the past couple of months.*

We need to go through each question that the patient answered positively to, and clarify the answers to each of them in the same way.

We also need to remember that the AUDIT does not give a detailed history about the patient's drinking. Just because the AUDIT has already been completed should not stop us from asking more detailed follow-up questions, either about issues already covered in the AUDIT (see the example above about someone suggesting that the patient cuts down his drinking) or about other important issues. Some of these other important questions that you might want to ask include:

Current drinking pattern

- *What do you normally drink?*
- *Where do you normally drink?*
- *Do you drink by yourself or do you drink with others?*
- *How often do you drink to the point of getting drunk?*

History, and past change attempts

- *At what age did you start drinking?*
- *Have you ever tried to give up drinking? What happened?*
- *What is the longest period you've gone without drinking?*

Clarifying problems

- *Has drinking ever caused you problems of any kind? E.g. problems with the police, financial problems, relationship problems, work problems, health problems, etc.*

This is by no means an exhaustive list and the kind of questions that you ask will depend on the information that the patient provides during the session. However, whatever the question, it is very important throughout the assessment process to use the skills mentioned under the section on effective

counselling skills in the Counselling Relationship manual and techniques to increase motivation described in this manual. Carrying out an assessment as if completing a check-list is very likely to negatively affect the counselling relationship with the patient.
Here is a brief example of an assessment done using counselling skills (recommended). In this example, the patient agreed that his wife should join the session.

Counsellor	*What do you normally drink?*
Patient	*Vodka*
Counsellor	*Are there times when you drink anything besides vodka?*
Patient	*On weekends I also have a couple of beers in the afternoon with my lunch.*
Counsellor	*Hmmm. That sounds as if you drink more on weekends than on other days of the week. Is that correct?*
Patient	*Yes, I suppose so.*
Wife	*Yes – he does drink quite a lot more at the weekends.*
Counsellor	*OK - Where do you normally drink?*
Patient	*In a bar...Sometimes at home.*
Counsellor	*Do you drink mostly at home or mostly at a bar, or is it about equal?*
Patient	*Mostly at the bar.*
Counsellor	*Is there anything that stops you from drinking more frequently at home?*
Patient	*My wife does not like it when I drink at home.*
Counsellor	*You mean to say that your wife disapproves of your drinking?*
Patient	*She is constantly telling me to stop my drinking.*
Counsellor (to wife)	*Is that the case?*
Wife	*Yes, none of us at home like him drinking so much.*

(We could continue this conversation with the wife at this point, and ask her why they don't like him drinking, as this will tell us a lot about how he behaves when he is drinking, but this is not likely to help him feel comfortable discussing his situation. Instead, we will continue with asking more from the patient, and note this down in our records to ensure that we remember it, so that we can come back to this later, when we are trying to get the patient to tell us what his reasons are for changing his drinking behaviour).

Counsellor	*Hmm. Do you drink by yourself or do you drink with others?*
Patient	*I only drink when I am with friends.*
Counsellor	*Why is that?*
Patient	*Well, all my friends drink. When I am with them they ask me to give them company by having at least one drink. Once I have had the first drink I find it difficult to stop. If I am by myself I usually take my mind off drinking by watching TV.*
Counsellor	*So are you saying that there is something about being with your friends that leads you to drink more? You say that when they ask you to join them for 'at least one drink' you do so – why is that?*<br>*(Depending on what the patient says, we could follow this up with further questions:*<br>• *Do you feel 'pressured' by your friends to drink? Or do you just not like to say 'no' to them?*<br>• *Or is it something else – given that you know that if you have one drink it is difficult to stop, why do you take that first drink?)*

And here is a brief example of how not to do an assessment, in which we ask a series of questions as if we are simply ticking boxes on a questionnaire.

Counsellor	*What do you normally drink?*
Patient	*Vodka*

Counsellor	*Where do you normally drink?*
Patient	*In a bar……….Sometimes at home.*
Counsellor	*Do you drink by yourself or do you drink with others?*
Patient	*I only drink when I am with friends.*
Counsellor	*Ok*

As you can see, in the earlier example, when the counsellor uses techniques like reflection, and asks for more details, he is able to get more useful information as compared to the second example.

As with everything else that we do in CAP, if the patient does not understand the logic behind what we are doing, he will not be able to cooperate fully and indeed might not benefit much from what we do. Many times patients might not understand why you are asking questions similar to what he was already asked during screening and might challenge us about it. If this happens we need to explain to him that the screening involved a few questions to identify who should be offered counselling and the detailed questions that you are asking will help us to get a better understanding of his drinking and thus allow us to help him.

All of this could be avoided if we start off the detailed assessment by explaining to the patient what we are trying to achieve. You could start the assessment by saying the following:

'Mr Raikar, my colleague outside asked you a few questions about you're drinking. That helped us decide to whom we should offer this counselling treatment. The questions that I am asking you now are more detailed and will help us to get a better understanding about your drinking and will allow us to help you better. Shall we do that?'

If you don't do that you often get into a situation where the patient challenges you. If that happens, the following script will demonstrate to you, how this could be done

Counsellor	*What do you normally drink?*
Patient	*Vodka*
Counsellor	*Are there times when you drink anything besides vodka*
Patient	*On weekends I also have a couple of beers in the afternoon with my lunch.*
Counsellor	*Hmmm. That sounds as if you drink more on weekends than on other days of the week. Is that correct?*
Patient	*Yes, I suppose so. But why are you asking me all these questions? Your friend outside already asked me all these questions.*
Counsellor	*Mr Khan, please let me try to explain to you what we are trying to do. My colleague outside asked you a few questions about you're drinking. That helped us to decide whom we should offer this counselling treatment. The questions that I am asking you now are more detailed and will help us to get a better understanding about your drinking and will allow us to help you better.*

<u>Note:</u> The treatment that we provide our patient will depend on the type of drinking problem that he has i.e. hazardous, harmful or dependent drinking. One way of deciding whether the patient has hazardous, harmful or dependent drinking is by looking at his AUDIT score. However, the AUDIT is not a perfect tool and can sometimes get it wrong. For example, the AUDIT might indicate that a patient is a harmful drinker but when you do a detailed assessment you realise that the patient is a dependent drinker, or vice versa. When this happens, you have to base your decision about what type of treatment the patient gets, on your assessment, and not the AUDIT. Furthermore, in such situations, the personalised feedback too should be based on your assessment and not the AUDIT score.

Tobacco use

A large proportion of patients having drinking problems also have problems with tobacco use**.** Though the primary focus of CAP is drinking problems, once we finish our assessment of alcohol we should ask our patient about the use of tobacco**.** If our patient tells us about his use of tobacco in any form then we should tell him that many of the skills we will learn in CAP will also be useful for tackling his tobacco problem, and if he wants to then we can specifically work on dealing with the tobacco

problem once we have completed the intervention for drinking problems. The details of performing the assessment of tobacco problems and how to help such a patient are mentioned in *Chapter 10***.**

Domestic violence
An important consequence of drinking that we must explore is domestic violence. Domestic violence is a pattern of abusive behaviours by one partner against another in an intimate relationship. This could take the form of actual or threats of physical aggression or assault (hitting, kicking, shoving, slapping, throwing objects), sexual abuse or emotional abuse (rejecting, ignoring, isolating, intimidating, neglecting). Please refer to the CR manual for details about assessing and helping to manage domestic violence.

Summarising our patient's drinking
At the end of this process of clarifying our patient's answers on the AUDIT, we need to sum up with the patient what we understand about his drinking, what his AUDIT score is, and what it means (i.e. that his drinking is harmful). We do this in the next section, on providing Personalised Feedback.

Using a drinking diary
Sometimes when we gather information about a patient's drinking, we find that he is not able to tell us in any detail about what he has been drinking – how much, when, where, and with whom. In those cases, and also when we are helping a patient to control his drinking (which we will discuss below), we often ask as a specific homework task that the patient completes a '*drinking diary*' [see *Appendix 1*]. Blank drinking diary forms are included on pg 19 in the patient booklet. Once the patient brings this completed form back, we will use the list we have of the quantity of alcohol contained in different drinks to calculate how much he is drinking.
The drinking diary task is an excellent way of helping both us and the patient to gain a fuller understanding of the quantity, frequency, and other aspects of his drinking. Getting a patient to monitor his or her own behaviour serves a number of other functions as well, especially raising his awareness about how the places he visits and the people he sees have an effect on his drinking.

The specific role of the Significant Other (SO) in helping a patient gain a better understanding of his drinking. If the patient has brought an SO with him and has agreed that this person should be present, we will need to also involve the SO in this process of assessment. The SO might be able to fill in gaps in the information which the patient is not able to provide for a range of reasons.

Note - If the patient suggests that we want to ask questions of his SO because we don't trust him, we need to emphasise that this isn't the case – instead, it is because if we involve an SO, the patient will have much more support in their lives outside of our few counselling sessions to enable them to make any changes that they want to. It is also the case that involving his SO may allow us to gather more comprehensive information, to help the patient.
An example of how the SO can be helpful during a session is as follows:

Counsellor	*So, how long have you been drinking?*
Patient	*Probably eight years now but I am not really sure. I tend to forget things quite a lot these days.*
Counsellor	*Could we confirm with your wife here?*
Patient	*Of course*
Wife	*I think it is probably 10 years. He started drinking a couple of years after our marriage, which was 12 years ago.*
Counsellor	*OK. Do you think that is about right?*
Patient	*Could be.*

Another example is as follows:

Counsellor	*Do you think alcohol affects your life in any way?*

Patient	*Not really. I have my drinks in the evening, come home and quietly go to bed.*
Wife	*That is not really true. There are times when you come home and get into arguments with your brother.*
Counsellor	*Is that true?*
Patient	*Well…….. That does happen. But rarely.*

Involving the SO in helping the patient gain a better understanding can have many advantages. The patient can get to hear more about how his drinking is affecting other people, which may increase his motivation to change; there may be things that the patient cannot recall that his SO can, which will lead to a more comprehensive picture being gained; the SO may learn more about the impact of drinking on the patient's life and may then be more motivated to help the patient change.

IMPORTANT

How to proceed from here will depend on the type of drinking problem that the patient has. This is usually determined by the AUDIT score, but if there is a discrepancy between the AUDIT score and your assessment, then your assessment should decide how we proceed from here.

Hazardous drinker: The AUDIT will usually identify almost all people with hazardous drinking. They will be dealt with by the HA, and we will not have to see them for counselling. However, as the AUDIT is not perfect, there will be some times when a hazardous drinker will be categorized as a harmful drinker or very rarely as a dependent drinker. In such cases, if we do our assessment properly we will be able to see that they are not a harmful or dependent drinker, but actually are a hazardous drinker. For such patients, we need to provide personalised feedback, hand them the leaflet for hazardous drinkers and discharge them from treatment.

Harmful drinker: Complete the rest of the session as described here. Such patients should get up to four sessions of CAP, including this one.

Dependent drinker: Complete the rest of the session as described here. Such patients should get up to four sessions of CAP, including this one. **In addition, at the end of our session with this dependent drinker, we must take the patient to the PHC doctor and request him/her to refer this patient to a psychiatrist for detoxification.**

Harmful or Dependent drinker who is currently abstinent: The AUDIT covers a period of one year. This means that anyone who has had drinking problems in the past year will be detected as such, even if he currently does not have drinking problems e.g. If the patient was drinking harmfully and has now stopped drinking for more than a month, he will still be classified as a harmful drinker according to the AUDIT. In such situations, it is important to explain this to the patient and tell him that CAP will still be helpful for him, to help him maintain his goal of reduced drinking or abstinence. We could say to the patient,

'Mr Fernandes, you have told me that you used to have a drinking problem / problems with your drinking until about a month ago. It is great that you have been able to stop by yourself and that you have not had a drink for the past one month. But it is important to remember that often, remaining absent is equally or more difficult than stopping drinking in the first place. In counselling we can work to identify situations which could put you at risk of going back to your old drinking patterns, and together we can devise strategies to counter such risks'

With such patients, after assessing the patient's level of motivation, we might be able to fast-track the patient to Phase 3, after completing Phase 1 in session 1.

GIVING OUR PATIENT PERSONALISED FEEDBACK ABOUT HIS DRINKING

As a result of the assessment above, we now have a better understanding of the patient's drinking. We can now give the patient (and his SO) personalised feedback about his drinking (that is, we let him know how and why his drinking may be harmful, and how it may be related to any problems or issues that he may have told us about, during the assessment). Providing personalised feedback to the patient (and his SO) is important as it is more likely to trigger a stronger commitment to change in the patient.

We have to first explain to the patient (and his SO) that we would like to feedback to him our interpretation of the information that he has given us.

'So, can I sum up here? My colleague asked you some questions when you came to the PHC today, and we have just gone over them again and had a detailed discussion about your drinking and its impact on your life. I would like to share my thoughts with (both of) you about this. This might then help you to make decisions about your drinking. Do you think it would be helpful to do that? You can then give me your opinion about what I'm about to say to you'

The information that you need to feed back during personalised feedback includes the following.

- AUDIT score, and what it means. Details about this are provided in **Appendix 5**.
- The extent and patterns of the patient's drinking.
- The various problems due to drinking that the patient has reported, and to relate that to a summary of the possible ill effects that have been reported in harmful drinkers in Goa.

Any information about the effects of drinking should be given to the patient only if it applies to him. E.g. If the patient has told you that he has lost many jobs then you can tell him how that is commonly seen in people who drink heavily.

Some of the patients we see will have an AUDIT score between 12 and 19 and will be likely to be **Harmful Drinkers**. Others will have an AUDIT score of 20 or more (and will probably have told you about various symptoms of dependency such as getting withdrawal symptoms if they stop drinking), and will be likely to be **Dependent Drinkers**. Examples of personalised feedback for both types of drinker are shown below.

An example of personalised feedback for a harmful drinker would be as follows:

'I would like to summarise some of the things that we discussed today. This would give us an opportunity to review what you've told me and make any changes or additions. As we discuss this we could think about working together in the future to develop ways that you could use to change your drinking.

You reported that your favoured drink is whisky and that you first started drinking about eight years ago (and your wife suggested that it might have been 10 years ago). You told me that you are presently drinking one quarter of whisky four or five days a week. Your AUDIT score is 16 which put you in the category of harmful drinking. This means you are drinking more heavily than most men in Goa. This also means that you drink at a level where it is causing harm to your physical and/or mental health. .

You told me that you have continued to drink even though it has been causing problems at home and at work. Both you and your wife have told me that your drinking has led to fights and to you getting into trouble with other people. Your doctor has now also told you that your liver is damaged because of your drinking. So you, your wife and your doctor have all said that drinking is causing you problems - with your health, and at home, and at work. I think that it will be useful for you to also know that men who drink as heavily as you do are also more likely to get psychological problems (such as depression or tension), as well as many more health and other problems of the sort that you are already getting. They are also more likely to go on to become dependent on alcohol, and to develop even more serious problems.'

An example of personalised feedback for a dependent drinker would be as follows:

'I would like to summarise some of the things that we discussed today. This would give us an opportunity to review what you've told me and make any changes or additions. As we discuss this we could think about working together in the future to develop ways that you could use to change your drinking.

You reported that your favoured drink is feni and that you first started drinking about 15 years ago. You told me that you are presently drinking half a bottle of feni every day. Your AUDIT score is 23 which puts you in the category of dependent drinking. This means that your body has become used to alcohol and you have developed symptoms like vomiting, shaking of the hands, headache etc. (this would depend on what symptoms the patient has reported) *if you don't have a drink.*
You seem to be drinking much more heavily than most men in Goa. You told me that you have continued to drink even though it has been causing problems at home and at work. Both you and your wife have told me that your drinking has led to fights and to you getting into trouble with other people. Your doctor has now also told you that your liver is damaged because of your drinking. So you, your wife and your doctor have all said that drinking is causing you problems - with your health, and at home, and at work. I think that it will be useful for you to also know that men from Goa who drink as heavily as you do are also more likely to get psychological problems (such as depression or tension), as well as many more health and other problems of the sort that you are already getting'

(At this point you can turn to the hand out '**Types Of Drinking Problems**' (pg 4) of the patient booklet. You can then explain the three types of drinking problems to the patient. You can also use the hand-outs '**Impact On Social And Family life**' on pg 4 and '**Health Effects**' (pg 5) at this point. Finally, you must use the hand out '**Understanding Your Drinking**' (pg 6) in which you must fill in the amount of alcohol the patient drinks every day, AUDIT score, tick the box adjacent to the drinking problem that applies to the particular patient and the amount of money the patient spends on alcohol every month.)

After you have given personalised feedback you can ask him for his overall response to the feedback.
'I've given you quite a bit of information here. Have I got everything right, do you think?'
(Followed by) '*I wonder what you make of all this.'*
Both the feedback and the follow-up query will sometimes directly elicit 'change talk' that can be reflected back to the patient. (We discussed 'Change Talk' in some detail in *Chapter 3*, under the section on 'Building and developing the patient's motivation for change'.)

The specific role of the SO in receiving personalized feedback
Providing feedback to the patient and his SO is important as it is more likely to trigger a stronger commitment to change in the patient. If the patient knows that his SO has also heard the personalised feedback and therefore understands that he has a drinking problem, he is not able to minimise to the SO the need to change.

HELPING OUR PATIENT (AND HIS SO) DECIDE ON HIS DRINKING AND OTHER GOALS

After performing a detailed assessment of patient's drinking problem and giving him personalised feedback about his drinking, the next important step in CAP is to work with the patient on defining the goals of treatment.

What if our patient does not think he has a drinking problem?
When we provide our personalised feedback to the patient (and his SO if present), and ask for the patient's reaction, we will quickly find out whether the idea that his drinking is at all problematic comes as a surprise to the patient or not, and whether he is agreeable to think about changing.
For most of the patients we see, it will not be a new idea that their drinking may be starting to be related to problems. Many patients will have told us in the assessment about health concerns they have which might be drink-related, or about other potential alcohol-related problem areas (such as arguments at home or with friends, accidents, problems at work, etc.).
Also of course, these patients will all have seen their doctor after the screening, who will (hopefully) also have told them that their drinking is related to their problems; and the Health Assistant will have taken consent for treatment, which again specifies that the treatment is for alcohol-related problems. So the very large majority of patients will be accepting of the idea that their drinking is related to various problems and that there is at least a conversation to be had about whether they want to change, and if so, how.

However, there may be a few patients who will not want to accept the personalised feedback we have provided, and will wish to argue that their drinking is not causing any problems.
In *Chapter 3* there is a section titled ‘Building and Developing the Patient’s Motivation for Change’ in which we discussed different levels of motivations, the area of focus for counselling at each level of motivation, and how to build and develop motivation throughout the three phases of CAP**.** It is worth reproducing the Table we had there, looking at different levels of motivation and what we should then do.

Table 2: Levels of motivation and the focus of the counselling process

**Level of motivation**	**Focus of counselling process**
Patient has never thought about changing his drinking behaviour	We should focus more on helping the patient to understand the advantages and disadvantages of drinking, and see if we can help him realise that he might want to start to change things
Patient has started thinking about making a positive change to his drinking behaviour, but at the same time has a strong feeling for continuing drinking	We should focus more on helping the patient to resolve the confusion and to make up his mind to change in a positive direction
Patient has already decided to either control or completely stop his drinking	We should focus more on helping the patient to develop the change plan and work with him on implementation of the change plan

The patient who does not believe that he has a drinking problem is similar to the patient who has never thought about changing his drinking behaviour. As you can see, for patients such as these, we should not enter into an argument with him, nor try to convince him that he really does have an alcohol problem. Instead, we need to focus more on working with him to look at the advantages and disadvantages of drinking, and see if we can help him realise that he might want to start to change things.

Counsellor	*I've given you quite a bit of information here. Have I got everything right, do you think?*<br>(Followed by) *I wonder what you make of all this.*
Mr Naik	*I don’t really understand what you have said. Yes, I know I drink sometimes, but I don’t have any problems because of it. I have come here today because I have a stomach upset – it is nothing to do with my drinking! I drink the same as many of my friends, and they do not have problems!*
Counsellor	*OK – you drink the same as many of your friends, and they do not have problems.*
Mr Naik	*Yes! They don’t have any problems, and neither do I!*
Counsellor	*I’m not trying to persuade you that you have an alcohol problem. I just want to get a better understanding of the impact that alcohol has on your life. Could we talk about that a little more?*
Mr Naik	*Why? There really is nothing to discuss about my drinking!*
Counsellor	*If your wife (or a good friend who doesn’t drink) was here, what would she/he say?*
Mr Naik	*Well sometimes my wife and I argue about it. My wife is always telling me that I drink too much! That is why we argue – and that is why I sometimes go out drinking.*
Counsellor	*Hmm… It sounds to me as if you are saying that the issue of you drinking does lead to some problems at home. Why do you think that your wife is telling you that she thinks that you are drinking too much?*

This session would then continue which the counsellor helping Mr Naik see that there might be an issue here which is worth more discussion. This could lead to the counsellor inviting Mr Naik to bring

Mrs Naik with him to his next session; and/or to a discussion of the pros and cons of his drinking, given that it seems to be both a strongly positive activity to him, but also one related to both arguments and health problems (stomach complaints). This is discussed further in the next section, on ‘working with patients who are ambivalent’.
Of course, the session above might not move into a discussion of ambivalence. Mr Naik could continue to be very resistant to accepting that he has any problems, or indeed, he might not have any significant problems that he is concerned enough about to want to change:

Counsellor	*So as far as you are concerned then, there haven't really been any problems or harm caused by your drinking.*
Mr Naik	*No!*
Counsellor	*OK – can I change the subject a little here? Can I ask you – do you ever, or have you ever had, any concerns at all about your drinking? Have you ever wondered, even a bit, whether you might be drinking too much, even on some occasions?*
Mr Naik	*Yes, of course I sometimes drink too much, but everybody does that, sometimes! But I don't think I have a problem with alcohol!*
Counsellor	*OK – So, on the one hand, you really don't want to be labelled as having a ‘problem with alcohol’ – but on the other hand, you can see that sometimes you feel that you do drink too much? Has anyone else ever suggested to you that maybe you drink too much?*
Mr Naik	*My wife is always telling me that I drink too much! That is why we argue – and that is why I sometimes go out drinking. You see, the problems are not caused by my drinking – my drinking is caused by my wife nagging me!*
Counsellor	*And that really annoys you?*
Mr Naik	*Yes of course it annoys me. Wouldn’t it annoy you if your wife was always nagging you!*
Counsellor	*So what do you think you can do about this, Mr Naik? I can hear from you that these arguments and nagging are annoying and upsetting. Do you think that there is anything you could do which might help the situation?*

Again, this session would then continue which the counsellor helping Mr Naik see that there might be things that he could do which might diffuse these tensions at home, and that one thing might be to sometimes not go out drinking. Again this could lead to the counsellor inviting Mr Naik to bring Mrs Naik with him to his next session, and/or to the discussion of the pros and cons of his drinking, as outlined above.
(When discussing the pros of not drinking, you can use the hand out ‘**Why It Is Important That You Take Charge Of Your Drinking**’ on pg 7 and get the patient to tick the relevant boxes if he has named any of the pros of not drinking listed in the hand out.)
However, sometimes, even with gentle but repeated questioning like this example above, the patient still does not want to discuss their drinking. We might say:

‘*I get the feeling that really you are quite happy with your drinking as it is?’*

Or

‘*Well, I can certainly hear that drinking is really important to you – even though your wife is asking you not to go drinking, the drinking sounds so important that it is worth having all of the arguments and the nagging, just to be able to have those drinks?’*

Or even

‘*I am not sure that you are really looking for any help to change your drinking at the moment.’*

If after all of this, the patient is still adamant that he does not have any issues related to his drinking that he wants to change, it is best to close the session down, while still leaving the door open for a future appointment:

‘*OK Mr Naik. From our discussion today, what I hear is that you do not think that your drinking is causing any problems. I respect your opinion and don’t want you to think that I want to*

force you into accepting my point of view. I can certainly hear that you don't want at the moment to discuss whether or not there is a relationship between your drinking and some of the problems that you have told me about. That is fine. I just want you to know that, if you have a think about what we have discussed today and want to come back to talk with me about it in the future, you can. Here is my telephone number here at the PHC, or you can just come in and ask to make an appointment with me, and I will fit you in.'

We might also ask this patient if he might consider filling out a drinking diary form on pg 19 of the patient booklet over the next week (also described above). We might suggest that this could enable him to look in a different way at his drinking, and if it did, we'd be happy for him to return to discuss it with us. And we should also give the patient a copy of the patient booklet.

What if our patient is ambivalent – if he accepts that maybe he is drinking too much or too often, but does not want to make any changes?

- Some patients are hesitant about change despite going through a detailed assessment and having personalised feedback. This is not unusual.
- Again, the table above is helpful here: with a patient who has accepted that there may be problems but who does not seem ready to start to change things, we need to work on that patient's ambivalence, to help him with his decision-making. It is very likely that the patient will have seen that there are problems arising with his drinking, but he will also feel that his drinking is giving him lots of positives, so he is loath to give it up or cut it down.
  In these cases, we must not try to push the patient into committing to change. If a patient feels forced into making a commitment before he is ready to take action he may withdraw from treatment rather than lose face over the failure to stick to a commitment.
- Instead, we need to focus more on helping the patient to resolve the dilemma – to resolve the ambivalence - and to make up his mind to change in a positive direction. Lots of the examples we gave in *Chapter 3* in the section on 'How to elicit change talk from the patient' could be used here. For e.g. you could ask the patient:*"On a scale of 0 to 10, how ready are you to make a change right now in your drinking?"* with 10 indicating 'ready to change right now', and 0 'not ready to consider a change'. If the patient rates his readiness as 3, you can inquire, "*Why did you not say 0?*" which might prompt the patient to voice reasons for considering a change.
- When, even after a discussion about pros and cons, a patient remains unsure or hesitant about making a commitment to deal with his drinking problem, we often ask him to postpone the decision until later (although a specific time does need to be agreed upon to revisit and evaluate the decision). We might say something like this:
  'It sounds like you're not really ready to make a firm decision yet about changing your drinking. That's perfectly understandable. This is a very difficult choice for you. It might be better not to rush into anything by making a decision right now. Why don't you think about it between now and the next session, think about the advantages and disadvantages both of making a change and of continued drinking in the way that you are at the moment. What other things could you do between now and next time that might be useful to help us think things through? We can look at these things in more detail next time. If you do some of these things, I'm sure that it will become clear to you what you want to do. OK?'

With most patients, however, going through the assessment and providing personalised feedback will lead to the patient being ready to discuss making a change in his drinking. When we get to that stage with a patient, we need to clarify with him what his goals are.
(At this point you might want to use the hand out '**Are You Ready For Change?**' on pg 7 of the patient booklet and get the patient to tick the box - with your help if needed - to indicate where he is with regards to thinking about his drinking.)

Drinking and other goals
There are two different types of goals:

1. The overall goal about what the patient wants to do about his drinking
2. The more detailed 'sub'-goals that arise when we try to help our patient work out how he is going to achieve this overall goal.

So, if for example, our patient decides that he is going to stop drinking entirely (the overall goal of treatment), that will give rise to many more detailed sub-goals as we (the patient, his SO and us) start to plan out how he is going to do this. He may decide that he needs to change some of his friends, how he spends his time, how he deals with stress, and so on – each of these are sub-goals and we will need to work with him and his SO in trying to get clear what these sub-goals are.

The diagram below shows the overall goal (to stop drinking) with the detailed sub-goals beneath:

Figure 4: Overall goal to stop drinking

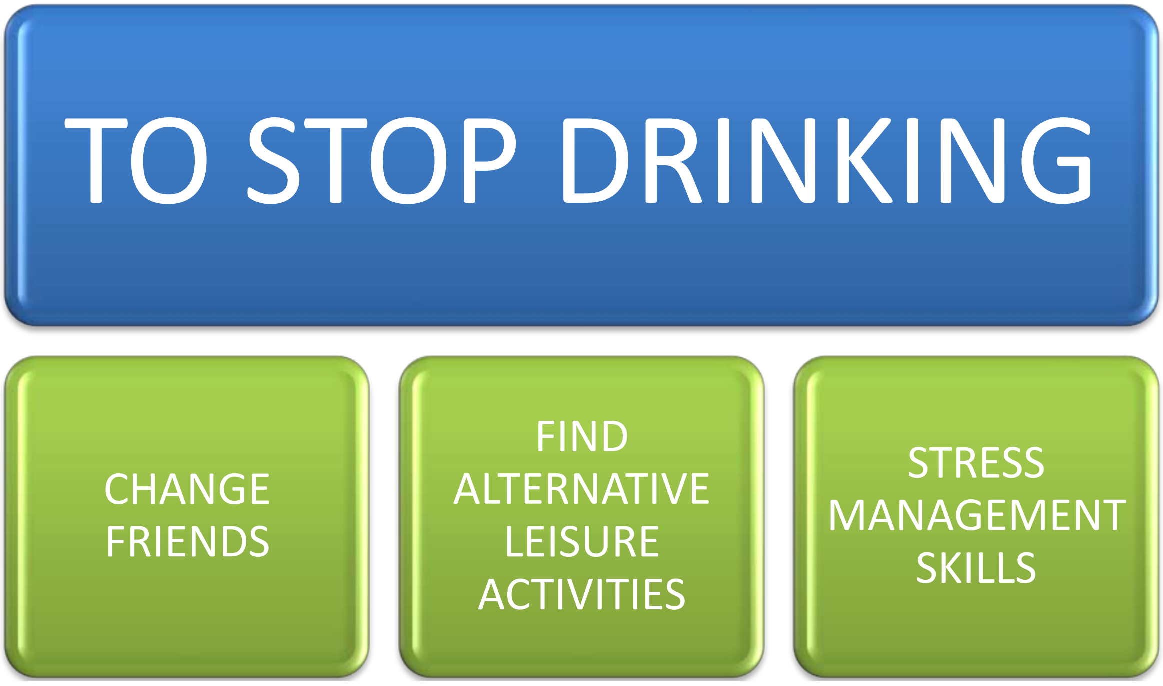

Types of drinking goal

Patients who want to change their drinking have two choices:

1. They can stop drinking completely, or
2. They can control their drinking.

'So Mr and Mrs Pereira, we have just gone over all of the issues about your drinking, and discussed the impact that it is having on your health and these other areas of your life. As you know, I am here to help you with these problems, but first we need to be clear what you want to do, if anything. What is your goal for this treatment? Is it to stop drinking entirely? Or is it to cut down on your drinking and try to control it?'

Although we may feel that, having done our assessment, one of these goals is more appropriate or realistic for our patient than the other, we should not choose drinking goals for our patients.

- If we choose the goal, it is much less likely that the patient will achieve it
- If the patient (and his SO if present) choose what to do, they are more likely to get there

Often our patient will be able to tell us which goal he would like to pursue. But sometimes it will be useful for us to have a discussion with him (and his SO if present) about the relative advantages and disadvantages of each goal, for this particular patient.

'I can see that you are not completely decided on which is the best goal to aim for. Often I find it helpful for us to look together at both the advantages and the disadvantages of each goal, the good things and the not-so-good things about each goal – often that makes it much clearer which one you might want to aim for. Are you happy with us trying this?

OK – let's look at cutting down on your drinking and try to control it. What do you think would be the main advantages of doing this? …..and what do you think would be the main disadvantages of doing

this? …. OK let's look at the other choice – stopping drinking altogether. What do you think would be the main advantages of doing this? …..and what do you think would be the main disadvantages of doing this?
I have written down all the advantages and disadvantages you have told me and, to sum up, you have said that …… Has this made things any clearer for you?'
Similarly, we have discussed these two drinking goals as if they are completely fixed. But an important principle that the patient could follow is to live 'one day at a time'. The idea of either giving up or controlling alcohol for the rest of his life can be a stressful thought for someone with alcohol problems. Instead of making such a lifelong commitment, he can make a commitment only for the next 24 hours. If he continues making this 24 hour commitment every day, this may lead to months and eventually years of abstinence.

Stopping drinking completely
If the patient chooses as the goal of treatment to stop drinking completely, this creates a relatively easier situation for us to handle. This is because most people understand what 'stop drinking completely' means, and we can concentrate on helping the patient (and his SO) to clarify how he can stop drinking completely, what the barriers to this might be, and how he can overcome each of these barriers.

Controlling drinking
If the patient chooses as the goal of treatment to control his drinking, this raises more difficulties, for a number of reasons.
First**,** many patients will simply wish to 'cut down on their drinking'. This sounds simple, but it often does not work. This is for a number of reasons. By the time people are drinking problematically, they will usually already have tried to cut down, and the fact that we are seeing them implies that they have not been successful. Also, 'trying to cut down' is not very clear – cut down by how much, and to what level? As we will explain below, 'controlled drinking' is a much more exact goal, which will specify in some detail both how much a patient will be aiming to drink, and all of the details of this – when, where, and so on. If the patients state that they do want to 'cut down', we need to find out if they have tried to do this in the past, and if so, what happened. If they have never tried to do this, it may be worth getting them to be clear about exactly how much they want to cut down to, and how they plan to do it. But in general, we should encourage patients who want to 'cut down' to aim for a more exact 'controlled drinking' goal, if they meet the other criteria below.
Second, there is a lot of evidence (though almost entirely from the West) about the types of people or situations where people find it much more difficult to control their drinking. If a patient chooses controlled drinking as a goal of treatment and falls in one of these groups, we need to explain to the patient (in a collaborative manner!) that this is the case.

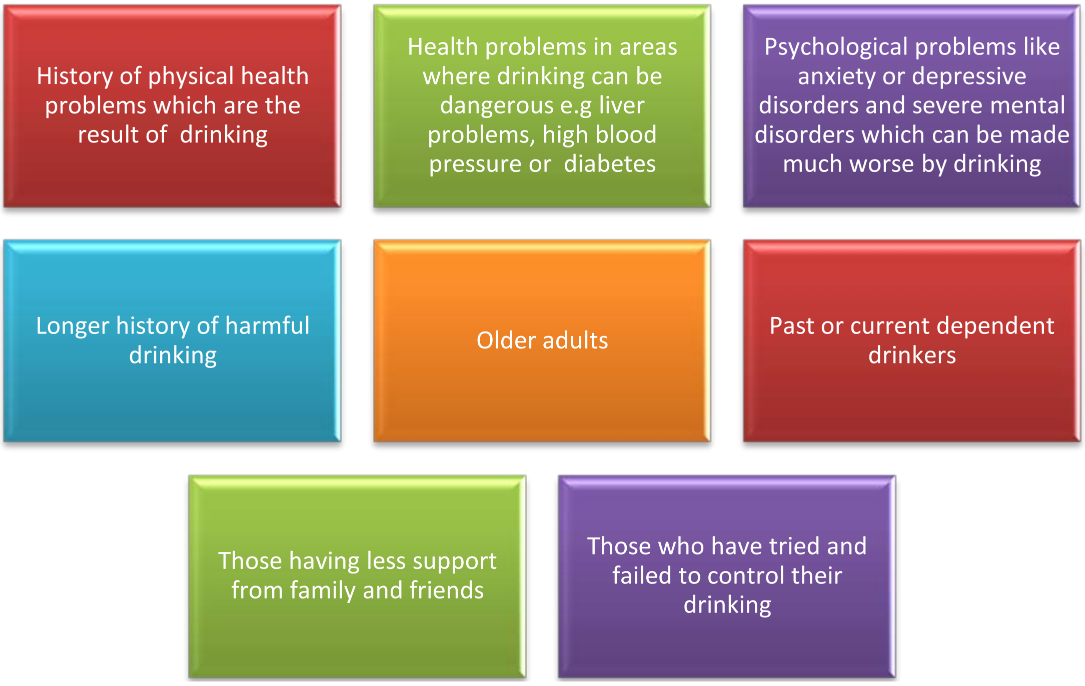

This is a challenging situation for us. On the one hand, we have a responsibility to inform our patients if they want to try something which might be detrimental to their health. Yet, on the other hand, we also need to maintain our collaborative way of working with the patient in which they choose the goal they want to work towards.

The following statements can help you to frame your response to such a situation

- *You are saying that you want to control your drinking (or to 'cut down'). Earlier, you had also told me that you have diabetes. What you decide to do is very much your decision, but I do want to tell you that there are real risks to you – to your physical health – if you do decide to continue drinking, even if you drink in a much more controlled fashion. And so even though this is very much your decision, I do want you to know that stopping drinking completely would be a safer choice for you. If you don't drink then you can be sure that you won't get any problems due to your drinking. However, whatever you decide, I will help you to achieve that goal.*
- *I understand that you want to control (cut down) your drinking and that is very much your choice and your decision* - *but I also do want you to know that there is a lot of research showing that people ….* (depending on what situation they have which is associated with a lesser chance of success with controlled drinking – *e.g. people who are older …, people who do not have a lot of support for controlled drinking from family and friends …, people with your history of health problems …,) are much less successful at controlling their drinking.* (At this point you can use the hand out '**When Stopping Is Advisable**' on pg 8 of the patient booklet and together with the patient, tick one or more reasons why not drinking at all would be safer and which apply to that particular patient.)
- *I understand that you want to control (cut down) your drinking and that is very much your choice and your decision. If after I give you some information you still want to try this, you will still have my help and support – but can I let you know what we already know about how people in your position have been unsuccessful at controlling (cutting down) their drinking?*
- *OK, you want to try to control (cut down) you're drinking. As I say, most people in your position do not manage to do this, but let's try – it may be different for you. But can we try this as an experiment? And if it turns out that controlling (cutting down) your drinking is more difficult than you thought it would be, we can go back and try stopping drinking entirely instead.*

However, even for the group of patients who don't have any of the above mentioned factors, there are a number of issues that we need to make the patient aware of:

Patients who want to control (or cut down) their drinking need to understand that this is much more difficult than stopping drinking completely. Stopping drinking completely requires one decision – not to drink alcohol. Controlled drinking requires many decisions: about how much to drink, when, where, and so on. It also requires the patient to re-make that decision once they have started to drink, and that is often very difficult. It is very common that people decide to limit their drinking, but after they have drunk one or two drinks, they make a different decision – i.e. to drink more. Another important thing that we need to make patients aware of is that drinking very moderately is much less common in India than it is in other parts of the world. In India, if someone drinks, they are more likely to drink each time until they become very intoxicated. So patients who have a history of frequent episodes of drinking to high levels of intoxication may find controlled drinking difficult.

Also, if we are to help a patient to control his drinking, we and that patient must have agreed on exactly what we both mean by controlled drinking. Many problem drinkers want to return to a time when they did not have a problem, and when their drinking was just 'social drinking', with no negative effects. We need to help our patients who want to control their drinking to understand that controlled drinking means that a person has to be aware, possibly for the rest of their lives, of exactly how much they are drinking, when, where, with whom, and so on. This is why it is called '*controlled drinking*' as opposed to 'return to social drinking' – there has to a strong element of conscious control, probably for the rest of the patient's life. This is another reason why controlled drinking is very difficult.

On the other hand, it is also the case that there are groups of problem drinkers who seem to be much more successful at controlling their drinking than they do at stopping completely. These groups are the opposite of some of the groups outlined above – that is, people who:

- Are younger
- Are in employment
- Have family support around them
- Have a shorter history of misuse
- Have lower consumption before coming for help
- Have less physical, mental, or social harm from their drinking
- Show no signs of physical dependence
- Choose controlled drinking
- Are all more likely to be successful at controlling than at stopping completely

Family members play an important role in the life of patients in India and it is recommended that the opinion of the close family members (who may include spouse, children, and parents, depending on the family) should be taken into consideration when deciding on a controlled drinking goal.

- Many family members may be against the drinker choosing a controlled drinking goal, and hence all efforts should be made by us to explain the controlled drinking goal to close family members. This is vital, because if the family members are not supportive of this goal, it is less likely to succeed.
- The patient also needs to understand that the support of their family is very important for whatever goal they want to achieve, so if the family is very much against their goal, they may need to reconsider that goal.
- Similarly, the family members need to understand how important their support is, so if they withhold their support, they are likely to not be helping their drinking relative.

Once we have helped our patient carefully go through the pros and cons outlined above in considering what their goal should be, we then need to help them achieve that goal (bearing in mind the idea that if this does not work out, we can always suggest later that they give the alternative goal a try).

How to help a patient stop completely

If our patient decides to stop completely, we need to work with him to develop a plan as to how to do this. We will need to consider a number of factors with him.

If he is a dependent drinker, we will need to make sure that he is referred to a Consultant Psychiatrist, who will also see him while we are undertaking CAP with him. This is because, as a dependent

drinker, he may need some medical help to control his withdrawal symptoms. As we stated above, with dependent drinkers, once we finish this first session we need to take the patient back to see the PHC doctor and ask the doctor to refer the patient to the psychiatrist.

For Dependent Drinkers we may also need to say:

'*From what you have told me, I understand that you will need some medicines to make you comfortable when you come off alcohol and this might even require admission to hospital for a few days. However, this will not in any way affect the work we can do together. What I would suggest, if that is alright with you, is that if you do need to go into hospital, we take a short break while you are in hospital and restart our sessions once you are discharged. If that happens, I will remain in touch with you over the phone. Is that fine by you?*'

Whether or not he is dependent, we will need to help him plan out a number of factors, including

- When he will stop (i.e. immediately, or this evening, or tomorrow)
- How he will deal with future drinking occasions which he may already have planned (visits to family friends, weddings, regular meetings with drinking friends, etc. We will work with him in later sessions on dealing with unplanned potential drinking occasions).

These will need to be looked at very carefully when we develop the Change Plan (see section 5 in this Chapter, below).

How to help a patient control his drinking?

In the same way, if our patient decides to control his drinking, we will also need to work with him to develop a plan as to how to do this. Again, we will need to consider a number of factors with him.

For most patients who want to control their drinking, and especially for those who are drinking heavily or with whom we think that control may be difficult to achieve, it is often useful to suggest that the patient has a period (which we and the patient can agree together – at least two weeks, more usually a month) where he does stop completely. This allows his physical system to start to recover; and it also makes a dividing line between his current (uncontrolled) drinking and his future **(**controlled**)** drinking. This division is also useful because once the patient starts implementing his Controlled Drinking Plan, he is likely to be using different skills, and possibly drinking a different drink to the one that he was drinking previously.

Helping a patient to control his drinking means that he has to be committed to doing a variety of things:

- Carefully monitoring his drinking, to be clear he is keeping to the prearranged limits – ask him to start completing the drinking diary immediately, as this is a habit that he will need to acquire
- Being absolutely clear about what those limits are, and how to deal with any lapses [Note – we cover dealing with lapses in the *Ending Phase*]
- Utilising strategies that mean it is far more difficult to change these limits once drinking has started (because we know that after having one or two drinks, many people do change the limit that they had agreed on, before starting to drink). Useful controlled drinking strategies include
  - Going to a bar much later (so there is less time in the evening to drink)
  - Taking only a limited amount of money
  - Telling drinking companions what the limit is and asking them to help by not offering alcohol once the limit has been reached, and so on
- Carefully planning out the details of the 'controlled drinking plan'. This involves us
  - Working out with the patient the times, places, and triggers responsible for his drinking going out of control
  - Teaching a range of alternative strategies, behaviours, and skills for dealing with these triggers

The key to controlled drinking is in our patients learning new skills related to appropriate and safe drinking

For many patients who have used alcohol in an uncontrolled fashion over an extended period, learning how to drink in a controlled way means re-learning, or learning for the first time, basic skills which make it easier to drink less. Many of these seem like helpful tips rather than skills, but what makes them skills is that they need to be learnt and practised. For example, the tip to have something to eat before going out drinking needs to be practised to such an extent that a client will never drink unless he or she has a full stomach.
Some of the skills that a patient aiming for controlled drinking might use are shown below, and the importance of helping the patient to be clear about why he is trying to change his drinking needs to be regularly reinforced by us in our counselling.

Before drinking

- Eat something first
- Go out later
- Take less money
- Don’t drink in places where you have previously drunk heavily
- Take alcohol-free drinks with you to parties, friends’ houses, etc

While drinking

<u>What to drink</u>

- Drink a large soft drink first
- Drink pints vs. large beers; singles vs. doubles; premium vs. strong beers
- Drink more diluted drinks - spirits with mixers as opposed to neat, shandy (beer plus lemonade or soda) vs. all-beer, half-and-half of alcohol-free and ordinary beer or lager
- Drink low-alcohol beers or wines
- Alternate between non-alcoholic and alcoholic drinks

<u>How to drink</u>

- Drink in sips rather than gulps
- Put the glass down between sips
- Don’t stand at the bar - sit down elsewhere
- Drink at the pace of the slowest drinker in your group
- Time yourself, for example only drink one drink every 30 minutes
- Avoid bar snacks like nuts and crisps, etc. - they make you thirsty
- Avoid rounds, but if they are unavoidable, don’t buy yourself one on your round
- Certainly make sure that you do not become intoxicated. If you think that having another drink might make you intoxicated, stop and do not have that other drink
- Practise refusing drinks
- Do something active while drinking – play cards or some other bar game?
- If you drink in a bar or friend’s house, go home when you have reached your limit

(When you are explaining these strategies to the patient, you can make use of the hand out ‘**What You Can Do To Control Your Drinking**’ on pg 9 of the patient booklet.)

A brief example of a controlled drinking plan might be:

Rules – what you want to achieve

- No more than 20 'drinks' (i.e. a small spirits, a small beer) drunk over the week
- Two days of clear of alcohol each week
- No more than four units on any day
- No drinking until at least 6.00pm, Monday-Friday inclusive
- Possibly (after six months) allow one 'binge' of an agreed maximum (say six units) once a month

How you are going to achieve it

- Monday and Thursday are days with no alcohol, unless altered by prior agreement with counsellor or with significant other, e.g. wife
- Only drink beer: problematic drinking was related to spirits, so now confine all drinking to beer (bar or when visiting friends). Avoid spirits.
- Preferred place of drinking is the bar. But only drink at the bar after an evening meal
- Cost of preferred beer is Rs50 per pint bottle. So take no more than Rs100 (the exact cost of two pint bottles), plus the cost of two soft drinks, with you to the bar
- First drink at the bar always to be alcohol-free (e.g a soft drink, unless alcohol-free beer is available)
- After each bottle of beer, always have a non-alcoholic drink as a spacer
- Ask the bar not to advance you credit
- Ask friends not to lend you money
- Ask friends not to buy you drinks

[A copy of a blank '**Controlled Drinking Plan**' is in the hand-outs in the patient booklet]

The key things about this controlled drinking plan include:

- The drinking limits and other strategies in the plan must be the patient's, not the counsellors
- Clearly, the preferred drinks, their costs, and other items can be altered to suit different patients, and the costs will certainly change over the years

The plan is set in conjunction with the patient learning other skills, such as how to deal with the issues which have been identified as triggers for this patient's drinking (e.g. how to cope with negative emotional states, or with pressure from friends)

Sub – goals

Once our patient (and his SO if present) have decided on what the overall goal of treatment is (stopping completely or controlling his drinking) we need to start to work out how he is going to do this. Many patients will say that they have now "simply decided", but generally patients need to do more than this, and plan out what they will need to do differently in their lives so that they can "just stop drinking" or can "stop drinking after 1 peg of feni".

When we do this work with our patient and their SO, they will come up with lots of sub-goals (about how they will spend their time, and with whom, and doing what). Our task is to help them work out what these smaller goals are, and to help make them specific.

These smaller goals need to be SMART-CV

Figure 5: Sub-goals

- **S**pecific and clear — S
- **M**easurable and verifiable — M
- **A**dequate to meet the problem — A
- **R**ealistic and achievable — R
- **T**imed (*when* the actions to achieve the goal will be taken) — T
- In the patient's **C**ontrol — C
- In line with the patient's **V**alues — V

Examples of what patients might say and how we might help to make them SMART-CV goals are:

	**Vague/unclear goal**	**SMART goal**
Specific and clear (S)	*I want to improve my relationships*	*I want to have far fewer arguments with my wife , my son and my friends, which occur due to my drinking*
Measurable and verifiable (M)	*I want to be in control of my drinking*	*I want to control my drinking to one peg of feni every week for the next two weeks*
Adequate to meet the problem (A)	*I will control my drinking so the problems with my liver can improve*	*I will stop drinking completely so the problems with my liver can improve*
Realistic and achievable (R)	*I will never ever drink again*	*I will commit not to drinking alcohol for at least the next month*
Timed (T)	*I will stop drinking in the coming days*	*I will stop drinking from next Monday*
In the patient's control (C)	*I will have a great relationship with my wife so I do not go back to drinking*	*Although I can't control my wife's behaviour, over the next two weeks I shall try hard to not react to any negative comments she makes*
In line with the patient's values (V)	*If I stop drinking I can have more money to spend on gambling*	*If I stopped drinking I would be able to work regularly, and be able to provide better care for my children*

Similarly, our patient's drinking may have led to problems in other areas of his life, and he may also want to develop goals to improve these.

These areas can include:

- Improvement in family relationships
- Reduction in domestic violence
- Improvement in work performance
- Regaining lost social status
- Developing interest in alternative pleasurable activities

We should work with the patient and the SO to help them identify specific areas from this list or any other areas perceived as important and requiring change by the patient and his SO. We do this by using the 'Change Plan', as described in the next section.

The specific role of the SO in goal setting

The SO has a major role to play in helping the patient decide on both his drinking goal and on detailed sub-goals.

With the drinking goal, SOs (especially spouses) often have very strong views in favour of the patient stopping drinking completely, so if the patient chooses this as their goal, then generally there are hardly any incidents of difference of opinion between the patient and their SO.

On the other hand, if the patient wants to try controlled drinking as a goal and the SO is convinced that he should stop completely, we will need to mediate between them to reach a decision over what the goal is, and to affirm the importance of the patient making decisions.

Patient	*I want to control my drinking. I am sure that I can.*
Counsellor	*OK Mr Patil, that is clear. Mrs Patil, are you in agreement with this as a goal?*
SO	*No I am not! It is very clear to me that my husband cannot control his drinking – I have been asking him to cut down for many months now, and he always says that he will, but he never does – and even if he does cut down for a week or two, he just starts drinking more and more again, and being very argumentative when he comes home.*
Counsellor	*Hmm – Mr Patil, what do you think about that? It seems to me that your wife is asking a good question here – if you have tried to reduce your drinking in the past and failed, why should it be different this time?*
Patient	*This time I have you to help me!*
Counsellor	*Yes, that is true – but I won't be with you in the bar when you are with all of your friends and you try to cut down and stop drinking after a peg.*<br><br>*Can I sum up here with what I think? We have gone over the fact that controlling your drinking is much harder for people than simply stopping, and also that it is more difficult for people of your age. But we have also gone over the fact that patients are most successful at doing what they want to do, and you are saying that you want to control and not stop. However, it is also the case that trying to achieve a goal without the support of your wife makes it very much harder to achieve that goal. So I have a suggestion. I think that we need to try one of these goals for the next week or two, and see what happens. We can try for you to control your drinking, and if we do that, Mrs Patil, I'd like you to try your hardest to support and encourage your husband in doing this. Or we can try stopping completely for the next week or two, again with you Mrs Patil trying your hardest to support and encourage your husband in doing this. So – all things considered Mr Patil, what do you think you should do?*<br><br>*[To be followed by a question to Mrs Patil - all things considered, and given what I have said about the importance of your support – will you be able to support your husband in trying to achieve this goal, for the next week or two?]*

Similarly, the SO has a big role in helping the patient decide on the more detailed sub-goals that will need to be thought through. The SO will have views and may also put in new information which will

help us clarify what the most sensible sub-goals might be. For example, if the patient is thinking about changing some of his friends, or about how he spends his time, or about how he deals with stress, and so on, his SO is likely to be able to contribute a lot to these discussions.
A difficulty that can come up is that although the SO will agree the drinking and other goals, they may have too high expectations about what can be achieved and how fast these achievements can be made. A counsellor needs to be alert for such over high expectations which could over pressurise the patient and have the opposite result. When that happens, it is the counsellor's duty to give the SO and the patient a realistic estimate of what can be achieved and how fast it can be achieved.

HELPING OUR PATIENT (AND HIS SO) TO DEVELOP A CHANGE PLAN AND AN ACTION PLAN

Whichever of the areas above are chosen to work on by our patient (and his SO), we need to help them work out a '*change plan*'. We will start on this in this *Initial Phase*, but we will do more work on it in the *Middle Phase*, when we concentrate on helping the patient to make the changes discussed in the plan. As well as the overall change plan, we will also clarify what the Action Plan is – i.e. what tasks the patient is committing to do in between this session and the next one. This will include any specific homework tasks, such as completing a drinking diary.

A change plan has two steps:

Discussing a plan:

As we described above, when our patient **(**and his SO**)** decide on the drinking goal, that gives rise to many more detailed goals as we (the patient, his SO and us) start to plan out how he is going to do this. Similarly, our patient's drinking may have led to problems in other areas of his life, and he may also want to develop goals to improve these.
In this phase of developing a 'change plan' we try to generate some ideas from the patient (and his SO) about how these sub-goals might be achieved. As always, we must not prescribe a plan for how the patient should change; instead we work with the patient (and his SO) to develop the change plan. The overall message is that "*only you can change your drinking and other areas of your life and so it's up to you (although I certainly can help you do this once you have decided what you want to do).*"
So if the patient (and his SO) decides that he needs to change some of his friends, or how he spends his time, or how he deals with stress, this phase of the plan is taken up with discussing with him how he might go about doing this.
There are some key areas that we will need to ensure that the patient (and his SO) thinks about, when they are considering each sub-goal.
These key areas include:

- *What are the changes that I want to make? E.g. Sub-goals under this main goal could be how do I want to spend my time differently? Or how do I want to deal with stress differently?* (Note: These sub goals would change from patient to patient)
- *Why do I want to make these changes? Have I thought through what the likely consequences are if I do make these changes?*
- *How do I think that I will actually make these changes – what steps will I actually take to spend my time differently or to deal with stress differently?*
- *How will I know if this plan is working? What will I expect to see or to feel which would tell me that I am dealing with stress or spending my time differently?*
- *What could go wrong or could interfere with this plan?*

However, do note that we will spend a lot of the *Middle Phase* on this issue, so do not expect to go into great detail on each of these areas. The purpose of doing this in the *Initial Phase* is so that the patient and his SO will be able to go away from the session with some sort of plan that they can try out in between this session and the next one.

The change and action plan worksheet

The **Change and Action Plan Worksheet** (CAPW) is useful to help in summarising the changes that the patient wants to make and the actions he wants to take before we meet for next session and also to plan homework with the patient. Specific meanings of change plan, action plan and homework which

are already described in the section on the overall structure of the session, are repeated below for easy reference

- **The Change Plan** contains the overall plan, outlining what the changes are that the patient wants to achieve in counselling (e.g. to completely stop drinking). The first two questions in the list above are related to the change plan.
- **The Action Plan** is the set of actions that the patient and the counsellor have agreed will be done in between one session and another. So a key task is to clarify what detailed steps are that we have agreed that the patient will be taking in between this session and the next. Last four questions in the **CAPW** are related to action plan. Once we have agreed about the areas of change and the reasons for change then we directly move on to working with the patient and SO (if present) on the action plan
- This may include any specific homework tasks (such as completing a Drinking Diary or acquiring some specific information) that we have agreed that the patient will do in between our sessions.

We must not do this just as a check-list. The answers need to come after a substantial discussion between us and the patient (and SO if present). The CAPW is a way of summarising the results of these discussions. We need to use the CAPW as a guide to ensure that we have discussed these aspects of the patient's plan:

- "*The changes I want to make are ...*" In what ways or areas does the patient want to make a change? Be specific. It is also useful to include goals that are positive (*I want to begin..., to increase..., to improve..., to do more of...*) as well as negative (*I want to stop..., to avoid... to reduce...*).It is also helpful to get our patient to organise the list, starting from the changes he wants to achieve first and ending with the changes which are last on the list of priority
- "*The most important reasons why I want to make these changes are ...*" What are the likely consequences of action and inaction? Which motivations for change seem the most important to the patient?
- "*Specific changes that I want to achieve before I meet with the counsellor again…*" Ask patient about the specific things that he wants to work on before he meets you next time. This list is referred to as an action plan and includes one or more target behaviours e.g. stop drinking completely or handling influence of friends in different way
- "*The steps I plan to take in changing are ...*" How does the patient plan to achieve these goals? How could the desired change be accomplished? What are some specific, concrete first steps that the patient can take? When, where and how will these steps be taken?
- "*I will know my plan is working if ...*" What does the patient hope will happen as a result of this change plan? What benefits could be expected from this change?
- "*Some things that could interfere with my plan are ...*". Help the patient to anticipate situations or changes that could come in the way of the plan. What could go wrong? How could the patient stick with the plan despite these problems or setbacks?
- "*Specific things I will finish as homework before we meet next time ….*" We can help the patient to decide on specific things that he will perform as homework before we come next time

Pre-printed Change and Action Plan Worksheet forms are convenient for us to use. (You can also use the '**How I Will Change My Drinking**' and '**Things I Will Do**' hand-outs on pg 15 and pg 21 of the patient booklet).

The specific role of the SO in developing the 'change and action plan'

The SO can work as an important resource for a patient in his efforts to work on the change plan.

If a patient is not accompanied by his SO, it will be useful to know from the patient about who this SO might be – who does this patient have in his close networks who can play a helpful role in implementing this change plan. As we have said already, it would be very helpful to invite that person to the next session, if the patient agrees.

If the SO is already present in the session then we can ask the patient about the possible role an SO can play implementing this change plan and involve the SO in this.

Counsellor	*Mr Pawar, how do you think your wife can help you in implementing this change plan?*
Mr Pawar	*It will be really helpful if she can prepare dinner early and give me a call at around six to remind me to come directly home and that the food is getting ready - then I may not get diverted to the bar on the road.*
Counsellor	*Will it be possible for you Mrs Pawar to do that?*
Mrs Pawar	*By all means!*
Counsellor	*Great. How about trying this during this week before we meet next?*
Mr & Mrs Pawar	*Sure we can*

If the SO has a different opinion about the change plan then we need to discuss this and reach some conclusion. Clearly if the SO is raising an objection which is likely to mean that the plan would not work, we need to re-consider the plan. But if the objection is simply about a preference (e.g. patient wants to go to the temple when he feels stressed and has the urge to drink but SO wants him to stay at home as she believes that going to the temple would only increase the risk of him meeting his drinking friends on the way), then it may be useful for us to discuss this preference briefly, but then explain to the SO that though their participation in the process in very important, the chances of success are much higher if we go by the patient's preference about change plans.

Ending session one
Five important things to do before we end our first session are:

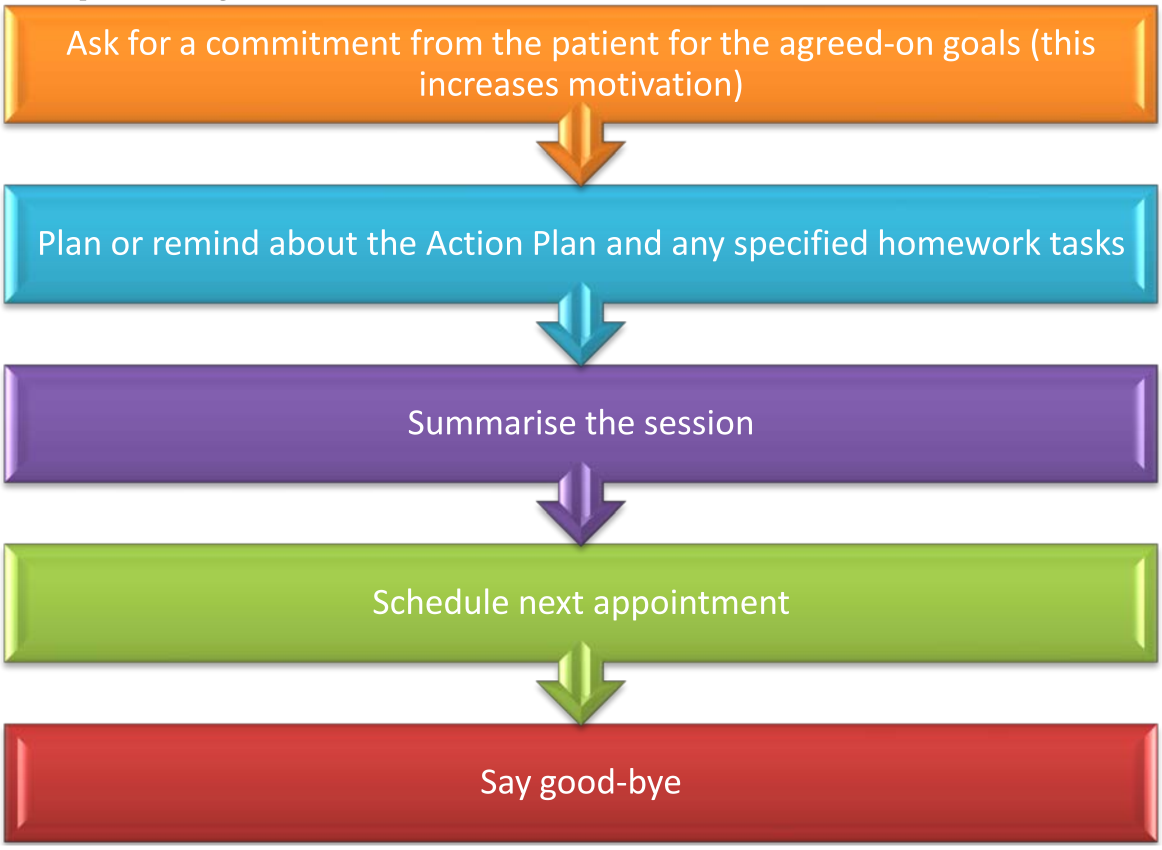

<u>Ask for commitment and further develop our patient's motivation for change</u>
Once we have developed this 'change plan', we want to get a 'formal' commitment to change. What we want is to get the patient to hear him make a verbal commitment to take concrete, planned steps toward change.
The key question (not necessarily in these words) is:

'Are you ready to commit yourself to making these changes?'

If the patient is willing to make a commitment, ask him to sign the CPW and bring it with him when he comes for the next session.
It is very important that we are very positive with the patient and his SO. Hearing us is positive, and hearing the patient re-state his commitment, are both very motivating factors.

Plan or remind about the action plan and any specific homework
There will always be tasks and activities that will have emerged in our sessions, things that either we or our patient (or his SO) will have suggested could be tried or undertaken in between sessions. We need to keep a record of these as we go through the session, and ensure that these are summarised in this Action Plan at the end. So this is a summary of what the patient and his SO have agreed that they will do in between now and the next session. Many of these things may already be listed under Section 2 of the CAPW *(The steps that I want to take).*Some of these tasks may be specific homework ones, such as gathering some specific information, or completing a drinking diary. It is useful to start by asking the patient to summarise what they have agreed their tasks are, and to then get the SO to add in any which have been forgotten, and finally to add in any further ones that the patient and his SO may have missed out.

Summarise
At the end of the session, it is useful to ask the patient to give a broad summary of what has been discussed in the session; ask the SO if they have anything to add; and then to reiterate the key things ourselves.
If the patient finds it difficult to summarise the discussion, we can ask the SO, and then we can offer our own summary. The summary should emphasise 'change talk', plans for change and the patient's and the SO's perceived consequences of changing and not changing. We can use our notes on the CAPW as a guide. Here is an example of how a summary might be worded:

'Can I summarise what I think we have done today? We reviewed the reasons why you have been worried about your drinking. There were a number of these. You were worried that your drinking has led to problems in your family, both between you and your wife and with your children. You were worried too about your blood results which showed that alcohol has been damaging your health. You seem to have been drinking more and more over the years. The accident you had has made you realise that it was time to do something about your drinking, but I think you were still surprised when I gave you your feedback, and you could see how much damage was being caused by your drinking.

You have said that you want to try out a period of not drinking at all, for say 3 months, to see how it goes and how you feel. You have also realised that most of the times you drink with your friends and saying 'no' to them is going to be a challenge and we have agreed that we should spend time in our second session discussing about how to say 'no' to friends. We have also agreed that you will try and note down in the drinking diary that I have given you every time between now and our next session when somebody offers you a drink and your responses to that. We have also discussed leaflet No X in the patient booklet where you can find some tips on how to handle a situation when somebody offers you a drink. You have also agreed to bring your wife with you for our next session so we can discuss with her about the possible role she can play in this process. Does that seem about right? Have I missed anything?'
If the patient offers additions or changes, reflect these and integrate them into your summary. Also take a note of them on the **CAPW (**pgs 15 and pg 21 of the patient booklet).

Scheduling session two:
The second session should be scheduled within the next one to two weeks based on the mutual convenience of the patient and the counsellor. Note that subsequent sessions can be done in the clinic, at the patient's home or over the telephone, as agreed with the patient. Please refer to the CR manual for details of how home visits and telephone sessions should be conducted.
Saying good-bye
The final part of the session is saying good-bye. Briefly, we should cover these points:

- The possible ways, in which the patient can contact us for any urgent matters, including changing the appointment
- A statement that if the patient misses his appointment, we will get in touch with him on the phone (if he has given us permission to do so) to arrange another appointment
- Finally, thank the patient for working with us, and bid him farewell warmly and with a reminder that we are looking forward to our next meeting

Special situation: Providing a very short version of session one

Note: We should only provide a short session if we have to - it should not be our first choice. We must try our best to convince the patient to stay for a full session. If it is absolutely not possible to do that only then should we consider the short session.

Some patients may be in a hurry to leave and may not be able to stay for the entire duration of 40 to 60 minutes that we need to deliver all the steps that we have outlined above. Before we begin the session we should ask the patient *"This discussion will take about 40 to 60 minutes. Will you be able to stay for this long?"* If the patient expresses agreement, then we proceed with the full first session. In case the patient expresses an inability to stay for the entire duration of the session, for whatever reason, we must deliver a brief session to the patient in which we cover the key points and then continue the remainder of the first session at the next visit.

We may say:

"Then we will need to spend about 10 minutes together now, as we do need to go over your answers to those questions that my colleague asked you before you came to see me and also how I might be able to help you if you decide to come back to see me for a full session (the 40-60 minutes I mentioned)".

We can then deliver a brief session in the following manner:

Step 1: Explain to the patient what his AUDIT score means and a brief personalised feedback based on what is recorded in the AUDIT.

You can say the following

'My colleague asked you some questions when you came to the PHC today, and I would like to share my thoughts with you about this. This might then help you to make decisions about your drinking.

Your AUDIT score is XX which puts you in the category of harmful drinking. It means that you drink more than many most men in Goa and at a level where it is causing harm to your physical and/or mental health. You have mentioned to my colleague that you drink a quarter of feni 2 to 3 times a week. You also mentioned that in the past year there have been quite a few times when you have been unable to remember what happened the night before because you had been drinking. Today when you saw the doctor he told you that your liver is damaged because of your drinking. I think that it will be useful for you to also know that men from Goa who drink as heavily as you do are also more likely to get psychological problems (such as depression or tension), as well as many more health and other problems of the sort that you are already getting. They are also more likely to go on to become dependent on alcohol, and to develop even more serious problems.'

Step 2: Provide a brief description of the counselling with an emphasis on providing encouragement and hope

'There are three parts to the counselling work that we do here. In the first part, we get to know each other, discuss your drinking and whether it has given rise to any problems, and we decide together what you want to do about it. We have made a start on doing that today, but when you come back, we will look at your drinking in more detail and see if there are any other areas of your life that it is harming. But we will also look at the positives that drinking is giving you, and whether there are ways that you can still get those positives if you reduce the amount that you drink. The most important thing is that at the end of this first part, you make a decision about what you want to do. In the second part, we plan out together how you are going to make any changes that you want to. In the third part, we work out how to make those changes long-lasting. We will meet a maximum number of

four times, although we may get these three parts competed faster – with some people it only takes one or two meetings, with others, 3, or all 4 that we have available.'

STEP 3: Explain what we didn't have time to talk about - this will include discussing details of the patient's problems, their effects on the patient's health and specific aspects of the counselling. Reassure the patient that this will all be covered the next time we meet. You could say the following:

'Ideally, if you had the time today, we would have looked in more detail at the answers you gave to the questions that my colleague asked you, and then I might have asked some more questions, so that I can get a better understanding about your drinking. At the end of that, I would have offered you my impression about your drinking. We could then have decided together whether you want to work with me to decide if you want to change anything about your drinking. Since you don't have time today, we can do all of this when we meet again'

Step4: Set the next session date. Try to keep the date as close to the screening date as possible. The longer the gap between screening and first session, the lesser the likelihood of the patient returning.

Step5: Complete appropriate documentation

SUMMARY

There are a number of tasks that we have in the Initial Phase of CAP.

- Set an agenda
- Introduce CAP to our patients, Talk about the specifics of counselling
- Complete the additional assessment of the drinking to supplement the AUDIT
- Give our patient personalised feedback about his drinking based on the AUDIT and the additional assessment we have done in the session
- Help our patient to decide on his drinking and other goals
- Help our patient to develop a change plan
- Ask for commitment and further develop our patient's motivation for change
- End the session well by summarising the Action Plan and any specific homework, getting the patient (or us doing this) to make an overall summary, setting the next session date and saying goodbye.

Phase One of CAP will usually take all of our first session.

With some people, it may take longer, and we will not get all of these tasks done in one session. In that case, we may finish the session before we have completed all of the tasks, and try to arrange to have a second session as soon as possible, preferably within a week and not more than two weeks later. We must try to motivate the patient as much as possible to come back so that we can finish making our assessment, providing personalised feedback, and then helping to decide the goals of treatment and the Change and Action plans.

With other people, we will complete all of Phase One and even make a start on Phase Two in this first session (or move on to Phase 3 is the person has already stopped drinking).

Chapter 6

CAP Session-By-Session Guide

C. Middle Phase (Usually Session One)

Learning Objectives

In this chapter, we will learn:

- Discussing what has happened in relation to the patient's drinking in between the first session and this present one
- Look again at the change plan and clarify whether this is still what the patient and his SO want to do
- Help the patient implement the change plan
- Work out with the patient (and his SO) how he is going to implement the change plan. This will also help us get clear what new skills and techniques the patient needs in order to make the changes that the change plan calls for (that is, we work with the patient on thinking and behavioural skills)

CONTENT

As discussed before, throughout this phase we still have the same key overarching task we described earlier – to develop the patient's motivation (engagement, working together, developing and encouraging change talk, identifying and discouraging sustained talk). Also, as in the *Initial Phase*, as far as possible we will try to involve a significant other (SO) in the treatment to help the patient achieve whatever goals we agree on (as long as the patient agrees that they want this 'significant other' to be involved).

Again, as outlined in *Chapter 2*, this Middle Phase is most commonly undertaken in Session Two, although for some patients we will get onto some of the tasks outlining in this chapter in the first session, and for others we'll spend all of the first two sessions on the *Initial Phase* and only get onto these aspects in the third session.

Also as discussed previously, this second session (and any future ones) will have a structure: We (the patient, the SO (if present) and us as the counsellor) will

- Set an agenda between us and the patient (and their SO)
- Review progress on the Action Plan and specific homework since the last session
- Work on the target behaviour(s)
- Plan Action Plan and specific homework
- Summarise.

[Patient's initial concerns: It is possible that the patient will have presented initially with physical health concerns (the reason why he attended the PHC in the first place). Although we do not raise this issue with the patient explicitly (we are counsellors, not doctors) we are assuming a) that the patients' health concerns will be dealt by the PHC doctor, and b) that our work helping the patient to change his drinking behaviour will deal with the health concerns in a different way.]

Once we have set the agenda for the second session and reviewed progress on the Action Plan (AP) and any specific homework since the last session with the patient (and his SO), most of the rest of this second phase will be dedicated to working with the patient and his SO (if accompanying) on these target behaviour(s).

- First, we look again with the patient and his SO at the change plan and clarify whether this is still what the patient and his SO want to do
- Second, we help the patient and his SO to implement the change plan. We work out with the patient and his SO how he is going to implement the change plan

Working this out with the patient and his SO will show us what new skills and techniques the patient needs in order to make the changes that the change plan calls for.

Counsellor	*It is good to see you again Mr Naik, and I am very pleased that you have brought your wife with you today – good morning Mrs Naik. Your husband may have told you that we start each session by working out what we will cover in the session, so can we do this now? Mr Naik, what are the things that you would like us to discuss this time?*
Mr Naik	*It has been difficult since we last met. I tried to control my drinking but* [Counsellor interrupts at this point]
Counsellor	*OK – can I stop you here Mr Naik – I understand that we need to discuss how things have gone since we last met – but I want us to get clear what we are going to talk about, before we actually talk about things. So one thing we will talk about is how things have gone and any problems you have had in controlling your drinking. Another thing I'd like us to talk about is to hear from you, Mrs Naik, about what your husband has told you about what we are doing here, and to see what your views on that are? Are there other things you would like us to talk about Mrs Naik?*
Mrs Naik	*Yes, my husband is still very argumentative when he has been drinking and it is causing many problems at home.*
Counsellor	*OK – we will certainly talk about that as well. So – can I summarise?*<br>• *We will start today with you, Mrs Naik, telling me what you know about what your husband and I talked about last week*<br>• *Then we will hear from Mr Naik about how things have gone with the Change*

	Plan that we discussed last time, and the specific things that we agreed last time that you, Mr Naik, would do<br>• *Based on the things that have happened since we met last, we will make sure that the goals that you chose and are in your Change Plan are still what you both want to have happen*<br>• *Then, we will work on getting clear why any problems that came up with you stopping / controlling your drinking happened, and what you can do to make sure that you can achieve your goal in the future.*<br>• *Finally we will talk about what you, Mrs Naik, have described as your husband's 'argumentativeness'.*<br>*Then at the end, we will work out what your homework is that you both will need to do.*

REVIEWING PROGRESS ON THE ACTION PLAN AND SPECIFIC HOMEWORK

Once we have set the agenda for the session with the patient and SO (if present) then we can move on to reviewing the progress made by the patient about the tasks that we agreed in the change plan worksheet and the Action Plan last session

There are various possibilities that could have happened during the period in between, including:

1. The patient has not followed any things that we agreed in CAPW (pgs 15 and pg 21 of the patient booklet)
2. The patient has followed some things but not all that we agreed in CAPW
3. The patient has followed all the things that we agreed in CAPW

Based on what the patient tells us, the second session will be focused on different things mentioned below.

1) <u>If patient has not followed on anything in the CAPW</u> there are two possibilities in this scenario

a) The patient has not developed enough motivation for committing to implementation of change plan and

b) The patient has developed motivation but is lacking in the skills for implementation of change plan

The course of action we take will depend on the reasons the patient and SO (if present) offer for the failure to implement the steps we had agreed on the change plan. If there is a problem in the level of motivation of the patient, then it is useful for us to continue the motivational development work with the patient using the skills we have learnt in the developing motivation skills section.

If we realise that the problem area is not the motivation of the patient but instead is their lack of skills to implement the change plan, then we can focus the second session on discussing and practicing the skills required for implementation of the change plan.

Four important skills which are generally useful in implementation of change plans are discussed in detail below. Based on what problems have arisen for the patient in trying to implement the plan, we can work with the patient on one or more of the skills described below. The important thing for us to remember is that when we work on the skills for implementation of a change plan, we need to do this in a collaborative way, rather than a bossy way as we have discussed. The Table below can help us to choose the skill(s) to work on based on the patient's problem area(s).

Table 3: Patient's problem areas and skills used to work on them

**Problem area**	**Skills**
Patient having multiple problems in life	Problem solving skills
Difficulty in saying 'no' to friends	Drink refusal skills
Excessive desire for drinking	Skills for management of drinking urge
Stressful life situations and difficulties in handling emotion	Emotions management skills

2) If the patient has successfully followed some but not all areas in the CPW and AP

In this scenario we should congratulate the patient and encourage him to talk about the changes that he has achieved. Once we have done this then we can move to areas which the patient has found difficult to implement and help him to tell us the reasons for the difficulties and the ways in which the difficulties can be handled. This process will follow the way described above for the first group of patients.

3) If the patient has worked on all the areas in the CAPW

Similar to the second scenario we should congratulate the patient on his achievements and ask him to elaborate on the efforts that he under took to achieve those goals. Once we do this then we can ask the patient for any other areas that he wants to discuss which he found difficult to manage while he worked on the CPW and work with him (if he wishes) to help him to sharpen the skills in the specific area e.g. the patient has managed to comply with the CPW but mentions that he really had a tough time in managing requests by friends for him to join them for a drink. In this scenario we can work with him on drink refusal skills in this session.

If the patient and SO (if present) do not come up with any challenging situations, then we can move to Phase Three of CAP, and discuss issues related to lapse and relapse (covered in *Chapter 7*).

WORK ON THE TARGET BEHAVIOUR(s)

As shown in *Table 3*, our patients will often have difficulties in implementing the various tasks that we agreed he would do in between our sessions. Difficulties in implementation usually arise

- Either because the patient is still ambivalent about changing, in which case we use the motivation-enhancement techniques which we discussed in *Chapter 3*, or
- Because the patient lacks some of the skills needed to actually do what we had agreed

There are four important thinking and behavioural skills which can be useful when implementing a change plan. For each one, we need to clarify

- If this is a skill that this patient needs for his situation, and
- Whether he already has these skills or if we need to teach them.

Again, the table above will help us in clarifying whether the difficulty in implementing some or the entire plan relates to one or another of these skills.

1. Problem solving
2. Drink refusal skills
3. Handling urge to drink
4. Handling your emotions

All of these skills are useful in achieving the patient's drinking related goal (either complete stopping of drinking or control over drinking). At the same time they are also useful preventing relapse which is described in details in phase three.

When we work on these four sets of skills, we will often be helping our patients develop and use their own '*coping skills*' [see *Appendix 1*]. Coping Skills are skills that our patients use to deal with (or cope with) challenging situations. These situations could include having an urge to drink, or feeling some negative emotion, or being pressurised to drink alcohol when it is not wanted. We will look at Coping Skills in some of the four different areas below.

1. Problem Solving

Learning Objectives

- What is the importance of problem-solving skills in the treatment of Harmful Drinking?
- What do we mean by problem solving?

CONTENT

WHAT IS THE IMPORTANCE OF PROBLEM-SOLVING SKILLS IN TREATMENT OF HD?

Patients with harmful drinking often report life problems that they find difficult to handle. Many times a patient's inability to handle these problems is closely related to his drinking (so for example a patient may say '*I argue with my wife and then I go out drinking*" or "*my worries about my work mean that I drink every night to help me forget them*'). The problems may be in the form of financial difficulties, relationship issues, health concerns or work-related issues. Working with a patient on problem-solving skills enables him to handle problems in life effectively, which in turn helps him to achieve his drinking related goals. Problem-solving is also very useful for working with patients with Depression and the steps described here are similar to the ones in the HAP manual.

WHAT DO WE MEAN BY PROBLEM-SOLVING?

Problem solving involves helping the patient to

- Learn the skills of identifying problems that are contributing to their harmful drinking
- Generate multiple solutions
- Apply the most appropriate one
- Review the solution for its effectiveness

WHEN DO WE USE PROBLEM-SOLVING SKILLS?

Problem solving skills are important counselling skills which can be used throughout each of the CAP sessions, but most importantly are used in the context of developing and working with the patient and his SO on the change plan work sheet.

HOW TO USE PROBLEM-SOLVINGSKILLS?

Steps of problem-solving

Problem solving is a six-step process (shown in *Figure 6* below). Before we start our work with the patient on their problem-solving skills, it is useful to ask the patient about his ways of solving problems in day-to-day life. During the course of life most of us develop our own methods of solving problems which often include some or other of the steps described below. It is useful to link the patient's own methods of solving problems with the problem-solving steps mentioned below.

Figure 6: Steps of problem-solving

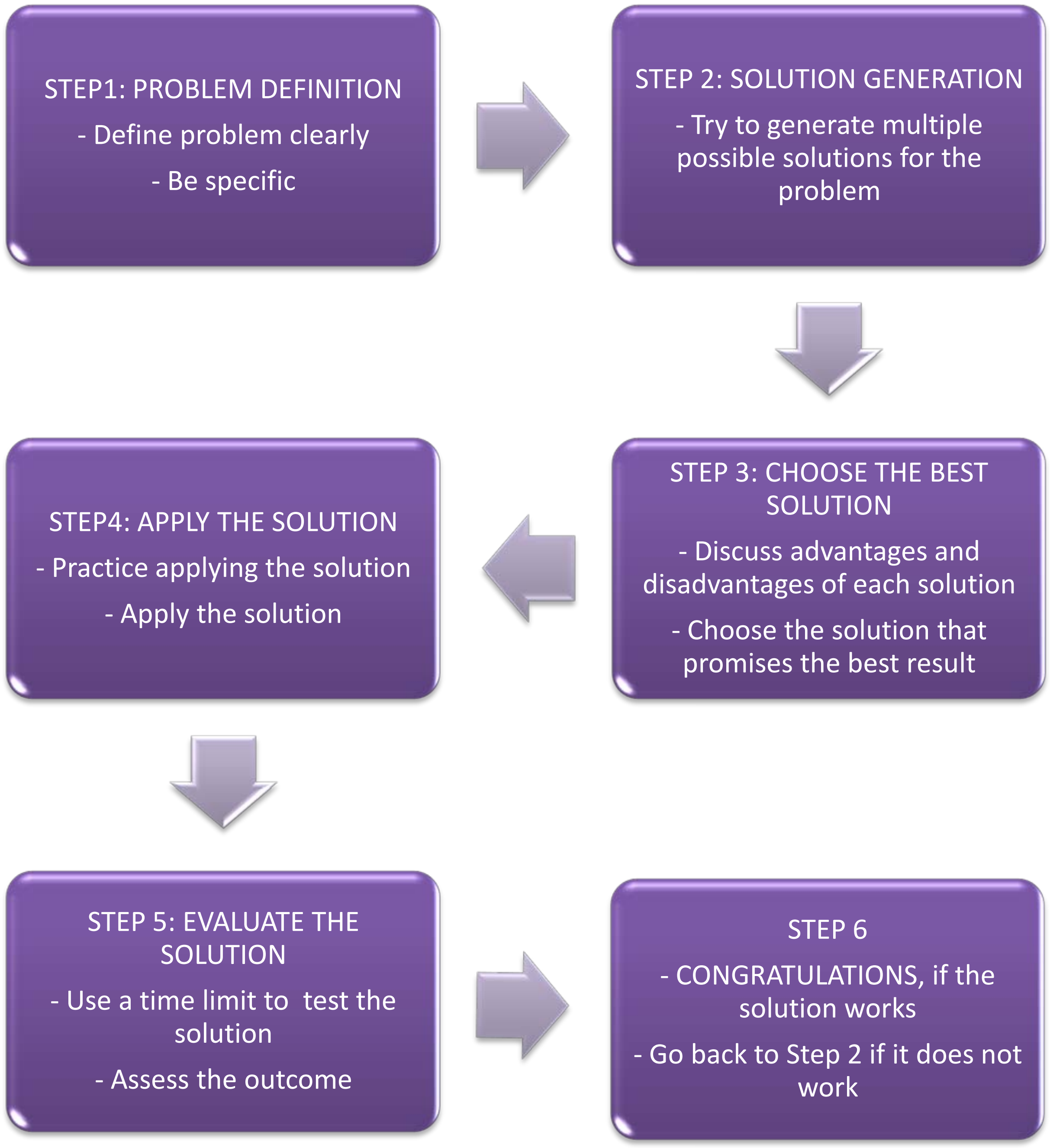

(You can use the hand out '**Solving Life Problems**' on pg 12 of the patient booklet to explain these steps to the patient.)

Sometimes, especially if the patient seems eager to learn or if we have enough time, it is useful to actually teach these six steps to the patient in Phase 2; at other times we can use them as a background framework to guide both our discussion during the session and the development of the Action Plan for the patient to implement between sessions.

It is also likely that we will return to these skills in Phase 3, when we deal with lapse and relapse: at that time we will go over these steps again and try to embed them with the patient, so that he will in future be able to go through the process on his own and solve future problems without needing our help.

The script below demonstrates the skills of problem solving.

Script 1

Umesh is a 32-year-old married male who is drinking harmfully. He reports that his financial problems are an important reason for his drinking

Who said?	What did they say?	Comments
**Counsellor**	*Umesh, in the last session you told me that you want to work on the relationship between your financial problems and your drinking – right?*	Counsellor revisits the discussion from the last session and helps patient to set agenda for this session
**Umesh**	*Yes we decided last time that we will talk about this when we meet next. You know I drink because I have this big loan I have taken to purchase a tourist car and now I am finding it really difficult to pay back the monthly instalment.*	Problem statement
**Counsellor**	*Would you like to tell me more about it?*	
**Umesh**	*Two years back I decided to start a tourist vehicle business so I took a loan from the local money lender as it was really hard for me to get a loan from the bank.*	Description of problem
**Counsellor**	*Hmm and what happened next*	Counsellor asks for elaboration
**Umesh**	*For one year the business was going well and I was able to pay the monthly instalments on time but then my father had a heart attack and I had to spend money on his treatment so I was not able to pay three instalments. My business also suffered as I have to spend lot of time with him in the hospital.*	
**Counsellor**	*Hmm Hmm*	Minimal encourager
**Umesh**	*As I could not manage to pay back the instalments the money lender started harassing me.*	Description of problem
**Counsellor**	*And how is that linked to your drinking?*	Counsellor tries to explore the link between the problem and drinking
**Umesh**	*I used to become very tense and upset when they came to my house to ask for money and then gradually I started drinking to escape that tension.*	Description of problem
**Counsellor**	*Did it help or worsen the problem?*	Counsellor tries to explore the pros and cons of the action
**Umesh**	*Actually each time I drink I feel numb for a little while, but then the stress returns or even worsens due to drinking.*	
**Counsellor**	*You just said the stress worsens due to drinking? Can you please tell me a bit more about this?*	Counsellor asks for further elaboration

**Umesh**	*Actually what happens when I drink is that many times I do not go to work the next day, and then the financial problem worsens even more?*	
**Counsellor**	*So what you seem to be telling me is that drinking is not working for you as a solution to your financial problem – and that it is actually making it worse – is that right?*	Counsellor makes a reflection to summarise patient's problem
**Umesh**	*Yes that's true. But when I get stressed due to financial problems I do start drinking. I just wish that I could manage my financial problems without drinking!*	Patient makes problem statement and starts thinking about solutions
**Counsellor**	*OK Umesh – I think that this is really good! You have been able to define your problem clearly and also you tell me that you want to come out of it. Have you ever thought about alternative ways to manage your financial problems apart from drinking?*	Counsellor encourages patient's effort and invites the patient to generate possible solutions
**Umesh**	*Actually sometimes it happens that when I have financial stress and still I have to go for work then I tell myself that drinking is not a solution to my problem. Sometimes, when I tell myself that, I DO manage to avoid drinking – but not too often!*	Patents starts thinking about solutions
**Counsellor**	*That's really great to know that you have managed not to drink at times, in spite of financial stress. When we tell ourselves things like this, we often describe this as 'self-talk' – do you think that you could use this 'self-talk' more often to avoid drinking when you are stressed?*	Counsellor encourages use of effective self-help strategy
**Umesh**	*Yes sure*	
**Counsellor**	*Are there any other ways you can think of for avoiding drinking due to financial problems?*	Counsellor continues efforts to generate alternative solutions to the problem
**Umesh**	*Actually I was thinking that - apart from controlling my urge to drink when I have a financial problem - I should also try to find out some ways to come out of my financial crisis.*	Patient comes with another alternative
**Counsellor**	*Umesh, it is really good that you are thinking about how to solve your root problem. So - can you think of any ways to get out of your financial problem?*	Counsellor asks for an elaboration
**Umesh**	*Actually I was thinking that I should try and take a loan from a bank or cooperative society and use that to settle the loan from the private money lender.*	Patient comes up with alternative
**Counsellor**	*What are advantages and disadvantages of that?*	Counsellor helps patient to weigh pros and cons
**Umesh**	*The advantage is obvious - that the interest rates will be much lower. Also, then I would not have to face this money lender daily. The disadvantage is that it is a bit of a complicated process and requires some documents - but I think rather than facing this money lender every day, and then drinking alcohol, it is better I spend some time and take a loan from the bank or the cooperative and settle it once for all!*	
**Counsellor**	*Ok! That sounds like a good plan! Are there any other alternatives that you can think of?*	Counsellor continues exploration for alternatives
**Umesh**	*I was thinking that I should try these two things that*	

	we decided first and then we can see what happens?	
**Counsellor**	*OK – that is a good idea. So, can you tell me what those two things are?*	Counsellor asks for summary
**Umesh**	*First, even if I have a financial problem I should tell myself that drinking is not a solution to my problem and avoid drinking; and second, I should try and take a loan from the bank and settle the loan with the private money lender.*	
**Counsellor**	*Well, this is great, Umesh! You have given us two solutions to this big problem.*<br>*But the key thing here is that you need to try these solutions out. Many times we say that we will do things and then we don't – so the big thing here Umesh is that you need to try these solutions out. And what I'd like us to do now is to go through both of these ideas again, but in a lot more detail. Is that OK? So, let's look at getting a loan from a bank or co-operative society. Which one were you thinking of – and why that one?*	

Role of SO in the problem-solving skills

If the patient agrees then the SO can play an important role in helping him with problem-solving skills. If the SO is present, then the counsellor can request the SO to share his/her views during each stage of problem solving. Input from the SO can be useful in defining the problem, generating multiple solutions for the problem, deciding on the first best solution, implementing the solution and giving feedback to patient on the progress. If there is any difference of opinion between the patient and his SO then it is responsibility of the counsellor to try and build a consensus between them about the issue. If the difference of opinion still persists then as a starting point we should give more importance to the patient's opinion.

SUMMARY

- Patients with harmful drinking often face various problems in their daily life
- Difficulties in solving these problems can be one of the important reasons for drinking alcohol
- Systematically working with the patient to help him identify and solve his problems is called 'problem solving'
- Important steps in problem solving involve identifying the problem, discussing the various solutions to that problem, selecting the most appropriate solution and applying the solution to the problem. These can all be done in one session. Clarifying whether this solution has worked, and (if it has not) understanding why, and dealing with those reasons, needs to be done at a later time - either by us in future sessions or by the patient (and his SO) if we do not meet again.

2. Drink Refusal Skills

Learning Objectives

- What are drink refusal skills and why do we need to know about them?
- What are the situations where a patient needs drink refusal skills?
- How to help a patient develop and use drink refusal skills?

CONTENT

WHAT ARE DRINK REFUSAL SKILLS?

Drink refusal skills are skills that a patient uses to say 'no' when offered or tempted by a drink.

WHY DO WE NEED TO KNOW ABOUT DRINK REFUSAL SKILLS?

Patients with harmful drinking frequently face situations where they are offered a drink. If a patient has stopped or reduced his drinking it is important for him to be prepared to handle such situations. If he does not know how to handle such situations (or to 'cope' with them) then this may lead to relapse or increased drinking. One of the skills used in such situations is called 'drink refusal skills'.

WHAT ARE THE SITUATIONS WHEN ONE NEEDS DRINK REFUSAL SKILLS

Basically, any situation where a patient is offered a drink – sometimes the patient will feel pressurised into accepting a drink, other times they will feel embarrassed in refusing, or feel that they will 'lose face', or possibly offend someone by refusing. Typical situations include:

- Attending a party (either alone or with friends or family)
- Friends or family members who drink visiting the patient at home
- Visit to a bar or a restaurant where alcohol is being served or where the waiter asks the patient what he would like to drink

Many times during the course of the assessment patients and their SOs share with the counsellor their history of drinking with friends or family members. Patients might also share their inability to say 'no' to a family member or friend as being one of the important challenges they face in achieving either stopping or controlling their drinking

An example of such a situation is:

Patient:

'*I do not drink daily. I drink only on Saturday and Sunday when I am with my friends. Many times it happens that I actually do not want to drink but when friends get together and offer me a drink I just cannot say no to them.*'

HOW TO HELP A PATIENT TO DEVELOP AND USE DRINK REFUSAL SKILLS

Most of our patients will have had the experience of saying 'no' to an offer of a drink at some time or another. We should always make it a point to ask our patient about their past experiences of successfully saying 'no' to an offer of a drink as a starting point of any discussion of drink refusal skills. The ways of refusing that they already use are part of their current 'coping skills'. The flow chart below summarises the key steps to be followed in drink refusal skills.

Because many patients tell us that it is relatively easy to say 'no' to an unknown person such as a bearer/waiter at a restaurant compared to saying 'no' to a friend or family member, it is useful to ask about both those situations. (The patient often thinks that he will lose the friend if he says 'no' to his offer of a drink.)

'I would like to know about all the different things that you have tried or think that you will try, if somebody offers you a drink which you don't want? What have you said if the person who asks is someone who you don't know, like a waiter in a restaurant? And what have you said if it is a friend or relative who you have often drank with in the past?'

If the patient cannot come up with an answer then we should ask the patient's permission to suggest certain strategies which other patients in similar situations have found useful:

'Will it be helpful if I suggest certain things which other patients have found useful when they are offered a drink?'

If the patient says yes then we can suggest the following:

'If somebody offers you a drink then you can use any of these responses:

- *No, thank you*
- *No, I am recovering from a drinking problem, so I am not drinking any more*
- *The doctor has told me not to drink as I have health problems*
- *I wouldn't mind having a fruit juice/cold drink or coffee instead*
- *I have problems with drinking so it will be really helpful if you don't make such offers to me in the future*

If the person still keeps on insisting on the patient having a drink it is useful to change the topic.

If even after changing the topic the person still keeps on insisting on the patient having a drink, it is useful sometimes to leave the company of the person or the place.

It is also important to discuss with the patient the importance of

- Maintaining eye contact with the person who offers you the drink when you say 'no'
- Speaking firmly and convincingly. The expression and tone should indicate that the patient is serious

Many patients think that saying 'no' to drinks will be considered by the friend as 'no' to the friendship. It is helpful to explain to the patient that saying 'no' to drinks and saying 'no' to friendship are two totally different things. The patient can make it obvious by saying

'Though I am saying no to the drink I want to tell you that I value your friendship very much.'

(You can use the hand out '**Saying 'No' To Drinks**' on pg 14 of the patient booklet when explaining to the patient the various situations in which his friends might force him to drink and also the various ways in which he can refuse them.)

Note: It is strongly recommended to practice these skills with the patient in a role play where the counsellor takes up the role of a person offering the drink and the patient tries to use the skill of saying 'no'. The script below demonstrates how drink refusal skills can be practised with the patient.

Script 2

Who said?	What did they say?	Comment
**Counsellor**	*Last time when we met we decided that we would discuss your difficulty in saying 'no' to your friends acting as an important hurdle for you in stopping your drinking – is that right?*	Counsellor reviews the discussion from last session
**Uday**	*Yes, that is true. It will be really helpful for you to help me out with handling this pressure from my friends.*	
**Counsellor**	*Can you please tell me in detail what exactly happens when you meet with these friends?*	
**Uday**	*We are a group of four friends and all of us work for different companies. Saturday and Sunday are the only two days when we can come together and have some fun. As all of my friends drink, then they also offer me a drink.*	Patient describes the situation
**Counsellor**	*Ok and what happens next?*	Counsellor asks for details
**Uday**	*Many times I try and say 'no' to them but then they insist on me having drinks.*	
**Counsellor**	*Tell me more about how they insist?*	Counsellor asks for elaboration
**Uday**	*If I say that I don't want to drink then they keep on asking me 'why you are not drinking?' and keep on saying that I should at least have some beer. And if I still deny then they keep on telling me that I am spoiling their party and all.*	Patient comes up with the difficulties in handling peer influence
**Counsellor**	*So what happens next?*	
**Uday**	*I think that why should I spoil their party so most of the times I end up drinking.*	
**Counsellor**	*Have you ever thought of any other ways of handling this situation?*	Counsellor asks for any other possible solutions
**Uday**	*Sometimes I feel that I should just stop going out with them but they are the only friends I have and the idea of losing their friendship makes me even more worried.*	Patients shares his worries about losing friends
**Counsellor**	*Ok so that is not like a reasonable alternative?*	
**Uday**	*Yes*	
**Counsellor**	*Can you think of anything else?*	

**Uday**	*Not really. Can you please suggest some alternatives to me?*	
**Counsellor**	*If you want I can definitely share with you some things which are known to be effective in such situation and some of the things which other patients have found useful in similar situations?*	
**Uday**	*Please I am eager to listen to them!*	
**Counsellor**	*Rather than me just telling you what those things are – why don't we try it out? Let's try it where you pretend to be the friend who pressurises you. You try and put some pressure on me to have a drink and I will pretend to be you, and suggest some things that you could say. How does that sound?*	Counsellor recommends use of role play
**Uday**	*That sounds great*	
**Counsellor**	*Ok let's start then*	
**Uday (playing role of friend)**	*Come on, have a drink.*	
**Counsellor (playing role of Uday)**	*No thanks! (with firmness in voice)*	Demonstration of saying 'no' with firmness
**Uday (playing role of friend)**	*Come on dude, it's just a beer*	
**Counsellor (playing role of Uday)**	*No thanks. I don't want to have a beer (again with firmness in voice). It is as I explained to you – I have decided not to drink alcohol anymore!*	Says 'no' with more firmness
**Uday (playing role of friend)**	*Oh you are spoiling our party*	
**Counsellor (playing role of Uday)**	*I'm sorry if you think that - but I have decided that I am not going to have a drink. But I also think that we can still have fun even if I am not drinking!*	Insists on not having drink and does not enter into argument but also firmly states about possibility of fun without drinks
**Uday (playing role of friend)**	*Oh man you have started lecturing us now*	
**Counsellor (playing role of Uday)**	*No I'm not - I am just saying that I won't have a drink and that we can still enjoy the party.*	Avoids going into argumentation
**Uday (playing role of friend)**	*You are insulting our friendship!*	
**Counsellor (playing role of Uday)**	*I am sorry if you feel that way but I think I am saying 'no' to have a drink, not any to our friendship. It is ridiculous to say that the only way we can be friends is if I drink alcohol! I certainly want to still be your friend – I just don't want to drink!*	Makes separation between drinking and friendship
**Uday (playing role of friend)**	*So you are determined that you do not want to have a drink*	
**Counsellor (playing role of Uday)**	*Yes absolutely but I want to repeat that does not mean a 'no' to our friendship*	Restates his decision with firmness
**Uday (playing role of friend)**	*Ok at least have a soft drink then*	
**Counsellor (playing role of**	*Yes, that's fine – I'd very much like to have a soft drink for sure*	

**Uday)**		
**Counsellor (back in his original role**	*Ok Uday so how did it go?*	
**Uday (Back in original role)**	*You handled it very well and I can also try to use similar tactics when they insist with me for a drink.*	
**Counsellor**	*And maybe an even better way to handle it is to start off – even before getting to the bar – by explaining that you have decided not to drink alcohol any more, and maybe why you have taken that decision. If your friends understand that you have stopped because it is causing you problems, then maybe they will not push you so much to drink – as they will realise that this might cause you harm.*<br><br>*OK – now we have tried it this way, can we try it the other way – this time I'll pretend to be the friend, trying to get you to have a drink, and you pretend to be you, using these ideas we just went through.*	

In the script above, the counsellor enacts a scenario of a friend pressurising the patient to drink and the patient spontaneously generates drink refusal statements as he goes along. An alternative strategy is to first explain all these different 'drink refusal skills' to the patient and then do the practice with the patient being himself and the counsellor playing the role of the friend. Often it is good to do both types of role plays, as shown at the very end of the example above – get the patient first to play the friend (or family member etc. - whoever he feels pressurised by), and then, once he has heard a range of ways of responding, to get the patient to be himself with us trying to pressurise him to take a drink, and him practicing these ways of refusing.
It is also important to get the patient to practice this outside of the session: with his SO or in front of the mirror.

Role of SO in drink refusal skills
The SO can play an important role in helping the patient to work on the problem with handling peer influences. The SO can help the patient in identifying potential places where drink refusal skills may be necessary. The patient can also practise the role play of drink refusal skills with their SO. If the patient agrees, then the SO can also remind the patient about being extra careful when there is chance of being subjected to peer influence for drinking (e.g. attending a party). Sometimes the SO can actually accompany the patient to such places, based on mutual convenience

SUMMARY

- Patients with harmful drinking frequently face situations where they are offered a drink
- If he is not prepared beforehand to handle such situations then this may lead to a relapse or increased drinking
- Visits of friends who drink, going to a party, meeting up with friends who drink, visiting a hotel or a restaurant where drinks are served, are all situations where patients may require these skills
- There are several ways of saying 'no' to drinks – and saying 'no' to a drink does not mean 'no' to a friendship

3. Handling An Urge To Drink

Learning Objectives

- What is an urge to drink?
- When does the urge to drink happen?
- How to work with our patient to help him handle the urge to drink?

CONTENT

WHAT IS AN URGE TO DRINK?

Many people whose drinking has become harmful tell us that they get 'urges' to drink. These are thoughts or feelings that push them to want to drink alcohol. Drinking-related thoughts and feelings may keep returning to our patient's mind for quite a long time after he has stopped drinking. When these urges become very intense and repetitive they are known as cravings.

WHEN DOES THE URGE TO DRINK HAPPEN?

The drinking-related urge can be triggered by:

- Internal experiences happening in the mind of the patient like memories of a party; and/or
- External factors like a visit to a place where the patient used to have drinks

It is helpful to know from your patient some details about both the internal and external triggers which lead to an urge to drink.

'*I would like to know about the things that make you feel like drinking*'

Patients describe their urge for drinking in different ways. The following are certain expressions used to describe drinking urges

'It (alcohol) calls me'
'I start to get the smell of it (alcohol)'
'I just can't get it out of my head'
'My mind says that I need it now'

Drinking-related urges are generally more frequent and intense during the first three to six months of stopping drinking, but they can occur even after having stopped for long periods.

HOW TO WORK WITH A PATIENT TO HELP THEM HANDLE THE URGE TO DRINK

It is always helpful to ask the patient about the ways in which he has handled the urge to drink in the past, as such skills could be very useful to practice in the future. These ways of handling urges to drink are part of our patient's existing 'coping skills' [see *Appendix 1*]. Each individual has his own methods of handling urges to drink and it is a useful strategy to ask him to elaborate on each one of them. It is also helpful to ask the patient how they can use these same coping skills if they have an urge to drink in the future.

If the patient does not come up with any coping skills to handle his urge to drink then the counsellor can ask the patient's permission to offer him information about coping skills which other patients have found helpful. For example,

'Is it ok if I suggest to you certain methods used by other people facing a similar problem of handling these urges to drink?'

If the patient agrees then we can offer him this list – the examples show the collaborative way in which this advice can be offered. As you can see, there are a lot of ideas here that we can use to help our patients deal with urges to drink.

<u>Getting involved in non-drinking activities</u>

Counsellor:

'Many people find that if they can distract themselves by getting involved in non-drinking activities, their urges to drink get much less. The sorts of distractions that other patients have used include walking, or physical activities like gardening. What distracting activities can you think of which might help when you have an urge to drink?'

Examples of other activities which other patients find useful to handle an urge to drink are:

- Preparing a meal or snack
- Going to visit a non-drinking friend
- Going to the cinema or watching TV
- Writing about one's thoughts and feelings
- Reading

Going to a 'safe' place

Counsellor:

'Many people find it useful to go to a place where generally drinking is not accepted. Places where other patients have gone include a non-drinking friend's house, a temple/church/mosque, or a library. Can you think of a 'safe place' where drinking is not acceptable that you could go to when you feel these urges to drink?'

Talking about the urge to drink

Counsellor:

'Lots of people find it useful to share their thoughts about drinking with a friend or a family member. It helps to distract them from the urge to drink and also to gets some help and support from them. Is there someone who you could talk to about these urges when you get them, who might help or support you?'

Realise that the urge is time limited

Counsellor:

'Urges to drink usually last for only a limited time. Some people find that if they make up their mind to withstand the urge for 5 minutes, they can. And if the urge does not go away, they decide to withstand it for another 5 minutes. Many people simply keep on 'deciding to withstand the urge' for repeated 5-minute periods until the urge simply disappears. What do you think – is this 'resisting for a short period' something that might work for you?'

Facing the urge

Counsellor:

'*Sometimes it is helpful to think about the urge for drinking as a wave in the sea. As the wave in the sea reaches its peak and then subsides, many times the urge to drink also reaches its peak and subsides on its own over a short period of time. Rather than resisting it or acting on it, sometimes it is useful just allow it to subside on its own. What do you think – would you like to give this a try?'*

Use of substitutes

Counsellor:

'*Some people find it useful to use substitutes like their favourite food or a non-alcoholic drink to cope with the urge to drink. Do you think that this might work for you?'*

Thought stopping

Counsellor:

'*Another form of distraction is 'thought stopping. Some people find it useful to do something which completely stops their 'urge to drink, by stopping their thoughts. For example, they wear a rubber band around their wrist and snap it each time they have an urge to drink. The short, sharp pain can stop the thought of drinking for a little while, giving the person a chance to reject the thought. Do you think that this might work for you?'*

Reminding about impact of drinking

Counsellor:

'A final idea is to use a 'reminder card'. Some people prepare a small reminder card and keep it in their pocket. They write on the card the reasons they had for controlling/stopping drinking and the negative impact that they have faced due to drinking (e.g. loss of job, separation from family). They refer to this card whenever they feel an urge to drink. What do you think – might this work for you?'

An example of a reminder card is as follows:

Reminder Card

Whenever I get a strong desire to drink, I will do the following things as I know these things have helped me in the past to overcome my desire for drinking:

- Call my wife and talk to her about my thoughts
- Eat something immediately
- Go to a safe place (like coming back home or going to the temple
- Remind myself of past times when I have managed to control my drinking desire
- Remind myself of the ill effects that my drinking is having (or could have) on my life, like losing my job and the impact on my children
- Remind myself of my children

Script

Who said it?	What did they say?	Comment
**Counsellor**	*Last time when we met we decided to discuss what can be done to handle drinking-related urges in this session. Will that still be useful for you? Ok then, can you tell me of a time where you found it difficult to control your drinking urge?*	Counsellor reviews the discussion in last session and sets the agenda for this session based on that
**Vinod**	*You know what happened yesterday? I was coming back from my office in a bus and I saw this big advertisement of the brand of alcohol that I used to drink and then I started thinking about alcohol.*	Patient describing what led to his drinking urge
**Counsellor**	*Hmm Hmm. Can you tell me a bit more about that?*<br>*OR*<br>*And what happened next?*	Counsellor asking for elaboration
**Vinod**	*I had this struggle in my mind about whether I should drink or not - but somehow I managed to take those thoughts out of my mind.*	Struggle in mind
**Counsellor**	*It’s really great to know that you managed to overcome these thoughts. Congratulations! Can you please tell me a bit more about how you did this?*	Affirmation
**Vinod**	*I reminded myself of the discussions we had about the ill effects of alcohol and I also reminded myself of the promise I have made to my wife about not drinking again*	Remembering about the ill effects and promises to Significant Other
**Counsellor**	*Well done – it sounds as if these ways of dealing with it worked for you yesterday – that is really good.*	Affirmation
**Vinod**	*Yes – but are there many other things that I could have done? These things worked yesterday, but they may not work the next time!*	
**Counsellor**	*You want me to suggest a few things to you?*	
**Vinod**	*Yes it will be really helpful.*	
**Counsellor**	*Ok, I’ll explain a few other things which other patients find*	Distraction activities

	useful in such a situation.<br><br>*Some patients find it useful to distract their mind from drinking-related thoughts by getting engaged in activities like reading, writing or listening to music?*	
**Vinod**	*This is a great suggestion. I like to listen to music. I can try this if I have such thoughts again.*	
**Counsellor**	*So now you know three things that you can do when you have drink-related thoughts again?*	
**Vinod**	*Yes that's true*	
**Counsellor**	*Could you tell me again what they are?*	
**Vinod**	*If I have a drinking urge again then I will think of the ill effects of the alcohol, and the promise that I have given to my wife, and the other thing I can do is to get involved in some other activity like music.*	Summary
**Counsellor**	*Do you want to know about some more skills that you might be able to use in such a scenario?*	
**Vinod**	*I will try these first and if I have any problems then I will come back to you.*	

Note that we can go on suggesting more activities from the list above if our patient finds it useful.

ROLE OF SO IN HANDLING URGE TO DRINK:

The SO can play a limited but important role in helping the patient to identify and deal with urges to drink. The SO can help the patient to identify the cues which trigger craving, and in finding out possible ways of dealing with the urge to drink. If the patient wants, then his SO can also help in handling the urge to drink by getting involved in activities like accompanying the patient to certain places, keeping the patient engaged in conversation, etc.

SUMMARY

- An urge to drink is either a strong thought or a strong feeling that the patient wants to drink alcohol
- Triggers for drinking urges can be either internal (e.g. the memory of a party) or external (e.g. visiting a place where the patient used to drink)
- Methods of handling drinking urges include: distraction (getting involved in non-drinking activities); going to place where drinking is not possible; talking about the urge with an understanding friend or relative; ignoring the urge and letting it pass with time; 'thought stopping'; and reminding oneself about the impact of drinking or the promises made to change behaviour

4. Handling Your Emotions

Learning Objectives

- What is the role of emotions (both negative and positive) in making it difficult for patients to implement their change plan?
- How can we help our patients to handle their emotions so that the emotions don't push them into drinking?

CONTENT

WHAT IS ROLE OF EMOTIONS IN MAKING IT DIFFICULT FOR PATIENTS TO IMPLEMENT THEIR CHANGE PLAN?

Emotions often work as internal triggers which can lead to our patients failing to implement their change plan – failing to stop or to control their drinking. Often these are negative emotions, but sometimes positive emotions can also trigger the same failure. It is easy to see how this can happen:

- A patient may feel angry with his wife and may decide, in his anger, to go out drinking ('*I have been trying so hard, and she does not appreciate me – I'll have a drink to show her that she should treat me better!*')
- Another patient may feel very happy and wish to celebrate, and may feel that having a drink to celebrate is the right thing to do ('*My son has just been born – I'll buy my friends a drink to celebrate and I'll have one as well!*')

Some of the emotions which can trigger such a failure to implement a Change Plan are:

**Negative**	**Positive**
Anger	Happiness
Sadness	Excitement
Anxiety/worry	Celebration
Guilt	
Boredom	
Stress	

During the course of our assessment, the patient might describe details of the association of his mood with his drinking ('*I drink to make myself feel better; I feel good when I drink when I go out with friends, and so I drink more*'). It is important to get details of these issues and write them down in our notes, and then finish the rest of the assessment, before coming back to these issues again when we work with the patient on the Change Plan Worksheet **(**pgs 15 and pg 21 of the patient booklet).

How to learn not to deal with negative emotions by, and not to associate positive emotions with, drinking

Step 1: Recognise your emotion accurately

If the patient is literate, asking him to keep a daily record of his important emotions is one helpful way to help the patient identify his emotions.

Step 2: Identify the thoughts behind the emotion

Emotions are often a reaction to situations we encounter in our daily lives and our thoughts work as an intermediate step between the situation and our feelings.

Situation → Thinking → Emotions

Think of two men, Umesh and Ramesh, who both get fired from their jobs. Umesh becomes terribly sad and stays at home feeling low, but Ramesh remains positive about it and decides to immediately

search for another job. Though both, Umesh and Ramesh, have been faced with the same situation, they end up in different emotional states because of different thinking patterns:

For Umesh

Losing Job - →	Negative thinking →	Negative emotions (Sadness)

For Ramesh

Losing Job →	Positive thinking →	Optimistic emotions

Step 3: Identify the consequences of the negative thoughts

'I've lost my job, I'm useless, I'm sad, and I'll drink to feel better.'

OR

'I've lost my job, I didn't like it anyway and now I'll get another, I'm pleased at how optimistic and positive I feel; I'll drink to celebrate.'

Note that difficulties in handling both negative and positive emotions could work as an important barrier to achieving the patient's drinking goal. However, patients more commonly drink as a consequence of **(**or to help them cope with**)** negative emotions than they do to celebrate positive ones.

Step 4: Challenging negative thoughts and replacing them with the positive ones

Once our patients start identifying the thoughts behind their behaviour, the next step is helping them to replace negative with positive ones. The most important step to be followed for replacing negative thoughts with positive ones is called 'challenging the thoughts'. In this process of challenging the thought we help our patient to examine the usefulness of his current thinking pattern in achieving his desired goals. Once our patient realises that the current negative thinking pattern is not helpful in achieving his desired goals, he becomes motivated to replace the current negative thinking pattern with a more positive one.

The example below helps us to understand how to challenge a negative thought with a positive one

Example

Our patient describes an incident (a quarrel at home) which led to him going out drinking.

Step 1: We get our patient to recognise his emotion accurately

In discussion, our patient recognises that the emotion he was feeling was a mixture of upset and anger.

**Incident**	**Emotion**
Quarrel at home	Upset & Anger

Step 2: We get the patient to identify the thoughts behind these emotions

**Incident**	**Emotion**	**Negative thought**
Quarrel at Home	Upset & Anger	I have to take a drink to feel better

Step 3: We get the patient to identify the consequences of these negative thoughts

**Incident**	**Emotion**	**Negative thought**	**Consequence**
Quarrel at Home	Upset & Anger	I have to take a drink to feel better	Drinking alcohol

Step 4: Challenging negative thoughts and replacing them with the positive ones

Here we will get our patient to challenge his thought processes by asking him questions like:

'*Do you really need a drink to control your feelings?*'

Or

'*Explain to me how drinking when you feel upset and angry helps you to feel better?*' or '*Is drinking helping you in any way to solve the problem at home?*'

Once the patient answers such a question negatively then we can work with the patient to help him to choose more positive thinking responses, such as:

‘*I can control my emotions without taking the help of drinks*’ Or ‘*It might be more helpful if I tried to solve the problem at home, instead of going drinking*’ or ‘*I certainly know that if I go drinking, I will only make things worse at home*’
Once our patient starts changing his negative thoughts into more positive ones, then the negative consequences also start changing in positive way.
The script below demonstrates the skills for handling difficult emotions.

Script
Akash is a 23-year-old unmarried man. He has a strained relationship with his father. Whenever he has a difference of opinion with his father, Akash becomes very upset and angry. This often leads him to go drinking. Akash has expressed his willingness to work on his emotion-handling skills in the second session.

Who said?	What did they say?	Comments
**Counsellor**	*We decided to discuss your emotion handling skills in this session - right Akash?*	Counsellor reviews discussion from last session
**Akash**	*Yes that is really important for me. I have a really bad relationship with my father. We often get into fights and most of the times after we have these fights I go and drink alcohol*	Patient describes his inability to control anger as an important reason for drinking
**Counsellor**	*Can you tell me how you feel when you have fights with your father?*	Counsellor asks for identification of feelings
**Aksah**	*I feel terribly upset when he starts scolding me about minor things.*	Patient describes the feelings
**Counsellor**	*Hmm what happens after that?*	
**Akash**	*I just cannot tolerate him scolding me for no good reason. I lose my temper and I also start thinking that he still treats me as a kid. Then I cannot hold myself back from getting angry with him even more*	Patient describes the thinking behind the feeling
**Counsellor**	*So your father scolding you for small things makes you angry and upset?*	
**Akash**	*Yes*	
**Counsellor**	*But does it happen every time when your father scolds you?*	Counsellor tries to explore different ways in which the same situation is handled by the patient
**Akash**	*Most of the times I guess. But sometimes I just ignore him thinking that he is getting old and I should not argue with him.*	
**Counsellor**	*And what happens then?*	
**Akash**	*I just get up and leave the place as that helps me to control my anger.*	
**Counsellor**	*Have you realised one thing Akash? Though the event of your father scolding you remains the same, your reaction varies. Sometimes you become angry and at other times you don’t. What might be the reason for this?*	Counsellor helps demonstrate to Akash how the same situation can be handled in different ways
**Akash**	*No I don’t know why*	
**Counsellor**	*Can I explain to you what I think is happening?*	
**Akash**	*Yes sure.*	
**Counsellor**	*Generally we tend to believe that our emotions are a reaction to the situations but if that was so your reaction to the situation of your father scolding you would be the same every time he scolds you. But as you*	Counsellor explains Aksah relationship between the situation and feelings

	yourself mentioned this is not the case – right?	
**Akash**	*Right*	
**Counsellor**	*Your reactions to the situation changed in the second instance because you changed your thinking. The first time, you were thinking that your father is treating you as a kid and hence you had feelings of anger. But the second time you were thinking that your father is getting old so you should not take his comments too seriously and so you were able to ignore them and control your anger.*	Counsellor explains to Akash how different ways of thinking can lead to different emotions in the same situation
**Akash**	*Yes that's right*	
**Counsellor**	*So can you summarise what we have discussed just now?*	Summary
**Akash**	*Whatever the situation is, I should think positively and not allow my emotions to take control of my life.*	Patient summarises emotional control skills
**Counsellor**	*That is a great summary. So - what will you do when your father starts scolding you again?*	Affirmation by counsellor
**Akash**	*I will just think that he is getting old and I should not get into an argument with him.*	
**Counsellor**	*Great, hopefully if you control your anger, then you may not think of going for a drink?*	Affirmation, and helping patient see the connection with drinking
**Akash**	*That's true - I generally do not drink if I am not angry.*	
**Counsellor**	*So how about trying that over the next week?*	
**Akash**	*Yes, sure I will*	

Role of SO in handling emotions

Sometimes patients think that particular reactions by a family member are the reason for their emotional disturbance (e.g. the example above, where Aksah thinks that his emotional disturbance is due to his father scolding him). The skills used in the example above are focused on changing Akash's emotional reaction to scolding by his father. Another way we can address this problem is by working with Akash's father on improving his communication pattern. In this case we can work with Akash's father to express his concerns about Akash without scolding him. In this way, if the SO is contributing to our patient's emotional disturbance, then we can work with their SO to modify his / her behaviour.

We can also ask both the patient and his SO about the possible roles that the SO can play in handling his emotions and then work with the patient and his SO on the specific areas they suggest.

SUMMARY

- If not handled properly, emotions (especially negative ones) can act as triggers for a failure to implement a change plan
- Anger, sadness, restlessness and guilt are important negative emotions, and excitement and celebration are important positive emotions, all of which can trigger a failure to implement a change plan
- Four important steps for the effective management of emotions are: identifying the emotions, identifying the thoughts behind the emotions, clarifying the consequences – the link that leads to emotion-thought-drinking, and changing negative thoughts into more positive thoughts

Role of the SO in phase two

We have given many examples during our description of these four sets of specific skills of how an SO can be helpfully involved in Phase Two.

As well as these, if the SO was present in the first session and he /she has agreed to take a role in the change plan, then it is useful to review what has happened about this role in the time between two sessions and the perceived advantages and disadvantages of the role played by the SO.

If the SO is coming for the first time to the sessions then we can follow a similar process followed in the *Initial Phase*, of asking the patient about the possible role s/he can play in the implementation of the change plan and involve the SO in the treatment accordingly.

Ending phase two (and session two)
As with the first session, we end this session and this phase with an Action Plan and a summary.
With some patients, counselling will end after two sessions. With others, we will continue for a further one or two sessions. Which of these happens will depend on

- What the patient wants
- How far along the three phases of CAP we have got

With some patients (who are very ready to change)

- We will have quickly moved in Phase One to developing an overall drinking goal and then to developing sub-goals and a Change Plan,
- We will in Phase Two have quickly clarified potential problems and dealt with needed thinking and behavioural skills
- We will have moved into Phase Three during this second session

So we will only end our counselling at the end of our second session **i**f the patient wishes to, or if we have already covered the main aspects of lapse and relapse management (i.e., Phase Three) in this second session.
If this is to be the last session, then we will need to end the counselling in a good manner. How to do this is covered in the *Ending Phase*, in the next chapter. The last bit of this chapter, therefore, will assume that we are continuing, and hence that we are expecting our patient to come back for a third session.

Action plan and specific homework
This Action Plan gathers together all of the actions, tasks, and activities which our patient (and his SO if present) have agreed that they will do as a result of this session.
Whether or not we are ending counselling at the end of this second session or continuing to a third /fourth session, we will always need to clarify what the actions are that the patient and his SO have agreed to do.

SUMMARY
Similar to session one, session two should also end by asking the patient to give a broad summary of what has been discussed in the session; then asking the SO if they have anything to add; and then reiterating the key things ourselves. As with session one, if the patient finds it difficult to summarise the discussion, we can move on to asking the SO, and then offering our own summary. Again, the summary should emphasise 'change talk', plans for change and the patient's and the SO's perceived consequences of changing and not changing. But this summary should also go over any and all progress made in between the first session and this one, and what key skills have been looked at in the session. The summary will also reiterate the Action Plan, and any specific homework.
At the end of this, we will raise with our patient the possibility of up to two further sessions, outline what we would cover in those sessions, and find out if the patient (and his SO) wants to attend these further sessions.

Here is an example of how a summary might be worded:
'*Thank you Mr and Mrs Fernandes – I thought that was a really helpful summary. Can I just go over what I think are the main points again?*
We discussed Mrs Fernandes what you knew about your husband's drinking problem and what he had told you about his session with me last week. Then we got feedback from you Mr Fernandes about what had happened in the time between our first session and you coming to see me today. And in that, we also discussed the Action Plan that we set last week and how well you had managed to do some of the tasks that we had agreed last time. Then we looked again at the Change Plan that you had developed with me last time, Mr Fernandes, and we clarified that both you and your wife wanted the

goal that we had agreed in that change plan – that you would stop drinking entirely. We also looked at some of the sub-goals in the Change Plan and you both agreed that you still wanted these as important sub-goals – that you managed to argue less between yourselves, and that you didn't feel so pressurised, Mr Fernandes, to drink when you were with your family.

Although you had achieved a lot, Mr Fernandes, in doing what the Action Plan had suggested, there were a few things that caused you problems in the week, and we looked in a bit more detail at some of these problems. We discussed how you could refuse offers of alcohol, and how you could deal in different ways with your feelings. And we listed together and wrote down all of the things that both of you said that you would try to do differently between now and our next session, so that both of you could continue to work towards the overall goal of stopping drinking. So you, Mr Fernandes, said that you would practice refusing drinks from your family, and from others, and that you would work on dealing in different ways with your feelings so that you were not triggered into drinking. And you, Mrs Fernandes, said that you would try hard to respond in a different way (not to nag and not to argue), and to prepare food at the times we agreed, and also to deal with his family differently, as all of these things seem to be related to triggering your husband to go out drinking.

Does that seem about right? Have I missed anything?'

If the patient offers additions or changes, we need to reflect these and integrate them into our summary.

Scheduling phase three (session three):
The third session should be scheduled at a time that allows the patient to have tried out the Action Plan and any specific homework tasks. This may be within the next one to two weeks, or it may be later than this – maybe 3 or 4 weeks' time - based on the mutual convenience of the patient and the counsellor. As before, subsequent sessions can be done in the clinic, at the patient's home or over the telephone, as agreed with the patient. These different ways of delivering sessions are discussed in the CR manual.

Saying good bye
The final part of the session is saying good-bye. Briefly, we should cover these points:

- The possible ways, in which the patient can contact us for any urgent matters, including changing the appointment
- A statement that if the patient misses his appointment, we will get in touch with him on the phone (if he has given us permission to do so) to arrange another appointment
- Finally, thank the patient for working with us, congratulate him on his successes so far, and bid him farewell warmly and with a reminder that we are looking forward to our next meeting

SUMMARY
There are a number of tasks that we have in the middle phase of CAP.

- Set an agenda
- Review progress on the Action Plan and any specific homework
- Work on the target behaviour by looking at any of the thinking / behavioural skills that are appropriate to this patient:
    - Problem Solving
    - Drink Refusal skills
    - Handling Emotions
    - Handling Cravings and Drinking Urges
- Elicit commitment and further develop our patient's motivation for change, as needed
- End the session well (as we did in the first session)

Chapter 7

CAP Session-By-Session Guide

C. Ending Phase (Sessions Three and Four)

Preventing and Dealing with Relapse, and Ending Well

Learning Objectives

In this chapter, we will learn:

- Discuss what has happened in relation to the patient's drinking in between the second session and this present one
- Look again at the change plan and clarify whether this is still what the patient and his SO want to do
- Work with the patient on relapse prevention and management
- End counselling well by reviewing the skills and strategies that the patient has learnt, clarifying any skills or information that is not clear, and reinforcing the patient's motivation to use these skills and strategies across other areas in his life

CONTENT

This Ending Phase (Phase Three) is most commonly undertaken in Session Three and sometimes Session Four, although for some patients we will get onto some of the tasks outlining in this chapter in the second session (and for some especially motivated and aware patients, we may complete all of our counselling in only one session).

There are certain things contained in previous chapters which are also important at the start of and throughout Phase Three. Because we have covered them already, we will simply list them here, but if you are in any doubt, go back to look at each relevant section.

- We still have the key overarching task – to develop the patient's motivation (engagement, working together, developing and encouraging change talk, identifying and discouraging sustained talk)
- We still want, as far as possible and as long as the patient agrees to it, to involve a significant other (SO) in the treatment to help the patient achieve his goals

And we still have the basic structure which we adhere to:

We (the patient, the SO (if present) and us as the counsellor) will

- Greet, and set an agenda between us and the patient (and their SO)
- Review progress on the Action Plan and specific homework since the last session
- Work on the target behaviour(s) (which in this session will mean that we will a) look again, if needed, at the four key thinking and behavioural skills we looked at last time, and then b) look at relapse prevention and management)
- Plan Action Plan and specific homework
- Summarise, and either end our counselling well, or book a last session

Once we have set the agenda for the third or fourth session and reviewed progress on the Action Plan (AP) and any specific homework since the last session with the patient and his SO, most of the rest of this third phase will be dedicated to working with the patient and his SO (if accompanying) on preventing lapses and relapses (and managing them if they occur), and then on Ending Well.

GREETING, AND SETTING THE AGENDA

Counsellor:

'It is good to see you again Mr and Mrs Kamat. It has been about three weeks since we met, so I am sure that we have a lot to catch up on. As you know, we start each session by planning out an agenda – what we are going to cover - and you may remember that I said that I'd start this next session by asking you what you'd like us to discuss today. Remember – I just want to know what the things are, not to have the discussion yet – I am just trying to get clear what we will need to cover today. – So, Mr Kamat, can we start with you – what would you like us to discuss today?'

As usual, once we have got items from Mr Kamat, we'll then ask Mrs Kamat for her items (all the time ensuring that we don't get drawn into discussing these items, just clarifying what they are); and then we add our own items. We may say:

'OK – what you have both added to the agenda is great and we will certainly discuss all of these. Can I add my own things too? One thing I want to discuss with both of you today is about relapse. I'll explain later what I mean about relapse, but I'd like us to discuss what we can do to avoid it, and if it happens, what to do about that'

Once we have got the agenda items, we will need to arrange them so that we cover things in a logical order, and then feed back to our patient (and his SO) what we think the agenda for the session looks like:

'OK, so let me go over what we are going to cover today. We will start with hearing from you, Mr Kamat, about how things have gone over the last few weeks, both in terms of your drinking goal and in terms of the other goals that you had and that we listed on the Change Plan last time. You'll remember that you went away with an Action Plan which summarised the things that you were going to try out to help you with these goals, and there were a couple of specific homework tasks as well

- *You were going to keep a Drinking Diary*

- *And you were going to get information from the panchayat about what you would need to do to open up the small general store that you and Mrs Kamat were talking about doing, so as to give you something to do*

While you are telling me how things have gone, you Mrs Kamat can also come in and tell us if there are things that you want to say about how things have been going – either where you agree, or where you think that there is a different way of putting things.

Then, from what you both tell me, we may want to look again at some of the skills that we discussed last time – you'll remember that we discussed:

- *A method of solving problems*
- *And about how to refuse offers of drinks,*
- *And about how to handle urges to drink,*
- *And about what to do about our emotions when they threaten to push us back to drinking.*

And then I think that it is important that we discuss lapse and relapse.

Then at the end, we will work out what you have both agreed that you will do, to ensure that your goals are achieved; and we'll see if you think that we need to meet again, or if what we have done will be sufficient.

REVIEWING PROGRESS ON THE ACTION PLAN AND SPECIFIC HOMEWORK

After setting the agenda for the session with the patient and SO (if present) we can then move on to reviewing the progress made since our last session by the patient. We will want to especially look at the tasks that we agreed in the change plan worksheet and the Action Plan last session.

If Phase Three takes place in the third or fourth session, we will still need to review progress in the Action Plan. We do not want a highly detailed account of everything which has occurred in Mr and Mrs Kamat's lives since we last saw them! But – we do want to know a lot of detail about any significant events which

- Either showed Mr Kamat successfully implementing the actions that we discussed last time
- Or showed him having problems with implementing these actions

Remember

- If he has been successful in doing things, this needs affirmation – praise
- If he has been unsuccessful, we need to
  a) See if there is anything there that we can re-frame as a success ('*I can see that you think that this went really badly, but actually, when you did 'x', I think that showed that you were doing what we planned, even if it was difficult to sustain that*')
  b) Try to more strongly re-frame it as an important learning opportunity and then
  c) Examine it in some depth, clarify why it was unsuccessful, what can be learnt from it, and what extra skills or extra practice he needs so that he will be more successful next time

Once we have gone through these steps, we can move into the main issue for this Ending Phase – working on relapse.

RELAPSE PREVENTION AND MANAGEMENT

What is lapse and relapse?

A lapse [see *Appendix 1*] is when a patient starts drinking again after a period of planned complete stopping, or when a patient who has been in control of his drinking starts drinking a larger amount and/or more frequently than specified in his controlled drinking plan.

When a patient has a lapse, if he stops drinking, or controls his drinking again, quite speedily, then the lapse remains as a lapse. But if he continues drinking in this way, then his lapse turns into a relapse [see *Appendix 1*].

So 'a lapse' means that there has been one single occasion when the patient has not kept to his plan of not drinking or controlled drinking. If the patient then moves to drinking more than just on that one 'lapse' occasion, or drinks more often than planned, or if each time (or most times) he drinks he drinks more than he had planned, then we'd call this 'a relapse' – not just one occasion but a series of occasions where he has not kept to our plan.

A relapse is not just an event of drinking alcohol again, but is a process which starts with thoughts about drinking alcohol, goes on to actual drinking, and then continues so that the patient is drinking at increasing quantity and frequency.
Counsellor:

'A relapse is when someone returns to his previous way of drinking. It happens quite a lot that someone – like you Mr Kamat – changes his behaviour and either stops drinking completely or gets it under a tight control – and then things happen and he finds himself back to his old style of drinking.
I know that you have told me that this will not happen with you, and that you are really determined, and that is really excellent! But in my experience, things can happen which push people off balance - even people who are really determined. So I like to work with people to prepare them for what might happen, and make sure that they have a plan so that if things do happen which push them off balance, they know what to do. It is a little likes practicing a fire drill in a big building – one hopes that there will not be a fire, but if there IS a fire, it is much better to know what to do, as opposed to panicking and having to work out how to get out of a burning building!
So, what I'd like us to do today is to work together to see what any possible areas there might be which might push or trigger you into relapse, and then work with you on how to deal with those, if they were to happen.'

Is relapse preventable?
Yes, relapse is preventable, if we work with the patient on relapse prevention as part of our counselling. To prevent it, both we and the patient need to understand how relapse happens.
It is commonly believed that relapse occurs suddenly and without any warning signs. This is not true. The process that generally happens is

Drinking triggers → Drinking Urge → Lapse → Relapse

The first thing that happens in the process of relapse is a drinking trigger. This can be an external (e.g. having an argument with someone) or an internal trigger (e.g. feeling very low) which can start the process of thinking about drinking. As the thoughts about drinking start becoming intense and more frequent, they get converted into an urge to drink. If the urge to drink is not successfully dealt with, the person has a Lapse. If he has stopped drinking, then the lapse occurs when he takes a drink; if he has been controlling his drinking, then the lapse occurs when he starts to drink in an uncontrolled fashion.

Lapse and relapse
We have two clear tasks when we work with our patients on lapse and relapse:

- We need to try to help our patients so that they do not lapse in the first place – they do not take even one drink if they have successfully stopped completely, or they do not drink excessively or in an uncontrolled fashion even on one occasion if they have been successfully controlling their drinking
- But we also need to help them so that, if they do lapse, they do not turn that lapse into a relapse. Often a lapse involves a patient taking a single drink, or a patient drinking on a single occasion. Our first task – the bullet point above - is really important, since, if our patients can prevent a lapse, there can be no relapse. But even if a patient does lapse, this does not have to mean that he will go on to have a full relapse. One important part of relapse prevention is helping patients, if they have a lapse, to stop there and not turn a single lapse into a full relapse

So there are two sets of skills that we need to equip our patients with:

- How to prevent a first lapse (since if patients can prevent this, then they will never relapse)
- How to stop a lapse turning into a relapse

Preventing a lapse
To prevent a lapse, it is important to know about potential triggers which often lead to a lapse.

Sometimes, triggers can be situations, sometimes they can be feelings. Many such situations and feelings are common across patients, although of course some situations and feelings are specific to the particular patient.

In what ways are these triggers different to the ones we looked at when we covered '**How to Fight the Urge to Drink**' (pg 11 of the patient booklet) and 'Dealing with Emotions'?
Often the triggers are the same – the differences arise in where they occur in the process of recovery from a drinking problem, and what we do about them if they trigger a lapse.
When we start to see a patient with a drinking problem, there are always two tasks that our patients have:

- First, they have to deal with their current drinking. They have to learn what triggers their current drinking, and how to overcome those triggers so that they can stop or control their drinking
- Often, once they have done this, they feel that they have succeeded, and they do not need further help. However, this is not true! Almost all patients, after successfully stopping or successfully controlling for some time, then go on to at least have a lapse, and they usually go on to relapse! So their second set of tasks is to realise that this is a danger, and work out both how to use similar skills in a different way, and which new skills they need to learn to deal with a lapse so that it does not turn into a relapse.

In some ways, the more confident a patient is that they will never relapse, the more important it is that we work with them on how to ensure that this is true. Patients who are very worried that they will relapse will be looking out for triggers that could push them into drinking, whereas over-confident patients will be so sure that they will be OK that they will not see the pitfalls in front of them, and may fall in, if we are not able to help them to be more careful.

The triggers that can knock a patient off balance can be either external or internal:
External:

- People: Drinking friends /relatives
- Places: Bars, parties
- Things: Easy availability of alcohol, excess cash in hand
- Time: Paydays, holidays, periods of stress, week-ends

Internal

- Stress
- Anger
- Shame and guilt
- Happiness – excitement
- Boredom
- Physical pain
- Becoming over-confident: 'I don't need to worry about drink now'

Another set of potential risk factors for lapse/relapse are the seemingly irrelevant decisions. These are those decisions which appear unimportant on the surface but actually increase the likelihood that the patient will be placed in a high-risk situation that can cause a lapse/ relapse. An example of such decisions is described below:
Ramesh, who had been abstinent for several weeks, drove home from work on a night when his wife was going to be away. On the way, he turned left rather than right at an intersection so he could enjoy the 'scenic route.' On this route, he drove past a bar he had frequented in the past, where he had bought alcohol and drank with his friends. Because the weather that day was hot, he decided to stop for a glass of Coca Cola. Once in the bar, however, he decided that since his problem was with whisky, it was fine to have a beer. After two beers, he lost control and he lapsed into drinking whisky.
As we can see, the one apparently irrelevant decision that Ramesh made (of driving down the 'scenic' route) led to a lapse.

Many times one apparently irrelevant decision can lead to a chain of other apparently irrelevant decisions as we can see in the following example:

Raghu has been abstinent from alcohol for the past six months. One day he goes out for a walk. He ends up at the ATM where he decides (seemingly irrelevantly) to withdraw some money. Because he may not go past an ATM in the next few days, he takes out more money than he might need for the present day. He ends up walking through the neighbourhood where he lived before. He meets and talks to an old drinking friend (just to encourage this old friend to stop drinking). Then he goes with his friend to a bar (just to show how he can resist drinking). However once he gets there he is not able to resist and as he has extra money in his pocket he starts drinking and lapses.
At each point, Raghu could have done something different. He could have not withdrawn extra money from the ATM, he could have decided not walk in his old neighbourhood, he could have chosen not to chat with his old drinking friend and he could have chosen not to go the bar. So the lapse process occurred in stages and could have been stopped at any time prior to the lapse itself.
One of the things about these chains of decisions that lead to drinking is that they are far easier to recognise and stop at the beginning. The closer the decision-making process is to the alcohol, the more difficult it is to stop e.g. it would have been easier for Raghu to change his decision to withdraw extra money at the ATM than to not buy a drink once he got to the bar.
Another important thing to know about such seemingly irrelevant decisions is that if the patient can get himself into the habit of recognising all the small decisions he makes every day, and thinking through safe versus risky consequences for those decisions, he will be less likely to land in high-risk situations.
As we have said above, these are similar triggers to the ones that used to push our patients into drinking in the first place. The difference is how we get our patients to take action in dealing with these triggers, after they have been successful in changing their drinking.

There are three key steps to prevent a lapse

- Identify triggers
- Prevent exposure to triggers
- Cope with triggers differently

It is important for us to link this work of relapse prevention and management to the Change Plan Worksheet (pgs 15 and pg 21 of the patient booklet) where we have discussed with the patient and his SO the potential triggers which might lead him to drink again.
An example of how this can be done is as follows
Counsellor:

‘*OK Uday, as we are discussing now about how to help you not to go back to drinking again, can we have another look at the Change Plan Worksheet, where we had some initial discussions about this?*’

In this way, we can start our relapse prevention work with the potential areas of relapse identified in CPW.
Sometimes patients find it difficult to imagine the potential areas of relapse even for the CPW. Below is an example of how to handle such scenario.
Counsellor:

‘*Can you think of situations in which you may feel like drinking again?*’

Sometimes the patient gives a straight ‘no’ answer to this question!
Patient:

‘*No I am fully determined to stop drinking for my whole life now*’

If this happens then it is recommended to ask permission of the patient to offer the experiences of other patients in this regard.
Counsellor:

‘*I fully support your strong commitment not to drink at all. However, sometimes it does happen that people might relapse despite such a strong commitment. Will it be ok if I share with you some experiences of such patients?*’

If the patient agrees we can then offer him an explanation about the process of relapse described above, and then we can ask the patient about the various triggers which he may face in the future.

If the patient agrees, then we can discuss again the following techniques that we learnt in Phase Two in relation to skills required for achieving drinking-related goals:

- Drink refusal skills
- Managing emotions
- Problem solving
- Handling drinking urges

We need to help our patients understand that even if they are strongly committed to stopping drinking or to drinking in a very controlled fashion, things may happen which will throw them 'off course'. So we want to make sure that they have the skills, and have practiced them, to be able to withstand these things (internal and external) which might happen in the future.

We need to reinforce that although the way of dealing with the triggers may be the same as those we learnt earlier, the big difference is that these triggers will often take us by surprise, and people often 'find themselves' in a state of lapse/relapse almost before they realise it. So a key set of skills relates to helping our patients see the triggers in time to prevent the lapse**.**

A useful technique is to ask our patient to think of a time when he successfully exerted control in the past (either by stopping, or by having his drinking under clear control), and to examine why and how things went wrong – why and how he relapsed.

Patients often find this difficult to do. Many say that the relapse 'just happened'. This is not true. We can understand why a patient will want to believe this - the area of relapse is associated with upset, failure, disappointment –and so it is no wonder that patients want to gloss over it as quickly as possible.

But our patients must be helped to see that relapse is not something which 'just happens', but is an endpoint in a chain of decisions and events which may have started some time before the actual relapse.

For example, let us look at a person who has a relapse after attending a party on December 31. In this case the patient can easily anticipate the high risk nature of the situation. The process of relapse starts from the moment the patient takes a decision to attend the party. After the decision to attend the party there are a series of other decisions which the patient takes, such as attending the party with friends who generally drink, and not preparing enough to handle peer influence. All of these can contribute to the process of relapse. So it is our responsibility to help the patient to identify that the relapse is not something that 'just happens' but that all patients make a series of decisions which lead to the end point of relapse. This can be seen easily in the corresponding problem of dieting, where people often plan out their relapses long in advance: it is common for people going on a holiday to start dieting in order to be able to eat excessively when they arrive on holiday, the implication being they know they are going to relapse at that time.

So – how do we help people recognise these triggers soon enough so that they can take action?

- Our patients who lapse usually do something without thinking, which puts them into danger of a lapse. They take some decision which seems irrelevant, but actually exposes them to danger. So, they go to visit a brother-in-law, or they agree to go to Mumbai to get a passport, or they see a friend in town and stop for a chat, or they go to an opening of an art exhibition
- Each of these (and our patients will be able to give us examples which are relevant to them) on their own are harmless decisions and are not associated with drinking – but each puts the patient into a high-risk situation which they have not prepared for, and hence for which they do not have any coping responses prepared
- So their brother-in-law offers a drink from his new bottle of whisky; going to Mumbai means our patient has to stay overnight and he does not know anyone, and passes a bar; when chatting to the friend in town the friend suggests getting out of the sun and having a quick drink; the opening of the art exhibition has free drinks as part of the event
- Being in a high-risk situation with no coping responses – not knowing what to do or say or think – makes people feel uncertain or insecure, and one response that our patients have had in the past to these feelings is to drink alcohol

What we know is that, often, our patients will be able to withstand such pressures – but that sometimes, they will not. So the more we can help our patients work out how these apparently irrelevant decisions are actually triggers taking them into high-risk situations, the less likely they are to lapse.

Our tasks in preventing lapse then are to:

- First, help our patients see and recognise these ‘apparently irrelevant decisions’ which put them into high-risk situations, and learn how to avoid these high-risk situations (and also learn the skills needed to be able to avoid them - how to refuse invitations, or forms of words that could be used so that a chat with a friend or a brother-in-law does not turn into having to refuse a drink)
- Second, our patients need to understand that avoidance does not always work, and sometimes they will find themselves in high-risk situations. So we need to help them develop the best coping responses – either by practicing drink refusal skills or working on managing their emotions or dealing with their drinking urges. As we say above, what counts as a high risk situation will be different for different patients? For some it will be a social situation where alcohol is present; but for others it will be a life-problem – waking up feeling depressed and moody, or living with poverty and unemployment, or being in conflict with other people. Each of these situations is manageable, and coping strategies can be developed to deal with all of them - but they must be recognised in order for them to be dealt with

How to prevent a lapse (if it happens) from turning into a relapse?

So, our first task (above) is to work with our patients to help them withstand the pressure to lapse. However, we need to be aware of the fact that a lapse can happen, despite the best efforts of both the patient and his SO, and ourselves. So we also have an important responsibility to work with our patient and his SO to help them deal with a lapse, if one happens, to avoid the lapse turning into a full blown relapse.

There are two elements here:

- Helping our patients prepare, so that if they do lapse they do not allow it to turn into a relapse
- Helping our patient to work through what happened which led to a lapse, and how to prevent that happening in the future

Helping our patients prepare

Even with all the work done to ensure that a lapse does not happen, many patients do lapse. So our patients need to know what to do if they lapse, to stop the lapse from turning into a relapse. What skills do they need to enable them to stop after one (or a small number of) drink(s), or after an occasion of uncontrolled drinking? There are a number of skills, which can be divided into things the patient can say to himself, and things the patient can do.

Our patient can say a variety of things to himself and to others if he has a lapse:

- ‘*There is no necessary connection between lapse and relapse*’
- ‘*Turning a lapse into a relapse is turning a small stumble into a catastrophe*’
- ‘*What I do, now I have lapsed, is under my own control – I can decide if I go on to drink more, or to stop at this point*’
- Our patient can rehearse to himself (or to others who are with him) the advantages of remaining on the pre-planned path of not returning to excessive drinking
- Our patient can calculate the money saved through not drinking
- Our patient can think about the improved relationships with family, friends, colleagues, and so on that he has, now that he is not drinking/ controlling his drinking

Our patient can also do things. Saying things to oneself is a useful set of strategies, but these things to say often desert people’s minds just when they are needed. Many patients report that before the lapse occurred they knew exactly what to say to stop them, but when they did take that first drink, they felt panic-stricken, and could think of nothing to do except to carry on drinking.

There are a number of useful things that patients can do to prevent a slip turning into a full-scale relapse

- We can get our patients to write out (or we can write out ourselves) a set of 'reminder cards', which are sealed but which patients carry around with them and which are only opened after a slip has occurred. These cards (see Appendix 5) rehearse the sorts of 'self-talk' outlined above
- Another strategy is for the client to tell as many people as possible about his or her aspirations about drinking, and to ask people to say some of the things discussed above if they see him or her start to drink. These people could also be asked to help by not offering the client alcohol.

Helping our patient to work through what happened which led to a lapse
As well as preparing, so that the patient does not allow a lapse to turn into a relapse, the patient also needs to go over what happened when he lapsed, to improve his skills so that a similar situation does not lead to him lapsing again.

As usual, there are various steps that our patient needs to take:

1) First, he needs to return to see us, so that we can help him work out in detail what has happened and why he has either had a drink (if he has stopped altogether) or drunk in an uncontrolled fashion.

Of course, our patient could work through all of the steps outlined below without our help – but certainly for a first lapse, it is usually very useful for the patient to be supported by us while going through the steps below.
Coming back to see us sounds easy, but many patients who have had a lapse feel that they have let both themselves, and us, down by the lapse, and so often our patients will avoid following up with us. In fact, such guilt feelings can often trigger a full blown relapse. This is one reason that we tell the patient (and his SO) that it is not unusual to have a lapse and explain to the patient and his SO that it is vital that they contact us if he has a lapse, to discuss what happened, to prevent a full relapse, and to avoid the lapse happening again in the future. If the lapse happens during our CAP counselling, in the intervals between our sessions, then he needs to contact us and we will bring forward our next appointment so that we can deal with this issue. If we have already completed CAP, then he can contact us and re-refer himself.

2) Once he does come to see us, we can work with the patient (and his SO) using the following skills:
   - Get specific details of the lapse from the patient – how it occurred, whether or not it was planned, what triggered it, what ways did the patient try to cope with the trigger instead of drinking, what did he tell himself when he decided to lapse, and how was he able to stop himself after the lapse and prevent it from it becoming a full relapse
   - Depending on the details, we then need to clarify at each step what the patient could have done differently, and what he will do differently if similar situations arise again. For example, if the patient had a lapse due to his inability to handle peer influence (e.g. his feeling that friends or family were pressurising him) then the counsellor needs to work with the patient to strengthen his drink-refusal skills
   - We also need to congratulate (affirmation) the patient for not allowing the lapse to lead to a relapse
   - It is also important to discuss with the patient his feelings about the lapse and help him overcome these feelings using techniques described in the section on 'handling your emotions'

Confidence: The part it plays in lapse
One common reason for a lapse is that a patient, after successfully stopping or successfully controlling for some time, starts to feel much more confident, and starts to feel that his problem was not as serious as he had thought initially. He has a lapse, and finds that he is OK – the patient who has stopped completely finds that having a drink did not lead to him immediately returning to very heavy

drinking; the patient who is controlling found that going out with his friends and getting very drunk has not immediately led to him doing it again the next night.
In these situations we need to go back to the Change Plan that the patient worked out with us, and go over again what the reasons were that this patient decided on this drinking goal, and work out carefully with him whether he is sure that he now wants to change his goal.
We may find that the patient who wanted to stop entirely now feels he would like to try to control his drinking. He may feel that having lapsed and finding that this did not lead immediately to heavy drinking might imply that he could control his drinking. If so, we need to look again at this patient's drinking history – has he had periods in the past where he stopped drinking, and then re-started drinking quite lightly with it gradually increasing? If so, this may be a repeat of the same situation. With any patient who wants to change his drinking goal to a controlled drinking one, we need to go over all of the things that have been discussed in *Chapter 5* of this manual about how to help someone decide on a controlled drinking goal, and then how to implement it.

Similarly**,** we may find that the patient who is controlling his drinking now wants to build into his Controlled Drinking Plan an occasional time (once a month maybe?) when he can go out with his friends and drink heavily. Part of the plan then would be him monitoring this to see that the 'once a month' did not start to creep up to being 'once every three weeks', and then 'once every two weeks', etc.
But these changes also need to be undertaken with caution – the patient needs to understand that re-deciding on a drinking goal can be a sign of someone starting back down the slippery slope into developing a drinking problem once again.

What if the lapse does turn into a relapse?
Even with all this preparation, many of our patients will allow a lapse to turn into a relapse. If this happens, we need to follow very similar steps to those above – try to get the patient to come to see us, and when he does, get specific details of the relapse from the patient, and then, depending on the details, clarify for each step what the patient could have done differently, and what he will do differently if similar situations arise again.
Again, we will need to re-visit the Change Plan and look at the drinking goal – if the drinking goal was Controlled Drinking, it may be that this relapse means that the patient wants to re-consider that goal and try completely stopping. If the drinking goal was to stop completely, it may be that this relapse means that the patient wants to re-consider this – he may feel that if he had known more about how to control his drinking, the lapse would never have turned into a relapse.

Counsellor:	*So Mr Kamat – it is great that you have been controlling your drinking so well for the past few weeks now. As you know, controlling your drinking is often very difficult for people whose drinking has already got out of control, so I really am impressed with how well you are doing!*
Mr Kamat:	*I'm pleased too! I only came back today to tell you that things are going well, so I don't suppose we'll meet any more.*
Counsellor:	*I'm glad you raised this. As I said when we were working out the agenda, there are some other issues we could discuss that I think will be helpful to you, if that is OK?*
Mr Kamat:	*OK, if you want.*
Counsellor:	*As I say, you have been doing really great! But I know that with all problems like this, things can go wrong. I know you have told me that they will not, but if things do go wrong, it will be really good to have worked out beforehand what to do! Spending a bit of time in this session and then with you thinking about things and practicing them outside of the session will help you to do that.*
Mr Kamat:	*Well – if you put it like that – yes, I can see that sounds sensible.*
Counsellor:	*OK. I'd like to explain what happens when things do go wrong, and then work out with you what we need to do to make sure that if things do go wrong, you are well prepared.*

	You are controlling your drinking at the moment, and that is your goal. If you were to not stick to your Controlled Drinking Plan and were to drink quite a lot more one time when you went out, we'd call this 'a lapse'. It means that there has been one single occasion where you have not kept to your plan. If you then move off of your plan and start going out drinking more often than you have planned, or if each time (or most times) you go out you then drink more than you have planned, then we'd call this 'a relapse' – not just one occasion but a series of occasions where you have not kept to your plan. Am I being clear?
Mr Kamat:	*Yes – if I break my plan once, it is a lapse – if I break my plan lots of times, it is a relapse.*
Counsellor:	*Great. We need to try to make sure that you don't have any lapses at all! But – if you do have a lapse, we need to make sure that you stop there, and do not allow the lapse to turn into a relapse.*<br>*So – how do we make sure that you don't have any lapses? We find that when someone has a lapse, it is triggered by something. An example might be you meeting a friend in Mapusa who you haven't seen for a while and having a chat, and him suddenly saying – 'let's get out of the sun and carry on talking – let's go to the bar over there'. So your harmless chat suddenly puts you into a 'high-risk situation' as you are going to be sitting in a bar, and this is not a day on your plan when you are intending to drink, and also your plan states that you won't drink until it gets dark in the evening.*<br>*Our job then is to work out together as many high-risk situations as we can, and work out for each one what you could do or say to get you out of the high-risk situation safely. Can we do this?*
Mr Kamat:	*OK …. Actually I can think of one – I went with my family at the weekend to visit my wife's brother, and he took me to another room and showed me this lovely bottle of whisky he had just got – a very expensive foreign brand! He suggested that we have a quiet drink while our wives and children were together. I felt really bad, but I managed to say 'no', as it was in the daytime.*
Counsellor:	*That is a great example Mr Kamat – and let's quickly goes through how you managed to say 'no' – what exactly did you say? …..*<br><br>[The counsellor then gets Mr Kamat to generate as many other high-risk situations as he can, with some prompting from the counsellor.]<br><br>*So, let's take each of these, one by one, and I'm going to ask you to work out what you could say or do to get out of that situation. OK? Often, you'll use some of the skills that we talked about last time, about handling your feelings, or about handling the urge to drink, or about saying 'no' to a drink.*<br><br>[The counsellor does this, and gets Mr Kamat (with some help and prompting) to come up with things that he could say or do for each situation.]<br><br>*That has been really great Mr Kamat – well done! Now I want you to go over all of these things at home, after our session, and over the next few days, and practice saying some of the things that we have just been talking about. I really want you to practice saying these things out loud – not just to yourself! You could do this in front of a mirror, or you could do it in front of your wife, or your son – the point is, I want you to get the experience of hearing yourself say these things, again and again, so that if you ever have to say them in a real situation, the words just flow easily. We'll add this task to the Action Plan at the end of this session.*
Mr Kamat:	*This has been really helpful. I can see that if I practice saying these things, or doing these things, then it will be much easier if such a situation comes up.*
Counsellor:	*OK. However – sometimes, even with all of our efforts, we do slip up! I know that you are really committed that this will not happen (and you showed that by turning down*

	your brother-in-law and his lovely whisky!), and you may be one of the people who doesn't slip up. But because lots of people do, I want us to go over now what to do if you do slip up.<br><br>*We have just gone over lots of things that you can say or do to get you out of high-risk situations. I want us to now go over things that you could say to yourself if you have a slip-up, and things that you could do, if you have a slip up? OK? So – if you were to slip up, what could you say to yourself to enable you to stop after an occasion where you're drinking was uncontrolled?*
Mr Kamat:	*I don't know ….. Maybe … 'I mustn't make a small stumble into a catastrophe?'*
Counsellor:	*Yes – that is great! And it is true as well! Can you think of any other things you could say? … And are there things that you could do, as well as say?*<br><br>[The counsellor gets Mr Kamat to generate as many things as he can and then adds in any others].<br><br>*OK – and there is one final thing in this fire drill! If you do have a lapse, I want you to come back to see me, so that we can discuss what happened and I can maybe help you to work out how to avoid it happening again. What we'd do is to go over exactly what happened, and why any of these things that we have talked about today didn't work, and then to work out what would have worked in the situation where you did lapse. I know that you could do all of that on your own, without me, but in my experience, it is better for us to do this together, especially the first time that you have a lapse.*<br><br>*So, I want you to promise me that, if you do have a lapse, you will come back to see me! Is that OK – will you make me that promise?*
Mr Kamat:	*Yes, surely – but I don't have any more appointments with you!*
Counsellor:	*That is OK – if you do have a lapse, just telephone or come in, and re-refer yourself, and I will see you.*<br><br>*One last thing before we move off this topic. Sometimes, patients who lapse do not stop after the lapse and go on to a full relapse. I am pretty sure that this won't happen to you – you are very determined – but you never know – relapses can take people by surprise. So – if you were to have a full relapse – please, please still come back to see me – re-refer yourself – and we will work together again to get you back on course! OK?*

The specific role of the SO in relapse prevention and management
The SO, if s/he is involved, will play a key role in helping

a) The patient to achieve his goals
b) To identify at-risk situations
c) The patient to practice skills to both avoid and manage with these situations

Because lapse and relapse happen outside of the counselling sessions, the SO (who is likely to be in much more contact with the patient than we are) will be able to intervene and assist the patient in our absence to implement the plans that we have all made together.

ENDING PHASE THREE
Sometimes we will get on to the issues of lapse and relapse in session 2 and complete our counselling at that stage. More usually, we will get onto these issues in session 3. Sometimes we will deal with these issues in session 3; with other patients we may need both sessions 3 and 4 to deal with Phase Three properly, in order that our patient can go away and practice some of the ideas and techniques we have suggested. But at some point in sessions 2 or 3 or 4, we will need to end our counselling.

Ending well aims at

1. Summarising and reviewing what has helped this patient (and his SO if present) during counselling
2. Clarifying with the patient (and his SO) how they need to continue to take such actions in the future

Overall, the patient (and his SO) needs to be able to

1. Identify potential triggers that may increase the risk of him lapsing or relapsing into Harmful or Dependent Drinking in the future; and
2. Understand what action he needs to take to ensure that he does not do this. That is, he (and his SO if s/he is engaged with this work) needs to
   - Look at each potential trigger
   - Work out what he could say or do to get himself out of that situation
   - Then practice saying or doing those things (in front of the mirror or with his SO)
   - So that he gets the experience of hearing himself say or do these things
   - So that if ever he has to say or do them in a real situation, they just flow easily
   - Plus, he (and his SO) needs to go over the things that he could say or do if he does actually lapse
   - Plus, if he does have a lapse, he needs to come back to see us, so we can work out how to avoid it happening again

We want to get our patient to commit to doing all of these things

We also need to end Phase Three with a summary of all of our discussions in the respective sessions. Often, it is a good idea to ask the patient and his SO to summarise all of the information and ideas and skills and techniques that we have gone over with us, then adding in any extra ones that they have forgotten. We could say:

'As this is our last session, I would like to know from you what it is that you have learnt from these sessions that we have had together.' [And we will need to clarify any information that is not clear.]

We can draw this last session together by going through the 'end of treatment' form with the patient, in the session. This involves the following steps:

1. <u>Go over the activities</u> that the patient has found useful during counselling and write these down. The list will include things that the patient is doing differently that he needs to maintain or increase.

'Over the time we have been meeting, there are things that you have begun doing that have helped you stop drinking / control your drinking. Can you tell me what these are?'

2. <u>Go over the things</u> that we have identified in counselling that are either 'triggers' for drinking, or are 'at-risk' situations – these are all areas that the patient must practice avoiding or dealing with

'Remember what we discussed about why you were drinking, when we first met? What were the things that were happening in your life and the effects these things had on you?'

3. <u>What can help</u> when difficult situations arise?

For example, breaking down activities or problems into small steps, or seeking the help of an SO in a difficult situation.

'So if you begin to feel the same way (or do the same things), how will you be able to take action to avoid or deal with the situation?'

It is also very useful for us to highlight specific actions that the patient has used to overcome their drinking problem that may not be clear in the patient's memory. For example, we may say:

'You told me that when you felt like drinking, watching a DVD of a favourite film helped to take your mind off that.'

We need to emphasise the patient's role in getting better. The patient may fail to recognise the efforts he has made and attribute his improvement to us. It is therefore important to remind him of this. For example, we may say:

'You have done really well in controlling your drinking. I know you think that you could not have got here without my help, but I want you to realise that every time you have said 'no' to a further drink, it has been you who has done that, not me! Every time you have decided not to go out drinking

as it is not a 'drinking day' it is you who has made that decision. And every time you have not reacted to your father's nagging and unreasonable behaviour by going off drinking, it is you who has done that, not me!'
We need to motivate patients to use the skills across other life situations. A patient seeing us may often be of the opinion that the skills learnt to solve a particular problem may apply to that problem only. In such a case, it can be helpful to encourage the patient to identify and apply the skills across different situations. For example, we may say:

'These skills of solving problems that we have gone through – you know, these don't only work when we try to look at at-risk situations for you going back to drinking. We can use exactly the same system to look at any area of difficulty – for example, you trying to decide whether to stay here in Goa and earn good money or to return back to Assam where your parents want you to get married and settle down near them'
Or
'You can use these skills of working out exactly what you could say and then practicing saying it out loud for other things as well. We did that when we were working out what you could say when you were having a quarrel with your wife – but you could also use the same technique when you think through how to deal in a calmer way with your seniors at work'

Again, we want to get our patient to commit to using these skills and techniques across other areas of his life.
At the end of this summary, we should then ask for his commitment to act on all of these decisions (e.g. the ones about dealing with at-risk situations, and the ones about using all of the skills in other areas of life).
Before we end the session we should share with the patient (and his SO) about how they can contact us or any other service agency for any problem related to their drinking in future. And we need to tell them that we will make contact with them again (by telephone if they have given permission for this) in about two months' time, just for a chat and to see how they are doing. We might even set a date now, for when we will contact them in two months' time.

The specific role of the SO in ending well
The SO, if s/he is involved, has an important role to play in helping summarise the sessions, and clarify the skills that the patient and the SO will use after the counselling has finished.

SUMMARY

- When a patient starts drinking again after a period of planned complete stopping or controlled drinking then it is referred to as a lapse. If the drinking continues and goes back to harmful levels, it is known as a relapse
- It is commonly believed that a relapse occurs suddenly and without any warning signs. Generally, this is not true
- There are both external and internal triggers for both lapse and relapse
- A counsellor can work with the patient to prevent a lapse from happening; and to avoid a lapse, if it does happen, from turning into a relapse
- We need to ensure that our counselling ends well, which we do by getting the patient (with help from ourselves) to review the skills and strategies that the patient has learnt, clarifying any skills or information that are not clear, and reinforcing the patient's motivation to use these skills and strategies across other areas in his life
- Although we are ending our sessions, we also need to let our patient know that he can re-refer himself to us, if needed, either in person or by telephone, requesting a re-referral
- And we need to tell our patient that we will be making a brief contact in about two months' time, just to say 'hello' and to check that everything is OK

Chapter 8

Dealing With Challenges

Learning Objectives

In this chapter, we will learn:

- Identify and tackle challenging situations which arise during counselling

CONTENT

GENERAL COMMENTS

Challenges can arise during any type of treatment, including counselling. Most of the challenges that arise can be tackled if we anticipate them and prepare accordingly.
However, some challenges cannot be anticipated and hence might appear difficult to tackle. When such challenges arise you can discuss them with your supervisor or fellow counsellors so that you can be prepared if a similar challenge arises the next time. The following are some of the more common challenging situations that you may encounter, with tips on how to handle them.

Two specific challenges – dealing with patients having Depression as well as Drinking Problems and dealing with Domestic Violence – are especially important. Dealing with Depression is covered in a short separate chapter, immediately following this one; and Dealing with Domestic Violence is dealt with in the 'Managing Domestic Violence' section within the 'Managing Personal Crises' chapter of the CR manual.

The challenges covered in this chapter are listed below.

CHALLENGES RELATED TO OUR OVERALL CAP COUNSELLING

- Not agreeing/thinking that he has a drinking problem
- Not being interested in counselling
- Wanting medicines
- Inability to attend counselling
- Missing appointments
- Expecting you to give him directive advice
- Doesn't feel comfortable talking about themselves
- Doesn't want to be there and has been coerced by family members or others to attend

CHALLENGES TO DO WITH DRINKING GOALS OR SEVERITY OF PROBLEMS

- Risky Drinking Goal
- Patients with alcohol dependence
- Severe physical health or mental health problems

CHALLENGES RELATED TO PARTICULAR ASPECTS OF A PATIENT'S PRESENTATION

- The drunk patient
- The hostile patient
- The suicidal patient
- The patient who makes advances

OTHER CHALLENGES

- SO and/or other family members interfere negatively during a session
- Not completing homework

CHALLENGES RELATED TO PATIENT'S EXPECTATIONS OF CAP COUNSELLING

**Not agreeing/thinking that he has a drinking problem**
**Challenge**: When we provide personalised feedback, the patient does not think that he has a drinking problem
**Reason**: Some patients do not wish to accept that they may have an alcohol-related problem. This may be because they have never previously thought about their alcohol use in this way.
**What we can do**: We should not enter into an argument with him, nor try to convince him that he really does have an alcohol problem. He may not have. Instead, we need to focus more on working

with him to look at the advantages and disadvantages of drinking, and see if we can help him realise that there may be benefits if he starts to change things. See the section titled ‘What if our patient does not think he has a drinking problem’ in *Chapter 5*, section 5, on helping our patient to decide on his drinking goal.

Not being interested in counselling

Challenge: Patients may say to you that counselling or just talking about the problem is not going to help. Some patients may demand or expect medicines (See also challenge on ‘wanting medicines’).

Reason: Patients coming to the PHC are accustomed to very short consultations with the doctor, very little counselling, and only receiving medicines. Thus, it is not surprising that some patients may express such views when they encounter counselling for the first time.

What we can do: Thank the patient for being for being honest about his feelings. You could say, "*I'm glad you expressed your concerns to me straight away*". Addressing the concern by talking about it openly might give the patient the confidence to give counselling a chance. Things that you could say in such situations include the following:

- *What I am offering is a well-recognised treatment for your health problems, just like a medicine. Also, this is an addition to any medicine you need (which the doctor has prescribed) and will increase the chance of recovery*
- *Whatever you decide is up to you, but it might be helpful for us to talk about why you are concerned about receiving counselling*
- *No one can guarantee that any particular treatment will work but now that you are here you could give this counselling a try and see if it helps you in any way?*

After saying any of these, you should also assure the patient that he has the right to quit counselling at any time, seek help elsewhere or decide to work on the problem on his own.

Wanting medicines

Challenge: The patient and/or his family members attend the counselling session expecting to be prescribed medications.

Reason: Culturally, because of their past experience, patients (and family members) in India expect a health professional (a counsellor like you) to prescribe medicines and they are not familiar with treatments like counselling which rely on talking with the patient.

What we can do: Explain to the patient and family that there is no ‘magic pill’ which will ‘cure’ drinking problems. The treatment for alcohol problems primarily comprises of counselling; medications are only used for people with very severe drinking problems (as discussed in *Appendix 3*). If the patient has alcohol dependence or alcohol withdrawal, we need to simultaneously request the PHC doctor to refer him to a consultant psychiatrist. Explain also that any medications which are needed for other health problems related to the drinking (e.g. reduced appetite) will be given by the PHC doctor.

Inability to attend counselling

Challenge: In this instance the patient tells you that he cannot attend counselling sessions.

Reason: This could include lack of reliable transport to the health centre; lack of money to pay for transport to the health centre; the patient is not free at times when the health centre OPD is working; or the patient is physically unwell to travel to the health centre.

What we can do: We could offer to do the counselling through a home visit or over the phone. (See ‘Conducting home visits’ and ‘Using the telephone in counselling’ sections within the ‘Creating the right conditions for getting started’ chapter of the CR manual)

Missing Appointments

Challenge: The patient does not attend a scheduled appointment with you.

Reason: There could be many reasons for a missed appointment. Some reasons could include the following:

- A practical problem like illness or transport problems
- Patient is not sure whether he needs counselling or not (e.g. *I don't really have that much of a*

problem)<br>• Patient is not sure if he wants to change his drinking behaviour or not<br>• Patient believes that changing his drinking behaviour is impossible and he will not be able to do it<br>• Anger at having to have treatment if he has been forced by family to do so<br>• Patient does not feel the sessions have helped him or has no faith in the counselling<br>• Feeling better/reduction in problem which leads the patient to feel he does not need counselling anymore
**What we can do:** When a patient misses an appointment, respond immediately; if possible, the same day. The longer you take to respond, the less likely it is that the patient will return to counselling. You could either try to reach him over the phone (or you could visit him at home if you have sought permission to do so in advance) (You should look at the appropriate Counselling Relationship manual sections). When you speak to the patient, do the following<br>• Say how disappointed you were not to see him at his appointment<br>• Clarify the reasons for the missed appointment<br>• Express your eagerness to see the patient again<br>• Express your optimism about the patient's capability to change his drinking behaviour<br>• Reschedule the appointment

**Expecting you to give him directive advice**
**Challenge**: Some patients might expect you to tell them what they should do for their alcohol problems rather than working collaboratively with you to generate their own solutions.
**Reason:** Culturally, because of their past experience, patients in India (and around the world) expect a health professional (a counsellor like you) to tell them what to do.
**What we can do**: It is possible to give advice but to do so in such a way that the patient has a say in it. As indicated throughout this manual, always ask for permission before providing advice. After providing advice, always ask if that was helpful and whether the patient has any queries.<br><br>**Counsellor:** *You told me earlier that you tend to drink because your friends force you to. Is there any way in which you could say 'no' to them when they force you to drink?*<br>**Patient:** *I don't know. I was hoping that you would tell me how to do that.*<br>**Counsellor:** *Would it be helpful if I made some suggestions here?*<br>**Patient:** *That would be good*<br>**Counsellor:** *Do you think that it might be helpful if you told your friends that you had decided to cut down on your drinking? If you also told them the tactics you were going to use (such as drinking a non-alcoholic drink every alternative drink, or drinking at half the rate that everyone else was drinking, or limiting yourself to 2 pegs) it may mean that they would be much less likely to try to persuade you to drink more? Do you think that would work?*

**Doesn't feel comfortable talking about himself**
**Challenge**: Some patients might feel very uncomfortable talking about themselves, or about personal issues.
**Reason**: Some patients are more shy and reticent than others; and culturally many people in India are unused to speaking about personal or private issues with strangers.
**What we can do**: We need to use the counselling skills outlined in the Counselling Relationship manual and in this manual to gently draw out our patients and SOs, and to respond helpfully and encouragingly, so that they do start to feel more comfortable talking with us.

**Doesn't want to be there and has been coerced by family members or others to attend**
**Challenge**: If patients have been coerced to attend them often feel resentful and can be uncooperative.
**Reason**: People do not like being coerced into doing most things, and if they are, they are likely to react negatively to it.
**What we can do**: We need to find out (by using our counselling skills) if they do feel coerced, and if

so a) explain that we have not been the ones that have coerced them and b) using our motivational techniques, try to get the patient to reflect on why their family member might have coerced them to come?

CHALLENGES TO DO WITH DRINKING GOALS OR SEVERITY OF PROBLEMS

Risky Drinking Goal

Challenge: Your patient says that he plans to cut down on his drinking rather than quitting altogether. You are concerned that this is not a realistic plan, and that not drinking at all would be a safer goal. [see ‘Drinking Goals’ section in *Chapter 5* of this manual**]**

Reason: There could be many reasons why a patient wants to try to cut down or control his drinking. These will include the fact that he may feel that his problems are not very severe, or that the positives associated with his drinking may outweigh the negatives.

What we can do: Reducing drinking to levels where it does not cause any harm will be a realistic goal for some patients. However there are some patients for whom this might not be a realistic option (See the section in *Chapter 5* on setting Drinking Goals), for example those who have alcohol-related physical or mental health problems. To deal with such situations we need to both be supportive of the client pursuing the goal that he wants, and indicate to him why we are concerned. We certainly must not respond using de-motivating strategies like lecturing, warning, or shaming him (e.g. *If you don’t stop drinking, you will kill yourself*), as this is very likely to make the patient become more resistant to stopping drinking altogether. But neither should we simply agree with him nor accept that must he do whatever he wants to. The patient needs to know that we are concerned about his decision and think that he should reconsider, but that we will support him whatever he does decide to do. We need to communicate effectively our concern, in a compassionate way and respecting the patient’s judgment even though we might not agree with it. This will increase the chances of the client hearing us and responding to our concern.

The following steps give an example of how to do this effectively:

Reflect the patient's goal about which you are concerned

e.g. *You really don’t want to quit drinking completely, but you do see some reasons to cut down*

Ask permission to express your concern

e.g. *I think I understand what you want to do, and why. I wonder if it would be okay for me to tell you a few things that occur to me as I listen to you, which you might want to consider*

State your concern

e.g. *I’m concerned because you told me that your liver is badly damaged and if you continue to drink even at areduced level the damage will get worse*

Ask patient to respond to your concern

e.g. *Do you understand why I’m concerned?*

It is important that you should not<br>• Say nothing<br>• State your concern weakly<br>• Lecture<br>• Argue<br>• Shame<br>It is important that you should<br>• Communicate compassionately<br>• Communicate your concern<br>Respect patient's judgment

**Patients with Alcohol Dependence**
**Challenge**: Patients who are Dependent Drinkers will need to be referred on to see a psychiatrist, as well as seeing us as counsellors.
**Reason**: Patients who screen positive for either Harmful or Dependent Drinking will join PREMIUM, and come to see us. Our counselling works with people who are either Harmful or Dependent drinkers. But if a patient is a Dependent Drinker it is likely that he will need other help, at the same time as our counselling. He may need medication to help with his withdrawal effects (see *Appendix 3*) and he may even need to spend some time in a hospital. The patient will have to be referred to the psychiatrist at the Asilo Hospital, Mapusa, or the IPHB in Bambolim as per patient preference.
**What we can do**: The referral pathway (See *Appendix 2*) in the manual will show us how to refer, when we encounter this situation.

**Severe physical health or mental health problems**
**Challenge**: The patient has screened positive for Harmful or Dependent Drinking, and after assessing him you realise that he also has severe physical health problems or severe mental health problems.
**Reason:** In PREMIUM we are screening solely for alcohol problems and for depression. If someone has such obvious mental health problems that they cannot even get screened, then they will not get through to us seeing them. But people with other serious physical or mental health problems will come to see us, and their problems may only become evident once we start our sessions with them.
**What we can do**: The referral pathway (See *Appendix 2*) in the manual will guide us to the appropriate step to take when we encounter any of these situations. When we refer to the psychiatrist for mental health problems and medical officer for the psychical problems or our supervisor there will be two potential scenarios: a) We will continue to see the patient simultaneously or b) We will stop seeing the patient. Here are examples of what we could say in either of these two scenarios:<br><br>a) For those with severe physical health problems where we will continue to see the patient<br>*'From what you have told me, I understand that your liver has been severely affected and you will have to be admitted to hospital for a few days. However, this will not in any way affect the work we can do together. What I would suggest, if that is alright with you, is that we take a short break while you are in hospital and restart our sessions once you are discharged. In the meantime I will remain in touch with you over the phone. Is that fine by you?'*<br><br>b) For those with severe mental health problems where we will have to stop seeing the patient<br>*'From what we have discussed today, I understand that you have some psychological health problems as well as problems with alcohol. You have also indicated to me that you want to stop/reduce your drinking of alcohol. However, considering the severity of your problems, it would be unsafe for you to do that without the supervision of a doctor. As much as I would like to help you, unfortunately, I am not trained to provide counselling to men like yourself who have such problems. Hence I will not be able to see you for any further sessions. However, I will refer you to my supervisor who is a psychiatrist who will be able to help you.'*

CHALLENGES RELATED TO PARTICULAR ASPECTS OF A PATIENT'S PRESENTATION

**The Drunk patient**
**Challenge**: The patient comes drunk to the counselling session or is drunk during a telephone session/home visit
**Reason**: Many patients find coming for counselling to be an anxiety-raising experience; and many others are highly ambivalent about discussing or changing their drinking. So it is not surprising that many of them find a drink helps them to get the courage or commitment to attend. Furthermore, some patients who are very dependent on alcohol need to have some alcohol in their system to stop them experiencing fits or other serious withdrawal effects. Often patients who had planned to just have a little bit to drink consume more, because they are anxious.
**What we can do**: Just because a patient has consumed alcohol is not a good reason not to continue with the counselling session. The key issue is whether or not the patient is clearly showing obvious signs of intoxication. You need, based on your experience, to make a judgement on whether the patient is too drunk to understand what you are saying or not. If so, the session shouldn't go ahead as it is not likely to be useful. If you have to terminate a face-to-face session (in the PHC or home visit) because the patient is too drunk, try to have a brief discussion aimed at finding a time as soon as possible where you can re-schedule; and if such a discussion is impossible, then give him an alternative appointment on an appointment card so that he does not forget once he is sober. Follow this up with a reminder phone call just in case he has lost the card. Similarly, if you have to terminate a telephone session because a patient is drunk then call again the next day to conduct a session or fix a time for a session. Try to call at a different time of the day i.e. if you had to terminate a session because the patient was drunk in the afternoon, make the next call earlier in the morning.

**The Hostile patient**
**Challenge:** Sometimes we encounter patients with Harmful or Dependent Drinking who are angry and hostile and put us in difficult to handle situations. Handling patients who are hostile (whether in a counselling session at the PHC or on the telephone or during a home visit) always remains a challenging situation.
**Reasons for patient turning hostile:**<br>• Patient under influence of alcohol<br>• Patient having antisocial tendencies<br>• Patient unsatisfied with the quality of the provided services
**What we can do**: The first and most important step in handling the patient who is hostile is to identify the signs and symptoms of hostility in the patient.<br>Below is a list of certain behaviours which indicate hostility:<br>• Blaming<br>• Arguing<br>• Talking over the counsellor<br><br>In addition to these behaviours, there are certain signs related to the body language of the patient which indicate hostility:<br>• Raising voice<br>• Patient staring or glaring at and into the eyes of the counsellor<br>• Tightening of fists<br><br>The most important thing that we should insure in such a scenario is our own safety. Please refer to the Counselling Relationship manual for guidelines for ensuring our own safety. Once we have ensured our own safety then the next important step is to help patient to control his hostile behaviour. The key thing is not to inflame the hostility, or to enter into a confrontation. All the skills mentioned for rolling with sustained talk or rolling with resistance are useful in handling the hostile patient.

The Suicidal patient

Challenge: Realising that our patient may be suicidal.

Reason: In face of difficult life situations patients with harmful drinking may have thoughts of harming themselves.

What we can do: The section on managing suicide risk in the Counselling Relationship manual has details about the skills to handle such a scenario.

Important things to be remembered in handling such a situation include:

- Conducting a detailed suicide risk assessment
- Assessment of risk and protective factors
- Plan appropriate action which includes timely referral to the psychiatrist

Key skills to be used when working with a patient where there is a risk of suicide:

- Establishing a trusting relationship with the patient
- Encouraging the patient to talk about his problems
- Restoring hope
- Focusing on protective factors
- Activate family/social supports
- Removal of all means of committing suicide
- Ensuring that the patient has immediate 24-hour access to suitable care
- Making a contract with the patient where he promises not to harm himself
- Always conduct an early follow-up assessment

The patient who makes advances

Challenge: Sometimes we encounter patients with Harmful or Dependent Drinking who make unwarranted advances towards us in the counselling session.

Reasons for the patient making advances: these may include the patient

- being under the influence of alcohol
- having antisocial tendencies
- misunderstanding the nature of the counselling relationship – the issues raised in the Counselling Relationship manual about the differences between being 'friendly' and being 'a friend'

What we can do: If a patient starts making advances, either verbally or physically, we need to stop him and deal with this immediately.

We could say:
'I am sorry Mr D'Silva, but what you are saying / doing is actually inappropriate. This is a counselling session, and I am a professional counsellor. I am not offering to be your girlfriend.'

Usually this will be sufficient and the patient will apologise, but if he continues, we must first threaten to stop the session ['*Let me be very clear, Mr D'Silva – if you do not stop saying things like this, we will have to stop this session*'] and if that does not work, we must stop the session, and discuss with our supervisor what to do next – offer another session (and if so, under what circumstances and with what safeguards) or not.

Other challenges

SO and/or other family members interfere negatively during a session

Challenge: Sometimes the SO accompanying the patient may be a hindrance to counselling. They could do this by

- Not allowing the patient to speak
- Constantly interrupting you and/or the patient
- Expressing a lack of confidence in the patient's ability to change
- Making negative comments about the patient

**Reasons**<br>• The person accompanying the patient is not an SO but a friend, family member or neighbour who is not necessarily invested in the patient changing his drinking<br>• They are so annoyed and disappointed with the patient that they want their feelings known<br>• Because of past experience, the SO genuinely believes that the patient cannot change
**What we can do:**<br>• We can say to the SO/accompanying person that you would like to listen to the patient first and then you will give him/her an opportunity to add his/her bit. You could say to the accompanying person *'What you have to say is important and we would like to hear about it. However, first I would like to listen to Mr Kamat's (patient) side of the story. Once he has finished, I will give you a chance to add to it'*<br>• If the SO/accompanying person continues to be difficult despite you telling him/her that you will give him/her an opportunity to speak after the patient has finished, we can ask him/her to wait outside while you conduct the session. He/she can then be invited in towards the end of the session to make their contribution. You could say to him/her *'What you have to say is very important and we would like to hear it. However, right now, I would like to listen to Mr Kamat's (patient) perspective. If you don't mind, you could wait outside and I will fetch you after some time'*<br>• Reflect the situation back to the SO/accompanying person e.g. *'This is becoming very difficult. I want to be able to listen what you have got to say. But I also have to listen to what Mr Kamat has to say. But your interrupting is not allowing me to do either of those. It would be helpful if you would not interrupt for some time while I hear what Mr Kamat has to say and then I will come back to you'*<br>• It might be difficult to do this when you are doing home visits and sometimes you might have to discontinue the session and reschedule the session to have it in the PHC, with only the patient present. However, this should always be your last resort.
**Homework**
**Challenge:** Patient does not complete the homework you had agreed on
**Reasons**<br>• Patient is illiterate and the homework which was set included reading/writing<br>• Patient does not understand the rationale for doing the homework
**What we can do:**<br>• The solution to this challenge will depend on the reason(s) why the patient did not complete the homework<br>• We will have to use the 'Problem Solving' skills outlined in this manual to find solutions to this challenge.<br>• One solution for the patient not completing the drink diary because he is illiterate is to complete it together with the patient during the session. Although this is not perfect, as we will have to rely on the patient's memory, it is better than not having any drink diary at all<br>• One important thing to remember is that the patient is more likely to complete the homework if he understands the logic behind it. Hence, it is very important that you should clearly explain to him why we are doing the homework and how it will help

Chapter 9

Dealing With Patients With Depression As Well As A Drinking Problem

Learning Objectives

In this chapter, we will learn to:

- Manage patients who have Depression as well as a drinking problem (Harmful or Dependent Drinking)

CONTENT

During the course of your work you will come across men who will screen positive for both Harmful or Dependent Drinking and Depressive Disorder.

In such patients,

- The two disorders could be unrelated to each other
- Or there could be a relationship:
  a) The patient might drink because of his depression (to reduce or forget the symptoms of depression); or
  b) The depression might be a result of his heavy drinking (and we know that heavy drinking does cause depression in some people).

However, most of the times it is difficult to pinpoint the exact relationship (or its absence) between the two.

HOW DO WE KNOW IF SOMEONE HAS BOTH HARMFUL DRINKING AND DEPRESSIVE DISORDER?

You will know this based on the results of the screening. Any person who has an AUDIT score of 12 and above as well as a PHQ-9 score of 15 and above is a patient who has co-existing HD and DD.

IN PATIENTS WITH BOTH THESE DISORDERS, WHICH ONE SHOULD WE TREAT FIRST?

The evidence suggests that if we treat the alcohol problem first, it is more likely to lead to a successful outcome for both disorders. Hence for everyone who screens positive for both disorders, we will treat the alcohol problems first, using the CAP approach. If we are successful and the patient reduces/stops drinking we might find that the symptoms of depression reduce automatically.

CAN WE TREAT BOTH DISORDERS AT THE SAME TIME USING CAP AND HAP?

Although you have been trained in counselling for both depression and harmful/dependent drinking, using both of them together will be complex for you and confusing for the patient. Hence it is important that we avoid using both these counselling methods at the same time for the same patient.

HOW WILL WE KNOW IF THE PATIENT IS STILL DEPRESSED AFTER COMPLETING CAP?

We must rescreen the patient with the PHQ-9 at the end of CAP, to see if they are still depressed.

WHAT DO WE DO IF THE PATIENT IS STILL DEPRESSED AFTER COMPLETING CAP?

However, if the symptoms of depression do not resolve spontaneously even after the patient stops/reduces drinking then we will refer the patient to a psychiatrist.

WHAT DO WE DO FOR PATIENTS WHO ARE SO SEVERELY DEPRESSED THAT THEY ARE NOT ABLE TO ENGAGE IN CAP TREATMENT?

A small proportion of patients with both Harmful Drinking / Alcohol Dependence and Depression will not be able to engage in CAP treatment because of their depressive symptoms. If we think that one of our patients fits this category, we must refer such patients to our supervisor so that they can decide what to do - such patients might require more specialised treatment.

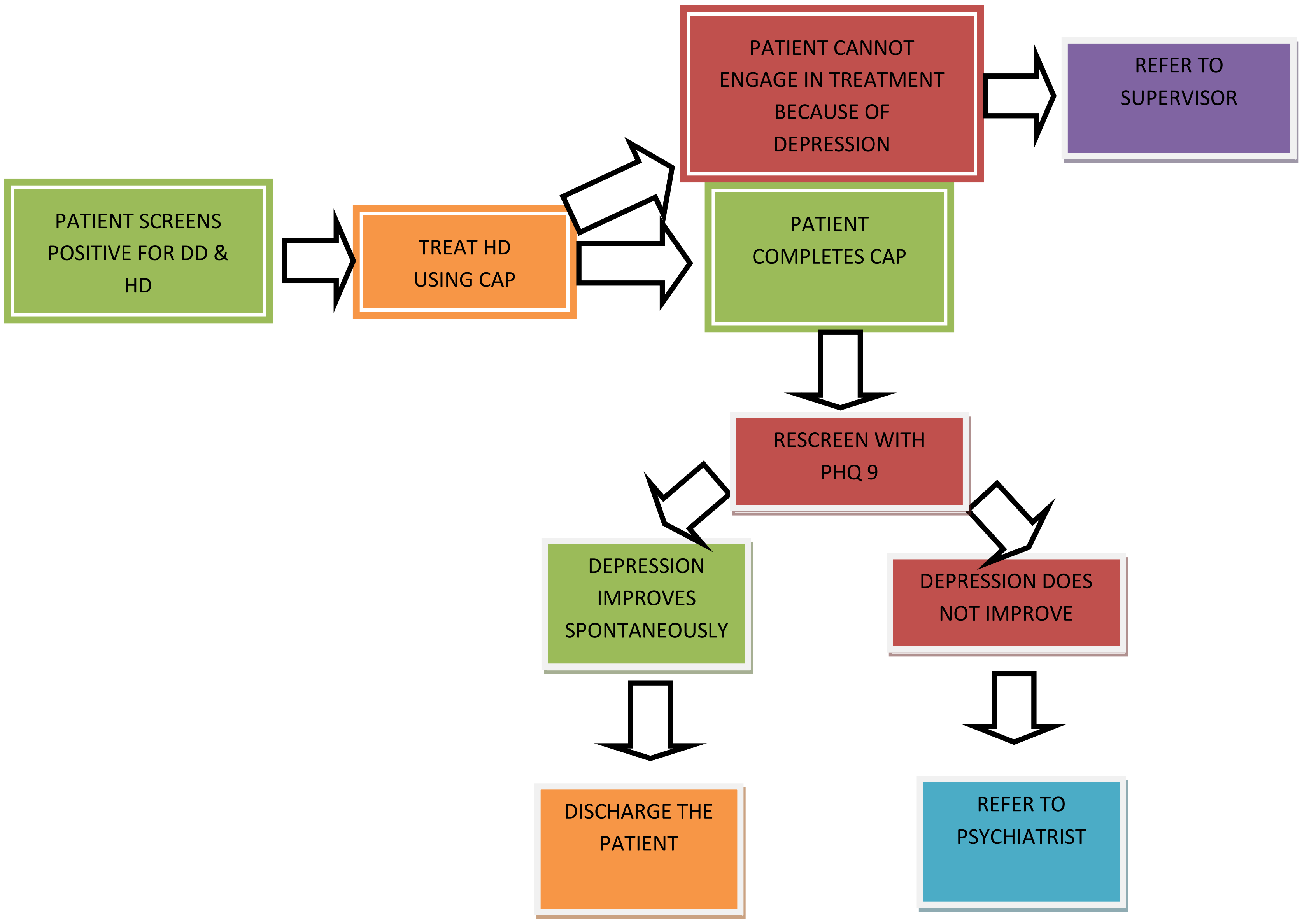
PATIENT SCREENS POSITIVE FOR DD & HD
TREAT HD USING CAP
PATIENT CANNOT ENGAGE IN TREATMENT BECAUSE OF DEPRESSION
REFER TO SUPERVISOR
PATIENT COMPLETES CAP
RESCREEN WITH PHQ 9
DEPRESSION IMPROVES SPONTANEOUSLY
DEPRESSION DOES NOT IMPROVE
DISCHARGE THE PATIENT
REFER TO PSYCHIATRIST

Chapter 10

Dealing With Patients With Tobacco Problem As Well As Drinking Problems

Learning Objectives

In this chapter, we will learn to:

- Manage patients who have tobacco as well as drinking problems

CONTENT

Tobacco use is a common problem that we encounter in patients with drinking problems. Tobacco is used in various forms including chewing, smoking, applying tobacco to teeth and gums, etc.

Tobacco used in any form is highly addictive because it contains nicotine, which itself is highly addictive. Most often, what starts as occasional use becomes a habit. Most people use tobacco because they think that it helps them to relax, improve their alertness or overcome boredom. However, use of tobacco in any forms is hazardous to health. Health consequences of tobacco use include a high risk of early death due to heart disease, breathing difficulties and many types of cancers especially of the mouth and lungs.

HOW WILL WE KNOW ABOUT THE USE OF TOBACCO BY A PATIENT?

As a routine procedure we ask all of our patients about the use of tobacco in the last part of our assessment (described in *Chapter 5*).

Once we finish our assessment of tobacco use, we explain to our patient that this intervention is primarily focused on drinking problems, but that if he wants to work on his tobacco problem then we can help him to try to stop or control his use once we finish the alcohol intervention.

It is also possible (though rarely) that our patient or their SO (if present) may disclose the use of tobacco as their primary concern and want to work on the problem of tobacco before alcohol.

It is important to make the patient aware in a collaborative manner that the focus of this intervention is drinking problems and though the use of tobacco is also important, we can handle that once we finish the alcohol intervention. We could say the following to the patient.

Mr Pednekar	*My smoking is bothering me more than my drinking these days. Can we deal with the smoking instead of drinking?*
Counsellor	*Mr Pednekar I can very well understand your concerns about smoking and I also want to work with you on your smoking, but the primary reason I am working here is to help patients who have drinking problems. If you want to work on your smoking first then I can refer you to a consultant psychiatrist and once you finish that then you can come to me for your drinking problem or we can start with the drinking first and then I can help you to work on your smoking. You can choose any of the options I just described*

HOW DO WE HELP A PATIENT WITH A TOBACCO PROBLEM?

Similar to patients with drinking problems, patients with tobacco use can also be divided into three groups based on their motivation to quit tobacco. We need to match our intervention to each patient's level of motivation as described in the table below:

Table 4: Matching intervention to level of motivation

**Level of motivation**	**Focus of counselling process**
The patient has never thought about changing his tobacco use	We should focus more on helping the patient to understand the advantages and disadvantages of tobacco use, and see if we can help him realise that he might want to start to change things
The patient has started thinking about making a positive change to his tobacco use, but at the same time has a strong feeling for continuing it	We should focus more on helping the patient to resolve the confusion and to make up his mind to change in a positive direction
The patient has already decided to either control or completely stop his tobacco use	We should focus more on helping the patient to decide on the quit date and implement the change plan

For all three groups of patients the intervention should start with the assessment of the tobacco problem.
The important things we need to know in the assessment of tobacco use are

- The form in which tobacco is consumed (smoking/ chewing / applying to gums)
- How long the person has been using tobacco
- The reasons for tobacco consumption
- Their past efforts in quitting tobacco use or reducing their tobacco use
- Their reasons for restarting the tobacco use, if they have reduced or stopped in the past

All the principles learnt during the assessment of drinking problems should also be applied during the assessment of tobacco use.

For the first two groups of patients where the patient is either not too motivated to stop tobacco use or has strong and ambivalent feelings at the same time about both stopping and continuing to use, we can work with the patient using the skills we have learnt in *Chapter 3* under the heading of 'building and developing motivation for change'.
It is also important to provide personalised feedback about the use of tobacco to all patients, regardless of their level of motivation to change. This is similar to the process of personalised feedback we provide for drinking problems. This feedback can include the summary of the information we have gathered in the assessment and also can provide information about the possible effects of the tobacco on the body.
If the patient is willing to work with us on stopping tobacco use after this then we can continue the intervention with the help of the skills mentioned below. If the patient wants some time to take the decision about tobacco then we can end the treatment here and ask him to contact us if they require any help in future.

For the third group of the patients who have decided to stop smoking, we can work through the following steps:

- <u>Congratulate the patient on the decision to quit</u>

This is really important! For many, giving up tobacco is like giving up a part of their lives, so it's important that they are genuinely appreciated for their courage in taking such an important decision – for their own good, and for the good of those they love.

- <u>Confirm the reason for the decision to stop tobacco use</u>

Below is the list of some reasons which patients generally mention as a reason to stop their use of tobacco. It is important for us to know the personal reason for stopping tobacco use as this reason generally works as an important motivating factor for the patient:

- Tobacco kills
- Cigarettes and chewing tobacco are full of toxic additives
- Using tobacco causes diseases like cancer, heart attacks and breathing problems.
- Using tobacco reduces fitness and appearance, and can make you depressed.
- Using tobacco affects fertility, babies and children.
- Using tobacco is expensive

- <u>Assist the patient in setting a Quit Date</u>

Don't let the person postpone. Ask for a decision**:** "I will stop it on ___day". Ask the person to mark this date on a calendar. Some patients might choose a date which holds some special significance, such as a religious occasion, a family member's birthday, etc. However, remember the patient can just pick a random date. What is more important is that he picks a date soon. Tell the person: '*Picking a date too far in the future gives you time to change your mind.*' So try to insist that the person choose.

- <u>Suggest that SOs are involved in the decision</u>

It may be helpful to involve SOs in the decision to quit. For example, a man can discuss this decision with his wife, and they can collectively take a decision that he will stop on a certain day, and that she will provide specific support and help for him over the next 7-14 days, to help him deal with withdrawal e.g. a father can tell his daughter that he will be quitting on her birthday and for a couple

of weeks after he quits if he feels like smoking he will tell her about it so that she can distract him from this craving.

- Encourage the smoker to ask for and accept offers of external help!

Encourage the person to accept the need for external help (family/friends/colleagues). Think: '*Who can help me in quitting tobacco?*'

- Help the patient choose a method for quitting

There is no one right way to quit. Some tobacco users prefer to quit by stopping it completely and all at once. They use tobacco until their "Quit Day" and then stop totally. Others may cut down on tobacco for a week or two before their "Quit Day". Ask the patient what he would prefer.

Advice for the patient who wants to taper off slowly

a) Progressive reduction: Every day cut down on one or more cigarettes/ bidis /packet than the previous day e.g. if you are currently smoking 20 per day, cut down to 15 in the first week, then 10 in the second week, etc. When you come down to the fifth day, take some time before making it 0 per day.
b) Postpone use: Try putting off the first use of the day by one or two hours. Start, for example, with no tobacco use until 9am (assuming you used to smoke your first cigarette in the day around 7am). The next day, make 11am the earliest you light up a cigarette. Go as long as you can without giving in to a craving (desire to smoke). Start by trying for at least 10 minutes, then longer and longer as you near your Quit Day.
c) Pick your three worst triggers (e.g. after waking up, after coffee, after food) and stop smoking or chewing at those times. This will be hard at first, but practise will make it easier.
d) Don't stock! Buy only the number of cigarettes/sachets of gutkha that you plan to smoke/chew each day.
e) On your Quit Date**,** stop tobacco altogether and begin your life as a non-tobacco user.

Advice for the person choosing Option B: Stopping all at once

a) Set your "Quit Date": Before the "Quit Date", make the following changes:
b) Clean up the place! Get rid of reminders of smoking/chewing (ash trays, empty packets, etc.).
c) Change your routine. For example, don't smoke/chew during your regular tobacco using times (e.g. the after-lunch cigarette) or at regular places (bathrooms, workplace, with friends, etc.).
d) "Quit Day": Whatever the method chosen, on the "Quit Day" the patient will need active support and be adequately prepared to deal with urges and withdrawals. The patient also needs to know that health professionals are available should he need any assistance. Here are some guidelines you can use to intervene with your patient on his "Quit Day": Advise the patient as follows:
e) The day before "Quit Day":
    - Keep substitutes handy to put in your mouth, like cloves, sweets, anything you would prefer.
    - Plan a new routine to do first thing in the morning, such as going to a place of worship, gym, yoga class. Let this be something different from your usual routine, so that it keeps your mind away from your regular tobacco use.
    - Ask a family member to be with you. Remind family, friends and co-workers that this is your "Quit Day" and ask them to help and support you.
    - Call and fix an appointment with you (their counsellor) if required
f) On your Quit Day, begin your life as a tobacco non-user.

Below is a list of suggestions to make your new tobacco-free life easier:

- Follow through on the routine you planned the day before.
- Keep busy and find new things to do, spending as much time as you can, in non-smoking places and among non-smoking people.
- Avoid situations where the urge to use tobacco is strong, such as passing by a tobacco shop or interacting with friends who smoke.
- Avoid alcohol. It increases the urge to smoke/chew.

- Drink plenty of cold water, fresh juices, lassi, etc. as substitutes.
- Don't skip food. Eat small amounts at multiple times.
- Learn to relax: Try slow deep breathing if you get tensed. Come and see me! If the urge is very strong and you are afraid you may smoke/chew, come to see me before you do so. Let me support you on this most important day in your life.
- Role of medications: There are certain medications which can help a patient to handle the urge to use tobacco. If your patient wants to try medicines as a part of his efforts to stop tobacco use then you can refer him to the psychiatrist.

Examples of medications which are effective for tobacco use is bupropion, nicotine gum, etc.

Preventing and dealing with relapse

Similar to drinking problems, people have a tendency to restart tobacco use, after they have stopped using it. Before we end a session on stopping or reducing tobacco, our patient should be made aware about the possibility of relapse, and we should work with patient to try to prevent or manage this.

'While some people are able to stop in their first attempt, most of those who stop will need to make several attempts before they are finally successful. In other words, even with the best intentions, most people go back to using tobacco due to reasons like craving or boredom. If this happens to you, do not feel guilty and disheartened as this will increase the chances of you continuing to use tobacco. Instead, accept that this is most often the case, refocus on the reasons on why you want to stop and go through the steps again using your experience usefully.'

All the skills mentioned in section seven on preventing and dealing with relapse can also be useful when we work with a patient about his relapse into tobacco use.

APPENDIX 1

Glossary of difficult words

**Difficult words**	**Meaning/alternate words**
Action Plan	The Action Plan is the set of actions that the patient and the counsellor have agreed will be done in between one session and another. The Action Plan relates to the overall plan and to the Target Behaviour(s) discussed in the session, but it is an account of some more detailed steps that the patient will be taking in between one session and another
Affirming	Using praise
Ambivalence / Ambivalent feeling about change	By ambivalence we mean 'having strong feelings in opposing directions'. For example, a patient with harmful drinking may realise that his drinking has started creating problems in his life and he should change; and at the same time he may also have a strong desire to continue drinking. Helping a patient to resolve such opposing feelings is a very important step in developing his 'motivation *to change*'.
Change Plan	The change plan is the overall plan, outlining<br>• What the changes are that the patient wants to achieve in counselling (e.g. to completely stop drinking)<br>• What his main reasons are for wanting to change<br>• What steps he wants to take to achieve this change<br>• Who can help him achieve this, and how<br>• How he will know if the plan is working<br>• What could interfere with the plan<br>• How these things could be dealt with
Change Talk	A patient's statements which show that they are *in favour of change* are termed '*change talk*'. It helps patients to change their behaviour if they hear themselves stating that they want to do this. If we can encourage the patient to make 'change statements' or to generate 'change talk', it is more likely that they will go on to change.
Coping skills	Coping Skills are skills that our patients use to deal with (or cope with) challenging situations. These situations could include having an urge to drink, or feeling some negative emotion, or being pressurised to drink alcohol when it is not wanted.
Depression	Persistent sadness and loss of interest with reduced sleep/appetite, guilt feelings, feelings of hopelessness, etc.
Drinking Diary	A single sheet of paper in the form of a diary, on which a patient monitors his drinking over a specific period (usually a week, sometimes longer), in a systematic fashion, collecting information about precisely how much is consumed, when, where, and with whom.
Hallucinations	Hearing voices or seeing visions even when one is alone.
Homework	Part of the Action Plan, Homework Tasks are specific tasks that we agree with the patient (and his SO) that he will undertake in between sessions, and tell us about during his next session, such as 'fill out the drinking diary', or 'go to the college and get information about the course that we have discussed', etc.
Lapse	A lapse is when a patient starts drinking again after a period of planned complete stopping, or when a patient who has been in control of his drinking starts drinking again in his previously uncontrolled way.
Motivation to change	The strength of an individual's motivation for and movement towards a specific goal (in this case, changing their drinking behaviour).
Personalised feedback	We let a patient know how and why his drinking may be harmful, and how it may be related to any problems or issues that he may have told us about, during the

	assessment.
Relapse	When a patient has a lapse, if he stops drinking, or drinking uncontrollably, quite speedily, then the lapse remains as a lapse. But if he continues drinking in this way, then his lapse turns into a relapse. Relapse is not just an event of drinking alcohol again but is a process which starts with thoughts about drinking alcohol, goes on to actual drinking, and then continues so that the patient is drinking at increasing quantity and frequency.
Resistance	Resistance is a specific type of 'sustain talk' which is generated through the counsellor's behaviour. If the counsellor tries to push the patient in a direction that he does not want to go, or pushes bossily, then the patient will resist.
Sustain Talk	A patient's statements which argue against any change in drinking behaviour are called 'sustain talk'. The more that patients hear themselves saying that they don't want to change, the less likely it is that they will change.
Target Behaviour	The Target Behaviour is the thing (or set of things) that we are working on during a counselling session. As in the examples in the text, "Typically, these include targets related to drinking (e.g. not drinking at all over the past week, or drinking on only some days and to a pre-agreed level) and those related to other things (e.g. not seeing friends who put pressure on the patient to drink; or dealing with conflict at work or at home in a different way, which does not involve drinking)"
Withdrawal Symptoms	These occur in the most severe form of drinking, called Dependent Drinking. People's bodies have become used to receiving alcohol regularly, and if they do not get it, their body reacts giving very unpleasant (and in extreme cases dangerous) effects, including sweating, nausea, and in serious cases uncontrollable shaking, vomiting, or fits.

APPENDIX 2

Referral pathways

**When to refer**	**Where to refer**
If the patient has a medical problem like heartburn or pain in the abdomen	Medical officer
• If the patient has signs suggestive of withdrawal symptoms like tremors, restlessness, rapid heartbeats, insomnia, etc.<br>• If the patient has alcohol dependence	Request medical officer to refer to Psychiatrist
If the patient has both HD/alcohol dependence and depression but is not able to engage with CAP because of depressive symptoms.	Psychiatrist
If the patient is suicidal (See appropriate section in Counselling Relationship manual)	
If the patient or his family members insist on medical treatment for his drinking	
If the patient has additional severe psychiatric problem like psychosis. Such patients might display unusual speech or behaviour e.g. report hearing things even when they are alone, believe that other people are doing black magic on them, etc.	Discuss with supervisor about referring to Psychiatrist

APPENDIX 3

The role of medication: Medicines for treatment of drinking problems

LEARNING OBJECTIVES

- Which medicines are used in treatment of alcohol-use disorders?
- What is the role of medicines in the treatment of patients with HD?

CONTENT

The use of medicines in the treatment of alcohol-use disorders is primarily limited to dependent drinkers. Although you will not prescribe these medicines themselves, it is important for you to have basic information about the medicines used in the treatment of dependent drinking as you will see some patients with dependent drinking.

There are mainly three different types of medicines used in the treatment of dependent drinking

1) Medication for treatment of withdrawal symptoms:
   Withdrawal is a state developed after sudden reduction/discontinuation of drinking by a person having dependent drinking. Withdrawal symptoms include restlessness, tremors (hand shaking), rapidly beating heart, sweating, sleep disturbances and fits (if there is severe dependency).
   Benzodiazepines like lorazepam (Ativan), diazepam (Valium) and Chlordiazepoxide (Librium) are used for a short duration (one to two weeks) to help patients overcome these symptoms.
2) Medication for treatment of craving for alcohol:
   There are some medicines which have been found to be effective to a certain extent in reducing the intense desire (craving) for drinking alcohol in some patients. These medicines are prescribed by a doctor, usually a psychiatrist. Acamprosate, Topiramate, Naltrexone and Baclofen are some important medicines from this group.
3) Medications used as aversion (i.e. making the person dislike alcohol) treatment:
   This group includes a medicine called Disulfiram or Antabuse. These medicines have a unique mode of action. They have no effect if no alcohol is consumed; however, if a patient consumes alcohol when he is on this medication then he can have unpleasant symptoms ranging from nausea and vomiting to seizure or raised blood pressure. Knowing that he will experience these very unpleasant effects is the reason why a patient avoids consuming alcohol when he is on this medicine. Disulfiram is to be used strictly under specialist observation and with utmost precautions.

APPENDIX 4

AUDIT

1	How often do you have a drink containing alcohol?<br>*Tu kitlem pavtti soro pieta?*	(0) Never (*Kednach na*)☐<br>(1) Monthly or less *(Mhoinean ek davo tem poros unnem*) ☐<br>(2) Two to four times in a month(*Mhoinean dhon te char pavtti*) ☐<br>(3) Two to three times a week (*Sumonan dhon te teen pavtti*) ☐<br>*(4)* Four or more times a week (*Sumonan char pavtti va te poros chodd)*☐
2	How many drinks do you have on a typical day when are you are drinking? (Note: 1 drink=10g).<br>*Je disam tu soro pieta, te disa tu kitlo pieta/ kitlem drinks pieta?*	____________________________________<br>(0) 1 or 2 drinks (*Ek vo dhon drinks*) ☐<br>(1) 3 or 4 drinks *(Teen vo char drinks*) ☐<br>(2) 5 or 6 drink (*Paanch vo so drinks*) ☐<br>(3) 7 or 8 drinks (*Sath vo aat drinks*) ☐<br>(4) 10 drinks or more (*Dhavo chodd drinks*) ☐
3	How often do you have 6 or more drinks on one occasion?<br>Note: 6 or more drinks are equal to 60gm or more. Hence the equivalent of 3 or more regular bottles of beer, 2 or more bottles of strong beer, 750ml bottle of wine, 1 quarter of IMFL or cajufeni, 1 pint of coconut feni or urrack, 6 bottles of premixed drink (breezer, Smirnoff Ice etc.), or any combination of these beverages that would add up to the listed amount of each beverage alone in one day. E.g. 1 strong (650ml) bottle of beer and 1 peg of IMFL taken together<br>*Ekda pieupaklaglear tu sov vo chodd drinks kitle pavtti pieta?*<br>*Sov vo chodd drinks mhunje 60gm vo chodd. Mhunje teen sadharan vo chodd beerecho battleo, dhon vo chodd kadak/ Strong beer, 750ml winechi batli, 1quarter IMFL chi vo kajufenichi, 1 pint madachi vo urrackacho, sov battleo poilinch misturkelale drinks (breezer, Smirnoff Ice, aadhi) vo anik kosloi soro aslolo misturzoxem promanem voir sanglelea soreachea promanem asa ,je ekach disak ghetla (audharan. ekkadak (650ml) beerchi batli ani IMFLcho ek peg ekaborobar)*	(0) Never (*Kednach na)* ☐<br>(1) Less than monthly (*Mhoinean ekdamhajea-poros unnem*) ☐<br>(2) Monthly (*Mhoinean ekdam*) ☐<br>*(3)* Weekly (*Sumonan ekdam*) ☐<br>(4) Daily or almost daily (*Sodanch vo lagilagi sodanch*) ☐
4	How often during the last year have you found that you were not able to stop drinking once you started?<br>*Gelea vorsa kitle pavtti ekda soro pieunk*	(0) Never (*Kednach na)* ☐<br>(1) Less than monthly (*Mhoinean ekdamhajea-poros unnem*) ☐<br>(2) Monthly (*Mhoinean ekdam*) ☐

	laglear tujean pieup tharaupak zaunk na?	(3) Weekly (*Sumonan ekdam*) ☐<br>(4) Daily or almost daily (*Sodanch vo lagilagi sodanch*) ☐
5	How often during the last year have you failed to do what was normally expected of you because of drinking?<br>*Gelea vorsa bhitor kitle pavtti soreache sonvoienk lagon tujea kodden apekshittikama / tuvem korpachitim kama tujean korpak zali na?*	*(0)* Never (*Kednach na)* ☐<br>(1) Less than monthly (*Mhoinean ekdamhajea-poros unnem*) ☐<br>(2) Monthly (*Mhoinean ekdam*) ☐<br>(3) Weekly (*Sumonan ekdam*) ☐<br>(4) Daily or almost daily (*Sodanch vo lagilagi sodanch*) ☐
6	How often during the last year have you needed a first drink in the morning to get yourself going after a heavy drinking session?<br>*Gelea vorsa bhitor kitle pavtti poile rati chodd soro piella karannan, dusre disa sokalli fuddem nettieupakhatir soro pieucho poddla*?	(0) Never (*Kednach na*) ☐<br>(1) Less than monthly (*Mhoinean ekdamhajea-poros unnem)* ☐<br>(2) Monthly (*Mhoinean ekdam*) ☐<br>*(3)* Weekly (*Sumonan ekdam)* ☐<br>(4) Daily or almost daily (*Sodanch vo lagilagi sodanch*) ☐
7	How often during the last year have you had a feeling of guilt or remorse after drinking?<br>*Gelea vorsa bhitor kitle pavtti piella uprant tu kadukh vo paschattap zala vo vaitt disla?*	(0) Never (*Kednach na)* ☐<br>(1) Less than monthly (*Mhoinean ekdamhajea-porosunnem*) ☐<br>*(2)* Monthly (*Mhoinean ekdam)* ☐<br>(3) Weekly (*Sumonan ekdam*) ☐<br>(4) Daily or almost daily (*Sodanch vo lagilagi sodanch*) ☐
8	How often during the last year have you been unable to remember what happened the night before because you had been drinking? *Gelea vorsa bhitor kitle pavtti soro pieta astana ratcheo gozalli dusrea disa soreacho nett denvtokuch tu kay aadurunk na?*	(0) Never (*Kednach na*) ☐<br>(1) Less than monthly (*Mhoinean ekdamhajea-poros unnem*) ☐<br>(2) Monthly (*Mhoinean ekdam*) ☐<br>(3) Weekly (*Sumonan ekdam*) ☐<br>(4) Daily or almost daily (*Sodanch vo lagilagi sodanch*) ☐
9	Have you or someone else been injured because of your drinking?<br>*Tuvem soro piella karannan tuka vo dusrea konnank maar bosla vo dukhapott zalea?*	(0) Never (*Kednach na*) ☐<br>(2) Yes, but not in the last year (*Hoi, pun gelea vorsa nhoi*) ☐<br>(4) Yes, during the last year *(Hoi, gelea vorsa*) ☐
10	Has a relative, friend, doctor or other health worker been concerned about your drinking or suggested that you should cut down?<br>*Tuzo soyro, ixtt, dotor vo anikonnui arogyasevok/ sevika tujea soro pieupa-vishim chinta porgotlam?*	(0) Never (*Kednach na*) ☐<br>(2) Yes, but not in the last year (*Hoi, pun gelea vorsa nhoi*) ☐<br>(4) Yes, during the last year *(Hoi, gelea vorsa*) ☐
	AUDIT score	____________________

INTERPRETATION OF AUDIT SCORES

For men who attend primary health care services in Goa, 6% score 12-19 on the AUDIT (Harmful Drinking) and 5% score 20 and above on the AUDIT (Dependent Drinking).

This information needs to be provided to the patients when you are summarising the AUDIT score for them and when you provide personalised feedback.

This is what you could say to them:
For Harmful Drinkers:

'*Your AUDIT score of x indicates that you are likely to be a harmful drinker. This means that your drinking is likely to have a negative impact on your health and social functioning. You seem to be drinking more heavily than most men who go to a doctor in Goa*.'

For Dependent Drinkers:

'*Your AUDIT score of x indicates that you are likely to be a dependant drinker. This means that your body has become used to alcohol and you are likely to develop symptoms like vomiting, shaking of the hands, headache etc. if you don't have a drink. You seem to be drinking much more heavily than most men who go to a doctor in Goa.'*.
