# Supplementary material for: Sustained effectiveness and cost-effectiveness of Counselling for Alcohol Problems, a brief psychological treatment for harmful drinking in men, delivered by lay counsellors in primary care: 12-month follow-up of a randomised controlled trial
Source: PLoS Med. 2017 Sep 12;14(9):e1002386. doi: 10.1371/journal.pmed.1002386 (PMC5595289; doi:10.1371/journal.pmed.1002386)
Supplement: S3 Text — (DOC) [file pmed.1002386.s014.doc]

**The PREMIUM randomised controlled trials of the effectiveness and cost-effectiveness of lay counsellor-delivered psychological treatments for harmful and dependent drinking and moderate to severe depression in primary care in India.**

Statistical Analysis Plan Final - 12 months outcomes

Version 2.0 FINAL

12/03/2017

(Registration numbers ISRCTN95149997; ISRCTN76465238)

**Investigators**

Vikram Patel*, Benedict Weobong, Abhijit Nadkarni, Helen A Weiss, Arpita Anand, Bhargav Bhat, Basavraj Katti, Daisy Singla, Ricardo Araya, Sona Dimidjian, Steve Hollon, Michael King, Jim McCambridge, Lakshmi Vijayakumar, David McDaid, A-la Park, Terrence Wilson, Pratima Murthy, Richard Velleman, Chris Fairburn, Betty Kirkwood

**Trials manager**

Benedict Weobong

**Data manager**

Bhargav Bhat

**Trials statistician**

Helen Weiss

**CONTENTS**

[1. Description of the trial 3](#__RefHeading___Toc454978628)

[1.1 Principal research objectives to be addressed 3](#__RefHeading___Toc454978629)

[1.2 Trial design 3](#__RefHeading___Toc454978630)

[1.3 Outcome assessment 8](#__RefHeading___Toc454978631)

[2 Variables 10](#__RefHeading___Toc454978632)

[2.1 Screening variables 10](#__RefHeading___Toc454978633)

[2.2 Baseline variables 10](#__RefHeading___Toc454978634)

[2.3 Outcome variables 10](#__RefHeading___Toc454978635)

[2.4 Serious Adverse Events 11](#__RefHeading___Toc454978636)

[2.5 Effect modifiers 11](#__RefHeading___Toc454978637)

[3 Data analysis plan 12](#__RefHeading___Toc454978638)

[3.1 Analysis and unblinding of primary endpoint data 12](#__RefHeading___Toc454978639)

[3.2 Recruitment and representativeness of recruited patients 12](#__RefHeading___Toc454978640)

[3.3 Adherence to allocated intervention 12](#__RefHeading___Toc454978641)

[3.4 Loss to follow-up and other missing data 12](#__RefHeading___Toc454978642)

[3.5 Adverse event reporting 12](#__RefHeading___Toc454978643)

[4 Outcome analysis 12](#__RefHeading___Toc454978644)

[4.1 Main analysis of intervention differences 13](#__RefHeading___Toc454978645)

[4.1.1 Analysis of outcomes at 12 months 13](#__RefHeading___Toc454978646)

[4.2 Statistical considerations 14](#__RefHeading___Toc454978647)

[4.3 Compliance analysis: 15](#__RefHeading___Toc454978648)

[4.4 Cost-effectiveness analysis: 15](#__RefHeading___Toc454978649)

[4.5 Planned sub-group (moderator) analyses 16](#__RefHeading___Toc454978650)

[4.6 Additional secondary/mediation analyses 16](#__RefHeading___Toc454978651)

[5. Reference List 16](#__RefHeading___Toc454978652)

[6. Appendices: dummy tables 18](#__RefHeading___Toc454978653)

[6.1 Healthy Activity Program tables 18](#__RefHeading___Toc454978654)

[6.2 Counselling for Alcohol Problems tables 21](#__RefHeading___Toc454978655)

# 1. Description of the trial

The goal of PREMIUM, a **Pr**ogram for **E**ffective **M**ental Health **I**nterventions in **U**nder-resourced Health Syste**m**s, is to implement a psychological treatment (PT) development and evaluation methodology that will lead to effective PTs for mental disorders that are culturally appropriate, feasible, acceptable and affordable in under-resourced settings. Details are given in the protocol publication[^1^](#_ENREF_1). The trials were conducted in 10 primary health centres (PHC) in the North district of Goa, a state on the west coast of India. The publicly-funded PHC are the first port of call in India for people who wish to seek health care in the public system.

## Principal research objectives to be addressed

The objectives of the two trials are to evaluate the effectiveness and cost-effectiveness of the Healthy Activity Program (HAP), for adults with moderately severe to severe depression, and the Counselling for Alcohol Problems (CAP), for adults with harmful drinking (HD) or alcohol dependence (AD), delivered by the same pool of lay counsellors in primary care in Goa, India.

The primary hypotheses are that the PT intervention in addition to enhanced usual care (EUC) will be superior to EUC alone in reducing the severity of symptoms and in increasing remission rates in participants with depression and harmful drinking at 3 months post-enrolment.

Secondary hypotheses will be measured at 3 and 12 months; and include that the PT intervention will reduce disability and suicidal attempts in both trials; reduce intimate partner violence, and improve behavioural activation in the HAP trial; and reduce consequences of alcohol use, perpetration of violence and depression in the CAP trial. We will also assess cost-effectiveness from a health systems perspective. That is, it would have a gain in quality-adjusted life years of no more than the annual per capita gross domestic product in India. Further to this, from a societal perspective, the intervention will be dominant over EUC, with both a reduction in costs and superior outcomes.

In this document, we present the analysis plan for the outcomes assessed at 12 months and over the duration of the follow up. Analyses of 3 month outcomes has been completed and followed an analysis plan (PREMIUM statistical analysis plan V2, 17.12.2015) which was originally drafted to address both 3 and 12 months outcomes. However, following the observations of the analyses of the 3 month outcomes, modifications for the 12 month outcome analyses have been proposed, and agreed by the TSC and DSMB. This has necessitated this revised Statistical Analysis Plan Final - 12 months outcomes.

Key trial design details including randomisation, sample size estimation, duration of intervention period, enhanced usual care, window of follow-ups, and data management, are described in this earlier document.

## 1.2 Trial design

The PREMIUM trial is a parallel arm individually randomised controlled trial design with equal allocation of participants between arms to evaluate both the HAP and CAP interventions. Table 1 summarises the outcomes collected at 12 months, the source of data, and the analyses groups of interest.

The flow chart (Figure 1) shows the process of recruitment and follow-up of participants in the trial. Whereas the HAP trial includes participants of both genders, the CAP trial only includes male participants as HD/AD are rare in women in India.

**Table 1: 12 month outcomes, tools and analysis groups for** the PREMIUM trials

| **Outcome** | **Source of data** | | **Analysis group** |
| --- | --- | --- | --- |
|  | **Healthy Activity Program** | **Counselling for Alcohol Problems** |  |
| Severity of symptoms | Beck Depression Inventory-II (BDI-II) | Time Line Follow Back (TLFB) | Depression^a^, HD^a^, AD |
| Remission | Patient Health Questionnaire (PHQ-9) | Alcohol use disorders identification test (AUDIT) | Depression^a^, HD^a^, AD |
| Disability levels | WHO Disability Assessment Schedule (WHO-DAS) | | Depression, HD, AD |
| Costs of illness | Client Service Receipt Inventory (CSRI) | | Depression, HD, AD |
| Consequences of alcohol use | NA | Short inventory of problems (SIP) | HD, AD |
| Recovery | Patient Health Questionnaire (PHQ-9) | Alcohol use disorders identification test (AUDIT) | Depression, HD, AD |
| Relapse (full/partial) | Patient Health Questionnaire (PHQ-9) | NA | Depression |
| Severity of symptoms |
| Suicidal thoughts | PHQ-9 item 9 on suicidal thoughts. | | Depression, HD, AD |
| Suicide attempt | A single question on suicide attempt added at the end of the BDI-II | | Depression, HD, AD |
| Experience of intimate partner violence | Two questions on experience of intimate partner violence (psychological/emotional, physical) added at the end of the BDI-II | NA | Depression |
| Perpetration of intimate partner violence | NA | A single question on perpetration of intimate partner violence added at the end of the SIP | HD, AD |
| Uptake of detoxification services | NA | CSRI | AD |
| Minimal Clinically Important Difference (MCID) | Two questions on patient’s perception of change in status of general health and target disorder | NA | Depression |

^a^ Primary hypotheses. AD, alcohol dependence; HD, harmful drinking; NA, not applicable.

**Figure 1: PREMUM trials flow chart**

**Assessed for eligibility**

**N=**

**Females**

Eligible for screening

**Total screened: PHQ-9**

Excluded

(reasons)

PHQ-9 score >14

Refused

(reasons)

AUDIT score <12

PHQ-9 score >14

Refused

(reasons)

PHQ-9<15

**Consultation with PHC doctor**

Declined to participate

AUDIT score <12

PHQ-9 score <15

AUDIT score 12+

Any PHQ-9 score

**Males**

Eligible for screening

**Total screened: AUDIT>PHQ-9 AUDIT+PHQ-9**

**Consultation with PHC doctor**

Declined to participate

Allocated to treatment A

Allocated to treatment B

3-month FU

**Randomized**

12-month FU

3-month FU

**Informed consent**

12-month FU

Allocated to treatment A

Allocated to treatment B

3-month FU

**Randomized**

12-month FU

3-month FU

**Informed consent**

12-month FU

## 1.3 Outcome assessment

Outcome data was collected at 3 months and 12-months post-enrolment. The 3-month outcome is the primary endpoint for both trials as the PT delivery would be completed by then and we would expect the optimal effect of the treatment at this time-point.

The 12-month end-point is included to evaluate the sustainability of the effect of the intervention. The outcome assessments at 12-months are summarized in Table 2.

**Table 2: Instruments and individual-level 12-month outcome measures for the PREMIUM trial**

| **Instrument** | **Trial** | **Description** | **Outcome (per participant)** |
| --- | --- | --- | --- |
| Beck Depression Inventory-II | HAP | 20^1^-item questionnaire assessment of depressive symptoms assessed on a scale of 0 to 3. | BDI-II mean score |
| Patient Health Questionnaire (PHQ-9) | HAP | 9-item questionnaire assessment of depressive symptoms assessed on a scale of 0 to 3. | - PHQ-9 score (remission, recovery, mean score) |
| WHO Disability Assessment Scale (WHO-DAS) | HAP CAP | 12-item questionnaire for measuring functional impairment over the previous 30 days. In addition, two items assess number of days the person was unable to work in the previous 30 days. | - Total disability score - Days unable to work score; - Quality Adjusted Life Years; |
| Client Service Receipt Inventory (CSRI) | HAP CAP | Questionnaire to collect data on the utilisation and costs of health care and lost productivity (including that of care-givers). | Costs of illness (direct and indirect)  Use of detox services (only CAP) |
| Time Line Follow Back (TLFB) | CAP | Calendar tool supplemented by memory aids to obtain retrospective estimates of daily drinking over past 2 weeks | - Mean daily alcohol (gms) consumed in past 2 weeks - Abstinence in past 2 weeks - Heavy drinking in past 2 weeks |
| AUDIT | CAP | 10-item questionnaire with 3 questions on the amount and frequency of drinking, 3 questions on alcohol dependence, and 4 on problems caused by alcohol. | AUDIT score (remission, recovery) |
| Short Inventory of Problems (SIP) | CAP | 15-item questionnaire which assesses physical, social, intrapersonal, impulsive, and interpersonal consequences of alcohol consumption. | SIP score |
| ^2^Violence items | HAP CAP | Additional questions on the BDI-II and SIP on intimate partner violence (IPV) | - Experience of IPV (for HAP trial only) - Perpetration of IPV (for CAP trial only) |
| Suicidal thoughts | HAP  CAP | PHQ-9 item 9 (thoughts of suicide) | Suicidal thoughts |
| Suicide attempt | HAP  CAP | A single close-ended question on suicide attempt added at the end of the PHQ-9 | Suicide attempt |
| Minimal Clinically Important Difference (MCID) | HAP | A set of questions on a Likert scale administered to all trial participants at 12 months post-enrolment to assess how much the patient feels his/her health/social factors have changed. | MCID score in relation to mean/median primary outcome mean scores. |

^1^ Excluded sex item as considered culturally inappropriate in this setting

^2^ Gender disaggregated

# 2 Variables

## 2.1 Screening variables

The codebook is included in Appendix 1. Key variables are listed below

## 2.2 Baseline variables

*From refusers:*

- Age
- Gender
- Education
- Marital status
- Occupation
- Reason for refusal

*From consented participants:*

- Age
- Marital status
- Education
- Occupation
- PHQ-9 score
- AUDIT score (CAP trial only)
- Readiness to make changes in drinking (CAP trial)
- Patient’s expectation of usefulness of counselling (HAP and CAP trials)

## 2.3 Outcome variables

These are listed in Table 3 and below:

- BDI-II score (HAP trial only)
- PHQ-9 score
- Ethanol consumption in grams (CAP trial only)
- WHODAS score
- Experience of intimate partner violence (HAP trial only)
- Perpetration of intimate partner violence (CAP trial only)
- Client Service Receipt Inventory (CSRI)
- AUDIT score (CAP trial only)
- Minimal Clinically Important Difference (HAP trial only)

**Table 3: Analysis methods and variable names for the 12 month PREMIUM outcomes**

| **Trial** | **Outcome** | **Measure of outcome[[1]](#footnote-2)** | **Variable name** |
| --- | --- | --- | --- |
| **PRIMARY OUTCOMES** | |  |  |
| HAP | BDI-II | Mean score of 20 items | oe12_bditotal |
|  | PHQ-9 | Remission: % with PHQ-9 < 10 | oe12_phq9_totscore |
| CAP | Ethanol consumption | TLFB estimate of standard number of drinks consumed[[2]](#footnote-3)  TLFB estimate of percent days abstinent (PDA), and percent days heavy drinking (PDHD) (heavy drinking defined as >70g alcohol/day | oe12_tlfb_ethgm_daily_int (derived from oe12_tlfb_all_tot_std_drinks) |
|  | AUDIT | Remission: % with AUDIT < 8 | oe12_Audit_tot |
| **SECONDARY OUTCOMES** | |  |  |
| HAP | PHQ-9 | Recovery: % with PHQ-9 < 5 at both 3 and 12 months | oe12_phq9_totscore |
|  |  | Mean score of 9 items |  |
|  |  | ‘Partial’ relapse: % with PHQ-9 score range (10 to 14) |  |
|  |  | ‘Full’ relapse: % with PHQ-9 score>14 |  |
| CAP | AUDIT | Recovery: % with AUDIT< 8 at both 3 and 12 months | oe12_Audit_tot |
| Both | WHO-DAS | Mean WHODAS score  Mean number of days out of work from WHODAS | oe12_das_tot  oe12_das15_act_unable |
| Both | Costs of illness (direct & in-direct) | Costs of resources to deliver PT  Health care, patient, and family-borne costs from CSRI | Computed variables |
| CAP | Consequences of alcohol use | Mean Short Inventory of Problems (SIP) score | oe12_siptotal |
| Both | Suicidal thoughts | % with a suicide thoughts | oe12_phq9_deadhurt |
|  | Suicide attempt | % with a suicide attempt | oe12_suicide_attempt |
| HAP | ^1^Intimate partner violence | % with experience of i) psychological/emotional or ii) physical violence | oe12_inti_violence1; oe12_inti_violence2 |
| CAP | Partner violence | % reporting perpetration of i) psychological/emotional or ii) physical violence | oe12_perp_violence1  oe12_perp_violence2 |
| HAP | Minimal Clinical important difference | % reduction in clinical outcome scores from baseline based on ROC thresholds of patient’s reporting ‘feeling better’. | oe12_mcidgenhealthap; oe12_mcidtension |

## ^1^ Gender disaggregated

## 2.4 Serious Adverse Events

- Death
- Suicide attempt
- Unplanned hospitalisation from any cause

## 2.5 Effect modifiers/mediators

## Moderators:

- Gender (HAP trial only)
- Chronicity of illness (HAP trial only)
- Severity of depression (HAP trial only)
- Severity of drinking (AUDIT score-CAP trial only)

**Mediators:**

- Readiness to change drinking (CAP trial only)
- Behavioural activation (HAP trial only)

# 3 Data analysis plan

Analyses will follow CONSORT guidelines for parallel-group randomised trials[^2^](#_ENREF_2). Analyses will be conducted in Stata version 13. Do-files will be prepared based on blinded data, and data will not be unblinded until the dataset is finalised and locked.

## 3.1 Analysis and unblinding of primary endpoint data

Analyses for primary endpoint outcomes has been completed (and manuscripts submitted for publication), following recommendations from the TSC/DMSC on 24^th^ July 2015.

## 3.2 Recruitment and representativeness of recruited patients

Initial analyses will compare baseline characteristics of individuals who did and did not complete 12-month outcome assessments, compared to individuals who completed 3-months outcome. A CONSORT flow chart will be constructed (Figure 1). This will include the number of eligible participants, number of participants agreeing to enter the trial, number of participants refusing and reasons, then by intervention arm: the number of participants allocated to each arm, the number seen at 3 months and 12 months respectively.

## 3.3 Adherence to allocated intervention

The following treatment variable will be summarized in the PT arms.

- the quantity/coverage of the active treatment (PT) as indicated by number of sessions by treatment completers and dropouts,

## 3.4 Loss to follow-up and other missing data

The numbers and proportions actively withdrawing from the trial and passively lost to follow-up will be reported overall and within intervention arm at 12 months. The data for those lost to follow-up will be used in the CONSORT flow chart. The reasons for withdrawal from the trial will be summarised.

## 3.5 Adverse event reporting

Serious adverse events (SAE) will be summarised (proportion of individuals with each type of SAE, and total number of SAEs) by arm. If there are a sufficient number of these, the risks and 95% CIs will be reported and the risks will be compared between intervention arms.

# 4 Outcome analysis

Stata will be used for data description and the main inferential analysis. The primary analyses will be intention-to-treat (modified to adjust for baseline values of the outcome measure and PHC) with imputation of missing outcome data

PHC will be adjusted for as a fixed effect in the analysis to allow for within-PHC clustering.

## 4.1 Main analysis of intervention differences

For each trial, the main statistical analyses will estimate the difference in mean outcome (BDI-II or ethanol consumption) between patients randomised to PT and EUC by intention-to-treat at the 12-month post-randomisation observation time point. The intervention effect will be reported as adjusted mean differences with 95% confidence intervals (CI).

For the binary outcomes with high prevalence, the intervention effect will be reported as the prevalence ratio estimated using the marginal standardisation technique with 95%CI for the prevalence ratios estimated using the delta method[^3^](#_ENREF_3). Differences in prevalence and 95%CI will also be reported. The intervention effect for rare outcomes will be reported as the odds ratio.

### **4.1.1 Analysis of outcomes at 12 months**

**i) HAP trial**

A linear mixed effects model with primary continuous outcome of BDI-II total score at 12 months with intervention arm as a covariate and adjusting for PHQ-9 score at baseline (BDI-II score was not collected at baseline) and PHC.

Logistic regression analysis using a logistic mixed effects model with proportion with PHQ-9<10 (remission) at 12 month as the binary primary outcome, with intervention arm as a covariate, and adjusting for PHQ-9 score at baseline and PHC.

**ii) CAP trial**

An appropriate model (e.g. zero-inflated negative binomial (ZINB)) will be used to analyse the primary continuous outcome of ethanol consumption in grams at 3 and 12 months months with intervention arm as a covariate and adjusting for baseline AUDIT score and PHC. The ZINB model is a mixture model which fits two parameters – the probability of a ‘zero’ outcome (no drinking reported), and the mean ethanol consumption among drinkers.

Logistic regression analysis using a logistic mixed effects model with proportion with AUDIT<8 (remission) as the binary primary outcome, with intervention arm as a covariate and adjusting for baseline AUDIT score and PHC.

The models may also include covariates that are associated with missing data. This is to increase the plausibility of the missing at random assumption that is made when fitting the models described above.

The analysis of the secondary outcomes will be similar to those done for the primary outcomes. For normally distributed continuous outcomes, the main statistical analyses will estimate the difference in mean outcome between patients randomised to PT and EUC by intention-to-treat at the 12-month post-randomisation observation time point. The intervention effect will be reported as adjusted mean differences, with 95% confidence intervals (CI).

For the binary outcomes, the intervention effect will be reported as the prevalence ratio estimated using the marginal standardisation technique with 95%CI for the prevalence ratios estimated using the delta method[^3^](#_ENREF_3). Differences in prevalence and 95%CI will also be reported.

Given that we have only two follow-up time points (3 & 12 months), repeated measures analysis will also be conducted, including analysis of change over time within each of the end-points. The repeated measures analysis will include a treatment by time interaction term to allow for a different intervention effect at 3 vs 12 months, although this will not be highly powered.

Separate analyses will be conducted for the sub-group of alcohol dependent patients, and will be similar to those done for the main trial outcomes, however these will be reported separately in order to be consistent with the published trials protocol.

No interim analyses of outcomes are planned.

## 4.2 Statistical considerations

*Stratification and clustering*

PHC clinic will be included as a fixed-effect covariate in all regression models. A sensitivity analysis will be conducted adjusting for PHC as a random effect.

*Adjustment for multiple outcomes and reporting p-values*

Interpretation of the intervention effect will be based on the strength of evidence of effect size and consistency of results for related outcomes.

*Missing baseline data*

The number of participants with complete data will be reported and missing values will either be imputed using an appropriate method as per the recommendations of White and Thompson[^4^](#_ENREF_4), such as mean imputation.

*Missing outcome data*

Missing outcome data will be imputed using multiple imputation, implemented in Stata.

*Model assumption checks*

The models assume normally distributed outcomes; this will be checked when describing the data. Model residuals will also be plotted to check for normality and inspected for outliers. If substantial departures from normality occur, transformations will be considered. If a suitable transformation cannot be found, a non-parametric analysis will be considered.

A sensitivity analysis that assesses the effect of deviations from the missing at random assumption on the intention to treat treatment differences for the primary outcomes may be considered if there are considerable amounts of missing data[^3^](#_ENREF_3). Sensitivity analyses will also include adjustment for counsellor as a random effect and complete case analyses.

## 4.3 Compliance analysis:

As we expect a proportion of our participants to have poor compliance to the PTs, we will in addition undertake Complier’s Average Causal Effect analyses, which estimates the effect of the PT on the participants who received it in full as intended by the original randomisation[^5^](#_ENREF_5). CACE is a measure of the causal effect of a treatment or intervention on the people who received it as intended by the original group allocation. Because it retains the initial randomized assignment, it overcomes the problems related to per-protocol and on-treatment analyses. The CACE analysis makes two assumptions – the first is that the members of the EUC arm in each trial have the same probability of non-compliance to the PT intervention as do members of the PT arm. The second assumption is that being offered the intervention has no effect on the outcomes. We assume that the same proportion of participants in the EUC arm would adhere to the intervention as in the PT arm, and assume that the outcomes in the hypothetical non-compliers would be the same as in the non-compliers in the PT arm. This enables us to estimate the outcome prevalence/mean in the hypothetical compliers in the EUC arm, and hence the intervention effect among compliers.

The numbers not treated will be given by arm. Not treated will be those who were randomised to PT but did not have any sessions. Compliance will be treatment resulting in a planned discharge excluding referrals to specialists.

## 4.4 Cost-effectiveness analysis:

Direct costs of the PT will be estimated by estimating resources required to deliver each component. Relevant monetary unit costs will then be attached to these resources. Costs of delivery will be estimated and applied to each individual based on the process indicators which reflect the actual uptake of the treatment. Other health care costs and other patient- or family-borne costs will be computed and compared at 3 and 12 months, and subsequently related to changes in principal health outcomes collected at those time points. Changes in principal outcomes at 3 months will be compared with changes in costs to calculate incremental cost effectiveness ratios. Changes in WHO-DAS scores at 3 and 12 months will be converted into utility scores to generate incremental cost utility ratios. The economic analysis will be conducted from the perspective of payers, as well as families and society. The economic costs of some secondary outcome data can be included in the economic analysis, using appropriate proxy mean wage rates, including agrarian wage rates, to estimate time out of usual activity. Indian estimates of the value of statistical life will be applied to estimate the costs of completed suicides or suicide attempts. In addition, the costs of implementing the intervention at the PHC level, costs of recovery, and costs of developing the treatments will be estimated.

In the event that dominance is not shown, i.e. the intervention is more effective but the costs are also more than the usual care group, incremental cost-effectiveness ratios will be computed, together with their confidence intervals (using bootstrapping techniques to overcome expected skewness of cost data). Cost-effectiveness acceptability curves will also be derived in order to show the probability of any cost-effective advantages for the psychological treatment at a range of 'willingness to pay' threshold levels. In further sensitivity analyses, decision modelling will be used to identify key threshold points at which PT becomes cost effective and potentially cost saving. Examples of parameters influencing thresholds may include levels of effectiveness, costs and uptake of intervention, as well as the perspective of the analysis. These analyses will also vary assumptions around key identified moderators to look at their impact on cost effectiveness.

## 4.5 Planned sub-group (moderator) analyses

A moderator analysis will be conducted to help clarify on whom and under what circumstances (moderators) the PT treatment works. We will assess modification of treatment effect by *a-priori* defined modifiers (gender, chronicity of illness (for HAP only), readiness to change (for CAP only), and baseline severity (for HAP & CAP) by fitting appropriate interaction terms and testing for heterogeneity of treatment effects in regression models.

## 4.6 Additional secondary/mediation analyses

Additional secondary analyses will be conducted to answer 4 key questions (below). The analysis will involve assessing the following:

1. The relationship between treatment completion/dosage and trial clinical outcomes using simple linear regression; (HAP and CAP Trials)
2. Assess sudden gains effects, as well as early response to treatment, and if these gains and early response are associated with sustained clinical outcomes, using linear regression and independent samples t-test. Analyses will be conducted separately and then compared to determine whether there is a difference in their influence on 3 and 12 month depression outcomes. These concepts are typically defined in different ways with the criteria for sudden gains being pre-determined using specific criteria between select sessions[^6^](#_ENREF_6); and early response being defined after the course of treatment based on response curves[^7^](#_ENREF_7); (HAP Trial only)
3. A mediation analysis will be conducted to help clarify the mechanisms (the *why*) of sustained treatment effect. We will assess mediation of treatment outcome by *a-priori* defined mediators (behavioural activation[^8^](#_ENREF_8) (for HAP), and readiness to change drinking[^9^](#_ENREF_9) (for CAP) assessed at 3 months, by applying MacKinnon’s approach to mediation analysis[^10^](#_ENREF_10) (HAP and CAP Trials).
4. Personalized advantage Index to estimate who are the best patients for the CAP and HAP treatment (HAP and CAP Trials).

# 5. Reference List

1. Patel V, Weobong B, Nadkarni A, et al. The effectiveness and cost-effectiveness of lay counsellor-delivered psychological treatments for harmful and dependent drinking and moderate to severe depression in primary care in India: PREMIUM study protocol for randomized controlled trials. Trials 2014;15:101.

2. Schulz KF, Altman DG, Moher D, Group C. CONSORT 2010 Statement: updated guidelines for reporting parallel group randomised trials. BMC medicine 2010;8:18.

3. Localio AR, Margolis DJ, Berlin JA. Relative risks and confidence intervals were easily computed indirectly from multivariable logistic regression. J Clin Epidemiol 2007;60:874-82.

4. White IR, Thompson SG. Adjusting for partially missing baseline measurements in randomized trials. Stat Med 2005;24:993-1007.

5. Hewitt CE, Torgerson DJ, Miles JN. Is there another way to take account of noncompliance in randomized controlled trials? CMAJ 2006;175:347.

6. Tang TZ, Derubeis RJ, Hollon SD, Amsterdam J, Shelton R. Sudden gains in cognitive therapy of depression and depression relapse/recurrence. Journal of consulting and clinical psychology 2007;75:404-8.

7. Grilo CM, White MA, Wilson GT, Gueorguieva R, Masheb RM. Rapid response predicts 12-month post-treatment outcomes in binge-eating disorder: theoretical and clinical implications. Psychol Med 2012;42:807-17.

8. Ekers D, Webster L, Van Straten A, Cuijpers P, Richards D, Gilbody S. Behavioural activation for depression; an update of meta-analysis of effectiveness and sub group analysis. PLoS One 2014;9:e100100.

9. Stein LA, Minugh PA, Longabaugh R, et al. Readiness to change as a mediator of the effect of a brief motivational intervention on posttreatment alcohol-related consequences of injured emergency department hazardous drinkers. Psychol Addict Behav 2009;23:185-95.

10. MacKinnon DP, Fairchild AJ, Fritz MS. Mediation analysis. Annu Rev Psychol 2007;58:593-614.

# 6. Appendices: dummy tables

## 6.1 Healthy Activity Program tables

**Table 1. Baseline characteristics of completers of outcome evaluation and those lost to follow-up (LTFU)**

|  | **Lost before 3 month evaluation**  **(n=)** | **Completed 3 month outcome evaluation (n=)** | **Completed 12 month outcome evaluation (n=)** |
| --- | --- | --- | --- |
| Age (years) (mean [SD]) |  |  |  |
| **Gender** (Female) (n [%]) |  |  |  |
| **Marital status** (n [%])  Married  Single  Separated/Divorced  Widow |  |  |  |
| **Education status (n [%])**  None  Primary  Secondary  Higher Secondary  Graduate/above |  |  |  |
| **Occupation** (n [%])  Unemployed  Unskilled manual labour  Skilled manual labour  Clerical & professional  Others |  |  |  |
| **Patient’s expectation of usefulness of counselling** (n [%])  Not useful  A little/somewhat useful  Moderately useful  Very useful |  |  |  |
| **Median PHQ score** (median [IQR]) |  |  |  |
| **PHQ category** (n [%])  Score 15-19 (Moderately severe)  Score 20-27 (severe) |  |  |  |

**Table 2**. Baseline characteristics of trial participants by arm

|  | **EUC (n=)** | **HAP & EUC (n=)** |
| --- | --- | --- |
| Age (years) (mean [SD]) |  |  |
| **Gender** (Female) (n [%]) |  |  |
| **Marital status** (n [%])  Married  Single  Separated/Divorced  Widow |  |  |
| **Education status** (n [%])  None  Primary  Secondary  Higher Secondary  Graduate/above |  |  |
| **Occupation** (n [%])  Unemployed  Unskilled manual labour  Skilled manual labour  Clerical & professional  Others |  |  |
| **Patient’s expectation of usefulness of counselling** (n [%])  Not useful  A little/somewhat useful  Moderately useful  Very useful |  |  |
| **Median PHQ score** (median [IQR]) |  |  |
| **PHQ category** (n [%])  Score 15-19 (Moderately severe)  Score 20-27 (severe) |  |  |

**Table 3. Process indicators for trial participants** in the PT (intervention) arm

| **Process indicator** | **Total** (n [%]) | |
| --- | --- | --- |
| Number of participants who entered PT arm |  | |
| Number of PT sessions received (n=) | Planned discharge (completers) (n=) | Unplanned discharge(drop-out) (n=) |
| 0  1-4  5-6  7-8 |  |  |
| Mean (95% CI) |  |  |

**Table 4. Intervention effect on outcomes at 12 months**

| **Outcome** | **HAP arm**  **(n=)** | **EUC arm**  **(n=)** | **Adjusted mean difference or prevalence ratio (95% CI)** | **p-value** |
| --- | --- | --- | --- | --- |
| **Primary** |  |  |  |  |
| Mean BDI-II score |  |  |  |  |
| Remission: PHQ-9<10- no. (%) |  |  |  |  |
| **Secondary** |  |  |  |  |
| Recovery: PHQ-9<5 at 3 and 12 months- no. (%) |  |  |  |  |
| Mean PHQ-9 score |  |  |  |  |
| Full relapse: PHQ-9 score>14; no. (%) |  |  |  |  |
| Partial relapse: PHQ-9 score>9<15; no. (%) |  |  |  |  |
| Mean disability score (SE) |  |  |  |  |
| Mean days unable to work (SE) |  |  |  |  |
| Suicide thoughts or attempts – no. (%)* |  |  |  |  |
| Intimate partner physical violence** – females no. (%) |  |  |  |  |
| Intimate partner psychological/emotional violence** – females no. (%) |  |  |  |  |
| Intimate partner psychological/emotional violence** – males no. (%) |  |  |  |  |
| Mean or median MCID score  Patient overall impressions of participating in trial- no. (%) |  |  |  |  |

* Suicidal thoughts over the past two weeks were assessed through the relevant PHQ-9 item while suicide attempts were assessed over the 3-month period since enrolment.

** Among married participants.

**Table 5. 12 month clinical outcomes by effect modifiers: adjusted* BDI-II and PHQ-9 scores at 12** months

|  | **EUC**  **(mean [SD])** | **HAP + EUC**  **(mean [SD])** | **Intervention effect: adjusted mean difference [95% CI; p value])** |
| --- | --- | --- | --- |
| **Gender** | | | |
| Male |  |  |  |
| Female |  |  |  |
| **Chronicity of depression** | | | |
| Median chronicity score (median [IQR]) |  |  |  |
| **Baseline PHQ-9 score** | |  |  |
| 15-19 |  |  |  |
| >=20 |  |  |  |
| *Adjusted for PHC as a fixed effect and baseline PHQ-9 as appropriate | | | |

**Table 6: Mediation effect of Behavioural Activation (BA) (parameter estimates and standard errors)**

| **Effect** | **Estimate** | **SE** | **95%Boostrap** |
| --- | --- | --- | --- |
| (c) Intervention 🡪 PHQ-9/BDI-II |  |  |  |
| (a) Intervention arm 🡪 Behavioural Activation |  |  |  |
| (b) Behavioural Activation 🡪 PHQ-9/BDI-II |  |  |  |
| axb |  |  |  |

## 6.2 Counselling for Alcohol Problems tables

**Table 1. Baseline characteristics of completers of outcome evaluation and those lost to follow-up (LTFU)**

|  | **Lost before 3 month evaluation**  **(n=)** | **Completed 3 month outcome evaluation (n=)** | **Completed 12 month outcome evaluation (n=)** |
| --- | --- | --- | --- |
| Age (years) (mean [SD]) |  |  |  |
| **Gender** (Male) (n [%]) |  |  |  |
| **Marital status** (n [%])  Married  Single  Separated/Divorced  Widow |  |  |  |
| **Education status** (n [%])  None  Primary  Secondary  Higher Secondary  Graduate/above |  |  |  |
| **Occupation** (n [%])  Unemployed  Unskilled manual labour  Skilled manual labour  Clerical & professional  Others |  |  |  |
| **Median AUDIT score** (median [IQR]) |  |  |  |
| **AUDIT category** (n [%])  Score 12-19  Score 20-40 |  |  |  |
| **Readiness to make changes in drinking** (n [%])  Not at all  A little/somewhat ready  Moderately ready  Already trying to change |  |  |  |
| **Patient’s expectation of usefulness of counselling** (n [%])  Not useful  A little/somewhat useful  Moderately useful  Very useful |  |  |  |

**Table 2. Baseline characteristics of trial participants by arm (primary analysis group)**

|  | **EUC (n=)** | **CAP & EUC (n=)** |
| --- | --- | --- |
| Age (years) (mean [SD]) |  |  |
| **Gender** (Male) (n [%]) |  |  |
| **Marital status** (n [%])  Married  Single  Separated/Divorced  Widow |  |  |
| **Education status** (n [%])  None  Primary  Secondary  Higher Secondary  Graduate/above |  |  |
| **Occupation** (n [%])  Unemployed  Unskilled manual labour  Skilled manual labour  Clerical & professional  Others |  |  |
| **Median AUDIT score** (median [IQR]) |  |  |
| **AUDIT category** (n [%])  Score 12-19  Score 20-40 |  |  |
| **Readiness to make changes in drinking** (n [%])  Not at all  A little/somewhat ready  Moderately ready  Already trying to change |  |  |
| **Patient’s expectation of usefulness of counselling** (n [%])  Not useful  A little/somewhat useful  Moderately useful  Very useful |  |  |

**Table 3. Process indicators for trial participants in the PT (intervention) arm**

| **Process indicator** | **Total** (n [%]) | |
| --- | --- | --- |
| Number of participants who entered PT arm |  | |
| Number of PT sessions received (n=) | Planned discharge (completers) (n=) | Unplanned discharge(drop-out) (n=) |
| 0  1-2  3-4 |  |  |
| Mean (95% CI) |  |  |

**Table 4. Intervention effect on outcomes at 12 months**

| **Outcome** | **CAP arm^1^**  **(n=)** | **EUC arm^1^**  **(n=)** | **Intervention effect (95% CI)^2^** | **p-value** |
| --- | --- | --- | --- | --- |
| **Primary** |  |  |  |  |
| Remission (AUDIT<8) (n [%]) |  |  |  |  |
| Daily standard ethanol consumed in the past 14 days^3^ |  |  |  |  |
| - Non-drinkers (n [%]) |  |  |  |  |
| - Ethanol consumption among drinkers (g) (mean (SD)) |  |  |  |  |
| **Secondary** |  |  |  |  |
| Recovery (AUDIT<8 at 3 and 12 months (n [%]) |  |  |  |  |
| Percent of days abstinent (n [%]) |  |  |  |  |
| Percent days of heavy drinking (n [%]) |  |  |  |  |
| Short inventory of problems (SIP) (mean (SD)) |  |  |  |  |
| WHO-DAS score (mean (SD)) |  |  |  |  |
| Days unable to work^3^  - None (n [%])  - Days unable to work when >1 day reported (mean (SD)) |  |  |  |  |
| Number of suicide attempts (n [%]) |  |  |  |  |
| Perpetration of intimate partner violence^4^ (n [%]) |  |  |  |  |

^1^ Among those with observed data at 3 and/or 12 months

^2^ Including imputed outcome data for those with missing data

^3^ Analysed with a zero-inflated negative binomial model which fits two parameters in one model i.e. the proportion with response of zero (e.g. no drinking in 14 days; or no days unable to work), and the mean count (e.g. ethanol consumption or days unable to work) among people with a non-zero (positive) response

^4^ Among married participants only

**Table 5. 12 month clinical outcomes by effect modifiers: adjusted* AUDIT and TLFB scores at 12** months

|  | **EUC**  **(mean [SD])** | **CAP + EUC**  **(mean [SD])** | **Intervention effect: adjusted mean difference [95% CI; p value])** |
| --- | --- | --- | --- |
| **Readiness to change at baseline/3 month outcome?** | | | |
| No. (%) in each category |  |  |  |
| **Patient expectations** | | | |
| No. (%) in each category |  |  |  |
| **Baseline AUDIT score** | |  |  |
| 12-14 |  |  |  |
| 15-19 |  |  |  |
| *Adjusted for PHC as a fixed effect | | | |

**Table 6: Mediation effect of Readiness to Change (parameter estimates and standard errors)**

| **Effect** | **Estimate** | **SE** | **95%Boostrap** |
| --- | --- | --- | --- |
| (c) Intervention 🡪 Alcohol Consumption |  |  |  |
| (a)Intervention arm 🡪 Readiness to Change |  |  |  |
| (b) Readiness to Change 🡪 Alcohol Consumption |  |  |  |
| axb |  |  |  |

1. Comparison of means assumes normally distributed outcomes. If substantial departures from normality occur, transformations or other models will be considered. [↑](#footnote-ref-2)
2. If, as anticipated, the distribution is a highly skewed distribution with an excess of zeros we will analyse with the zero-inflated negative binomial model which estimates two parameters – the proportion reporting zero drinks, and the mean alcohol content among those reporting alcohol use. [↑](#footnote-ref-3)
